# Bifunctional iminophosphorane catalysed enantioselective sulfa-Michael addition of alkyl thiols to alkenyl benzimidazoles†
†Electronic supplementary information (ESI) available. CCDC 1833189. For ESI and crystallographic data in CIF or other electronic format see DOI: 10.1039/c8sc01804a


**DOI:** 10.1039/c8sc01804a

**Published:** 2018-07-23

**Authors:** Michele Formica, Geoffroy Sorin, Alistair J. M. Farley, Jesús Díaz, Robert S. Paton, Darren J. Dixon

**Affiliations:** a Department of Chemistry , Chemistry Research Laboratory , University of Oxford , Mansfield Road , Oxford OX1 3TA , UK . Email: darren.dixon@chem.ox.ac.uk; b Faculté des Sciences Pharmaceutiques et Biologiques , Unité CNRS UMR 8638 COMETE , Paris Descartes University , Sorbonne Paris Cité , 4 Avenue de l'Observatoire , 75270 Paris Cedex 06 , France; c Departamento de Química Orgánica , Universidad de Extremadura , Avda. Universidad , s/n, 10003 Cáceres , Spain; d Department of Chemistry , Colorado State University , Fort Collins , Colorado 80523 , USA

## Abstract

The first enantioselective sulfa-Michael addition of alkyl thiols to alkenyl benzimidazoles, enabled by a bifunctional iminophosphorane (BIMP) organocatalyst, is described. The iminophosphorane moiety of the catalyst provides the required basicity to deprotonate the thiol nucleophile while the chiral scaffold and H-bond donor control facial selectivity. The reaction is broad in scope with respect to the thiol and benzimidazole reaction partners with the reaction proceeding in up to 98% yield and 96 : 4 er.

## Abstract 2

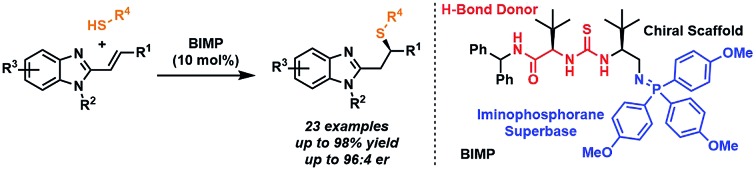
The first enantioselective sulfa-Michael addition of alkyl thiols to alkenyl benzimidazoles, enabled by a bifunctional iminophosphorane (BIMP) organocatalyst, is described.

## 

N-Containing heterocycles are ubiquitous motifs in both biologically active molecules and natural products. Their functionalization, especially when performed in an enantioselective manner, is therefore of particular interest in the field of organic synthesis. Alkenyl azaarenes have been used extensively as synthetic precursors for the functionalization of N-containing heterocycles.^1^ The electron deficiency of the aromatic ring, part-activates the conjugated alkene towards Michael-type additions,^2^ allowing for the rapid generation of molecular complexity. Most recently, the groups of Harutyunyan, Terada and Meng reported elegant, highly enantioselective Michael additions to alkenyl N-heterocycles employing organocuprates,^3^ pyrazoles^4^ and B_2_(pin)_2_ 5 respectively.

Our research has focused on developing enantioselective methods utilizing novel bifunctional iminophosphorane (BIMP) organocatalysts,^6^ which combine a chiral H-bond donor scaffold^7^ with an organo-superbase.^8^ More specifically, BIMP catalysis has been employed in the enantioselective addition of thiols^9^,^10^ to unactivated esters.6c,g This encouraged us to consider replacing the enoate electrophile with isoelectronic alkenyl benzimidazoles in order to access complex, chiral drug-like scaffolds with perfect atom economy and potential applications to medicinal chemistry (Fig. 1).^11^ To the best of our knowledge, there have been no reports to date of the enantioselective base catalysed Michael additions to alkenyl benzimidazoles^12^ and herein we wish to report our work leading to the first example, under BIMP catalysis.

**Fig. 1:**
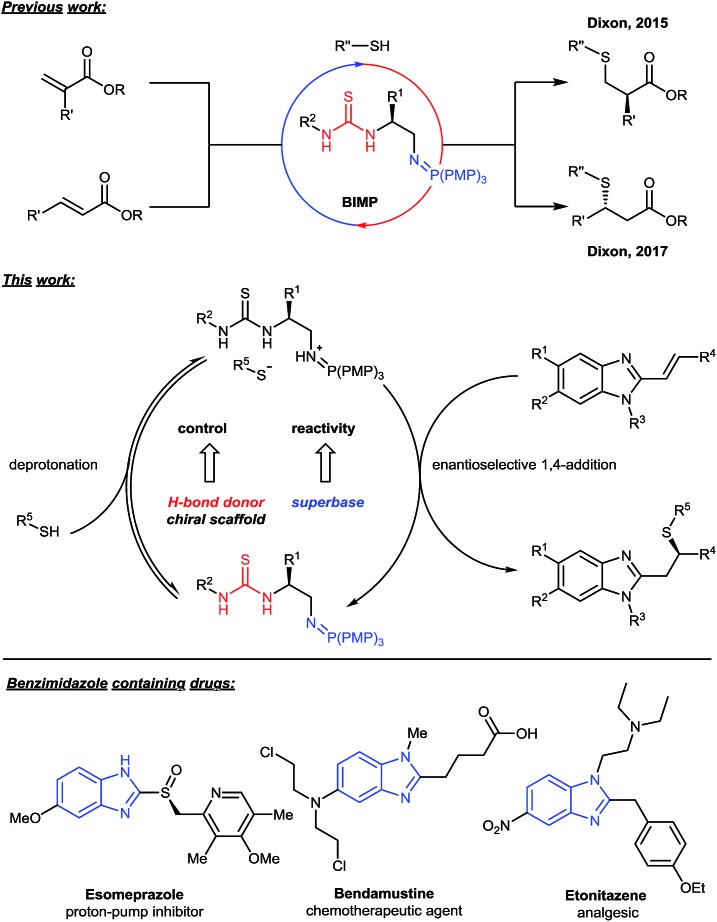
(Top) Previous BIMP catalysed sulfa-Michael additions to unactivated esters and current application to conjugated alkenyl benzimidazoles with a plausible catalytic cycle. (Bottom) Selected relevant benzimidazole containing drugs. PMP = *para*-methoxyphenyl.

We chose the readily prepared^4^ (*E*)-2-propenyl-1-tosyl-benzimidazole **1** and commercially available 1-propanethiol as model coupling partners to investigate reactivity and selectivity with a selection of bifunctional Brønsted base/H-bond donor catalysts using 3 eq. of thiol at 0.5 M concentration in THF at 22 °C for 24 hours (Fig. 2, Table 1). Quinidine derived catalyst **A** (entry 1) only provided **2** in 12% yield and a negligible 53 : 47 er. We therefore chose to investigate the more basic and more active BIMP catalysts in this reaction and were very pleased to find that known BIMP catalyst **B**6*a* bearing one stereocenter provided desired product **2** in 80% yield and 83 : 17 er (entry 2). With significant catalyst-enabled reactivity and stereocontrol identified we then proceeded to investigate second generation catalyst **C**6*g* which provided **2** in improved yield and er at 92% and 86 : 14 respectively (entry 3). Shifting the thiourea moiety further away from the iminophosphorane (**D–E**)6*d* showed no improvement in er over **B** (entries 4 and 5). We therefore focused on exploring catalysts built around the same chiral scaffold as **C**. Catalyst **F**6*g* bearing ^*t*^Bu groups at both stereocenters in the (*S*,*S*) configuration afforded **2** in 90% yield and 90 : 10 er (entry 6). Interestingly, a control reaction without any catalyst was found to go to completion (entry 7), indicating that an uncatalysed background reaction^13^ pathway was leading to an erosion in the enantiomeric ratio of the product. To suppress this background reactivity, the reaction was diluted to [0.06 M], cooled to 0 °C and only 1.2 eq. of thiol were used. The new set of conditions, combined with a solvent switch from THF to Et_2_O, provided **2** in 93% yield and 94 : 6 er using catalyst **F** (entry 8). Surprisingly a further decrease of the temperature to –40 °C led to an erosion of the enantiomeric ratio (entry 9). To further boost the enantiomeric ratio, diastereoisomeric catalyst **G**6*g* was screened. Pleasingly, catalyst **G** outperformed corresponding diastereomer **F** affording the desired product in 98% yield and 95 : 5 er (entry 10).

**Fig. 2:**
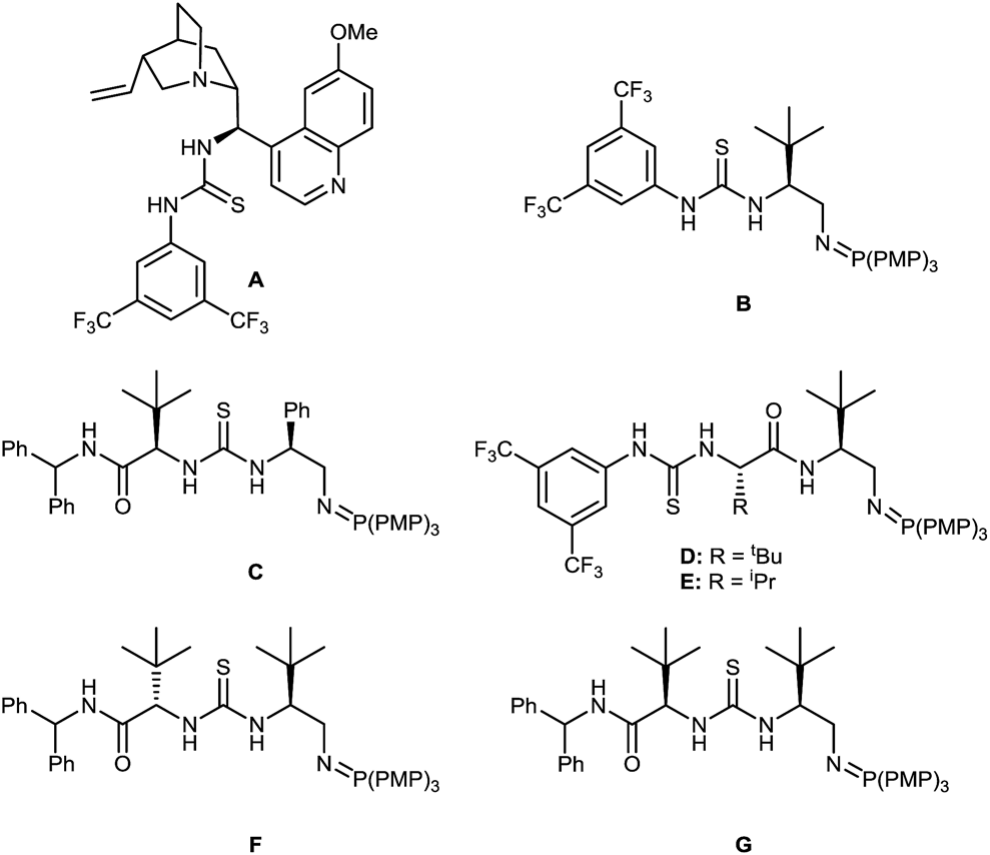
Selected BIMP catalysts investigated for the optimization of the sulfa-Michael addition. PMP = *para*-methoxyphenyl.

**Table 1:** Reaction optimization. Full optimization data available in the ESI

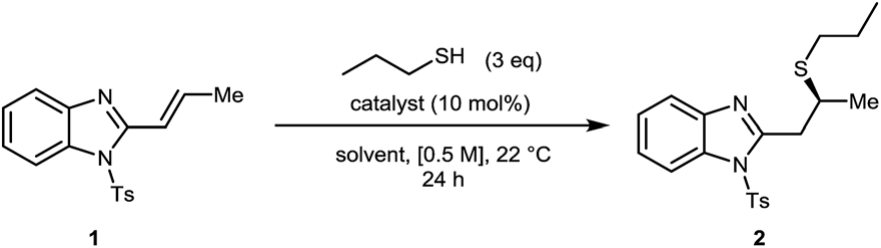				
Entry	Catalyst	Solvent	Yield^*a*^ (%)	er^*b*^
1 (ref. 14)	**A**	THF	12	53 : 47
2	**B**	THF	80	83 : 17
3^*c*^	**C**	THF	92	86 : 14
4	**D**	THF	83	66 : 34
5	**E**	THF	95	83 : 17
6	**F**	THF	90	90 : 10
7^*d*^	None	THF	95	50 : 50
8^*e*^	**F**	Et_2_O	93	94 : 6
9^*f*^	**F**	Et_2_O	88	82 : 18
**10** ^*e*^	**G**	****Et****_**2**_ ****O****	**98**	**95** **:** **5**

^*a*^Isolated yield.

^*b*^Determined by HPLC analysis on a chiral stationary phase.

^*c*^Reaction carried out at 0.25 M concentration.

^*d*^Reaction carried out over 72 h.

^*e*^Reaction carried out at 0 °C, using 1.2 eq. of thiol and 0.06 M concentration.

^*f*^Reaction carried out at –40 °C, using 1.2 eq. of thiol and 0.06 M concentration.

With optimal conditions established, we proceeded to explore the scope and limitations of this transformation (Scheme 1). Initially the steric and electronic properties of the thiol nucleophile were varied. Higher order linear, branched and cyclic alkyl substituents on the thiol all provided the corresponding Michael adducts (**3–5**) with high yields and enantioselectivities. The introduction of a phenyl ring was well tolerated providing **6** in outstanding yield and good er. Appending a silyl group to the thiol nucleophile showed no detrimental effect providing **7** in excellent yield and er. Benzyl thiols provided corresponding Michael adducts **8–10** in high yields in all cases and good enantioselectivity, albeit slightly diminished when compared to simpler alkyl thiols.^15^,^16^

**Scheme 1:**
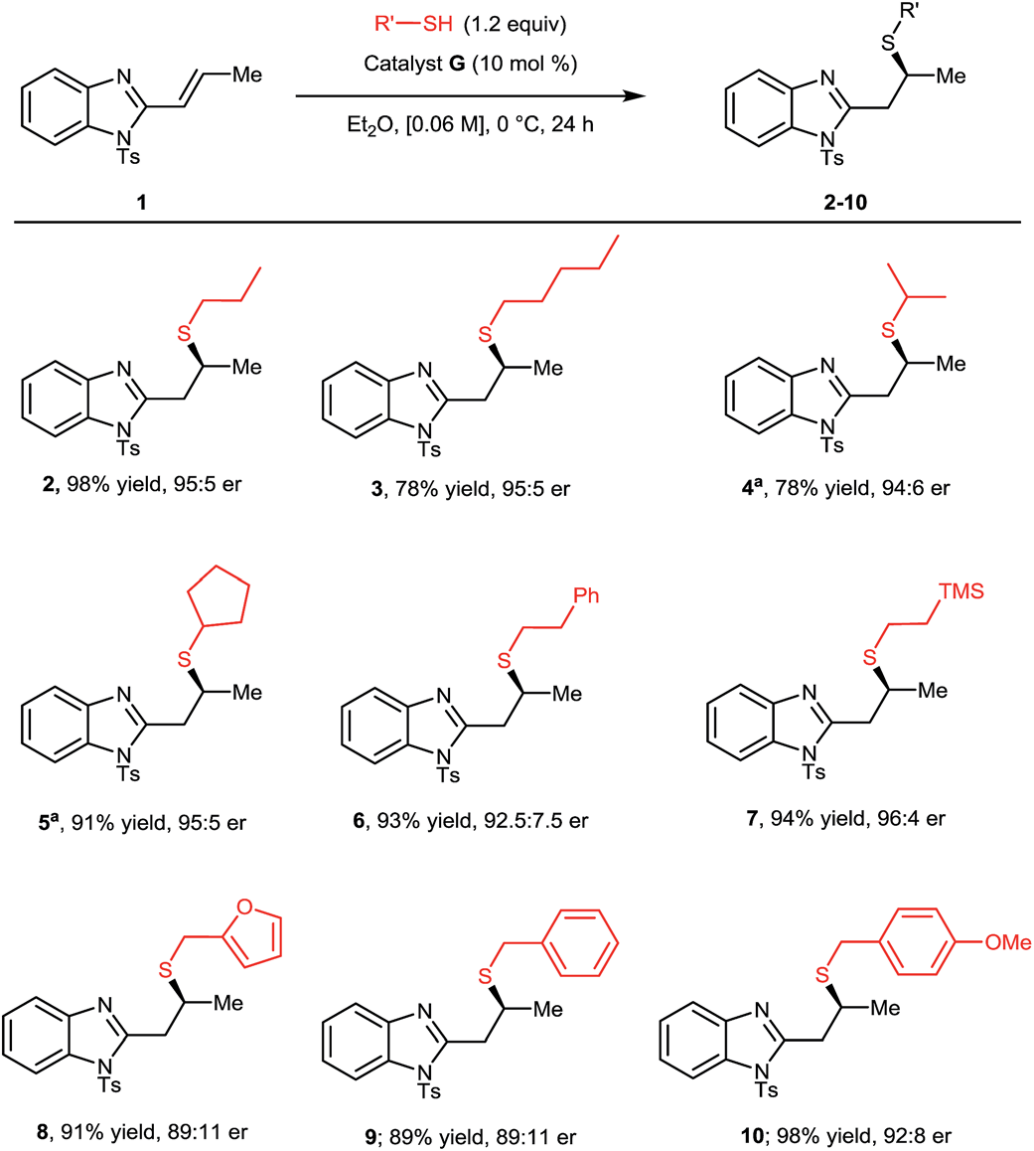
Scope of the thiol coupling partner. ^a^Reaction carried out at 22 °C.

Having investigated the thiol component, we then focused on substituent effects on the benzimidazole core (Scheme 2). Variations to the phenyl backbone did not affect reactivity, disubstitution at C5 and C6 with methyl groups afforded corresponding adduct **11** in 81% yield and 86 : 14 er. Alternating monosubstitution between C5 and C6 did not have a large effect, with bromine containing substrates affording the corresponding Michael adducts (**12**, **13**) in greater than 75% yield and 86 : 14 er allowing for potential further functionalization at both positions.^17^

**Scheme 2:**
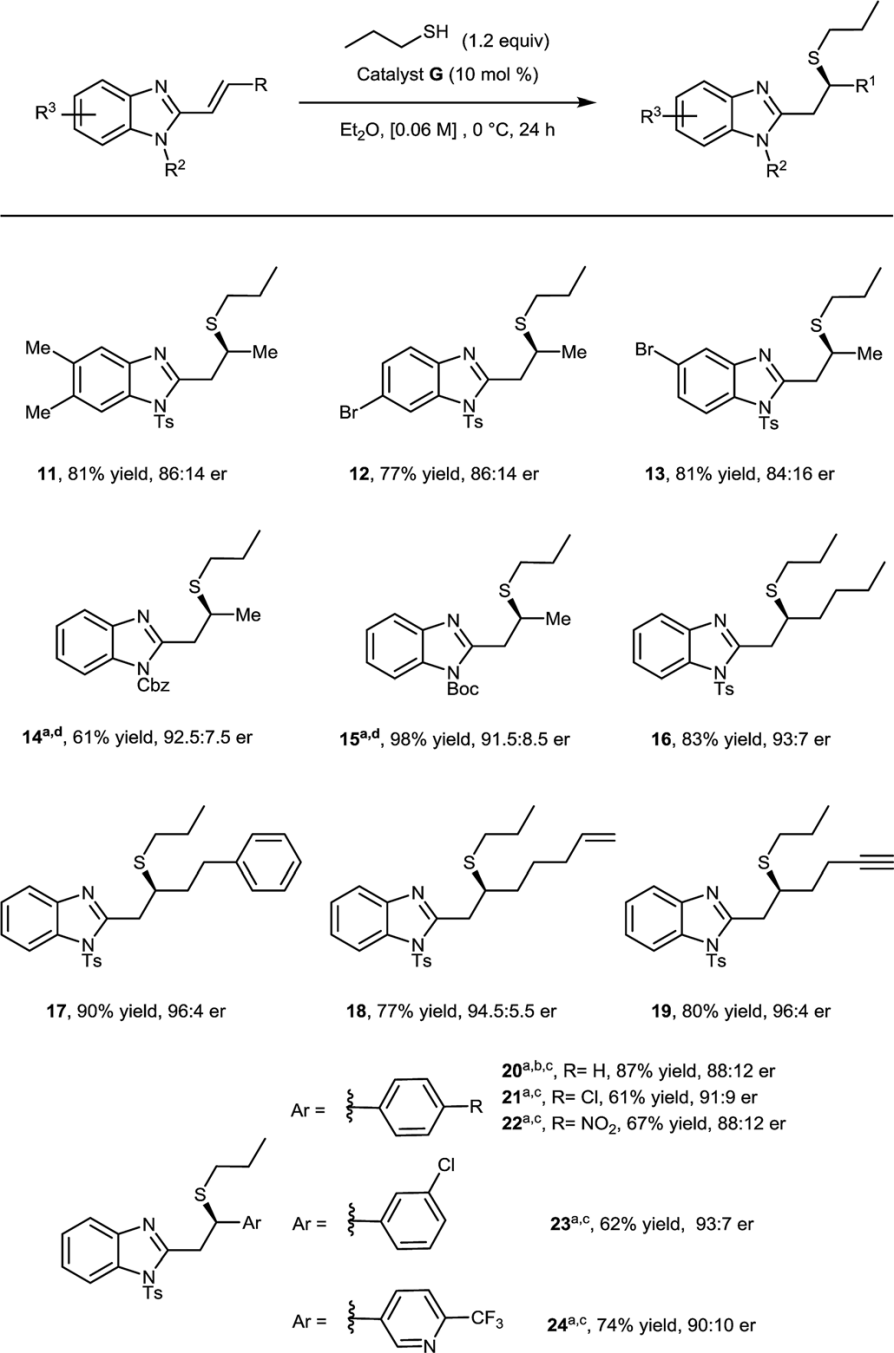
Scope of the alkenyl benzimidazole coupling partner. ^a^Reaction carried out at 22 °C. ^b^Reaction carried out using catalyst **F**. ^c^Reaction carried out in THF. ^d^Reaction carried out using 3 eq. of thiol.

We were pleased to find that the high enantioselectivity of the reaction was largely maintained when the nitrogen protecting group was changed from *N*-tosyl to *N*-Cbz (**14**) or *N*-Boc (**15**), however in these cases reactivity was found to diminish. This was easily circumvented by running the reaction at 22 °C using 3 equivalents of 1-propanethiol.^18^

Having varied the substitution pattern on the benzimidazole, we proceeded to investigate the scope with respect to substituents on the alkenyl moiety. The introduction of higher order linear alkyl chains, bearing aromatic, alkene and alkyne substituents, was well-tolerated with all *n*-propyl thiol Michael additions providing the corresponding products (**16–19**) in excellent yield and enantioselectivity. When substituting the alkene moiety with an aromatic group, the solvent was switched to THF and reactions were run at 22 °C due to decreased solubility and reactivity of the substrates. When a phenyl substituent was introduced on the alkenyl moiety, catalyst **G** only provided a moderate Michael adduct **20** in 77 : 23 er, however this was boosted to 88 : 12 when using diastereomeric catalyst **F**. Introducing electron withdrawing groups at either the *para* or *meta* positions of the phenyl ring afforded the corresponding products **21–23** in good yield and enantioselectivity; in these cases, however, catalyst **G** proved superior to **F**. Finally, when the phenyl ring was exchanged with a 3-pyridyl moiety, it smoothly afforded the corresponding adduct **24** in 74% yield and 90 : 10 er.

Increasing the reaction scale 10-fold (1 mmol) afforded **2** in equal yield and er, which upon treatment with HCl (5 M aq.) gave corresponding deprotected product **25** in quantitative yield. Single crystal X-ray analysis of **25** allowed the absolute configuration of sulfa-Michael product **2** to be determined as *S* when using catalyst **G**. We were also pleased to find that, upon treatment of **2** with *m*-CPBA, sulfone **26** was obtained in 95% yield with no loss of optical purity (Scheme 3).

**Scheme 3:**
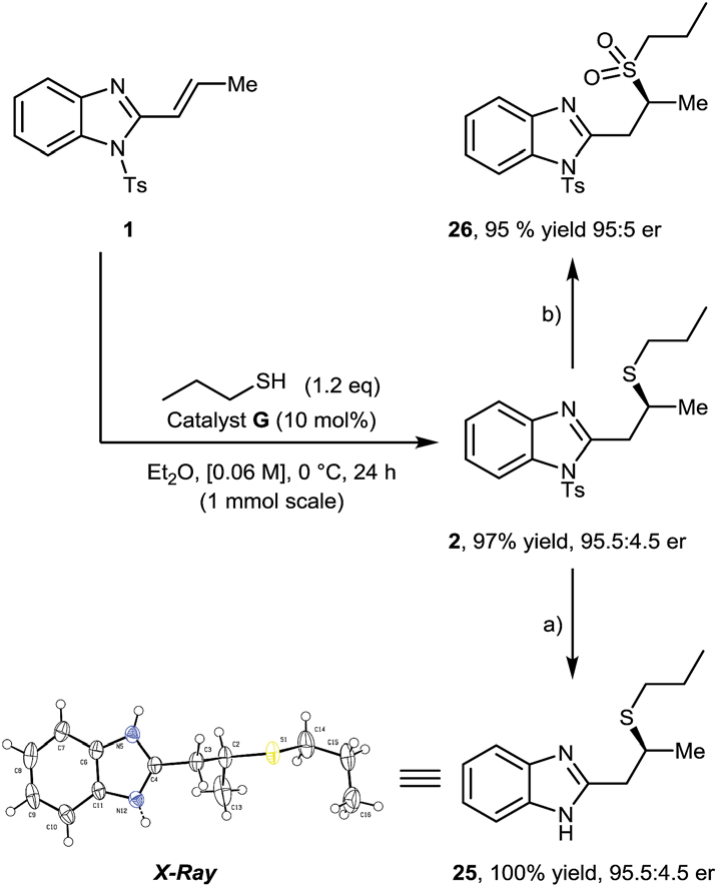
Scale up (to 1 mmol) and derivatization of compound **2** and determination of absolute configuration of **25** by single crystal X-ray analysis. (a) 5 M aq. HCl, 40 °C, THF, 10 h. (b) *m*-CPBA, CH_2_Cl_2_, 22 °C, 4 h.

We used density functional theory (DFT) to investigate the origins of enantioselectivity, performing calculations at the wB97XD/6-31G(d) level of theory (Fig. 3).^19^ Calculations considered PPh_3_-derived catalyst G* with the PMP-groups of **G** modelled by Ph-groups. The most stable conformation of (most enantioselective) catalyst G* has substituents either side of the urea oriented with a hydrogen atom towards sulfur: other rotamers are disfavoured. This creates a pocket with the iminophosphorane positioned above the thiourea (from the perspective of Fig. 3). Two substrate activation modes are possible (**A***vs.***B**) and either could in principle lead to the formation of the major observed enantiomer. Computationally, we find that the interaction of the thiolate nucleophile with the protonated iminophoshorane and the benzimidazole with the thiourea (mode A) is energetically favored by 4–5 kcal mol^–1^ over the alternative (mode B) in which the thiourea binds the nucleophile and the benzimidazole to the protonated iminophoshorane. This mode of activation is consistent with the observed sense of enantioselectivity, and with earlier mechanistic proposals of Takemoto. Recent theoretical studies of Grayson and Houk have emphasized the importance of activation mode B in sulfa-Michael reactions promoted by Cinchona-derived catalysts.^20^ Our present results suggest that both activation modes may be operative, depending on catalyst and substrate, as originally hypothesized by Soós and I. Pápai.^21^

**Fig. 3:**
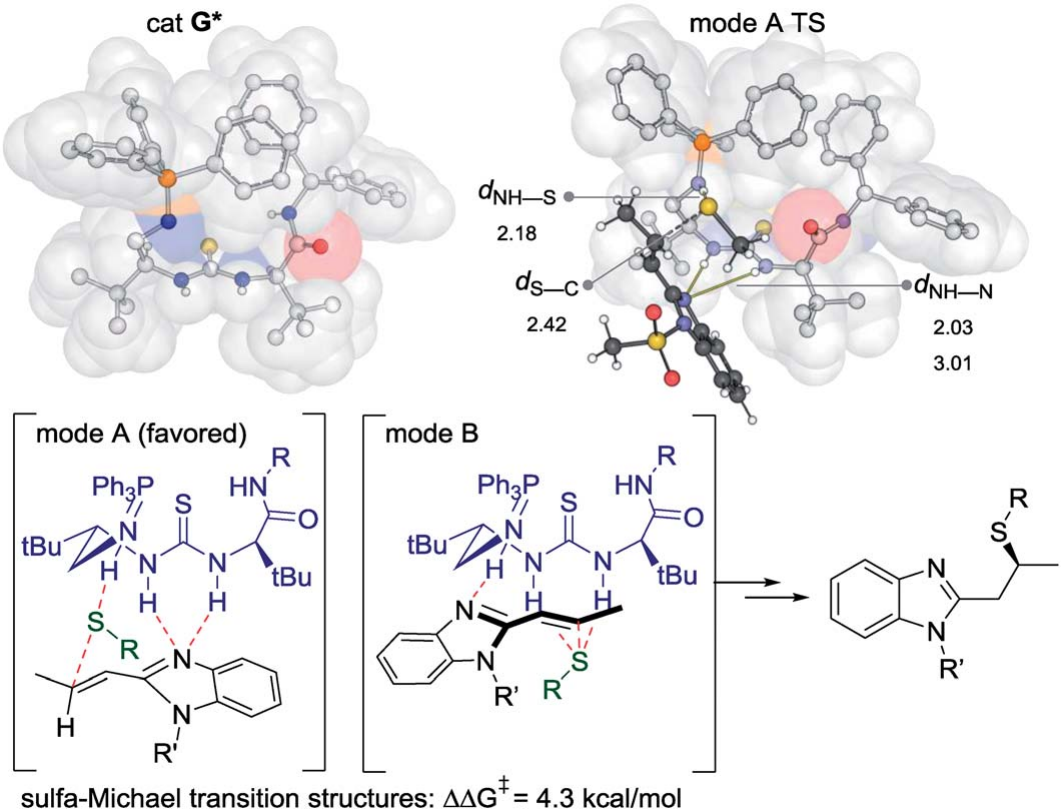
SMD-wB97XD/6-31G(d) computed structure of catalyst G* and the most favourable transition structure leading to the major enantiomer.

## Conclusions

In summary, the first enantioselective sulfa-Michael addition of alkyl thiols to alkenyl benzimidazoles has been described. Excellent yields and good enantioselectivities were achieved across a broad range of alkyl thiol and alkenyl benzimidazole reaction partners using a second generation BIMP organocatalyst. This work further demonstrates the versatility and high activity of the BIMP catalyst family, as well as expanding its use in methodology for the synthesis of biologically relevant chiral benzimidazole derivatives. Further investigations into new catalyst designs and applications for BIMP promoted reactivity are underway in our laboratories.

## Conflicts of interest

There are no conflicts to declare.

## Supplementary Material

Supplementary information

Crystal structure data
