# Supplementary material for: Bifunctional iminophosphorane catalysed enantioselective sulfa-Michael addition of alkyl thiols to alkenyl benzimidazoles
Source: Chem Sci. 2018 Jul 23;9(34):6969–74. doi: 10.1039/c8sc01804a (PMC6124915; doi:10.1039/c8sc01804a)
Supplement: Supplementary file 1 [file SC-009-C8SC01804A-s001.pdf]

Electronic Supplementary Material (ESI) for Chemical Science.
This journal is © The Royal Society of Chemistry 2018

Supporting Information

Bifunctional Iminophosphorane Catalyzed Enantioselective Sulfa-Michael Addition of Alkyl Thiols to Alkenyl Benzimidazoles

Michele Formica†[a], Geoffroy Sorin†[a,b], Alistair J. M. Farley[a], Jesús Díaz[c] Robert S. Paton[d]*and Darren J Dixon[a]*

Abstract: The first enantioselective sulfa-Michael addition of alkyl thiols to alkenyl benzimidazoles, enabled by a bifunctional iminophosphorane (BIMP) organocatalyst, is described. The iminophosphorane moiety of the catalyst provides the required basicity to deprotonate the thiol nucleophile while the chiral scaffold and H-bond donor control facial selectivity. The reaction is broad in scope with respect to the thiol and benzimidazole reaction partners with the reaction proceeding in up to 98% yield and 96:4 er.

[a] **M. Formica, Dr. G. Sorin, Dr. A. J. M. Farley, Prof. Dr. D. J. Dixon**
Department of Chemistry, Chemistry Research Laboratory
University of Oxford
Mansfield Road, Oxford OX1 3TA (UK)
E-mail: darren.dixon@chem.ox.ac.uk
[b] **Dr. G. Sorin**
Faculté des Sciences Pharmaceutiques et Biologiques
Unité CNRS UMR 8638 COMETE
Paris Descartes University, Sorbonne Paris Cité
4 avenue de l'observatoire, 75270 Paris cedex 06 (France)
† Authors contributed equally to this work
[c] **Dr. J. Díaz**
Departamento de Química Orgánica,
Universidad de Extremadura, Avda.
Universidad, s/n, 10003 Cáceres (Spain)
[d] **Prof. Dr. R. S. Paton**
Department of Chemistry
Colorado State University
Fort Collins, Colorado 80523 (USA)

Contents

1/ General Information 3
2/ Reaction Optimization 4
3/ Control Experiments 6
4/ Synthesis of precatalysts and catalysts 7
5/ Preparations of *N*-Ts protected alkenyl benzimidazole 7
6/ General Procedures: 14
7/ Cleavage of *N*-Ts group (25) 27
8/ Oxidation of sulfur (26) 28
9/ Removal of *para*-Methoxybenzyl group (27) 29
10/ Introduction of a 4-Me group (29) 30
11/ ^{1}H and ^{13}C NMR spectra 32
12/ HPLC traces 72
13/ Single Crystal X-Ray Diffraction Data 97
14/ Computational Methods 101

1/ General Information

Reactions were carried out under a nitrogen atmosphere in oven-dried glassware at room temperature (22 °C) unless stated otherwise. Standard inert atmosphere techniques were used in handling all air and moisture sensitive reagents. Thin-layer chromatography (TLC) was performed using Merck aluminium backed sheets coated with Merck Kieselgel 60 F254 (230-400 mesh) fluorescent treated silica, which were visualised under UV light (λmax= 254 or 365 nm). Flash column chromatography was performed using Merck Kieselgel (230-400 mesh). All ^{1}H,^{13}C and ^{19}F NMR spectra were recorded using a Bruker 500 MHz and Bruker 400 MHz spectrometers and are quoted in ppm for measurement against a tetramethylsilane (TMS) or residual solvent peak internal standard. Coupling constants (J) are reported in hertz (Hz). Two-dimensional spectroscopy (COSY, HSQC and HMBC) was used to assist in the assignment and the data is not reported. IR spectra were recorded on a Bruker Tensor 27 FT-IR spectrometer deposited as a thin film. Melting points were recorded using a Leica Galen III hot-stage microscope apparatus and are reported uncorrected in degrees Celsius (°C). Low resolution mass spectra were recorded on a Waters LCT premier XE Micromass spectrometer (ESI). High resolution mass spectra (ESI) were recorded on a Bruker MicroTof mass spectrometer. Optical rotations were recorded using a Perkin Elmer 341 polarimeter; $[\alpha]_D$ T values are reported in 10^{-1} deg·cm^{2} g^{-1} ; concentrations (c) are quoted in g/100 mL; D refers to the D-line of sodium (589 nm); temperatures (T) are given in degrees Celsius (°C). (+) and (–) compound number prefixes indicate the sign of the optical rotation. The enantiomeric excesses were determined by HPLC analysis on an Agilent 1200 Series instrument employing a chiral stationary phase column specified in the individual experiment and by comparing the samples with the appropriate racemic mixtures. Concentration under reduced pressure was performed by rotary evaporation at the appropriate pressure and temperature. Reagents used were obtained from commercial suppliers or purified according to standard procedures. Petroleum ether refers to distilled light petroleum of fraction 30 - 40 °C. Anhydrous toluene, tetrahydrofuran, dichloromethane and diethyl ether were dried by filtration through activated alumina (powder ~150 mesh, pore size 58 Å, basic, Sigma-Aldrich) columns. Dimethyl sulfoxide and dimethylformamide were used as supplied. Deuterated solvents were used as supplied.

2/ Reaction Optimization

Table S1: Catalyst Screen

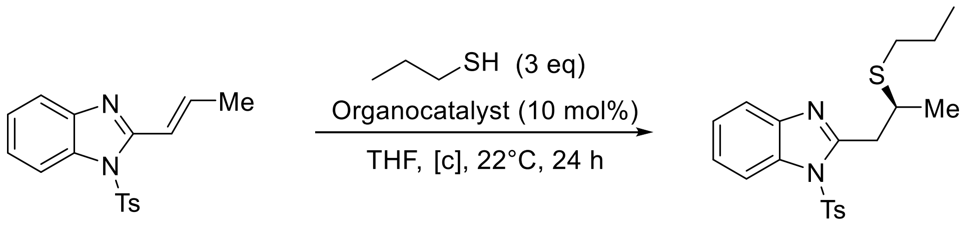

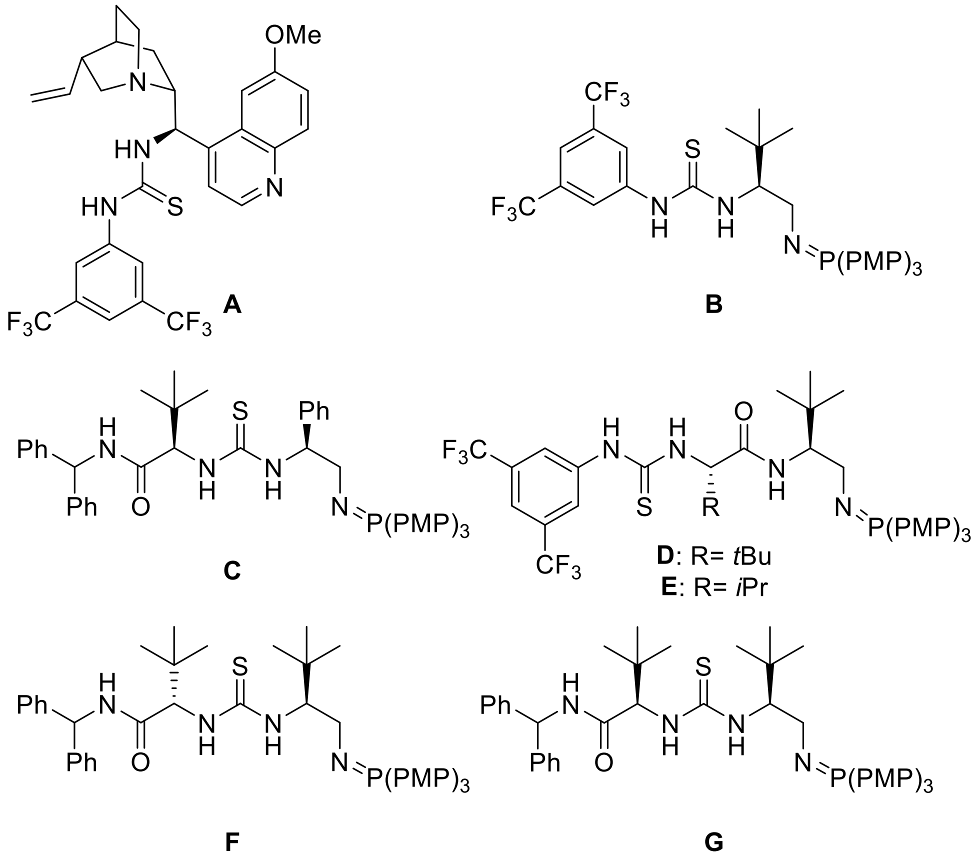

Entry	Catalyst	[c] Mol/L^{-1}	Yield %	e.r
1	A	0.50	12	53:47
2	B	0.50	80	83/17
3	C	0.25	92	86/14
4	D	0.50	83	66/34
5	E	0.50	95	83/17
6	F	0.50	90	90/10
7	G	0.06	90	93/7

Table S2: Solvent and Condition optimisation

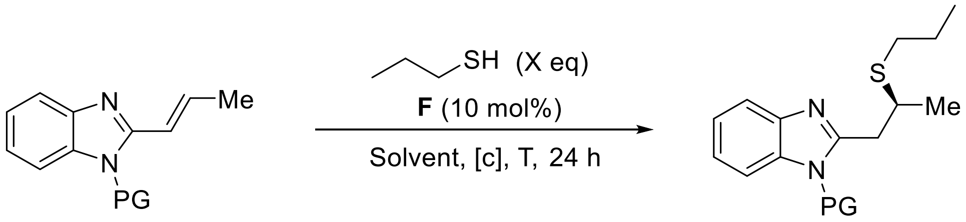

Entry	Protecting Group	Solvent	Thiol equiv.	[c] Mol/L^{-1}	Temp. (°C)	Yield %	e.r
1	Ts	THF	3.0	0.50	22	90	90/10
2	Ts	THF	3.0	0.25	22	86	91/9
3	Ts	THF	3.0	0.125	22	86	90/10
4	Ts	THF	3.0	0.50	12	83	90/10
5	Ts	THF	3.0	0.50	-15	93	92/8
6	Ts	THF	3.0	0.50	-40	88	82/18
7	Ts	TBME	3.0	0.50	22	80	91/9
8	Ts	2-MeTHF	3.0	0.50	22	80	91/9
9	Ts	1,4-dioxane	3.0	0.50	22	79	89/11
10	Ts	CH_2Cl_2	3.0	0.50	22	70	91/9
11	Ts	MeCN	3.0	0.50	22	81	73/27
12	Ts	Toluene	3.0	0.50	22	92	89/11
13	Ts	2-MeTHF	3.0	0.06	22	55	91/9
14	Ts	Et_2O	3.0	0.06	22	92	93/7
15	Ts	Et_2O	2.0	0.25	22	92	91/9
16	Ts	Et_2O	1.2	0.25	22	94	92/8
17	Ts	2-MeTHF	3.0	0.50	0	85	91/9
18	Ts	TBME	3.0	0.50	0	92	92/8
19	Ts	THF	0.5 then 0.7	0.50	22	86	90/10
**20**	**Ts**	**Et_2O**	**1.2**	**0.06**	**0**	**93**	**94/6**
21	Boc	THF	3.0	0.50	22	84	91/9
22	Boc	TBME	3.0	0.50	22	90	90/10
**23[a]**	**Ts**	**Et_2O**	**1.2**	**0.06**	**22**	**98**	**95/5**

[a]Performed using catalyst **G**. All reactions performed on 0.1 mmol scale.

3/ Control Experiments

Table S3: Control Experiments

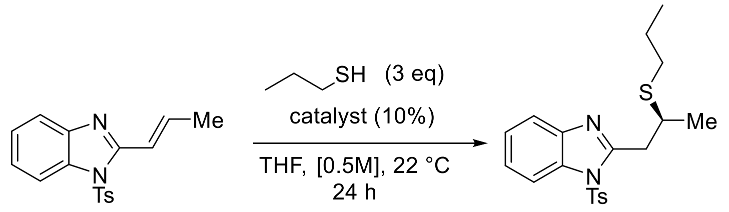

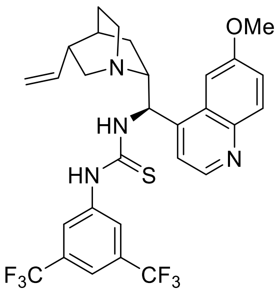

A

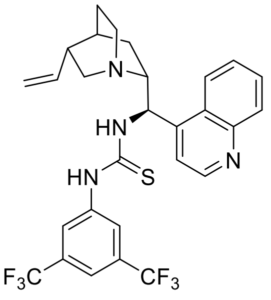

H

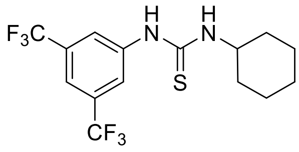

I

Entry	Catalyst	Yield	er
1	None	24 %	N/A
2	**A**	12 %	53/47
3	**H**	6 %	52/48
4	None (Basified Thiol)[a]	12%	N/A
5	Et_3N (Basified Thiol)[a]	trace	N/A
6	Benzoic Acid (Basified Thiol)[a]	89%	N/A
7	**A** (Basified Thiol)[a]	12 %	52/48
8	**I** (Basified Thiol)[a]	33 %	N/A
9[b]	**G** (Basified Thiol)[a]	87%	94.5/5.5

[a]Thiol was basified by allowing it to stand over K_2CO_3 for 24 h. [b] Reaction performed in Et_2O at 0°C using 1.2 eq of 1-propane thiol.

Experimental Procedures

4/ Synthesis of precatalysts and catalysts

Catalysts **A**,[1] **B**,[2] **C** and **F-G**,[3] **D-E**,[4] **H**,[5] **I**[6] were prepared according to literature procedures.

5/ Preparations of *N*-Ts protected alkenyl benzimidazole

Non commercially available aldehydes were prepared by simple oxidation of the corresponding alcohol with Dess-Martin Periodinane following the procedure reported by Snowden *et al.*[7]
tert-butyl-2-((diethoxyphosphoryl)methyl)-1*H*-benzo[*d*]imidazole-1-carboxylate (**Bz0**), *N*-Ts benzimidazoles **1**, **Bz1**, **Bz2**, *N*-Boc- and *N*-Cbz benzimidazoles **Bz3** and **Bz4** were prepared as described by Terada *et al.* [8]

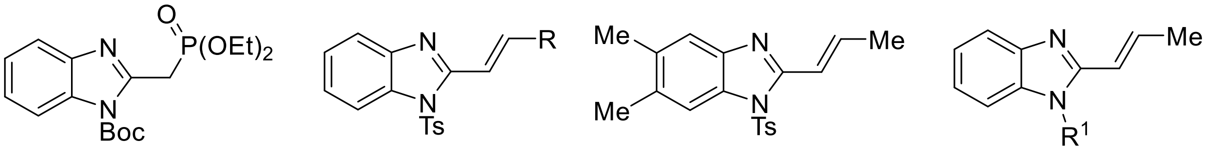

Bz0 R = Me **1**, Ph **Bz1** **Bz2** R[1] = Cbz **Bz3**, Boc **Bz4**

Synthesis of (*E*)-6-bromo-2-(prop-1-en-1-yl)-1-tosyl-1*H*-benzo[*d*]imidazole and (*E*)-5-bromo-2-(prop-1-en-1-yl)-1-tosyl-1*H*-benzo[*d*]imidazole

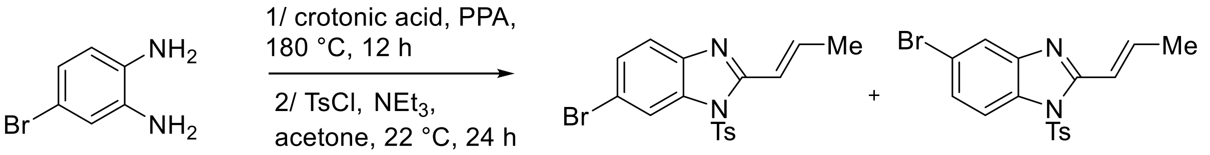

Bz5 **Bz6**

To a mixture of crotonic acid (2.68 g, 30 mmol) in polyphosphoric acid (12 g) was added 4-bromo-1,2-diaminobenzene (5.61 g, 30 mmol). The reaction was heated at 180 °C for 12 h. The mixture was cooled down to 22 °C and poured carefully onto a saturated solution of $NaHCO_3$. Solid $NaHCO_3$ was added until neutral pH was obtained. The solution was diluted with EtOAc and water then stirred vigorously for 10 min. The aqueous phase was extracted twice with EtOAc and the combined organic phase were washed with brine, dried over $MgSO_4$ and concentrated under vacuum affording 3.9 g of purple solid.
The crude residue (2.0 g, 8.4 mmol) was dissolved in acetone (40 mL), NEt_3 (1.41 mL, 10.1 mmol) was added then TsCl (1.77 g, 9.3 mmol). The mixture was stirred for 24h at 22 °C. The solvent was removed

---

[1] B. Vakulya, S. Varga, A. Csámpai and T. Soós, *Org. Lett.*, 2005, **7**, 1967.
[2] M. G. Núñez, A. J. M. Farley and D. J. Dixon, *J. Am. Chem. Soc.*, 2013, **135**, 16348.
[3] J. Yang, A. J. M. Farley and D. J. Dixon, *Chem. Sci.*, 2017, **8**, 606.
[4] G. P. Robertson, A. J. M. Farley and D. J. Dixon, *Synlett*, 2016, **27**, 21.
[5] J. Ye, D. J. Dixon and P. S. Hynesa *Chem. Commun.*, 2005, **0**, 4481.
[6] Y. Okino, Y. Hoashi, T. Furukawa and X. Xu, Y. Takemoto, *J. Am. Chem. Soc.*, 2005, **127**, 119.
[7] M. K. Gupta, Z. Li and T. S. Snowden *J. Org. Chem.*, 2012, **77**, 4854.
[8] Y-Y. Wang, K. Kanomata, T. Korenaga and M. Terada *Angew.Chem. Int. Ed.*, 2016, **55**, 927.

under vacuum and the crude was purified by flash chromatography on silica gel (pentane 9/ EtOAc 1). A subsequent trituration of each compound in a mixture of pentane/ Et_2O (1/1) afford pure (*E*)-6-bromo-2-(prop-1-en-1-yl)-1-tosyl-1*H*-benzo[*d*]imidazole (**Bz5**) as a white solid (m = 0.38 g, 12 %) and (*E*)-5-bromo-2-(prop-1-en-1-yl)-1-tosyl-1*H*-benzo[*d*]imidazole (**Bz6**) as an off-white solid (m= 0.45 g, 14%).

(*E*)-6-Bromo-2-(prop-1-en-1-yl)-1-tosyl-1*H*-benzo[*d*]imidazole (**Bz5**)

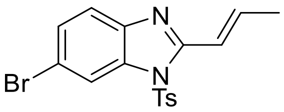

Mp : 160°C (from EtOAc/Petrol); **^{1}H NMR** (400 MHz, $CDCl_3$) δ 8.24 (d, *J* = 1.8 Hz, 1H), 7.78 (d, *J* = 8.5 Hz, 2H), 7.50 (d, *J* = 8.5 Hz, 1H), 7.44 (dd, *J* = 8.5, 1.8 Hz, 1H), 7.30 (d, *J* = 7.8 Hz, 2H), 7.20 – 7.14 (m, 1H), 7.10 (dq, *J* = 15.3, 6.2 Hz, 1H), 2.40 (s, 3H), 2.05 (d, *J* = 5.2 Hz, 3H); **^{13}C NMR** (101 MHz, $CDCl_3$) δ 151.6, 146.3, 141.6 140.4, 135.2, 133.9, 130.4, 128.5, 127.0, 120.9, 118.2, 118.1, 117.0, 77.5, 77.2, 76.8, 21.8, 19.3; **IR** (film) υ_{max}/cm^{-1}: 1642, 1597, 1423, 1378, 1166, 1039, 812, 710, 666; **HRMS** (ESI+): calcd. for $C_{17}H_{16}O_2N_2BrS$ $[M+H]^+$ 391.0109 and 393. 0089, found 391.0113 and 393.0091.

(*E*)-5-Bromo-2-(prop-1-en-1-yl)-1-tosyl-1*H*-benzo[*d*]imidazole (**Bz6**)

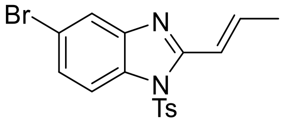

Mp : 128°C (from EtOAc/Petrol); **^{1}H NMR** (400 MHz, $CDCl_3$) δ 7.90 (d, *J* = 8.8 Hz, 1H), 7.77 – 7.70 (m, 3H), 7.41 (dd, *J* = 8.8, 1.9 Hz, 1H), 7.29 – 7.22 (m, 2H), 7.21 – 7.12 (m, 1H), 7.09 (dq, *J* = 15.3, 5.3 Hz, 2H), 2.37 (s, 3H), 2.04 (d, *J* = 5.3 Hz, 3H); **^{13}C NMR** (101 MHz, $CDCl_3$) δ 152.2, 146.7, 143.9, 140.8, 135.7, 132.0, 130.3, 127.8, 126.9, 122.7, 118.2, 118.2, 115.7, 77.5, 77.7, 76.8, 21.8, 19.3; **IR** (film) υ_{max}/cm^{-1}: 1644, 1596, 1446, 1379, 1166, 1048, 809, 732, 665; **HRMS** (ESI+): calcd. for $C_{17}H_{16}O_2N_2BrS$ $[M+H]^+$ 391.0109 and 393.0089, found 391.0110 and 393.0089.

Other *N*-Ts benzimidazoles Michael acceptors were prepared according the sequence reported below:

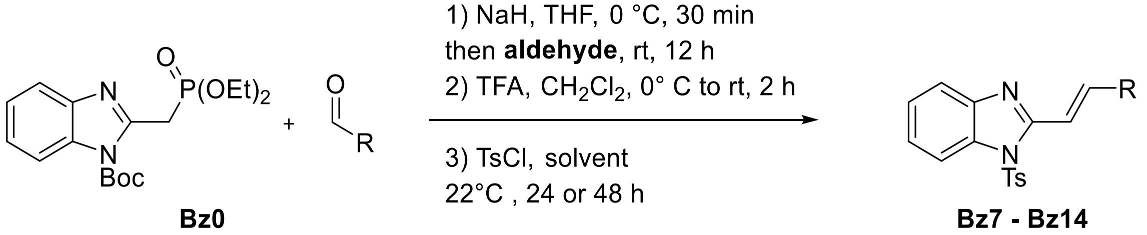

General procedure I for the synthesis of *N*-Ts precursors (GP I):

To a solution of *N*-Boc protected benzimidazole **Bz0** (368 mg, 1.0 mmol) in dry THF (12 mL) at 0°C was added portionwise NaH 60% (w/w in oil) (64 mg, 1.6 mmol) and stirred under N_2 at 0 °C for 30 min. The corresponding aldehyde (2.0 mmol) was added at 0 °C then the reaction was allowed to warm to 22 °C and stirred for 12 h. The mixture was quenched by adding water and the aqueous phase was extracted with EtOAc. The combined organic phase were washed with brine, dried over $MgSO_4$ and concentrated under reduced pressure.
The residue was taken in dry CH_2Cl_2 (1 mL/ 1mmol) and cooled to 0°C. TFA (1mL/ 1 mmol) was added and the reaction was stirred at 22 °C until TLC shows complete disappearance of the starting material. The mixture was quenched by adding a saturated solution of $NaHCO_3$. The aqueous layer was extracted with EtOAc. The combined organic phase were washed with brine, dried over $MgSO_4$ and concentrated under reduced pressure.
The crude was dissolved in CH_2Cl_2, acetone or DMF (5 mL/1 mmol), then NEt_3 (2.0 eq) and TsCl (1.2 eq) was added. The mixture was stirred over 48h at 22 °C then volatiles were removed under vacuum. The resulting residue was submitted to a flash chromatography on silica gel or triturated to give the pure product.

(*E*)-2-(Hex-1-en1-yl)-1-tosyl-1*H*-benzo[*d*]imidazole (**Bz7**)

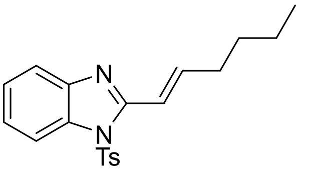

(*E*)-2-(Hex-1-en1-yl)-1-tosyl-1*H*-benzo[*d*]imidazole was synthesized following **GP I** using hexanal (200.3 mg, 2.0 mmol, 246 µL) as the aldehyde. Acetone was used as a solvent for the *N*-tosylation. Purification by flash chromatography on silica gel (8 pentane/2 Et_2O) afforded pure **Bz7** as a colourless oil. (156 mg, 0.440 mmol, 50% overall yield).

^{1}H NMR (400 MHz, $CDCl_3$) δ = 8.10 – 8.01 (m, 1H), 7.76 (d, *J* = 8.0 Hz, 2H), 7.67 – 7.59 (m, 1H), 7.36 – 7.28 (m, 2H), 7.24 (d, *J* = 8.0 Hz, 3H), 7.18 (dt, *J* = 15.5, 1.3 Hz, 1H), 7.07 (dt, *J* = 15.5, 6.9 Hz, 1H), 2.40 – 2.32 (m, 5H), 1.58 – 1.48 (m, 2H), 1.47 – 1.34 (m, 2H), 0.95 (t, *J* = 7.3 Hz, 3H); **^{13}C NMR** (101 MHz, $CDCl_3$) δ = 151.3, 145.9, 144.8, 142.6, 135.6, 133.1, 130.2, 127.0, 125.1, 124.9, 119.9, 117.3, 114.0, 33.2, 30.8, 22.4, 21.8, 14.1; **IR** (film) υ_{max}/cm^{-1}: 2957, 2928, 1640, 1448, 1377, 1169, 1120, 1089, 743, 670; **HRMS** (ESI+): calcd. for $C_{20}H_{23}O_2N_2S$ $[M+H]^+$ 355.1474, found 355.1473.

(*E*)-2-(4-Phenylbut-1-en-1-yl)-1-tosyl-1*H*-benzo[*d*]imidazole (**Bz8**)

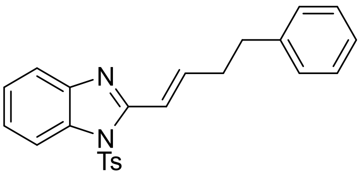

(*E*)-2-(4-Phenylbut-1-en-1-yl)-1-tosyl-1*H*-benzo[*d*]imidazole was synthesized following **GP I** using 3-phenylpropanal (268.3 mg, 2.0 mmol, 263 µL) as the aldehyde. DMF was used as a solvent for the tosylation. Purification by flash chromatography on silica gel (9 pentane/1 EtOAc) afforded pure **Bz8** as a yellow solid. (130 mg, 0.322 mmol, 30% overall yield).

Mp : 90°C (from EtOAc/Petrol); ^{1}**H NMR** (400 MHz, $CDCl_3$) δ = 8.10 – 7.98 (m, 1H), 7.68 (d, *J* = 8.5 Hz, 2H), 7.65 – 7.62 (m, 1H), 7.36 – 7.30 (m, 3H), 7.28 – 7.24 (m, 3H), 7.23 – 7.19 (m, 3H), 7.14 (dt, *J* = 15.5, 6.5 Hz, 1H), 2.89 (dd, *J* = 8.8, 6.5 Hz, 2H), 2.75 – 2.68 (m, 2H), 2.36 (s, 3H); ^{13}**C NMR** (101 MHz, $CDCl_3$) δ 151.0, 145.9, 143.3, 142.6, 141.1, 135.4, 133.0, 130.2, 128.6, 128.6, 126.9, 126.2, 125.16, 125.0, 119.9, 117.9, 114.0, 77.5, 77.2, 76.8, 34.9, 34.9, 21.8; **IR** (film) υ_{max}/cm^{-1}: 3026, 2923, 1641, 1597, 1449, 1377, 1173, 1049, 743, 670; **HRMS** (ESI+): calcd. for $C_{24}H_{23}O_2N_2S$ $[M+H]^+$ 403.1474, found 403.1476.

(*E*)-2-(Hepta-1,6-dien-1-yl)-1-tosyl-1*H*-benzo[*d*]imidazole (**Bz9**)

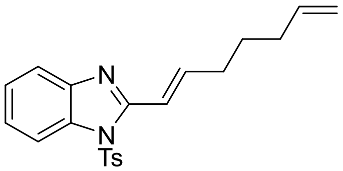

(*E*)-2-(Hepta-1,6-dien-1-yl)-1-tosyl-1*H*-benzo[*d*]imidazole was synthesized following **GP I** hex-5-enal (196.3 mg, 2.0 mmol) as the aldehyde. CH_2Cl_2 was used as a solvent for the *N*-tosylation. Purification by flash chromatography on silica gel (9 pentane/1 EtOAc) afforded pure **Bz9** as a colourless oil. (310 mg, 0.846 mmol, 44% overall yield).

^{1}**H NMR** (400 MHz, $CDCl_3$) δ 8.08 – 8.03 (m, 1H), 7.76 (d, *J* = 8.4 Hz, 2H), 7.70 – 7.58 (m, 1H), 7.39 – 7.29 (m, 2H), 7.25 (d, *J* = 0.8 Hz, 2H), 7.20 (dt, *J* = 15.4, 1.4 Hz, 1H), 7.07 (dt, *J* = 15.5, 6.9 Hz, 1H), 5.84 (ddt, *J* = 16.9, 10.2, 6.7 Hz, 1H), 5.12 – 4.97 (m, 2H), 2.45 – 2.38 (m, 1H), 2.36 (s, 3H), 2.22 – 2.05 (m, 2H), 1.65 (p, *J* = 7.4 Hz, 3H); ^{13}**C NMR** (101 MHz, $CDCl_3$) δ 151.2, 146.0, 144.3, 142.6, 138.3, 135.5, 133.1, 130.2, 126.9, 125.2, 124.9, 119.9, 117.6, 115.2, 114.0, 33.4, 32.8, 27.9, 21.8; **IR** (film) υ_{max}/cm^{-1}: 3076, 2927, 1640, 1448, 1378, 1171, 1049, 919, 744, 670; **HRMS** (ESI+): calcd. for $C_{21}H_{23}O_2N_2S$ $[M+H]^+$ 367.1477, found 367.1474.

<u>(*E*)-2-(Hex-1-en-5-yn-1-yl)-1-tosyl-1*H*-benzo[*d*]imidazole (**Bz10**)</u>

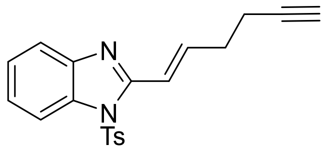

(*E*)-2-(Hex-1-en-5-yn-1-yl)-1-tosyl-1*H*-benzo[*d*]imidazole was synthesized following **GP I** using pent-4-ynal (164.2 mg, 2.0 mmol) as the aldehyde. CH_2Cl_2 was used as a solvent for the tosylation. Purification by flash chromatography on silica gel (8 pentane/2 EtOAc) afforded pure **Bz10** as a white solid. (170 mg, 0.485 mmol, 26% overall yield).

Mp : 104°C (from EtOAc/Petrol); **^{1}H NMR** (400 MHz, $CDCl_3$) δ = 8.07 – 8.01 (m, 1H), 7.78 (d, *J* = 8.5 Hz, 2H), 7.67 – 7.60 (m, 1H), 7.35 – 7.27 (m, 3H), 7.23 (d, *J* = 8.5 Hz, 2H), 7.09 (dt, *J* = 15.5, 6.7 Hz, 1H), 2.59 (brq, *J* = 6.7 Hz, 2H), 2.44 (td, *J* = 7.1, 2.6 Hz, 2H), 2.34 (s, 3H), 2.03 (t, *J* =2.6 Hz, 1H); **^{13}C NMR** (101 MHz, $CDCl_3$) δ 150.7, 146.0, 142.5, 141.5, 135.4, 133.0, 130.2, 127.0, 126.9, 125.2, 125.1, 112.0, 118.6, 114.0, 83.2, 77.5, 77.2, 76.8, 69.5, 32.2, 21.7, 18.1; **IR** (film) υ_{max}/cm^{-1}: 3295, 2920, 1644, 1596, 1448, 1377, 1175, 1049, 744, 669; **HRMS** (ESI+): calcd. for $C_{20}H_{19}O_2N_2S$ [M+H]$^+$ 351.1168, found 351.1159.

<u>(*E*)-2-(4-Chlorostyryl)-1-tosyl-1*H*-benzo[*d*]imidazole (**Bz11**)</u>

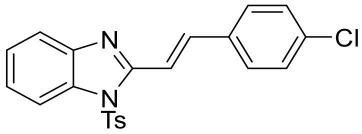

(*E*)-2-(4-Chlorostyryl)-1-tosyl-1*H*-benzo[*d*]imidazole was synthesized following **GP I** using 0.68 mmol (250 mg) of *N*-boc protected benzimidazole phosphonate and 4-chlorobenzaldehyde (127.9 mg, 0.91 mmol) as the aldehyde. CH_2Cl_2:Et_3N 1:1 was used as a solvent for the *N*-tosylation. Purification by flash chromatography on silica gel (pentane to 4 pentane/1 EtOAc) afforded pure **Bz11** as an off-white powder. (168 mg, 0.411 mmol, 61% overall yield).

Mp : 168 °C (from EtOAc/Petrol); **^{1}H NMR** (400 MHz, $CDCl_3$) δ 8.12 – 8.02 (m, 1H), 7.90 (d, *J* = 4.5 Hz, 2H), 7.82 – 7.77 (m, 2H), 7.75 – 7.69 (m, 1H), 7.64 – 7.58 (m, 2H), 7.47 – 7.41 (m, 2H), 7.40 – 7.36 (m, 2H), 7.25 (d, *J* = 8.1 Hz, 2H), 2.37 (s, 3H); **^{13}C NMR** (101 MHz, $CDCl_3$) δ 150.9, 146.1, 142.8, 138.4, 135.5, 135.4, 134.4, 133.3, 130.3, 129.4, 129.0, 126.9, 125.5, 125.3, 120.1, 115.1, 114.1, 21.8; **IR** (film) υ_{max}/cm^{-1}: 3057, 1512, 1490, 1447, 1378, 1344, 1200, 1185, 1168, 1150, 1090, 1051, 1013, 812, 764, 744, 677, 665, 645; **HRMS** (ESI+): calcd. for $C_{22}H_{18}O_2N_2ClS$ [M+H]$^+$ 409.0772, found 409.0770.

(*E*)-2-(4-Nitrostyryl)-1-tosyl-1*H*-benzo[*d*]imidazole (**Bz12**)

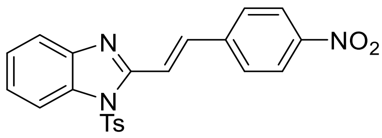

(*E*)-2-(4-Nitrostyryl)-1-tosyl-1*H*-benzo[*d*]imidazole was synthesized following **GP I** using 0.60 mmol (220 mg) of *N*-boc protected benzimidazole phosphonate using 4-nitrobenzaldehyde (120.9 mg, 0.8 mmol) as the aldehyde. CH_2Cl_2:Et_3N 1:1 was used as solvent for the *N*-tosylation. Purification by flash chromatography on silica gel (9 pentane/1 EtOAc to 7 pentane/3 EtOAc) afforded pure **Bz12** as a yellow powder. (100 mg, 0.238 mmol, 35% overall yield).

Mp : 194 °C (from EtOAc/Petrol); **^{1}H NMR** (400 MHz, $CDCl_3$) δ 8.36 – 8.25 (m, 2H), 8.15 – 8.07 (m, 2H), 7.98 (d, *J* = 15.9 Hz, 1H), 7.83 – 7.77 (m, 4H), 7.77 – 7.70 (m, 1H), 7.49 – 7.35 (m, 2H), 7.27 (d, *J* = 7.3 Hz, 2H), 2.38 (s, 3H); **^{13}C NMR** (101 MHz, $CDCl_3$) δ 150.0, 148.1, 146.4, 142.7, 142.1, 136.8, 135.3, 133.3, 130.4, 128.3, 126.9, 125.9, 125.7, 124.5, 120.4, 118.9, 114.1, 21.8; **IR** (film) υ_{max}/cm^{-1}: 3078, 1597, 1520, 1377, 1344, 1171, 748, 670; **HRMS** (ESI+): calcd. for $C_{22}H_{18}O_4N_3S$ $[M+H]^+$ 420.1009, found 420.1012.

(*E*)-2-(3-Chlorostyryl)-1-tosyl-1*H*-benzo[*d*]imidazole (**Bz13**)

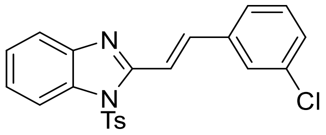

(*E*)-2-(3-Chlorostyryl)-1-tosyl-1*H*-benzo[*d*]imidazole was synthesized following **GP I** using 1.09 mmol (400 mg) of *N*-boc protected benzimidazole phosphonate and 3-chlorobenzaldehyde (205.0 mg, 1.46 mmol). CH_2Cl_2:Et_3N 1:1 was used as a solvent for the *N*-tosylation. Purification by flash chromatography on silica gel (8 pentane/2 EtOAc) afforded pure **Bz13** as a colourless powder. (186 mg, 0.455 mmol, 42% overall yield).

Mp : 126 °C (from EtOAc/Petrol); **^{1}H NMR** (400 MHz, $CDCl_3$) δ 8.11 – 8.04 (m, 1H), 7.92 (d, *J* = 15.9 Hz, 1H), 7.84 (d, *J* = 15.9 Hz, 1H), 7.80 – 7.75 (m, 2H), 7.72 – 7.67 (m, 1H), 7.62 (q, *J* = 1.4 Hz, 1H), 7.55 – 7.49 (m, 1H), 7.41 – 7.32 (m, 4H), 7.25 – 7.19 (m, 2H), 2.35 (s, 3H); **^{13}C NMR** (101 MHz, $CDCl_3$) δ 150.7, 146.2, 142.8, 138.3, 137.8, 135.4, 135.1, 133.3, 130.3 (d, *J* = 3.8 Hz), 129.6, 127.5, 126.9, 126.1, 125.5 (d, *J* = 4.9 Hz), 120.2, 116.0, 114.1, 21.8; **IR** (film) υ_{max}/cm^{-1}: 3060, 1594, 1511, 1497, 1378, 1343, 1258, 1232, 1201, 1185, 1168, 1150, 1122, 1089, 1052, 782, 764, 744, 702, 690, 671, 645; **HRMS** (ESI+): calcd. for $C_{22}H_{18}O_2N_2ClS$ $[M+H]^+$ 409.0772, found 409.0770.

(*E*)-1-Tosyl-2-(2-(6-(trifluoromethyl)pyridin-3-yl)vinyl)-1*H*-benzo[*d*]imidazole (**Bz14**)

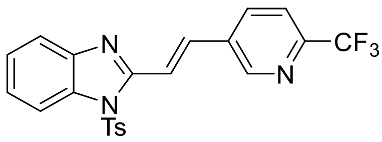

(*E*)-1-Tosyl-2-(2-(6-(trifluoromethyl)pyridin-3-yl)vinyl)-1*H*-benzo[*d*]imidazole was synthesized following **GP I** using 0.68 mmol (250 mg) of *N*-boc protected benzimidazole phosphonate and 6-(trifluoromethyl)nicotinaldehyde (159.4 mg, 0.91 mmol) as the aldehyde. CH_2Cl_2:Et_3N 1:1 was used as a solvent for the *N*-tosylation. Purification by flash chromatography on silica gel (pentane to 8 pentane/2 EtOAc) afforded pure **Bz14** as an off-white powder. (151 mg, 0.341 mmol 51% overall yield).

Mp : 158-160 °C (from EtOAc/Petrol); **^{1}H NMR** (400 MHz, $CDCl_3$) δ 8.96 (d, *J* = 2.1 Hz, 1H), 8.17 – 8.03 (m, 3H), 7.96 (d, *J* = 16.0 Hz, 1H), 7.78 (dd, *J* = 8.3, 4.6 Hz, 3H), 7.76 – 7.71 (m, 1H), 7.47 – 7.37 (m, 2H), 7.28 (d, *J* = 7.6 Hz, 2H), 2.38 (s, 3H); **^{13}C NMR** (101 MHz, $CDCl_3$) δ 149.7, 149.5, 148.3 (q, *J* = 35.2 Hz), 146.4, 142.6, 135.3, 135.0, 134.4, 134.1, 133.3, 130.5, 126.9, 126.0, 125.7, 120.8 (d, *J* = 2.8 Hz), 120.4, 119.1, 114.1, 21.8; **^{19}F NMR** (376 MHz, $CDCl_3$) δ -67.9; **IR** (film) υ_{max}/cm^{-1}: 3062, 1379, 1338, 1171, 1136, 1086, 746, 672; **HRMS** (ESI+): calcd. for $C_{22}H_{17}O_2N_3F_3S$ $[M+H]^+$ 444.0988, found 444.0984.

6/ General Procedures:

General Procedure for the Thio-1,4 Addition (GP II)

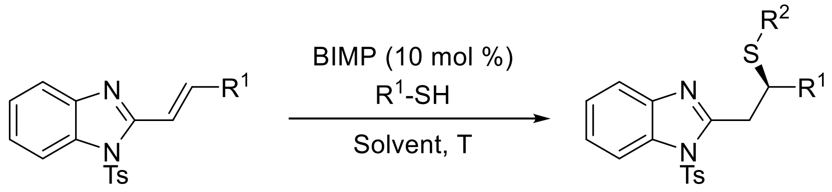

To the corresponding organoazide (0.010 mmol) and tris(4-methoxyphenyl)phosphine (0.010 mmol) under argon atmosphere was added THF (0.2 mL) and the reaction mixture was stirred for 24 h. The formation of the organocatalysts was monitored by TLC. Upon completion volatiles were removed under a stream of N_2 yielding the expected iminiphosphorane which was used without further purification.
To the corresponding Michael acceptor (0.10 mmol) and BIMP organocatalyst (0.01 mmol) under argon atmosphere was added Et_2O (1.60 mL) and thiol (0.12 mmol, 1.2 equivalents) then the reaction was stirred at 0 °C for 24 h. The reaction mixture was loaded directly onto silica gel and purified by flash column chromatography as specified in the individual experiment to afford pure sulfa-Michael addition product. The two enantiomers were separated by chiral HPLC using conditions specified in the individual experiment.

General Procedure for the Synthesis of Racemate 1,4 Addition Products (GP III)

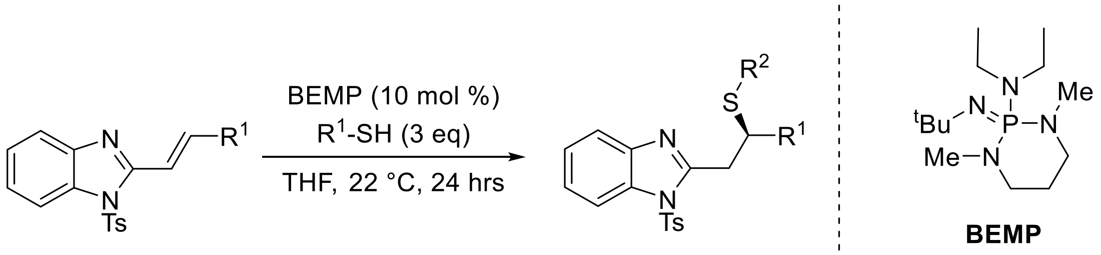

To the corresponding Michael acceptor (0.10 mmol) and 2-*tert*-Butylimino-2-diethylamino-1,3-dimethylperhydro-1,3,2-diazaphosphorine (BEMP) (3 µL, 0.010 mmol) under argon was added solvent (0.2 mL) and thiol (0.30 mmol) and the reaction was stirred at 22 °C for 24 h. Volatiles were removed under a stream of N_2 and the crude product was purified by flash column chromatography as specified in the individual experiment to afford the racemic 1,4-addition product. The two enantiomers were separated by chiral HPLC using conditions specified in the individual experiment.

(*S*)-2-(2-(Propylthio)propyl)-1-tosyl-1*H*-benzo[*d*]imidazole (**2**)

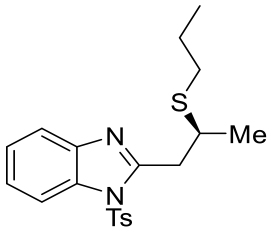

Compound **2** was synthesized according to **GPII** from **1** (31.2 mg) using catalyst **G** and 1-propanethiol (9.1 mg, 11 µL) . Clear viscous oil (7 Petrol/ 3 EtOAc) (38.0 mg, 0.098 mmol, 98% yield, 95/5 er [determined by HPLC, Chiralpak AD-H, hexane/isopropanol = 95/5, 1 mL/min, λ = 220 nm, t (major) = 16.63 min, t (minor) = 18.00 min])

$[\alpha]_D^{25}$ = -2.0 (c = 0.31, $CHCl_3$); **^{1}H NMR** (400 MHz, $CDCl_3$) δ 8.06 – 7.97 (m, 1H), 7.80 – 7.71 (m, 2H), 7.69 – 7.63 (m, 1H), 7.37 – 7.28 (m, 2H), 7.27 – 7.18 (m, 2H), 3.62 – 3.44 (m, 2H), 3.33 – 3.20 (m, 1H), 2.68 – 2.46 (m, 2H), 2.35 (s, 3H), 1.60 (h, *J* = 7.2 Hz, 2H), 1.35 – 1.27 (m, 3H), 0.95 (t, *J* = 7.3 Hz, 3H); **^{13}C NMR** (101 MHz, $CDCl_3$) δ 152.8, 146.1, 142.1, 135.5, 133.1, 130.4, 126.9, 125.1, 124.9, 120.1, 113.9, 38.5, 37.9, 33.0, 23.3, 21.8, 21.3, 13.7; **IR** (film) υ_{max}/cm^{-1}: 2871, 1596, 1481, 1377, 1173, 1189, 765, 667; **HRMS** (ESI +): calcd. for $C_{20}H_{25}O_2N_2S_2$ $[M+H]^+$ 389.1352, Found 389.1352.

(*S*)-2-(2-(Pentylthio)propyl)-1-tosyl-1*H*-benzo[*d*]imidazole (**3**)

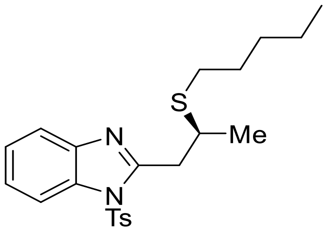

Compound **3** was synthesized according to **GPII** from **1** (31.2 mg) using catalyst **G** using pentane-1-thiol (12.5 mg, 15 µL). Clear oil (7 Petrol/ 3 EtOAc) (32.7 mg, 0.078 mmol, 78% yield, 95/5 er [determined by HPLC, Chiralcel OG, hexane/isopropanol = 97/3, 1 mL/min, λ = 220 nm, t (minor) = 8.48 min, t (major) = 10.33 min]).

$[\alpha]_D^{25}$ = -3.7 (c = 0.48, $CHCl_3$); **^{1}H NMR** (400 MHz, $CDCl_3$) δ 8.08 – 7.93 (m, 1H), 7.82 – 7.71 (m, 2H), 7.70 – 7.61 (m, 1H), 7.39 – 7.29 (m, 2H), 7.28 – 7.23 (m, 2H), 3.66 – 3.42 (m, 2H), 3.36 – 3.20 (m, 1H), 2.71 – 2.49 (m, 2H), 2.37 (s, 3H), 1.66 – 1.51 (m, 2H), 1.41 – 1.20 (m, 7H), 0.87 (t, *J* = 7.1 Hz, 3H); **^{13}C NMR** (101 MHz, $CDCl_3$) δ 152.8, 146.1, 142.1, 135.5, 133.1, 130.3, 126.9, 125.0, 124.8, 120.1, 113.9, 38.5, 37.9, 31.3, 31.0, 29.6, 22.4, 21.8, 21.3, 14.1; **IR** (film) υ_{max}/cm^{-1}: 2956, 2928, 2859, 1451, 1378, 1172, 1089, 1044, 1014, 745, 703, 667; **HRMS** (ESI +): calcd. for $C_{22}H_{29}O_2N_2S_2$ $[M+H]^+$ 417.1665, Found 417.1663.

(*S*)-2-(2-(Isopropylthio)propyl)-1-tosyl-1*H*-benzo[*d*]imidazole (**4**)

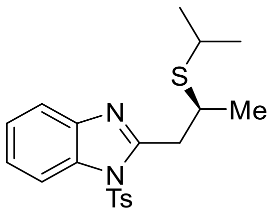

Compound **4** was synthesized according to a modified of **GPII** from **1** (31.2 mg) using catalyst **G**, propane-2-thiol (9.13 mg, 11 µL) and stirred at 22 °C. Clear viscous oil (7 Petrol/ 3 EtOAc) (29.8 mg, 0.077 mmol, 78% yield, 94/6 er [determined by HPLC, Chiralpak AD-H, hexane/isopropanol = 95/5, 1 mL/min, λ = 220 nm, t (major) = 15.60 min, t (minor) = 18.00 min]).

$[\alpha]_D^{25}$ = -7.5 (c = 0.58, $CHCl_3$); **^{1}H NMR** (400 MHz, $CDCl_3$) δ 8.06 – 7.95 (m, 1H), 7.82 – 7.74 (m, 2H), 7.70 – 7.62 (m, 1H), 7.39 – 7.28 (m, 2H), 7.26 (dt, *J* = 7.3, 0.8 Hz, 2H), 3.66 – 3.55 (m, 1H), 3.50 (dd, *J* = 15.4, 5.2 Hz, 1H), 3.34 – 3.24 (m, 1H), 3.04 (p, *J* = 6.7 Hz, 1H), 2.38 (s, 3H), 1.35 – 1.27 (m, 6H), 1.24 (d, *J* = 6.7 Hz, 3H); **^{13}C NMR** (101 MHz, $CDCl_3$) δ 152.8, 146.1, 142.1, 135.5, 133.1, 130.3, 126.9, 125.0, 124.8, 120.1, 113.9, 38.2, 37.3, 34.5, 24.1, 23.8, 21.8, 21.7; **IR** (film) υ_{max}/cm^{-1}: 2960, 1541, 1337, 1293, 1149, 1089, 745, 667; **HRMS** (ESI +): calcd. for $C_{20}H_{25}O_2N_2S_2$ $[M+H]^+$ 389.1352, Found 389.1352.

(*S*)-2-(2-(Cyclopentylthio)propyl)-1-tosyl-1*H*-benzo[*d*]imidazole (**5**)

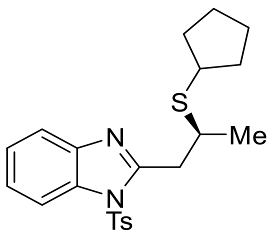

Compound **5** was synthesized according to a modified version of **GPII** from **1** (31.2 mg) using catalyst **G**, cyclopentanethiol (12.3 mg, 13 µL) and stirred at 22 °C. Clear oil (7 Petrol/ 3 EtOAc) (37.7 mg, 0.091 mmol, 91% yield, 95/5 er [determined by HPLC, Chiralcel OG, hexane/isopropanol = 97/3, 1 mL/min, λ = 220 nm, t (minor) = 10.11 min, t (major) = 11.28 min]).

$[\alpha]_D^{25}$ = -6.3 (c = 0.48, $CHCl_3$); **^{1}H NMR** (400 MHz, $CDCl_3$) δ 8.02 – 7.89 (m, 1H), 7.76 – 7.67 (m, 2H), 7.64 – 7.55 (m, 1H), 7.31 – 7.22 (m, 2H), 7.21 – 7.12 (m, 2H), 3.60 – 3.44 (m, 2H), 3.29 – 3.05 (m, 2H), 2.30 (s, 3H), 2.12 – 1.77 (m, 2H), 1.74 – 1.34 (m, 6H), 1.26 (d, *J* = 6.7 Hz, 3H); **^{13}C NMR** (101 MHz, $CDCl_3$) δ 152.9, 146.1, 142.0, 135.5, 133.1, 130.3, 126.9, 125.0, 124.8, 120.0, 113.8, 43.0, 38.5, 38.1, 34.6, 34.3, 24.9, 21.8, 21.5; **IR** (film) υ_{max}/cm^{-1}: 2957, 2960, 1541, 1377, 1293, 1149, 1089, 745, 667; **HRMS** (ESI +): calcd. for $C_{22}H_{27}O_2N_2S_2$ $[M+H]^+$ 415.1508, Found 415.1507.

2-(2-(Phenethylthio)propyl)-1-tosyl-1*H*-benzo[*d*]imidazole (**6**)

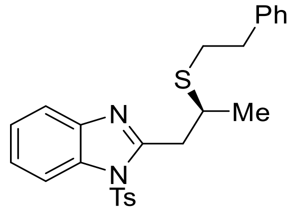

Compound **6** was synthesized according to **GPII** from **1** (31.2 mg) using catalyst **G** and 2-phenylethane-1-thiol (16.6 mg, 16 µL). Pale yellow amorphous solid (7 Petrol/ 3 EtOAc) (42.1 mg, 0.093 mmol, 93% yield, 92.5/7.5 er [determined by HPLC, Chiralpak AD-H, hexane/isopropanol = 95/5, 1 mL/min, λ = 220 nm, t (minor) = 27.01 min, t (major) = 28.93 min])

$[\alpha]_D^{25}$ = 6.9 (c = 0.71, $CHCl_3$); **^{1}H NMR** (400 MHz, $CDCl_3$) δ 8.08 – 8.01 (m, 1H) 7.78 (d, *J* = 8.4 Hz, 2H), 7.73 – 7.66 (m, 1H), 7.41 – 7.32 (m, 2H), 7.32 – 7.17 (m, 7H), 3.70 – 3.51 (m, 2H), 3.32 (dd, *J* = 15.0, 8.5 Hz, 1H), 3.01 – 2.75 (m, 4H), 2.38 (s, 3H), 1.38 (d, *J* = 6.7 Hz, 3H); **^{13}C NMR** (101 MHz, $CDCl_3$) δ 152.7, 146.1, 142.0, 140.7, 135.5, 133.1, 130.4, 128.6, 128.5, 126.8, 126.4, 125.1, 124.9, 120.1, 113.9, 38.7, 37.9, 36.5, 32.4, 21.7, 21.3; **IR** (film) υ_{max}/cm^{-1}: 2980, 1452, 1378, 1252, 1170, 1045, 668; **HRMS** (ESI +) calcd. for $C_{25}H_{27}O_2N_2S_2$ $[M+H]^+$ 451.1508, Found 451.1505.

(*S*)-1-Tosyl-2-(2-((2-(trimethylsilyl)ethyl)thio)propyl)-1*H*-benzo[*d*]imidazole (**7**)

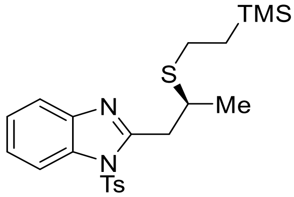

Compound **7** was synthesized according to **GPII** from **1** (31.2 mg) using catalyst **G** and 2-(trimethylsilyl)ethane-1-thiol (16.1 mg, 19 µL). Clear oil (7 Petrol/ 3 EtOAc) (41.3 mg, 0.094 mmol, 94% yield, 96/4 er [determined by HPLC, Chiralcel AD-H , hexane/isopropanol = 95/5, 1 mL/min, λ = 220 nm, t (minor) = 10.54 min, t (major) = 9.56 min]).

$[\alpha]_D^{25}$ = -11.1 (c = 0.82, $CHCl_3$); **^{1}H NMR** (400 MHz, $CDCl_3$) δ 8.09 – 7.99 (m, 1H), 7.84 – 7.77 (m, 2H), 7.73 – 7.65 (m, 1H), 7.41 – 7.32 (m, 2H), 7.32 – 7.27 (m, 2H), 3.69 – 3.50 (m, 2H), 3.32 (dd, *J* = 15.1, 8.5 Hz, 1H), 2.75 – 2.52 (m, 2H), 2.40 (s, 3H), 1.37 (d, *J* = 6.7 Hz, 3H), 0.99 – 0.77 (m, 2H), 0.01 (s, 9H); **^{13}C NMR** (101 MHz, $CDCl_3$) δ 152.9, 146.1, 142.0, 135.5, 133.2, 130.4, 126.9, 125.1, 124.9, 120.1, 113.9, 38.5, 38.0, 26.7, 21.8, 21.2, 17.4, -1.7; **IR** (film) υ_{max}/cm^{-1}: 2953, 1597, 1542, 1451, 1378, 1248, 1191, 1171, 1149, 1090, 1044, 859, 745, 687, 666; **HRMS** (APCI +) calcd. for $C_{22}H_{31}O_2N_2S_2Si$ $[M+H]^+$ 447.1591, Found 447.1589.

(*S*)-2-(2-((Furan-2-ylmethyl)thio)propyl)-1-tosyl-1*H*-benzo[*d*]imidazole (**8**)

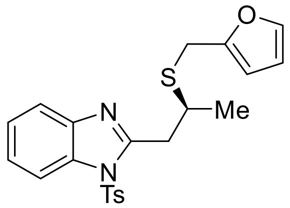

Compound **8** was synthesized according to **GPII** from **1** (31.2 mg) using catalyst **G** and furan-2-ylmethanethiol (13.7 mg, 12 µL). Clear oil (7 Petrol/ 3 EtOAc) (38.7 mg, 0.091 mmol, 91% yield, 89/11 er [determined by HPLC, Chiralcel AD-H , hexane/isopropanol = 85/15, 1 mL/min, λ = 220 nm, t (minor) = 15.18 min, t (major) = 13.72 min]).

[α]$_D^{25}$ = -27.5 (c = 0.83, $CHCl_3$); ^{1}**H NMR** (400 MHz, $CDCl_3$) δ 8.04 – 7.97 (m, 1H), 7.82 – 7.73 (m, 2H), 7.69 – 7.62 (m, 1H), 7.39 – 7.30 (m, 3H), 7.29 – 7.22 (m, 2H), 6.33 – 6.16 (m, 2H), 3.90 – 3.77 (m, 2H), 3.65 – 3.56 (m, 1H), 3.55 – 3.46 (m, 1H), 3.36 – 3.25 (m, 1H), 2.37 (s, 3H), 1.34 (d, *J* = 6.7 Hz, 3H); ^{13}**C NMR** (101 MHz, $CDCl_3$) δ 152.6, 151.9, 146.1, 142.1, 142.1, 135.5, 133.1, 130.4, 126.9, 125.1, 124.9, 120.1, 113.8, 110.6, 107.5, 38.9, 37.9, 27.9, 21.8, 21.1; **IR** (film) $υ_{max}$/cm^{-1}: 2923, 1596, 1541, 1451, 1376, 1233, 1191, 1170, 1149, 1121, 1089, 1045, 1012, 744, 703, 685, 666, 643; **HRMS** (APCI +) calcd. for $C_{22}H_{23}O_3N_2S_2$ $[M+H]^+$ 427.1144, Found 427.1141.

(*S*)-2-(2-(Benzylthio)propyl)-1-tosyl-1*H*-benzo[*d*]imidazole (**9**)

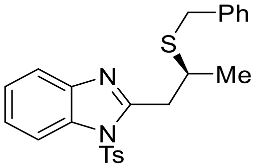

Compound **9** was synthesized according to **GPII** from **1** (31.2 mg) using catalyst **G** and phenylmethanethiol (14.9 mg, 14 µL). Clear oil (7 Petrol/ 3 EtOAc) (41.2 mg, 0.089 mmol, 89% yield, 89/11 er [determined by HPLC, Chiralpak AD-H, hexane/isopropanol = 95/5, 1 mL/min, λ = 220 nm, t (major) = 24.54 min, t (minor) = 29.98 min]).

[α]$_D^{25}$ = -19.5 (c = 0.73, $CHCl_3$) ^{1}**H NMR** (400 MHz, $CDCl_3$) δ 7.95 – 7.88 (m, 1H), 7.65 (d, *J* = 8.4 Hz, 2H), 7.61 – 7.53 (m, 1H), 7.31 – 7.07 (m, 10H), 3.75 (d, *J* = 4.3 Hz), 3.54 – 3.42 (m, 2H), 3.28 – 3.13 (m, 1H), 2.28 (s, 3H,), 1.24 (d, *J* = 6.5 Hz, 3H); ^{13}**C NMR** (101 MHz, $CDCl_3$) δ 152.6, 146.1, 142.0, 138.5, 135.5, 133.1, 130.3, 128.9, 128.6, 127.0, 126.8, 125.0, 124.8, 120.1, 113.8, 38.9, 37.8, 35.7, 21.8, 21.3; **IR** (film) $υ_{max}$/cm^{-1}: 3060, 3028, 2964, 1451, 1376, 1170, 1044, 643; **HRMS** (ESI +) calcd. for $C_{24}H_{25}O_2N_2S_2$ $[M+H]^+$ 437.1352, Found 437.1344.

(*S*)-2-(2-((4-Methoxybenzyl)thio)propyl)-1-tosyl-1*H*-benzo[*d*]imidazole (**10**)

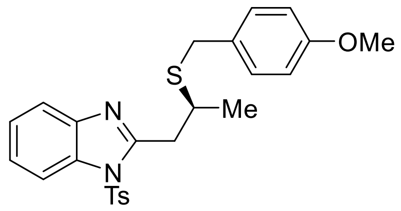

Compound **10** was synthesized according to **GPII** from **1** (31.2 mg) using catalyst **G** and (4-methoxyphenyl)methanethiol (18.5 mg, 17 µL). Amorphous colourless solid (7 Petrol/ 3 EtOAc) (45.5 mg, 0.098 mmol, 98% yield, 92/8 er [determined by HPLC, Chiralpak AD-H, hexane/isopropanol = 85/15, 1 mL/min, λ = 220 nm, t (major) = 14.95 min, t (minor) = 18.30 min]).

$[\alpha]_D^{25}$ = -19.9 (c = 0.52, $CHCl_3$); **^{1}H NMR** (400 MHz, $CDCl_3$) δ 8.05 – 7.98 (m, 1H), 7.80 – 7.71 (m, 2H), 7.70 – 7.62 (m, 1H), 7.40 – 7.29 (m, 2H), 7.29 – 7.16 (m, 4H), 6.85 – 6.76 (m, 2H), 3.78 (s + m, 5H), 3.60 – 3.47 (m, 2H), 3.36 – 3.20 (m, 1H), 2.37 (s, 3H), 1.33 (d, *J* = 6.5 Hz, 3H); **^{13}C NMR** (101 MHz, $CDCl_3$) δ 158.6, 152.7 , 146.1, 142.1, 135.5, 133.1, 130.4, 130.3, 130.0, 125.1, 124.9, 120.1, 114.0, 113.9, 55.4, 38.8, 37.9, 35.1, 21.8, 21.3; **IR** (film) υ_{max}/cm^{-1}: 3023, 1541, 1500, 1451, 1376, 1171, 1013, 884, 668; **HRMS** (ESI+) calcd. for $C_{25}H_{27}O_3N_2S_2$ [M+H]+ 467.1457, Found 467.1454.

(*S*)-2-(2-(Propylthio)hept-6-en-1-yl)-1-tosyl-1*H*-benzo[*d*]imidazole (**11**)

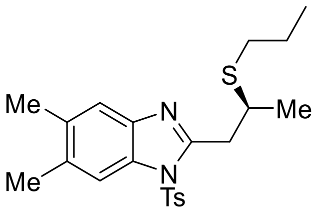

Compound **11** was synthesized according **GP III** from **Bz2** (34.0 mg) using catalyst **G**, 1-propanethiol (9.1 mg, 11 µL). Clear oil (9 Pentane/ 1 EtOAc) (33.7 mg, 0.081 mmol, 81% yield, 86/14 er [determined by HPLC, Chiralcel AD-H, hexane/isopropanol = 95/5, 1 mL/min, λ = 220 nm, t (minor) = 22.89 min, t (major) = 21.24 min]).

$[\alpha]_D^{25}$ = -3.5 (c = 1.0, $CHCl_3$); **^{1}H NMR** (400 MHz, $CDCl_3$) δ = 7.78 (s, 1H), 7.75 (d, *J* = 8.4 Hz, 2H), 7.41 (d, *J* = 0.9 Hz, 1H), 7.26 (dd, *J* = 8.5, 1.0 Hz, 2H), 3.56 – 3.44 (m, 2H), 3.27 – 3.18 (m, 1H), 2.63 – 2.48 (m, 2H), 2.38 (s, 3H), 2.37 (s, 3H), 2.32 (s, 3H), 1.67 – 1.53 (m, 2H), 1.30 (d, *J* = 6.8 Hz, 4H), 0.97 (t, *J* = 7.3 Hz, 3H); **^{13}C NMR** (101 MHz, $CDCl_3$) δ = 152.0, 145.9, 140.5, 135.8, 134.3, 133.8, 131.6, 130.3, 126.7, 120.2, 114.1, 38.5, 37.9, 33.0, 23.3, 21.7, 21.2, 20.8, 20.3, 13.7; **IR** (film) υ_{max}/cm^{-1}: 2962, 2925, 1596, 1463, 1375, 1230, 1190, 1172, 1090, 1037, 811, 667; **HRMS** (ESI+): calcd. for $C_{22}H_{29}O_2N_2S_2$ $[M+H]^+$ 417.1665, found 417.1663.

(*S*)-6-Bromo-2-(2-(propylthio)propyl)-1-tosyl-1*H*-benzo[*d*]imidazole (**12**)

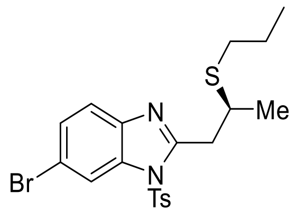

Compound **12** was synthesized according **GP III** from **Bz5** (39.1 mg) using catalyst **G**, 1-propanethiol (9.1 mg, 11 µL). Clear oil (95 Pentane/ 5 EtOAc) (36.0 mg, 0.077 mmol, 77% yield, 86/14 er [determined by HPLC, Chiralcel AD-H, hexane/isopropanol = 95/5, 1 mL/min, λ = 220 nm, t (minor) = 17.65 min, t (major) = 15.93 min]).

$[\alpha]_D^{25}$ = -5.9 (c = 1.0, $CHCl_3$); **^{1}H NMR** (400 MHz, $CDCl_3$) δ 8.21 (d, *J* = 1.8 Hz, 1H), 7.78 (d, *J* = 8.4 Hz, 2H), 7.52 (d, *J* = 8.6 Hz, 1H), 7.44 (dd, *J* = 8.5, 1.8 Hz, 1H), 7.31 (d, *J* = 7.8 Hz, 2H), 3.57 – 3.41 (m, 2H), 3.24 (dd, *J* = 15.4, 8.5 Hz, 1H), 2.62 – 2.49 (m, 2H), 2.40 (s, 3H), 1.61 (h, *J* = 7.3 Hz, 3H), 1.32 (d, *J* = 6.5 Hz, 4H), 0.97 (t, *J* = 7.3 Hz, 3H); **^{13}C NMR** (101 MHz, $CDCl_3$) δ = 153.5, 146.5, 141.1, 135.3, 134.1, 130.5, 128.3, 127.0, 121.2, 118.5, 117.0, 38.4, 37.9, 33.0, 23.3, 21.9, 21.4, 13.7; **IR** (film) υ_{max}/cm^{-1}: 2961, 2926, 1730, 1596, 1447, 1424, 1379, 1269, 1191, 1162, 1089, 1037, 939, 813, 708, 664; **HRMS** (ESI+): calcd. for $C_{20}H_{24}O_2N_2BrS_2$ $[M+H]^+$ 467.0457 and 469.0436, found. 467.04565 and 469.0433

(*S*)-5-Bromo-2-(2-(propylthio)propyl)-1-tosyl-1*H*-benzo[*d*]imidazole (**13**)

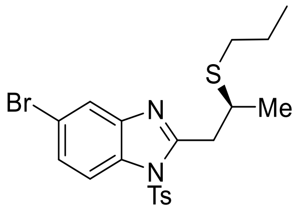

Compound **13** was synthesized according **GP III** from **Bz6** (39.1 mg) using catalyst **G**, 1-propanethiol (9.1 mg, 11 µL). Clear oil (95 Pentane/ 5 EtOAc) (38.8 mg, 0.083 mmol, 81% yield, 84/16 er [determined by HPLC, Chiralcel AD-H, hexane/isopropanol = 95/5, 1 mL/min, λ = 220 nm, t (minor) = 19.17 min, t (major) = 20.44 min]).

$[\alpha]_D^{25}$ = -4.4 (c = 1.0, $CHCl_3$); **^{1}H NMR** (400 MHz, $CDCl_3$) δ 7.89 (d, *J* = 8.7 Hz, 1H), 7.80 (d, *J* = 1.9 Hz, 1H), 7.75 (d, *J* = 8.4 Hz, 2H), 7.45 (dd, *J* = 8.8, 1.9 Hz, 1H), 7.32 – 7.25 (m, 2H), 3.57 – 3.43 (m, 2H), 3.32 – 3.20 (m, 1H), 2.64 – 2.47 (m, 2H), 2.39 (s, 3H), 1.68 – 1.54 (m, 3H), 1.33 (d, *J* = 6.8 Hz, 3H), 0.97 (t, *J* = 7.3 Hz, 3H); **^{13}C NMR** (101 MHz, $CDCl_3$) δ 154.1 , 146.5 , 143.4 , 135.3 , 132.2 , 130.5 , 128.0 , 126.9 , 123.1 , 118.0 , 115.1 , 38.4 , 37.9 , 33.0 , 23.3 , 21.8 , 21.4 , 13.7; **IR** (film) υ_{max}/cm^{-1}: 2921, 2926, 1596, 1535, 1448, 1379, 1230, 1172, 1089, 1044, 910, 809, 705, 665; **HRMS** (ESI+): calcd. for $C_{20}H_{24}O_2N_2BrS_2$ $[M+H]^+$ 467.0457 and 469.0436, found 467.0454 and 469.0431.

Benzyl-2-(2-(propylthio)propyl)-1*H*-benzo[*d*]imidazole-1-carboxylate (**14**)

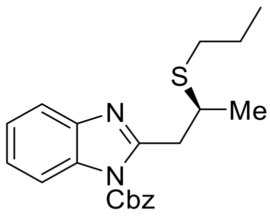

Compound **14** was synthesized according to a modified version of **GPII** from **Bz3** (29.2 mg) using catalyst **G**, 3 equivalents of 1-propanethiol (22.8 mg, 0.3 mmol, 28 µL) and stirred at 22 °C. Clear oil (9 Petrol/ 1 EtOAc) (22.3 mg, 0.061 mmol, 61% yield, 92.5/7.5 er [determined by HPLC, Chiralpak AS-H, hexane/isopropanol = 97/3, 1 mL/min, λ = 220 nm, t (minor) = 10.23 min, t (major) = 11.45 min])

$[\alpha]_D^{25}$ = +2.0 (c = 0.40, $CHCl_3$); **^{1}H NMR** (400 MHz, $CDCl_3$) δ 7.92 – 7.83 (m, 1H), 7.75 – 7.65 (m, 1H) 7.55 – 7.49 (m, 2H), 7.47 – 7.38 (m, 3H), 7.35 – 7.25 (m, 2H), 5.51 (d, *J* = 1.0 Hz, 2H), 3.63 (dd, *J* = 14.6, 5.3 Hz, 1H), 3.47 – 3.36 (m, 1H), 3.31 (dd, *J* = 14.6, 8.8 Hz, 1H), 2.67 – 2.44 (m, 2H), 1.59 (h, *J* = 7.4 Hz, 2H), 1.34 (d, *J* = 6.7 Hz, 3H), 0.96 (t, *J* = 7.3 Hz, 3H); **^{13}C NMR** (101 MHz, $CDCl_3$) δ 154.3, 150.5, 142.3, 134.3, 132.8, 129.3, 129.1, 129.0, 124.8, 124.6, 119.9, 115.2, 69.8, 38.9, 38.2, 32.7, 23.3, 21.4, 13.7; **IR** (film) υ_{max}/cm^{-1}: 2964, 1749, 1455, 1385, 1338, 1296, 1258, 1194, 1088, 1081, 746; **HRMS** (ESI +) calcd. for $C_{21}H_{25}O_2N_2S$ $[M+H]^+$ 369.1631, Found 369.1626.

tert-butyl-2-(2-(propylthio)propyl)-1*H*-benzo[*d*]imidazole-1-carboxylate (**15**)

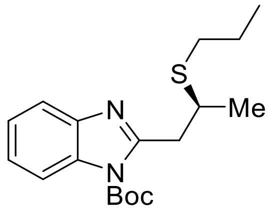

Compound **15** was synthesized according to a modified version of **GPII** from **Bz4** (25.8 mg) using catalyst **G**, 3 equivalents of 1-propanethiol (22.8 mg, 0.3 mmol, 28 µL) and stirred at 22 °C. Clear oil (8 Petrol/ 2 EtOAc) (32.7 mg, 0.097 mmol, 97% yield, 91.5/8.5 er [determined by HPLC, Chiralpak AD-H, hexane/isopropanol = 97/3, 1 mL/min, λ = 220 nm, t (major) = 7.30 min, t (minor) = 6.61 min])

$[\alpha]_D^{25}$ = - 3.6 (c = 0.43, $CHCl_3$); **^{1}H NMR** (400 MHz, $CDCl_3$) δ 7.93 – 7.85 (m, 1H), 7.75 – 7.61 (m, 1H), 7.35 – 7.27 (m, 2H), 3.64 (dd, *J* = 14.7, 5.3 Hz, 1H), 3.43 (ddd, *J* = 9.0, 6.8, 5.3 Hz, 1H, 3.30 (dd, *J* = 14.7, 9.0 Hz, 1H), 2.66 – 2.46 (m, 2H), 1.72 (s, 9H), 1.60 (dt, *J* = 14.7, 7.4 Hz, 2H), 1.37 (d, *J* = 6.7 Hz, 3H), 0.97 (t, *J* = 7.3 Hz, 3H); **^{13}C NMR** (101 MHz, $CDCl_3$) δ 154.4, 149.1, 142.2, 137.7, 124.4, 124.2, 119.8, 115.1, 85.7, 38.9, 38.1, 32.6, 28.2, 23.3, 21.3, 13.7 ; **IR** (film) υ_{max}/cm^{-1}: 2970, 1745, 1454, 1341, 1153, 1120, 745; **HRMS** (ESI +) calcd. for $C_{18}H_{27}O_2N_2S$ $[M+H]^+$ 335.1787, Found 335.1787

(*S*)-2-(2-(Propylthio)hexyl)-1-tosyl-1*H*-benzo[*d*]imidazole (**16**)

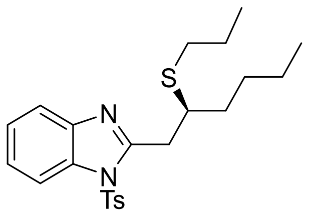

Compound **16** was synthesized according **GP III** from **Bz7** (35.5 mg) using catalyst **G,** 1-propanethiol (9.1 mg, 11 µL). Clear oil (7 Pentane/ 3 EtOAc) (35.7 mg, 0.083 mmol, 83% yield, 93/7 er [determined by HPLC, Chiralcel AD-H, hexane/isopropanol = 95/5, 1 mL/min, λ = 220 nm, t (minor) = 13.39 min, t (major) = 22.26 min]).

[α]$_D^{25}$ = -14.9 (c = 1.0, $CHCl_3$). **^{1}H NMR** (400 MHz, $CDCl_3$) δ = 8.06 – 7.98 (m, 1H), 7.78 (d, *J* = 8.4 Hz, 2H), 7.70 – 7.64 (m, 1H), 7.41 – 7.30 (m, 2H), 7.27 (d, *J* = 8.4 Hz, 2H), 3.55 – 3.31 (m, 3H), 2.58 – 2.42 (m, 2H), 2.38 (s, 3H), 1.66 – 1.49 (m, 5H), 1.38 – 1.22 (m, 3H), 0.93 (t, *J* = 7.3 Hz, 3H), 0.86 (t, *J* = 7.2 Hz, 3H); **^{13}C NMR** (101 MHz, $CDCl_3$) δ = 153.2, 146.1, 142.1, 135.7, 133.2, 130.3, 126.9, 125.0, 124.8, 120.1, 113.9, 44.3, 36.9, 34.5, 33.1, 29.0, 23.3, 22.7, 21.8, 14.2, 13.7; **IR** (film) υ_{max}/cm^{-1}: 2958, 2928, 2871, 1451, 1377, 1237, 1189, 1177, 1148, 1120, 1089, 1045, 1014, 812, 765, 745, 703, 666, 623; **HRMS** (ESI+): calcd. for $C_{23}H_{31}O_2N_2S_2$ $[M+H]^+$ 431.1820, found 431.1821.

(*S*)-2-(4-Phenyl-2-(propylthio)butyl)-1-tosyl-1*H*-benzo[*d*]imidazole (**17**)

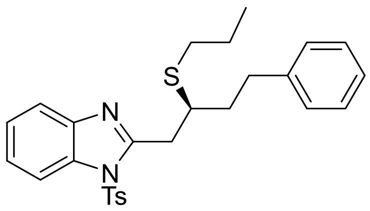

Compound **17** was synthesized according **GP III** from **Bz8** (40.3 mg) using catalyst **G**, 1-propanethiol (9.1 mg, 11 µL). Clear oil (7 Pentane/ 3 EtOAc) (43.3 mg, 0.090 mmol, 90 % yield, 96/4 er [determined by HPLC, Chiralcel OG, hexane/isopropanol = 97/3 , 1 mL/min, λ = 220 nm, t (minor) = 13.04 min, t (major) = 15.69 min]).

[α]$_D^{25}$ = -9.1 (c = 1.12, $CHCl_3$); **^{1}H NMR** (400 MHz, $CDCl_3$) δ 8.09 – 8.00 (m, 1H), 7.84 – 7.76 (m, 2H), 7.74 – 7.64 (m, 1H), 7.43 – 7.32 (m, 2H), 7.31 – 7.24 (m, 4H), 7.23 – 7.13 (m, 3H), 3.66 – 3.56 (m, 1H), 3.55 – 3.35 (m, 2H), 2.95 (ddd, *J* = 13.7, 10.7, 5.0 Hz, 1H), 2.83 – 2.66 (m, 1H), 2.66 – 2.48 (m, 2H), 2.38 (s, 3H), 2.06 – 1.95 (m, 1H), 1.87 (dddd, *J* = 13.9, 10.7, 8.1, 5.0 Hz, 1H), 1.68 – 1.59 (m, 2H), 0.99 (t, *J* = 7.3 Hz, 3H); **^{13}C NMR** (101 MHz, $CDCl_3$) δ 152.8, 146.1, 142.1, 141.9, 135.6, 133.2, 130.3, 128.5, 128.4, 126.8, 125.9, 125.0, 124.8, 120.1, 113.9, 43.9, 36.8, 36.3, 33.1, 33.0, 23.3, 21.8, 13.7; **IR** (film) υ_{max}/cm^{-1}: 3026, 2960, 2925, 2870, 1597, 1452, 1190, 1176, 1147, 1121, 1089, 1047, 812, 765, 745, 701, 667, 643; **HRMS** (APCI+): calcd. for $C_{27}H_{31}O_2N_2S_2$ $[M+H]^+$ 479.1821, found 479.1816.

(*S*)-2-(2-(Propylthio)hept-6-en-1-yl)-1-tosyl-1*H*-benzo[*d*]imidazole (**18**)

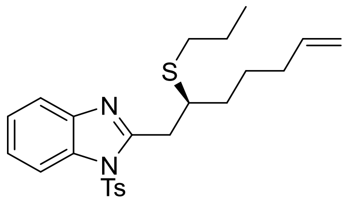

Compound **18** was synthesized according **GP III** from **Bz9** (36.6 mg) using catalyst **G**, 1-propanethiol (9.1 mg, 11 µL). Clear oil (8 Pentane/ 2 EtOAc) (34.1 mg, 0.077 mmol, 77% yield, 94.5/5.5 er [determined by HPLC, Chiralcel I-B, hexane/isopropanol = 98/2, 1 mL/min, λ = 220 nm, t (minor) = 8.41 min, t (major) = 8.87 min]).

[α]$_D$^{25} = -17.9 (c = 1.0, $CHCl_3$); **^{1}H NMR** (400 MHz, $CDCl_3$) δ = 8.05 – 7.98 (m, 1H), 7.77 (d, *J* = 8.4 Hz, 2H), 7.69 – 7.64 (m, 1H), 7.37 – 7.29 (m, 2H), 7.26 (d, *J* = 8.6 Hz, 2H), 5.75 (ddt, *J* = 16.9, 10.2, 6.6 Hz, 1H), 4.96 (dq, *J* = 17.1, 1.6 Hz, 1H), 4.90 (ddt, *J* = 10.2, 2.1, 1.1 Hz, 1H), 3.51 (dd, *J* = 14.0, 5.3 Hz, 1H), 3.45 – 3.39 (m, 1H), 3.36 (dd, *J* = 14.6, 7.7 Hz, 1H), 2.59 – 2.41 (m, 2H), 2.37 (s, 3H), 2.05 – 1.98 (m, 2H), 1.72 – 1.47 (m, 7H), 0.93 (t, *J* = 7.3 Hz, 3H); **^{13}C NMR** (101 MHz, $CDCl_3$) δ = 153.0, 146.1, 142.1, 138.7, 135.6, 133.2, 130.3, 126.9, 125.0, 124.8, 120.9, 114.7, 113.9, 77.5, 77.2, 76.8, 44.1, 36.9, 34.1, 33.6, 33.1, 26.1, 23.3, 21.8, 13.7; **IR** (film) υ_{max}/cm^{-1}: 2930, 2859, 1735, 1452, 1378, 1247, 1177, 1089, 1045, 745, 666; **HRMS** (ESI+): calcd. for $C_{24}H_{31}O_2N_2S_2$ [M+H]$^+$ 443.1815, found 443.1821.

(*S*)-2-(2-(Propylthio)hex-5-yn-1-yl)-1-tosyl-1*H*-benzo[*d*]imidazole (**19**)

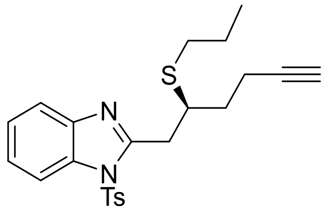

Compound **19** was synthesized according **GP III** from **Bz10** (35.0 mg) using catalyst **G**, 1-propanethiol (9.1 mg, 11 µL). Clear oil (8 Pentane/ 2 Et_2O) (34.1 mg, 0.080 mmol, 80% yield, 96/4 er [determined by HPLC, Chiralcel AD-H, hexane/isopropanol = 97/3, 1 mL/min, λ = 220 nm, t (minor) = 35.17 min, t (major) = 37.67 min]).

[α]$_D$^{25} = -17.8 (c = 1.0, $CHCl_3$); **^{1}H NMR** (400 MHz, $CDCl_3$) δ =8.04 – 7.97 (m, 1H), 7.78 (d, *J* = 8.4 Hz, 2H), 7.69 – 7.65 (m, 1H), 7.38 – 7.30 (m, 2H), 7.27 (d, *J*=7.7, 2H), 3.56 (dd, *J* = 14.5, 6.2 Hz, 1H), 3.53 – 3.46 (m, 1H), 3.38 (dd, *J* = 14.5, 7.2 Hz, 1H), 2.61 – 2.46 (m, 2H), 2.45 – 2.34 (m+s, 5H), 2.00 – 1.89 (m, 1H), 1.93 (t, *J* = 2.6 Hz, 1H), 1.80 – 1.71 (m, 1H), 1.58 (dqd, *J* = 14.7, 7.2, 1.8 Hz, 2H), 0.93 (t, *J* = 7.3 Hz, 3H); **^{13}C NMR** (101 MHz, $CDCl_3$) δ = 152.5, 146.1, 142.1, 135.5, 133.2, 130.4, 126.9, 125.9, 124.9, 120.2, 113.9, 83.9, 77.5, 77.2, 76.8, 68.9, 43.5, 36.9, 33.5, 33.2, 29.8, 23.3, 21.8, 16.3, 13.7; **IR** (film) υ_{max}/cm^{-1}: 3298, 3058, 2960, 2923, 2852, 1596, 1540, 1451, 1376, 1189, 1047, 765, 666; **HRMS** (ESI+): calcd. for $C_{23}H_{27}O_2N_2S_2$ [M+H]$^+$ 427.1506, found 427.1508.

(*R*)-2-(2-Phenyl-2-(propylthio)ethyl)-1-tosyl-1*H*-benzo[*d*]imidazole (**20**)

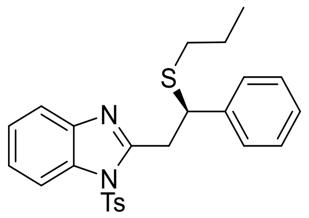

Compound **20** was synthesized according to a modified version of **GPII** from **Bz1** (37.4 mg) using catalyst **F**, 1-propanethiol (22.8 mg, 11 µL) in THF (1.6 mL) and stirred at 22 °C. Amorphous white solid (Petrol 7/ 3 EtOAc) (39.1 mg, 0.087 mmol, 87% yield, 88/12 er [determined by HPLC, Chiralpak AD-H, hexane/isopropanol = 97/3, 1 mL/min, λ = 220 nm, t (major) = 42.23 min, t (minor) = 62.91 min]).

$[\alpha]_D^{25}$ = +5.8 (c = 0.79, $CHCl_3$); **^{1}H NMR** (400 MHz, $CDCl_3$) δ 7.93 – 7.90 (m, 1H), 7.68 – 7.66 (m, 2H), 7.65 – 7.58 (m, 1H), 7.43 – 7.36 (m, 2H), 7.33 – 7.11 (m, 7H), 4.71 (t, *J* = 7.5 Hz, 1H), 3.88 – 3.62 (m, 2H), 2.33 (s, 3H), 2.32 – 2.17 (m, 2H), 1.54 – 1.37 (m, 2H), 0.83 (t, *J* = 7.3 Hz, 3H); **^{13}C NMR** (101 MHz, $CDCl_3$) δ 152.1, 146.0, 142.1, 142.0, 135.5, 133.1, 130.3, 128.6, 128.0, 127.3, 126.9, 125.0, 124.8, 120.2, 113.8, 47.6, 37.2, 33.6, 22.7, 21.8, 13.6; **IR** (film) υ_{max}/cm^{-1}: 2981, 1627, 1597, 1449, 1378, 1378, 1149, 1089, 1051, 669; **HRMS** (ESI +) calcd. for $C_{25}H_{27}O_2N_2S_2$ [M+H]$^+$ 451.1508, Found 451.1505.

(*R*)-2-(2-(4-Chlorophenyl)-2-(propylthio)ethyl)-1-tosyl-1*H*-benzo[*d*]imidazole (**21**)

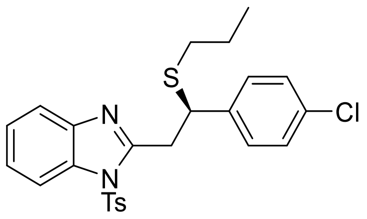

Compound **21** was synthesized according **GP III** from **Bz11** (40.9 mg) using catalyst **G**, 1-propanethiol (22.8 mg, 11 µL) in THF (1.6 mL) and stirred at 22 °C. Clear oil (8 Pentane/ 2 EtOAc) (29.6 mg, 0.061 mmol, 61% yield, 91/9 er [determined by HPLC, Chirapak AD-H, hexane/isopropanol = 95/5, 1 mL/min, λ = 220 nm, t (minor) = 28.80 min, t (major) = 22.55 min]).

$[\alpha]_D^{25}$ = +12.3 (c = 0.32, $CHCl_3$); **^{1}H NMR** (400 MHz, $CDCl_3$) δ 7.99 – 7.91 (m, 1H), 7.67 (d, *J* = 8.5 Hz, 2H), 7.67 – 7.59 (m, 1H), 7.38 – 7.28 (m, 4H), 7.27 – 7.17 (m, 4H), 4.70 (dd, *J* = 8.1, 7.0 Hz, 1H), 3.70 (qd, *J* = 15.9, 7.5 Hz, 2H), 2.38 (s, 3H), 2.31 (td, *J* = 7.8, 6.8 Hz, 2H), 1.66 – 1.40 (m, 2H), 0.88 (t, *J* = 7.3 Hz, 3H); **^{13}C NMR** (101 MHz, $CDCl_3$) δ 151.8, 146.1, 142.0, 140.6, 135.5, 133.1, 132.9, 130.4, 129.5, 128.7, 126.8, 125.1, 124.9, 120.2, 113.8, 46.9, 37.2, 33.7, 22.7, 21.8, 13.6; **HRMS** (APCI+): calcd. for $C_{25}H_{26}O_2N_2ClS_2$ [M+H]$^+$ 485.1118, found 485.1117.

(*R*)-2-(2-(4-Nitrophenyl)-2-(propylthio)ethyl)-1-tosyl-1*H*-benzo[*d*]imidazole (**22**)

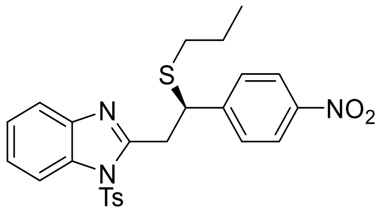

Compound **22** was synthesized according **GP III** from **Bz12** (41.9 mg) using catalyst **G**, 1-propanethiol (22.8 mg, 11 µL) in THF (1.6 mL) and stirred at 22 °C. Yellow oil (8 Pentane/ 2 EtOAc) (33.4 mg, 0.067 mmol, 67% yield, 88/12 er [determined by HPLC, Chirapak AD-H, hexane/isopropanol = 80/20, 1 mL/min, λ = 220 nm, t (minor) = 12.69 min, t (major) = 16.93 min]).

[α]$_{\mathbf{D}}^{\mathbf{25}}$ = +15.7 (c = 1.21, $CHCl_3$); ^{1}**H NMR** (400 MHz, $CDCl_3$) δ 8.10 – 8.08 (m, 2H), 8.01 – 7.95 (m, 1H), 7.76 – 7.70 (m, 2H), 7.67 – 7.61 (m, 1H), 7.60 – 7.55 (m, 2H), 7.34 (ddd, *J* = 7.0, 5.0, 1.7 Hz, 2H), 7.28 (dd, *J* = 8.9, 1.0 Hz, 2H), 4.83 (dd, *J* = 8.4, 6.8 Hz, 1H), 3.84 – 3.67 (m, 2H), 2.39 (s, 3H), 2.38 – 2.23 (m, 2H), 1.65 – 1.41 (m, 2H), 0.91 (t, *J* = 7.3 Hz, 3H); ^{13}**C NMR** (101 MHz, $CDCl_3$) δ 151.1, 149.8, 147.1, 146.3, 141.8, 135.4, 133.1, 130.4, 129.0, 126.8, 125.3, 125.0, 123.8, 120.2, 113.8, 46.9, 36.8, 33.8, 22.6, 21.8, 13.5; **IR** (film) υ_{max}/cm^{-1}: 2962, 2929, 1596, 1519, 1451, 1371, 1349, 1189, 1049, 745, 668; **HRMS** (APCI+): calcd. for $C_{25}H_{26}O_4N_3S_2$ $[M+H]^+$ 496.1359, found 496.1356.

(*R*)-2-(2-(3-Chlorophenyl)-2-(propylthio)ethyl)-1-tosyl-1*H*-benzo[*d*]imidazole (**23**)

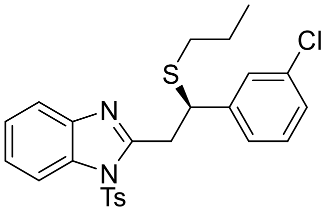

Compound **23** was synthesized according **GP III** from **Bz13** (40.1 mg) using catalyst **G**, 1-propanethiol (22.8 mg, 11 µL) in THF (1.6 mL) and stirred at 22 °C. Clear oil (7 Pentane/ 3 EtOAc) (29.9 mg, 0.062 mmol, 62% yield, 93/7 er [determined by HPLC, Chiralpak AD-H hexane/isopropanol = 90/10, 1 mL/min, λ = 220 nm, t (minor) =17.19 min, t (major) = 11.32 min]).

[α]$_{\mathbf{D}}^{\mathbf{25}}$ = +17.1 (c = 0.66, $CHCl_3$); ^{1}**H NMR** (400 MHz, $CDCl_3$) δ 8.00 – 7.94 (m, 1H), 7.75 – 7.69 (m, 2H), 7.67 – 7.61 (m, 1H), 7.42 – 7.38 (m, 1H), 7.34 – 7.28 (m, 3H), 7.25 (m, 2H), 7.22 – 7.10 (m, 2H), 4.70 (t, *J* = 7.5 Hz, 1H), 3.71 (t, *J* = 7.6 Hz, 2H), 2.37 (s, 3H), 2.34 – 2.20 (m, 2H), 1.55 – 1.44 (m, 2H), 0.87 (t, *J* = 7.5 Hz, 3H); ^{13}**C NMR** (101 MHz, $CDCl_3$) δ 151.7, 146.1, 144.3, 142.0, 135.5, 134.4, 133.2, 130.4, 129.8, 128.1, 127.6, 126.8, 126.4, 125.1, 124.8, 120.2, 113.8, 47.0, 37.1, 33.7, 22.6, 21.8, 13.6. **IR** (film) υ_{max}/cm^{-1}: 3058, 2961, 1596, 1452, 1377, 1189, 1089, 1048, 765, 668; **HRMS** (APCI+): calcd. for $C_{25}H_{26}O_2N_2ClS_2$ $[M+H]^+$ 485.1118, found 485.1118.

(*R*)-2-(2-(Propylthio)-2-(6-(trifluoromethyl)pyridin-3-yl)ethyl)-1-tosyl-1*H*-benzo[*d*]imidazole (**24**)

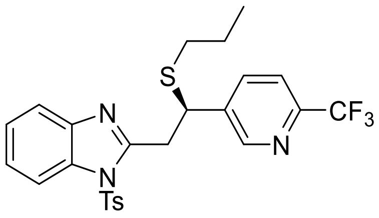

Compound **24** was synthesized according **GP III** from **Bz14** (44.3 mg) using catalyst **G**, 1-propanethiol (22.8 mg, 11 µL) in THF (1.6 mL) and stirred at 22 °C. Clear oil (8 Pentane/ 2 EtOAc) (38.4 mg, 0.074 mmol, 74% yield, 90/10 er [determined by HPLC, Chiralpak IA, hexane/isopropanol = gradient 995/005 to 70/30, 1 mL/min, λ = 220 nm, t (minor) = 41.05 min, t (major) = 42.29 min]).

$[\alpha]_D^{25}$ = +13.2 (c = 0.54, $CHCl_3$); **^{1}H NMR** (400 MHz, $CDCl_3$) δ 8.74 (d, *J* = 2.1 Hz, 1H), 7.99 (ddd, *J* = 7.9, 1.9, 1.0 Hz, 2H), 7.75 – 7.68 (m, 2H), 7.66 – 7.59 (m, 2H), 7.35 (ddd, *J* = 7.0, 4.8, 1.7 Hz, 2H), 7.28 (dd, *J* = 8.6, 1.0 Hz, 2H), 4.83 (dd, *J* = 8.3, 6.8 Hz, 1H), 3.78 (dd, *J* = 7.6, 4.0 Hz, 2H), 2.40 (s, 3H), 2.39 – 2.26 (m, 2H), 1.65 – 1.47 (m, 2H), 0.93 (t, *J* = 7.4 Hz, 3H); **^{13}C NMR** (101 MHz, $CDCl_3$) δ 150.8, 150.1, 147.0 (q, *J* = 34.6 Hz), 146.4, 141.8, 141.3, 136.8, 135.4, 133.1, 130.5, 126.7, 125.4, 125.0, 120.4 (d, *J* = 2.6 Hz), 120.2, 113.8, 44.3, 36.8, 33.8, 22.6, 21.8, 13.5; **^{19}F NMR** (376 MHz, $CDCl_3$) δ -67.8; **IR** (film) υ_{max}/cm^{-1}: 3057, 2963, 2931, 1452, 1378, 1337, 1174, 1137, 1088, 1051, 745, 669; **HRMS** (APCI+): calcd. for $C_{25}H_{25}O_2N_3F_3S_2$ $[M+H]^+$ 520.1334, found 520.1333.

7/ Cleavage of *N*-Ts group (25)

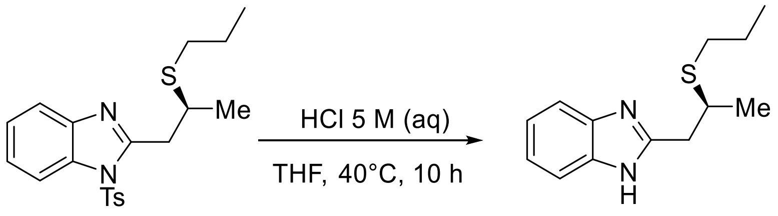

2 25

To a solution of **2** (50 mg, 0.129 mmol) in THF (1 mL) was added 5 M aq. HCl (1 mL). The mixture was stirred at 40°C for 10 h. The reaction was quenched with a solution of saturated $NaHCO_3$ and the aqueous phase extracted twice with EtOAc (5 mL). The combined organics were washed with brine, dried over $MgSO_4$ and concentrated under vaccum. The residue was purified by flash chromatography with pure EtOAc affording product **25** as a white solid (30.2 mg, 0.129 mmol, 100% yield and 95.5/4.5 er) [determined by HPLC, Chiralcel OD-H, hexane/isopropanol = 95/5, 1 mL/min, λ = 220 nm, t (minor) = 33.17 min, t (major) = 41.12 min]).

Mp : 94°C (from EtOAc); **$[\alpha]_D^{25}$** = -38.9 (c = 0.4, $CHCl_3$); **^{1}H NMR** (400 MHz, MeOD) δ =7.48 (dd, *J* = 6.1, 3.2 Hz, 2H), 7.18 (dd, *J* = 6.1, 3.2 Hz, 2H), 4.87 (s, 2H), 3.34 – 3.23 (m, 2H), 3.14 (dd, *J* = 14.3, 6.7 Hz, 1H), 2.97 (dd, *J* = 14.4, 8.0 Hz, 1H), 2.48 (td, *J* = 7.2, 3.5 Hz, 2H), 1.55 (h, *J* = 7.3 Hz, 2H), 1.27 (d, *J* = 6.8 Hz, 3H), 0.91 (t, *J* = 7.3 Hz, 3H); **^{13}C NMR** (101 MHz, MeOD) δ = δ 152.4, 121.3, 113.4, 38.2, 36.2, 31.6, 22.1, 19.8, 11.7; **IR** (film) υ_{max}/cm^{-1}: 3054, 2959, 2869, 1538, 1453, 1437, 1376, 1272, 1026, 742; **HRMS** (ESI+): calcd. for $C_{13}H_{19}N_2S$ $[M+H]^+$ 235.1263, found 235.1263.

8/ Oxidation of sulfur (26)

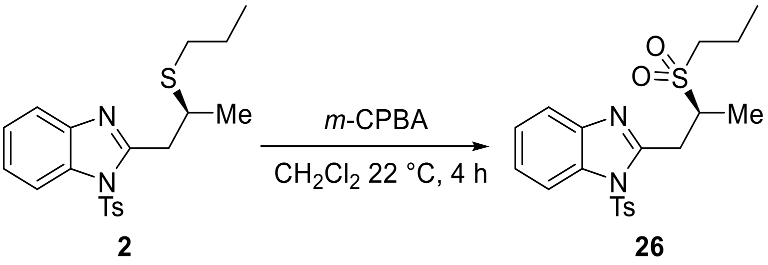

To a solution of **2** (40 mg, 0.103 mmol) in CH_2Cl_2 (1 mL) at 22 °C was added *m*-CPBA (77% in water) (53 mg, 0.236 mmol). The mixture was stirred for 4h. The reaction was diluted with CH_2Cl_2 (5 ml) and quenched with a solution of saturated $Na_2S_2O_3$. The organic layer was washed with a solution of saturated $NaHCO_3$, dried over $MgSO_4$ and concentrated under reduced pressure. The residue was purified by flash chromatography on silica gel (6 Et_2O/ 4 pentane) affording the pure compound **26** as a colorless oil (41.1 mg, 0.098 mmol, 95% yield and 95/5 er [determined by HPLC, Chiralcel AD-H, hexane/ isopropanol = 90/10, 1 mL/min, λ = 220 nm, t (minor) = 37.12 min, t (major) = 49.98 min]).

$[\alpha]_D^{25}$ = -1.8 (c = 1.7, $CHCl_3$); **^{1}H NMR** (400 MHz, $CDCl_3$) δ = 8.06 – 8.01 (m, 1H), 7.82 (d, *J* = 8.4 Hz, 2H), 7.68 – 7.62 (m, 1H), 7.41 – 7.32 (m, 2H), 7.30 (d, *J* = 7.8 Hz, 2H), 3.97 (dqd, *J* = 9.6, 6.9, 3.8 Hz, 1H), 3.86 (dd, *J* = 16.5, 3.8 Hz, 1H), 3.37 (dd, *J* = 16.5, 9.6 Hz, 1H), 3.06 – 2.92 (m, 2H), 2.38 (s, 3H), 2.01 – 1.84 (m, 2H), 1.47 (d, *J* = 6.9 Hz, 3H), 1.07 (t, *J* = 7.5 Hz, 3H); **^{13}C NMR** (101 MHz, $CDCl_3$) δ = 150.6, 146.5, 141.8, 135.1, 133.3, 130.6, 127.0, 125.5, 125.0, 120.1, 113.8, 55.3, 52.4, 30.0, 21.8, 15.6, 13.6, 13.4; **IR** (film) υ_{max}/cm^{-1}: 2971, 1596, 1541; 1452, 1376, 1292, 1170, 1122, 1088, 746, 668; **HRMS** (ESI+): calcd. for $C_{20}H_{25}O_4N_2S_2$ $[M+H]^+$ 421.1250, found 421.1240.

9/ Removal of *para*-Methoxybenzyl group (27)

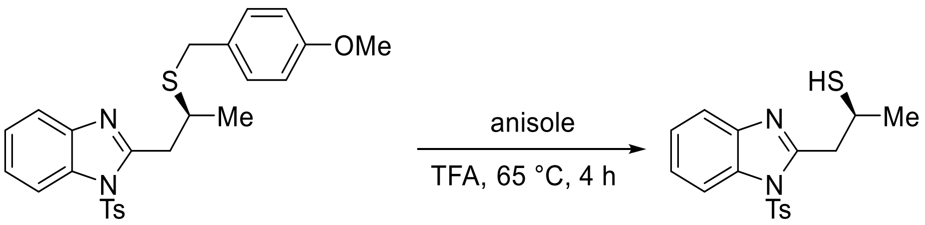

10 **27**

A procedure from the literature[9] was modified as follows. To a solution of **10** (45 mg, 0.095 mmol, 91/9 er) in TFA (621 µL) was added anisole (72 µL, 0.66 mmol) and was stirred at 65°C for 4 hrs. Volatiles were then removed under a stream of N_2 and the resulting crude was dissolved in Et_2O (1.6 mL) and passed through a plug of K_2CO_3. The resulting solution was loaded directly onto silica gel (7 Petrol/ 3 EtOAc) to afford pure **27** as a clear oil (16.1 mg, 0.048 mmol, 50% yield, 91/9 er [determined by HPLC, Chiralcel AD-H, hexane/ isopropanol = 95/5, 1 mL/min, λ = 220 nm, t (minor) = 19.00 min, t (major) = 22.53 min]).

$[\alpha]_D^{25}$ = +3.9 (c = 1.0, $CHCl_3$); **^{1}H NMR** (400 MHz, $CDCl_3$) δ 8.03 – 7.98 (m, 1H), 7.82 – 7.77 (m, 2H), 7.71 – 7.65 (m, 1H), 7.39 – 7.31 (m, 2H), 7.30 – 7.27 (m, 2H), 3.79 – 3.68 (m, 1H), 3.51 – 3.37 (m, 2H), 2.38 (s, 3H), 2.04 (dd, *J* = 6.5, 0.5 Hz, 1H), 1.47 (dd, *J* = 6.5, 0.5 Hz, 3H); **^{13}C NMR** (101 MHz, $CDCl_3$) δ 152.6, 146.2, 142.1, 135.5, 133.1, 130.4, 126.9, 125.2, 124.9, 120.2, 113.8, 41.5, 33.5, 24.7, 21.8; **IR** (film) υ_{max}/cm^{-1}: 2968, 2923, 1452, 1378, 1254, 1232, 1170, 1089, 1048, 746, 668; **HRMS** (APCI+): calcd. for $C_{17}H_{19}O_2N_2S_2$ $[M+H]^+$ 347.0883, found 347.0880.

[9] Y. Liu, B. Sun, B. Wang, M. Wakem, and L. Deng, *J. Am. Chem. Soc.*, 2009, **131**, 418.

10/ Introduction of a 4-Me group (29)

(*E*)-4-methyl-2-(prop-1-en-1-yl)-1-tosyl-1*H*-benzo[*d*]imidazole (**28**)

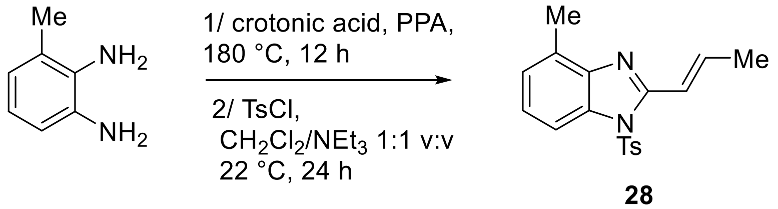

To a mixture of crotonic acid (2.68 g, 30.0 mmol) in polyphosphoric acid (12 g) was added 3-methylbenzene-1,2-diamine (3.66 g, 30.0 mmol). The reaction was heated at 180 °C for 12 h. The mixture was cooled down to 22 °C and poured carefully onto a saturated solution of $NaHCO_3$. Solid $NaHCO_3$ was added until neutral pH was obtained. The solution was diluted with EtOAc and water then stirred vigorously for 10 min. The aqueous phase was extracted twice with EtOAc and the combined organic phase were washed with brine, dried over $MgSO_4$ and concentrated under vacuum affording 4.06 g of crude light brown solid.
The crude residue (2.49 g, 14.5 mmol) was dissolved in CH_2Cl_2 (35.0 mL), NEt_3 (35.0 mL) was added then TsCl (3.01 g, 16.0 mmol). The mixture was stirred for 24h at 22 °C. The solvent was removed under vacuum and the crude was purified by flash chromatography on silica gel (CH_2Cl_2). A subsequent trituration in pentane afforded pure (*E*)-4-methyl-2-(prop-1-en-1-yl)-1-tosyl-1*H*-benzo[*d*]imidazole (3.76 g, 11.5 mmol, 79% yield) as a colourless solid.

Mp : 178°C (from CH_2Cl_2); **^{1}H NMR** (500 MHz, $CDCl_3$) δ 7.88 (d, *J* = 8.2 Hz, 1H), 7.76 (d, *J* = 1.7 Hz, 2H), 7.26 – 7.19 (m, 4H), 7.16 – 7.06 (m, 2H), 2.59 (s, 3H), 2.35 (s, 3H), 2.05 (dd, *J* = 6.8, 1.7 Hz, 3H); **^{13}C NMR** (126 MHz, $CDCl_3$) δ 150.3, 145.7, 141.7, 139.1, 135.5, 132.6, 130.0, 129.9, 126.8, 125.5, 124.6, 118.8, 111.3, 21.6, 19.0, 16.5; **IR** (film) υ_{max}/cm^{-1}: 2980, 1597, 1517, 1445, 1374, 1339, 1203, 1177, 1161, 1092, 965, 830, 812, 777, 667; **HRMS** (ESI+): calcd. for $C_{18}H_{19}O_2N_2S$ $[M+H]^+$ 327.1162, found 327.1160.

4-methyl-2-(2-(propylthio)propyl)-1-tosyl-1*H*-benzo[*d*]imidazole (**29**)

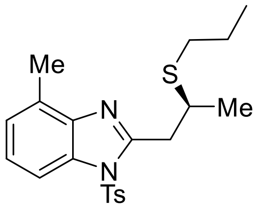

Compound **29** was synthesized according to a modified version of **GPII** from **28** (31.2 mg) using catalyst **G**, 3 equivalents of 1-propanethiol (22.8 mg, 0.3 mmol, 28 µL) and stirred at 22 °C in 0.6 mL of THF. Clear viscous oil (7 Petrol/ 3 EtOAc) (24.1 mg, 0.060 mmol, 60% yield, 50/50 er [determined by HPLC, Chiralpak IC, hexane/isopropanol = 99/1, 1 mL/min, λ = 220 nm, t (major) = 16.69 min, t (minor) = 19.91 min])
^{1}H NMR (400 MHz, $CDCl_3$) δ 7.82 (dt, *J* = 8.2, 0.9 Hz, 1H), 7.80 – 7.72 (m, 2H), 7.29 – 7.23 (m, 3H), 7.21 (d, *J* = 7.9 Hz, 1H), 7.12 (dt, *J* = 7.5, 1.0 Hz, 1H), 3.65 – 3.47 (m, 2H), 3.34 – 3.22 (m, 1H), 2.67 – 2.48 (s + m, 5H), 2.37 (s, 3H), 1.63 (h, *J* = 7.2 Hz, 2H), 1.35 (d, *J* = 6.6 Hz, 3H), 0.97 (t, *J* = 7.3 Hz, 3H); **^{13}C NMR** (101 MHz, $CDCl_3$) δ 152.0, 145.9, 141.3, 135.8, 132.9, 130.3, 130.2, 126.9, 125.4, 124.8, 111.3, 38.6, 38.1, 33.0, 23.3, 21.8, 21.4, 16.6, 13.7; **IR** (film) υ_{max}/cm^{-1}: 2962, 2925, 2871, 1374, 1190, 1186, 1103, 1012, 812, 778, 766, 703, 678, 648; **HRMS** (ESI+): calcd. for $C_{21}H_{27}O_2N_2S_2$ $[M+H]^+$ 403.1509, found 403.1506.

11/ ^{1}H and ^{13}C NMR spectra

Spectra for **Bz5**:

^{1}H NMR:

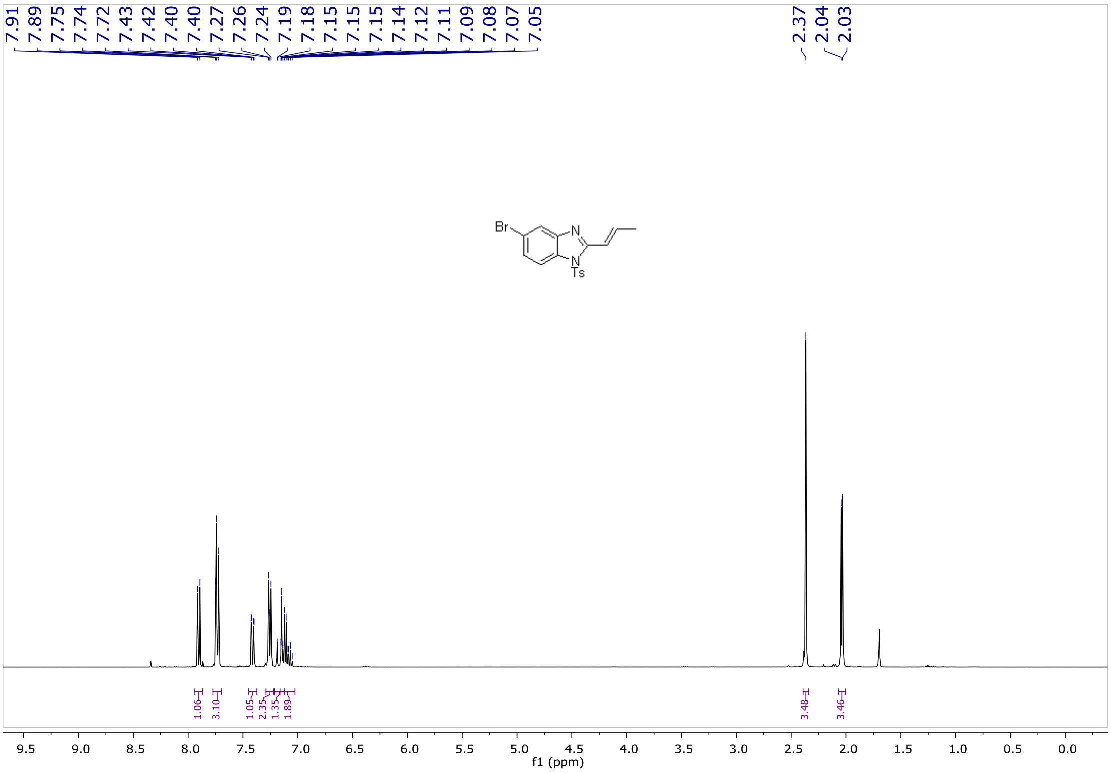

^{13}C NMR:

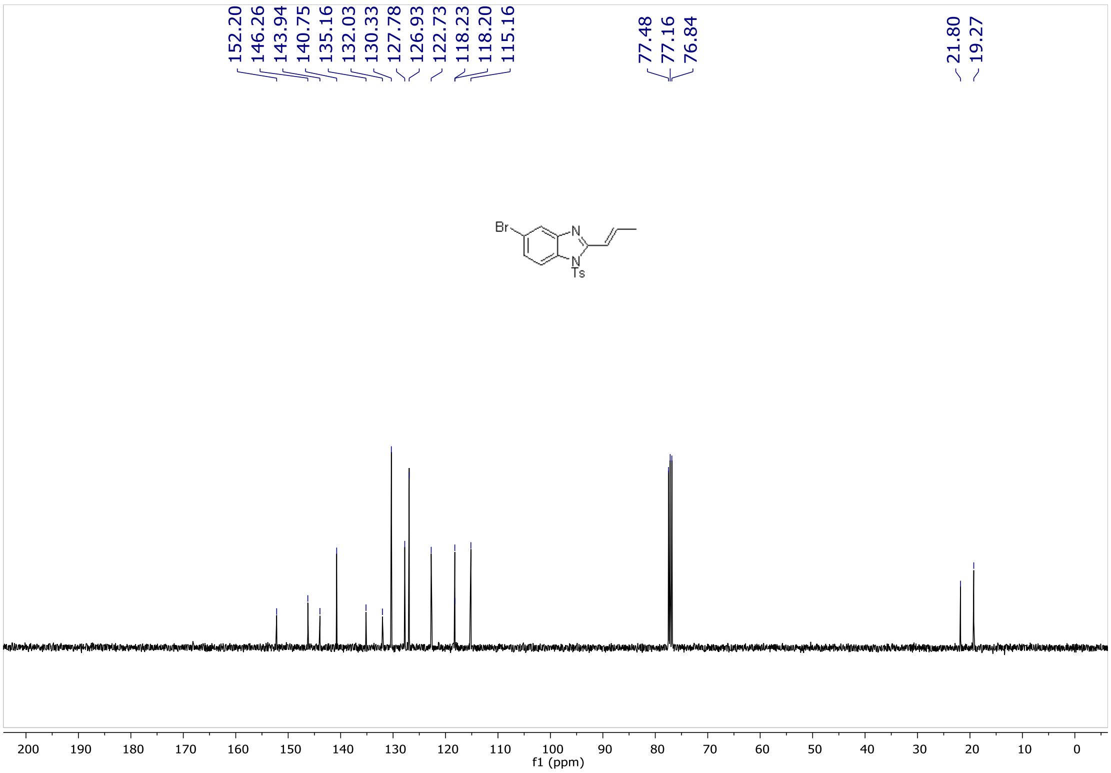

Spectra for **Bz6**:

^{1}H NMR:

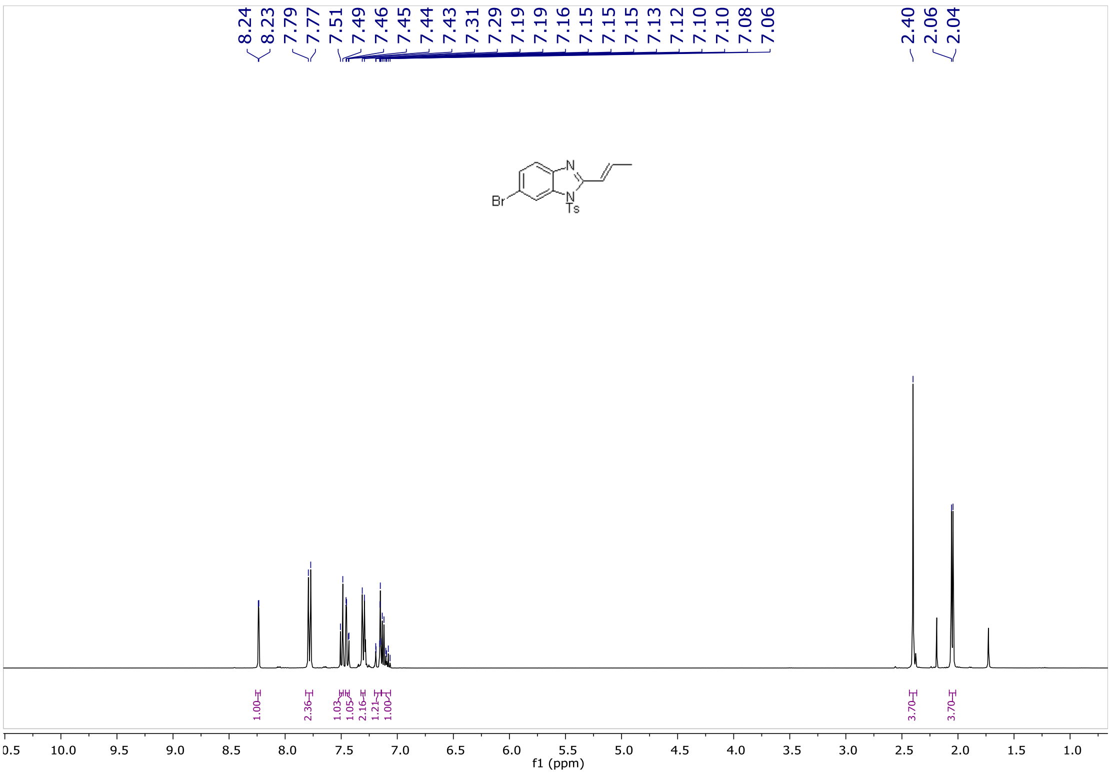

^{13}C NMR:

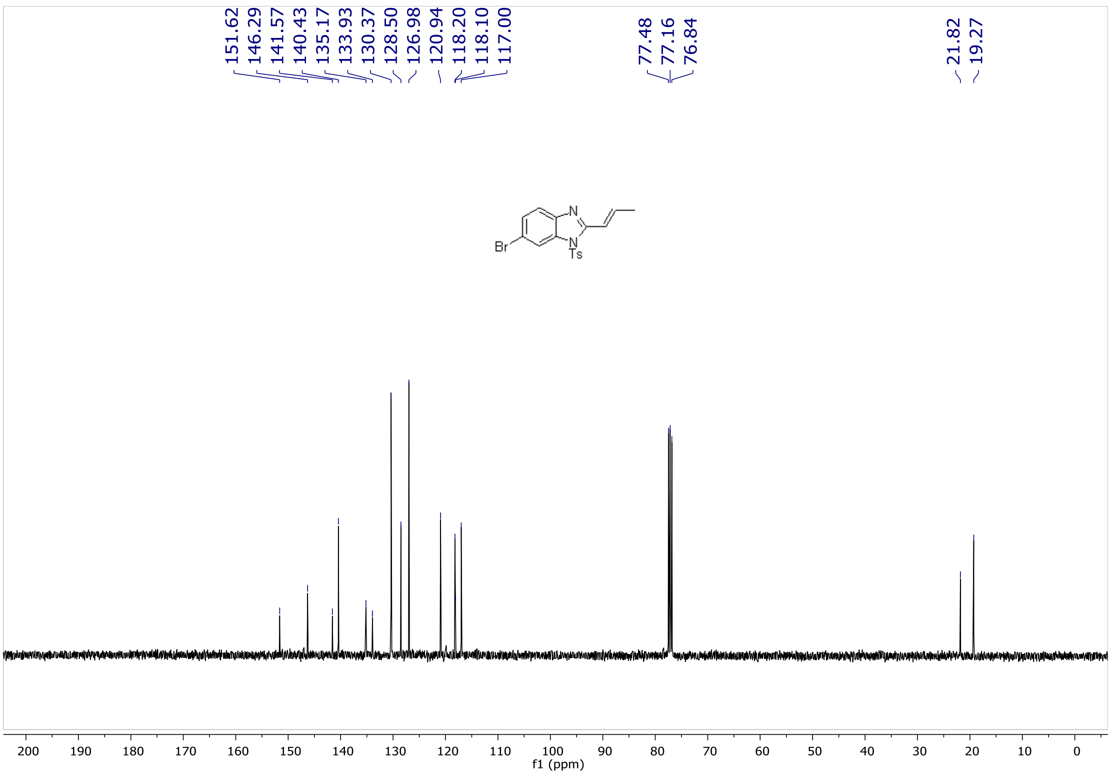

Spectra for **Bz7**:

^{1}H NMR:

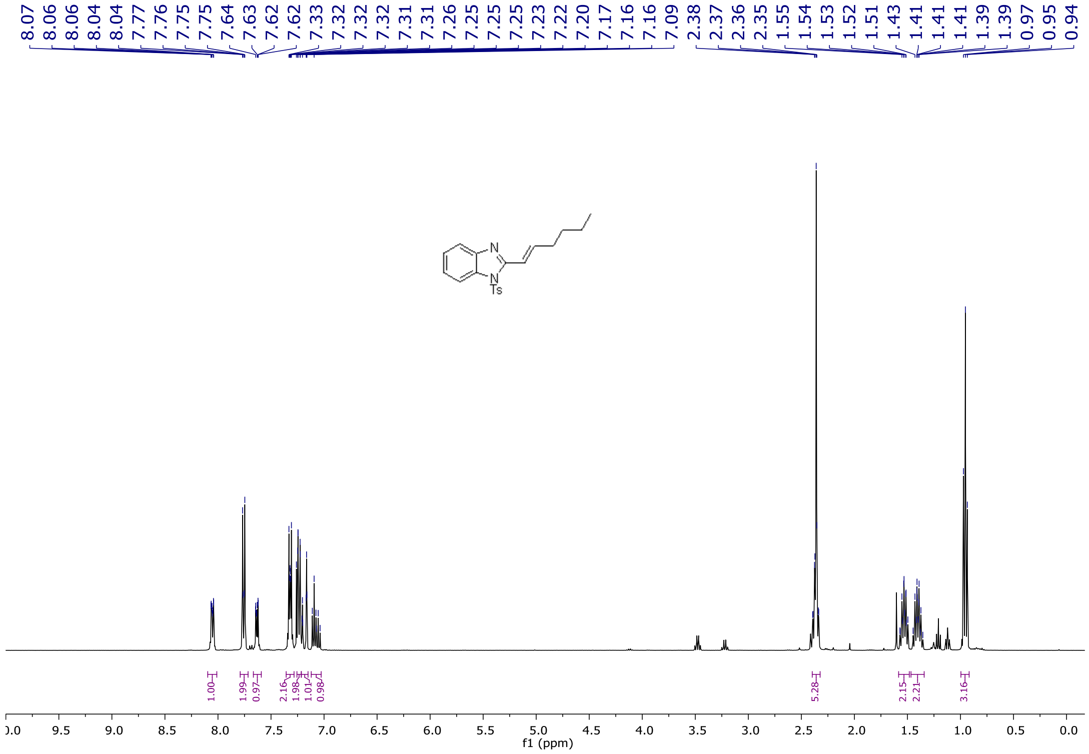

^{13}C NMR:

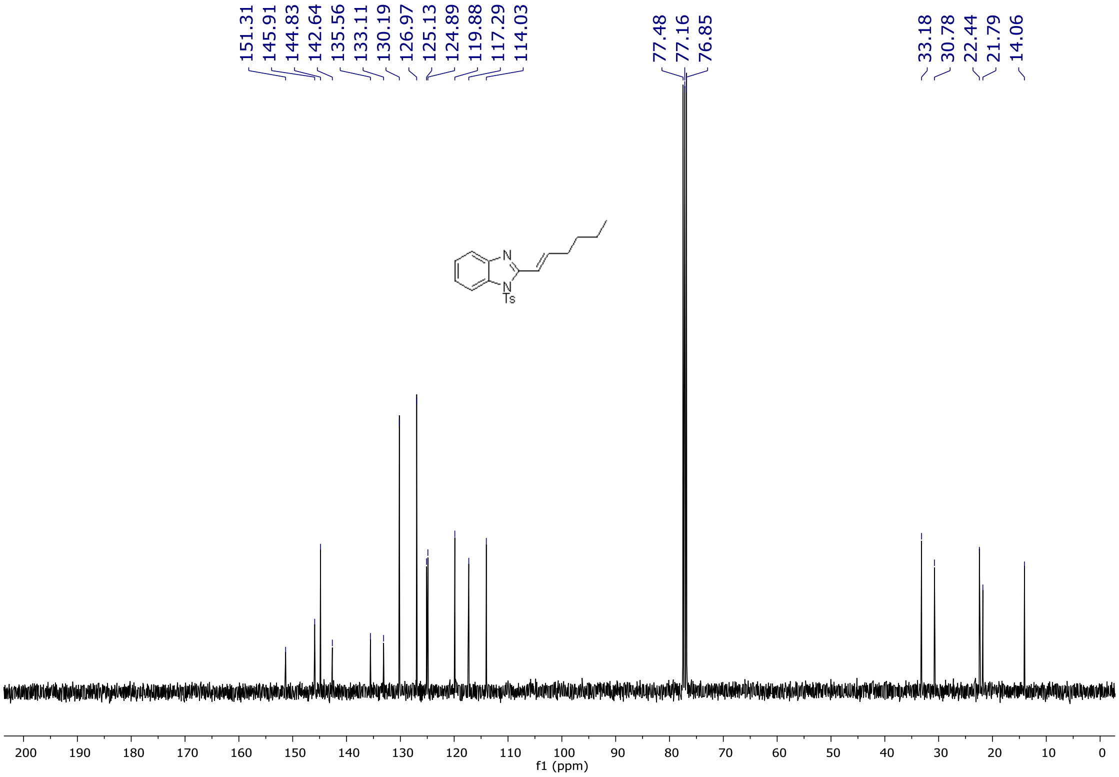

Spectra for **Bz8**:

^{1}H NMR:

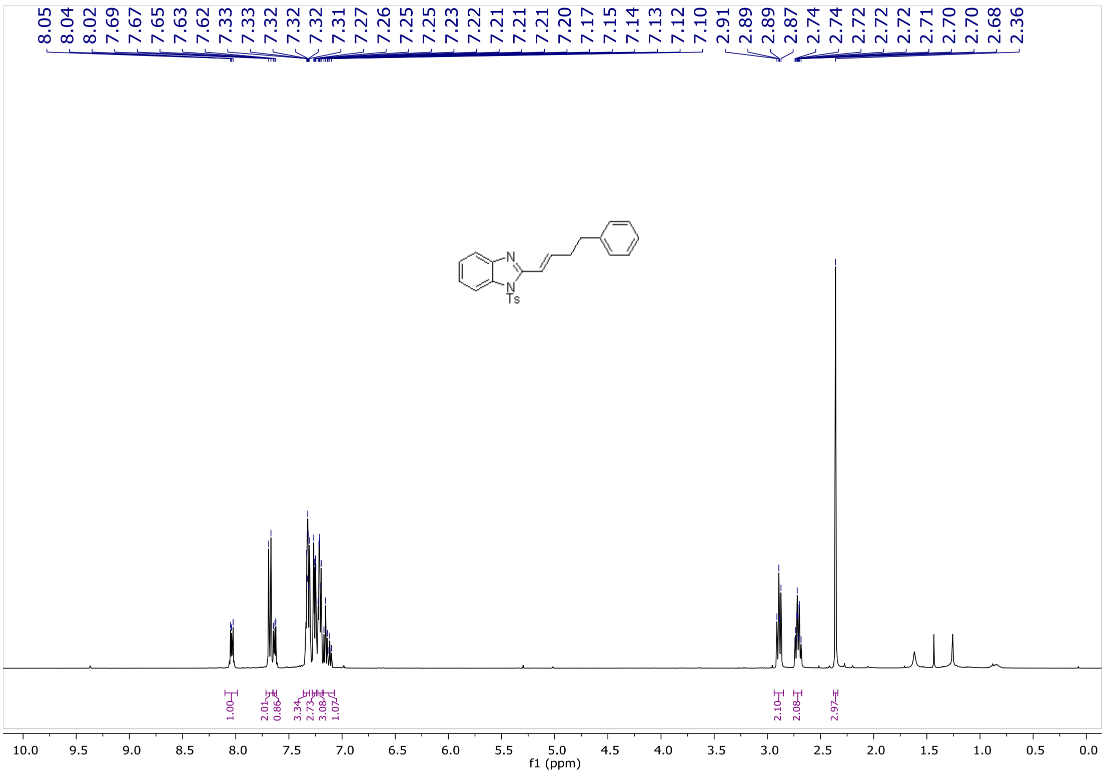

^{13}C NMR:

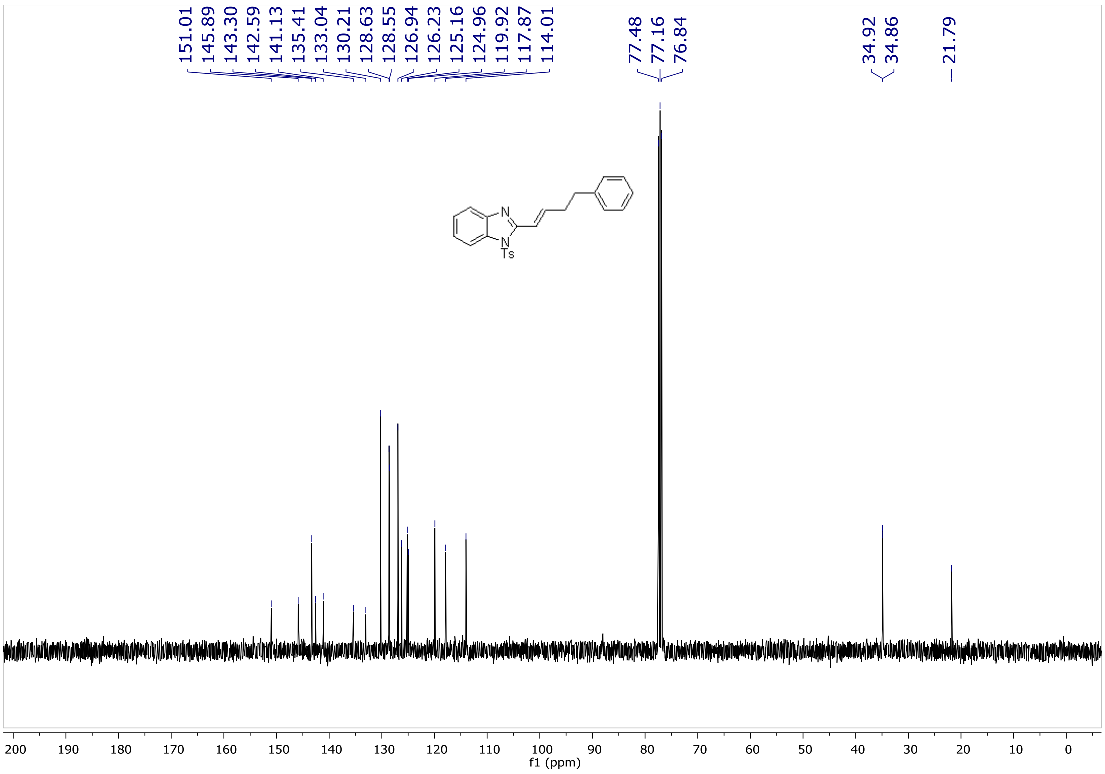

Spectra for **Bz9**:

^{1}H NMR:

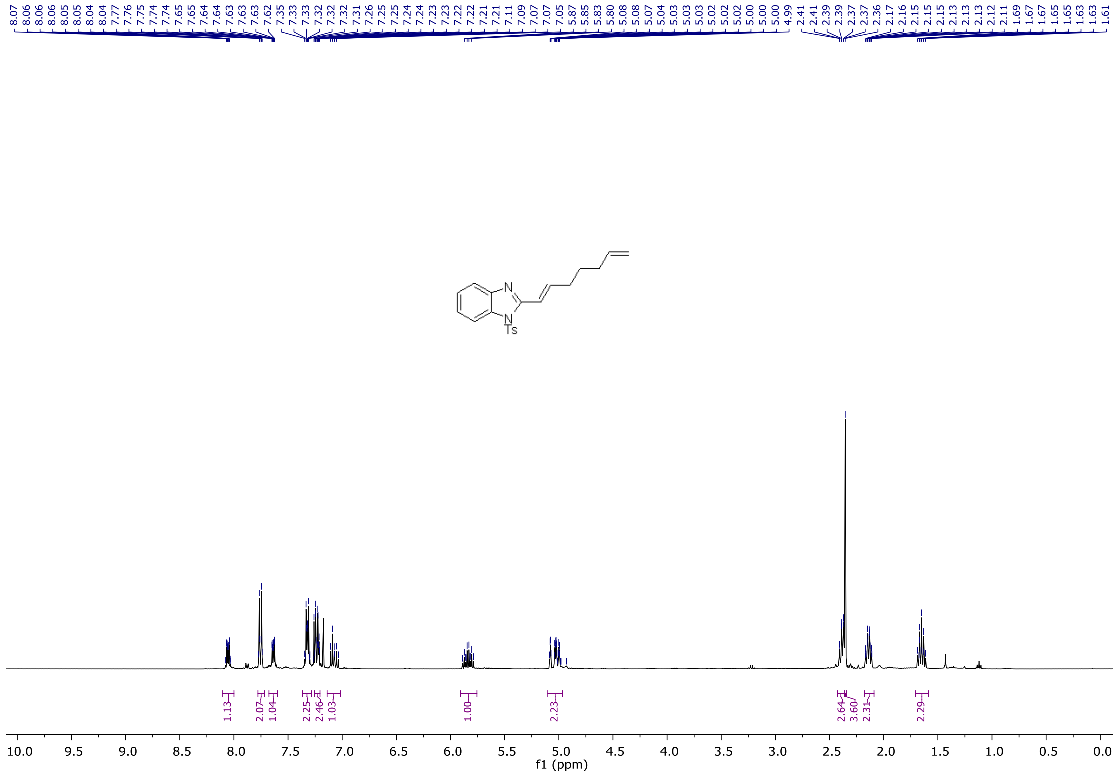

^{13}C NMR:

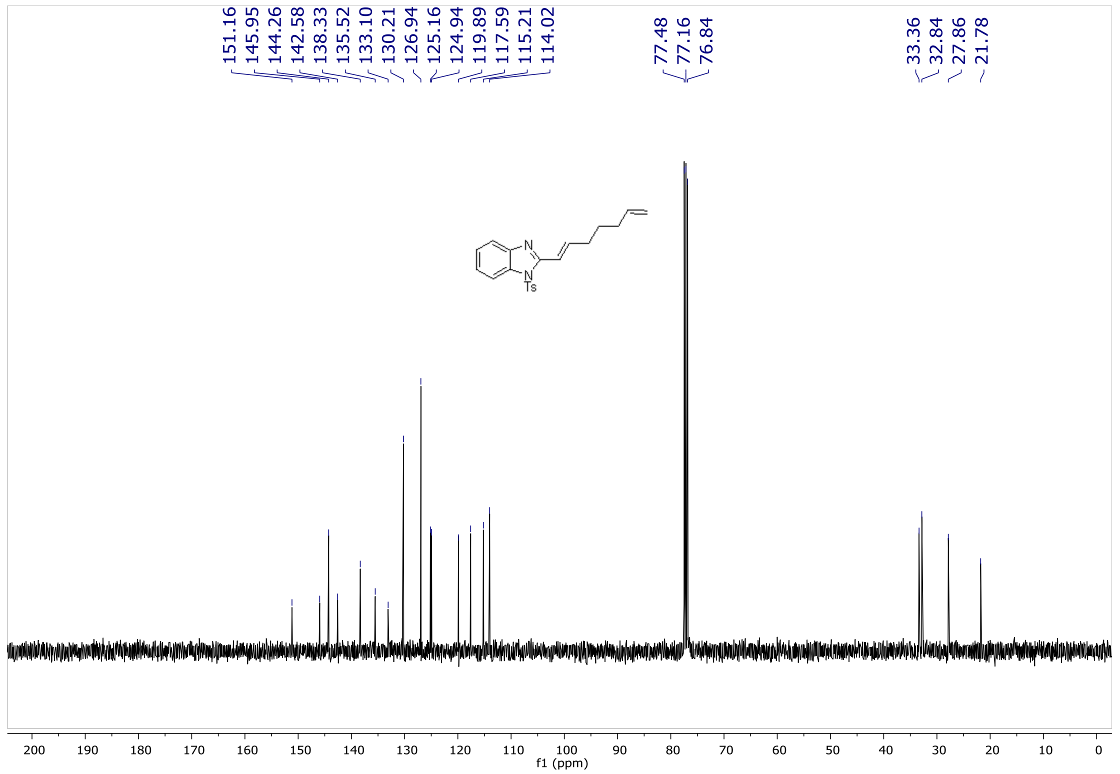

Spectra for **Bz10**:

^{1}H NMR:

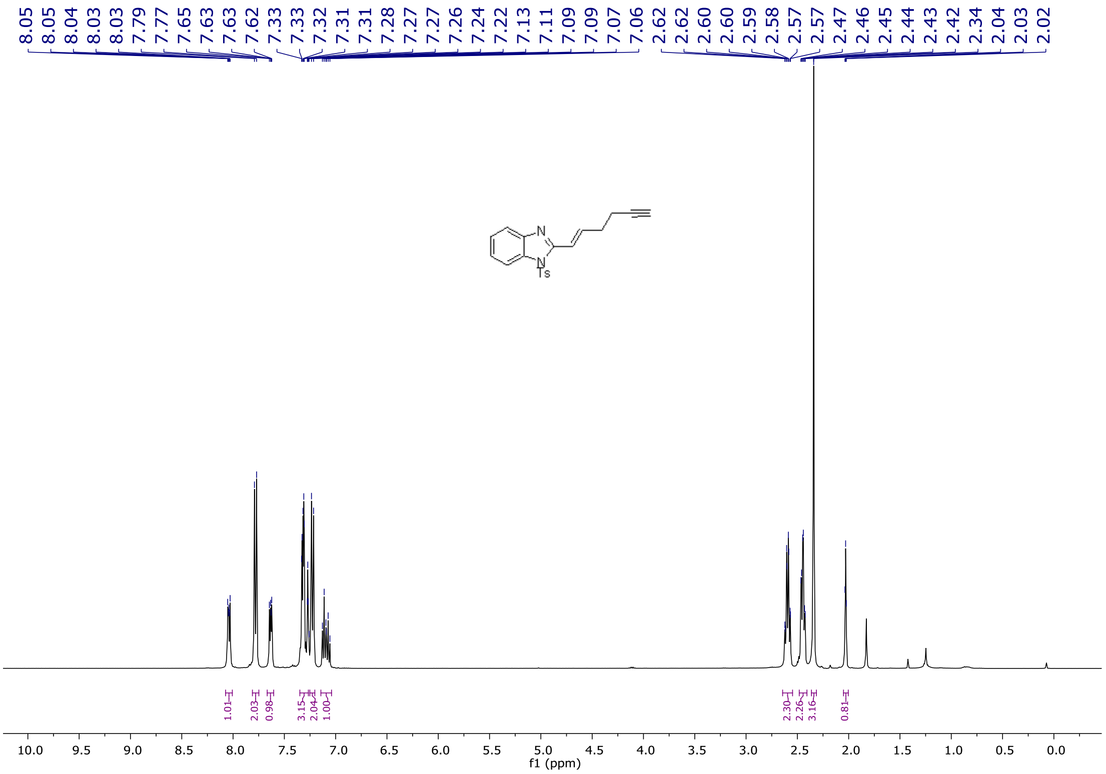

^{13}C NMR:

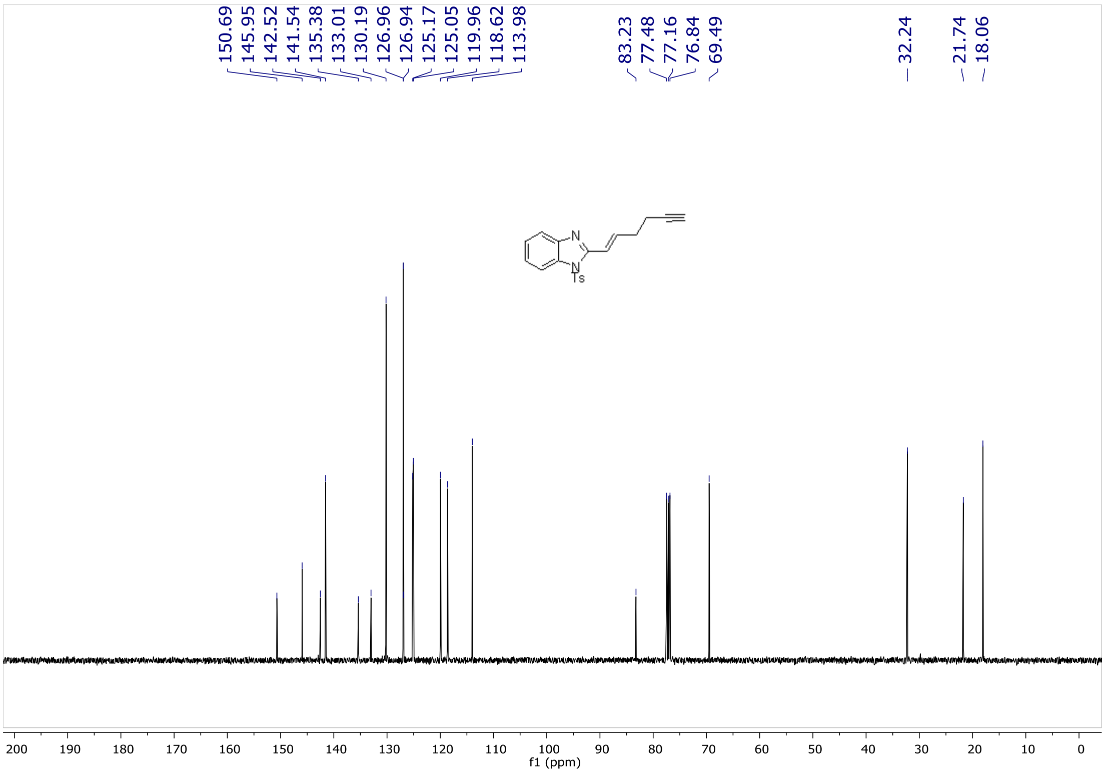

Spectra for **Bz11**:

^{1}H NMR:

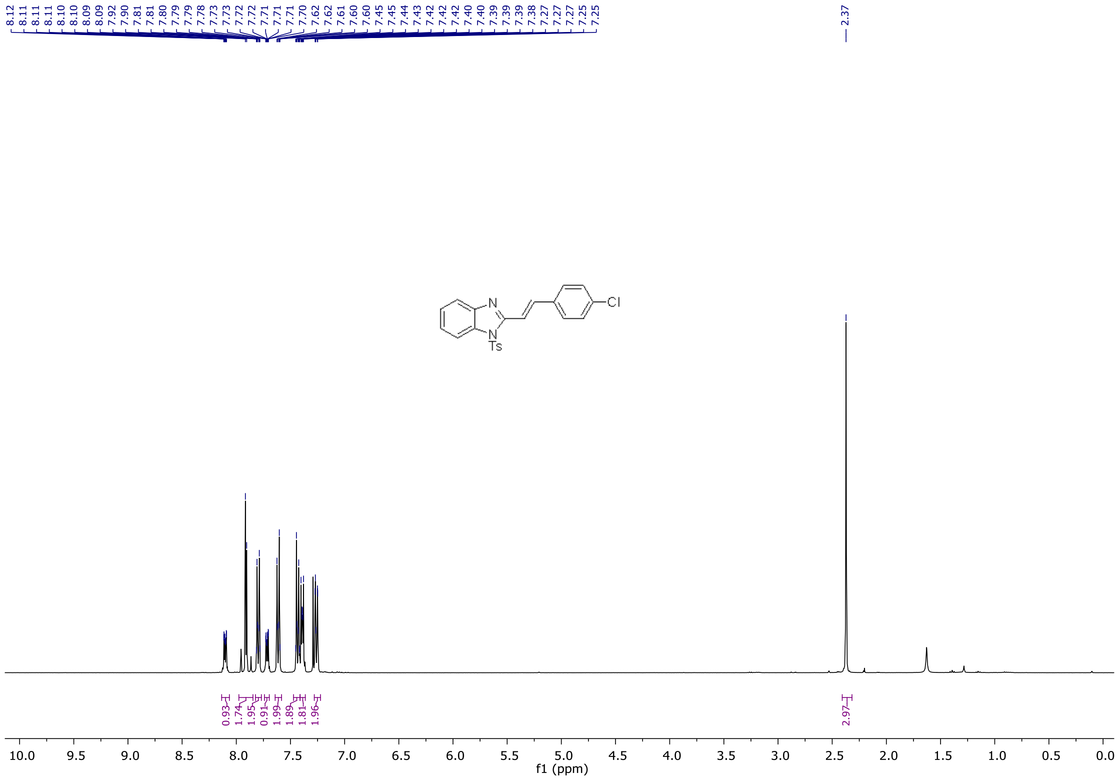

^{13}C NMR:

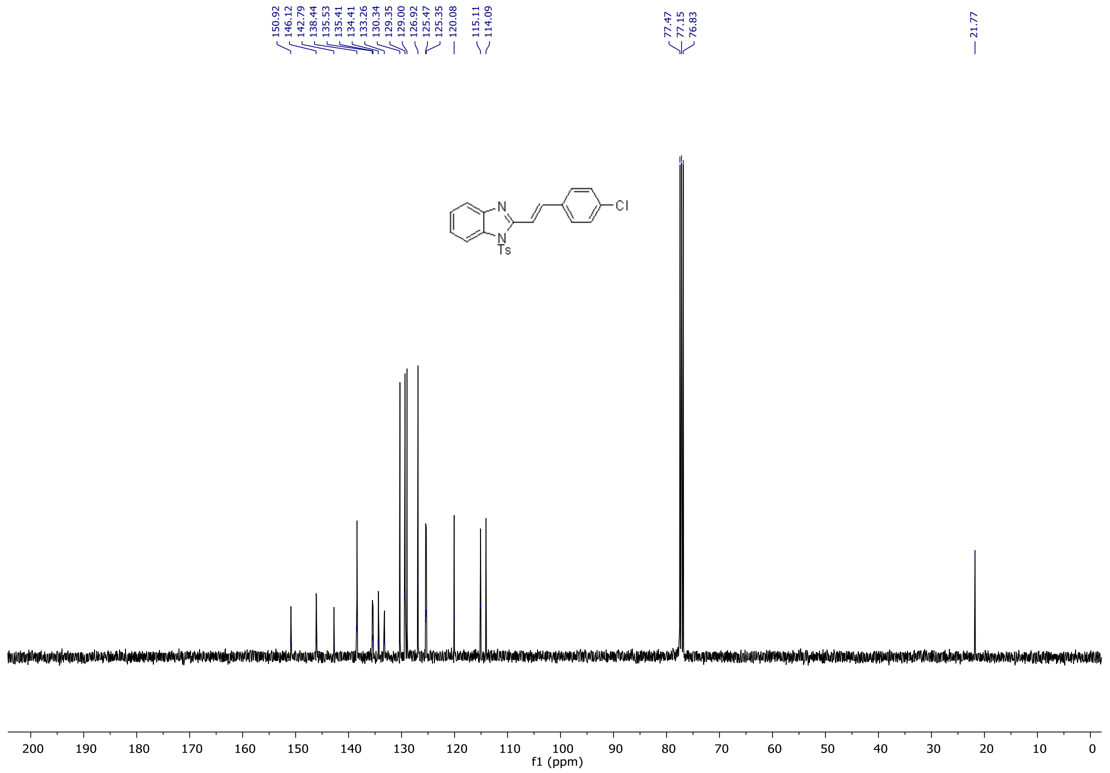

Spectra for **Bz12**:

^{1}H NMR:

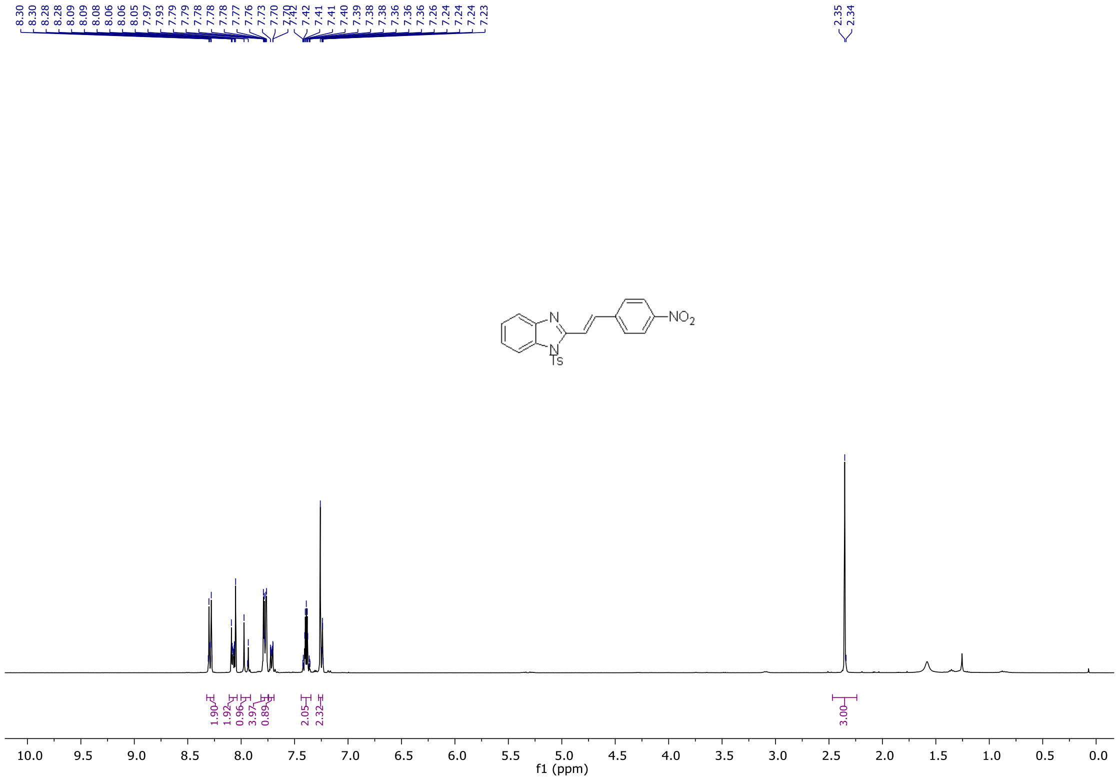

^{13}C NMR:

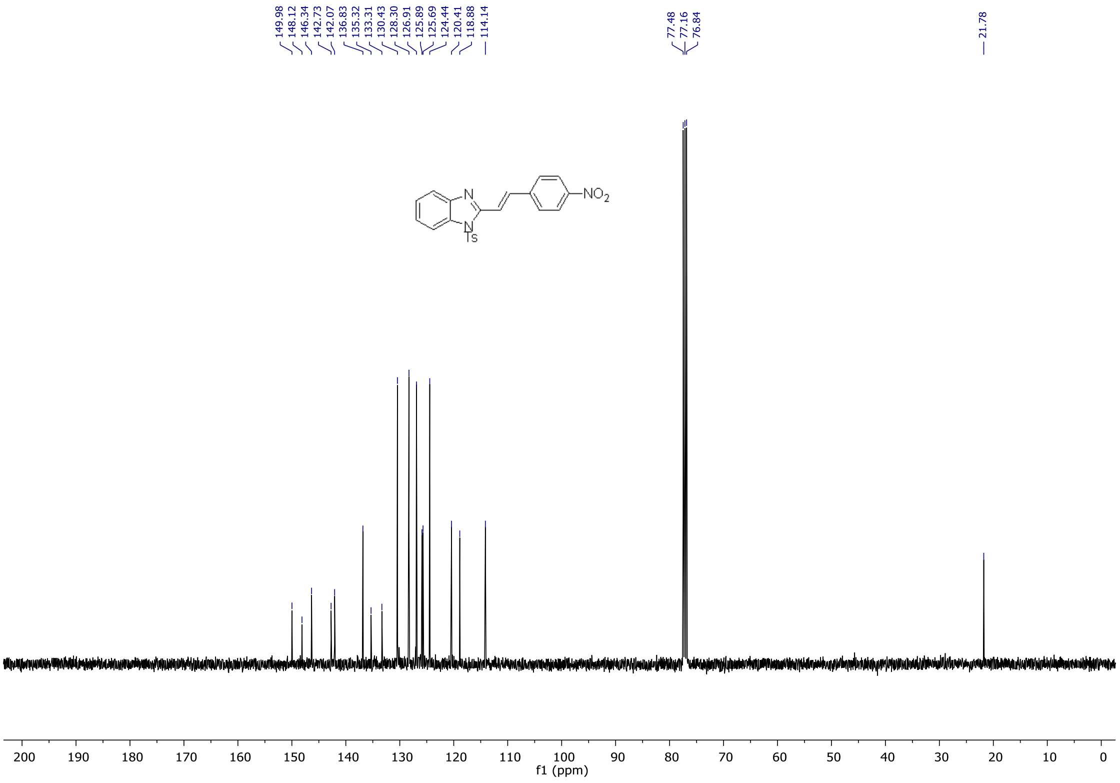

Spectra for **Bz13**:

^{1}H NMR:

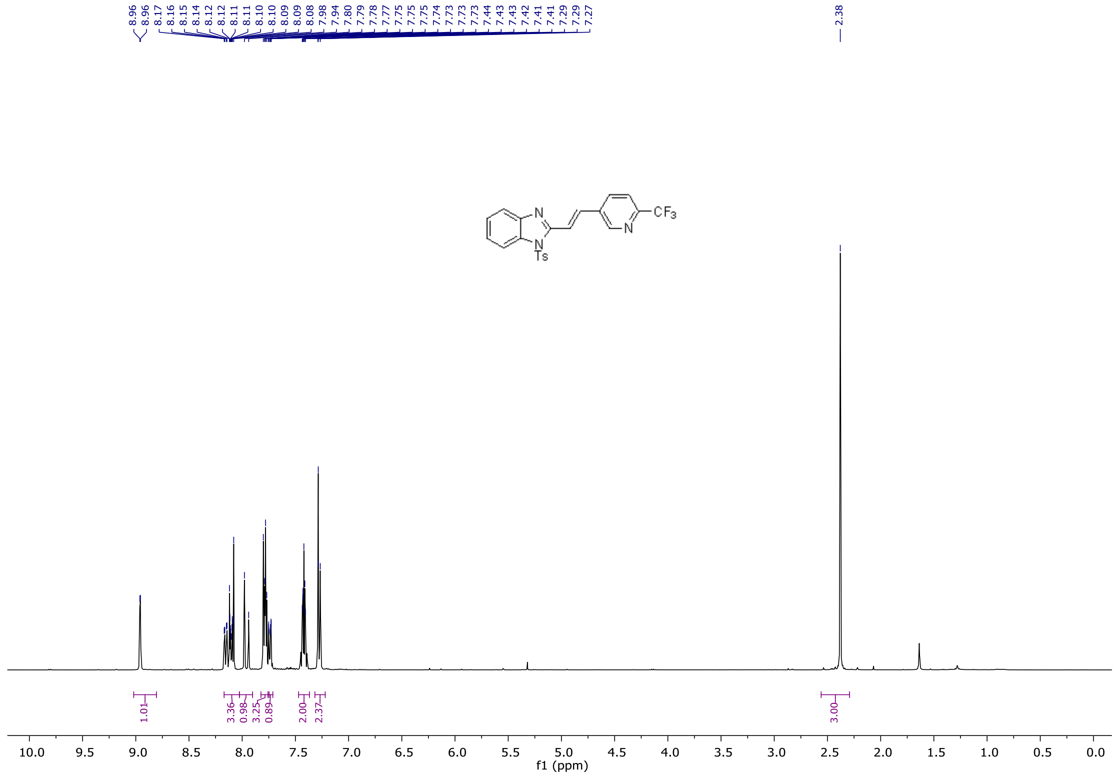

^{13}C NMR:

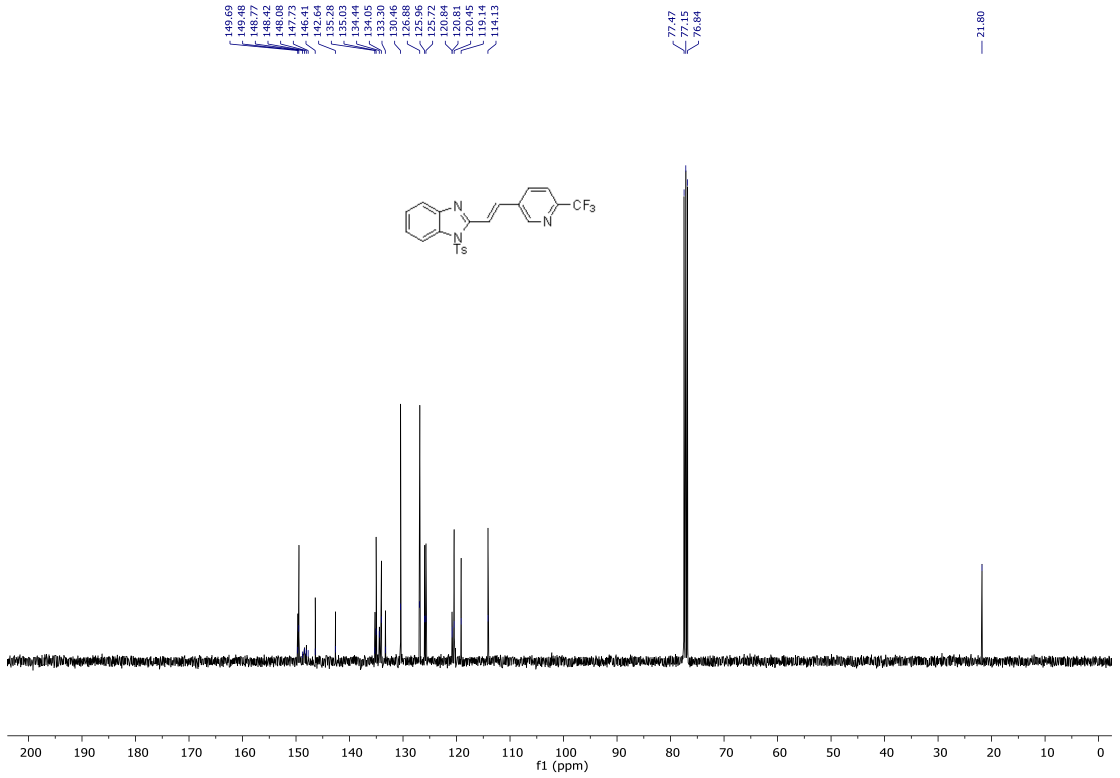

^{19}F NMR:

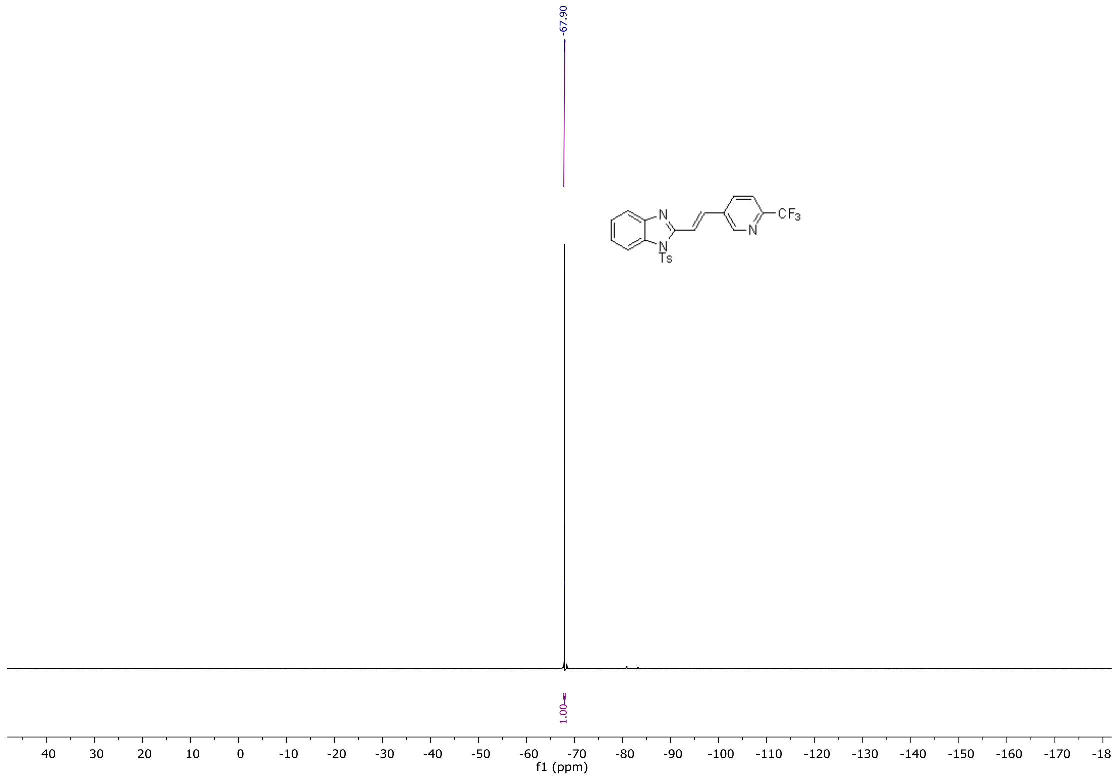

-67.90
CF3
N
N
Ts
N
1.00
40
30
20
10
0
-10
-20
-30
-40
-50
-60
-70
-80
-90
-100
-110
-120
-130
-140
-150
-160
-170
-18
f1 (ppm)

Spectra for **2**:

^{1}H NMR:

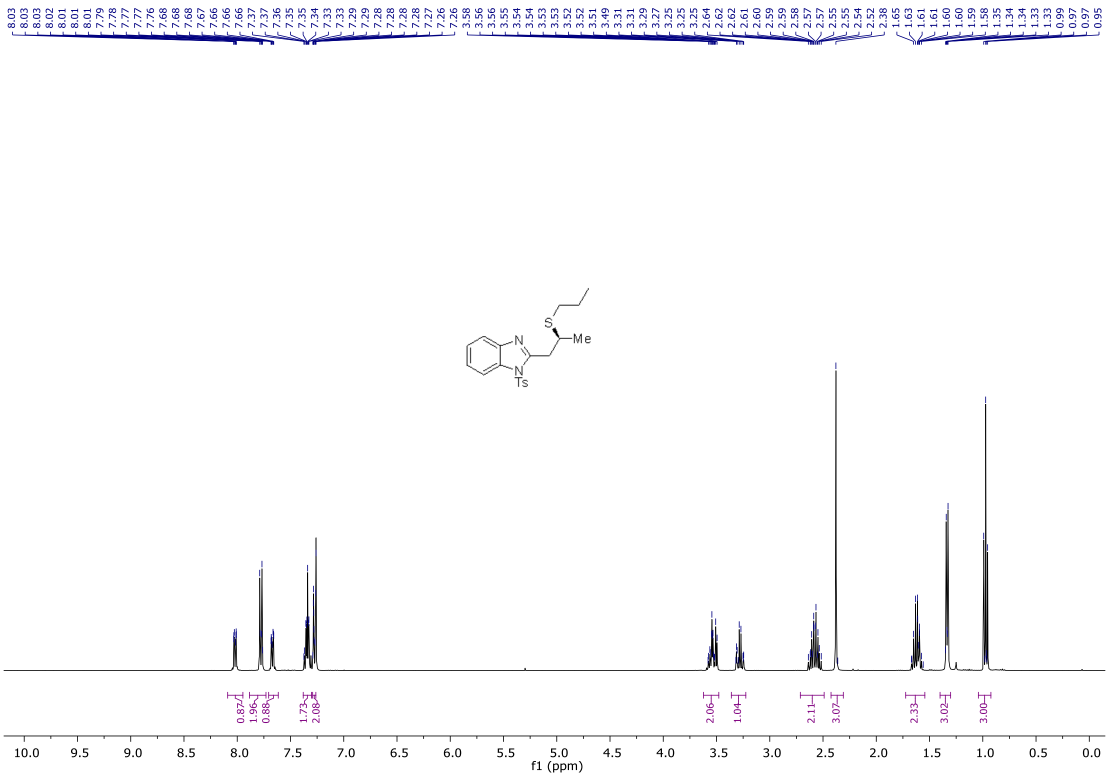

^{13}C NMR:

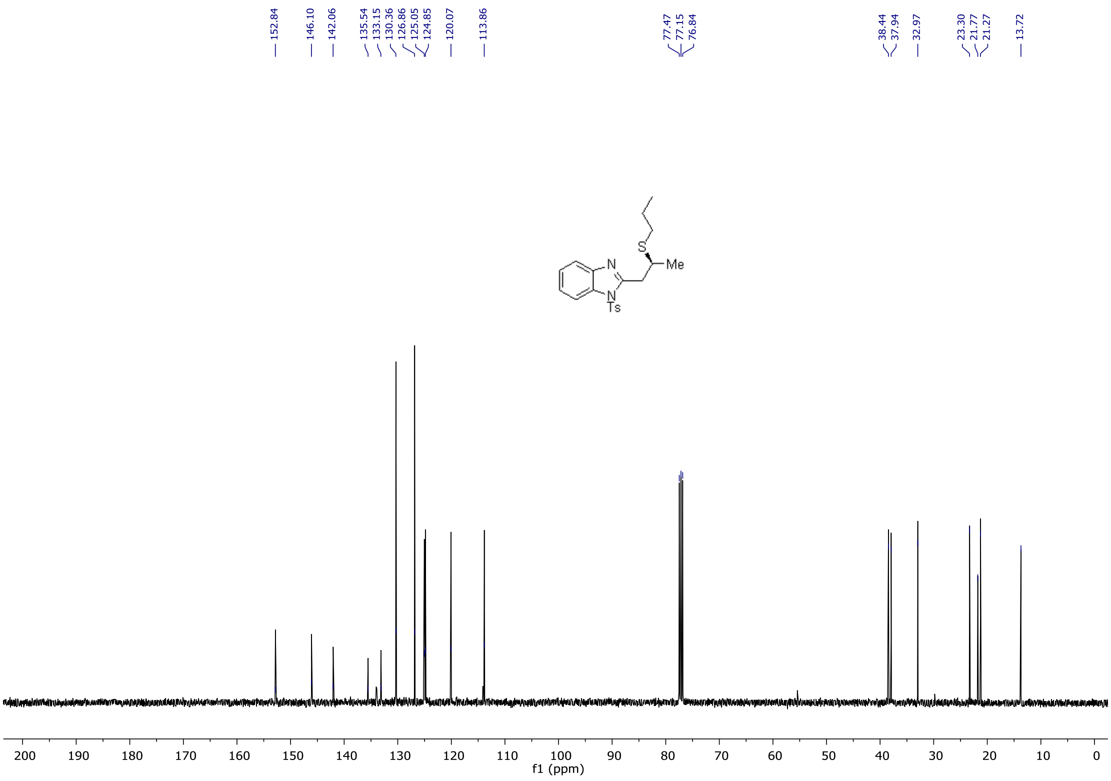

Spectra for **3**:

^{1}H NMR:

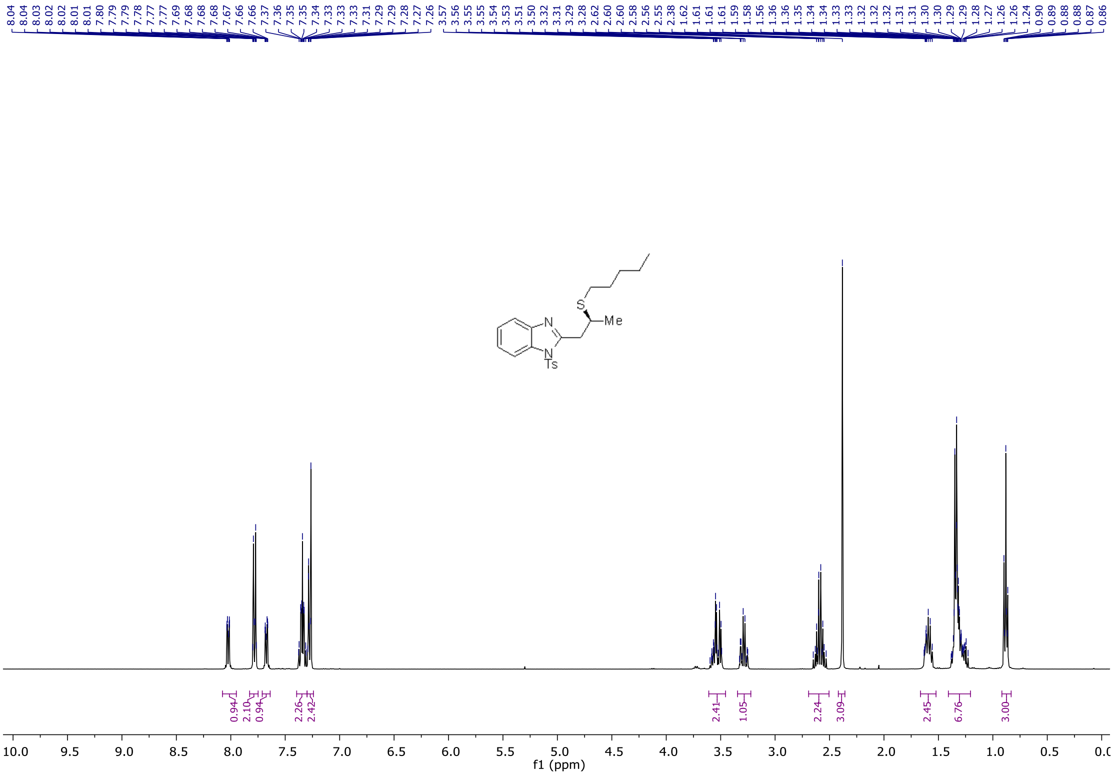

^{13}C NMR:

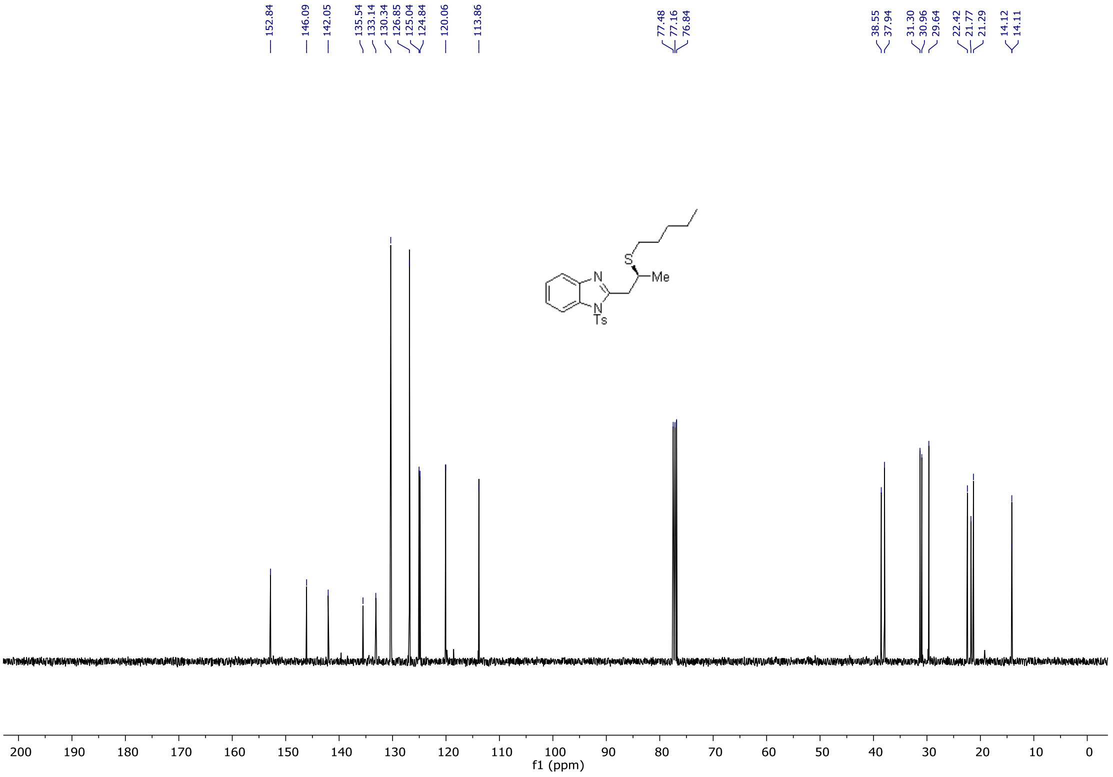

Spectra for **4**:

^{1}H NMR:

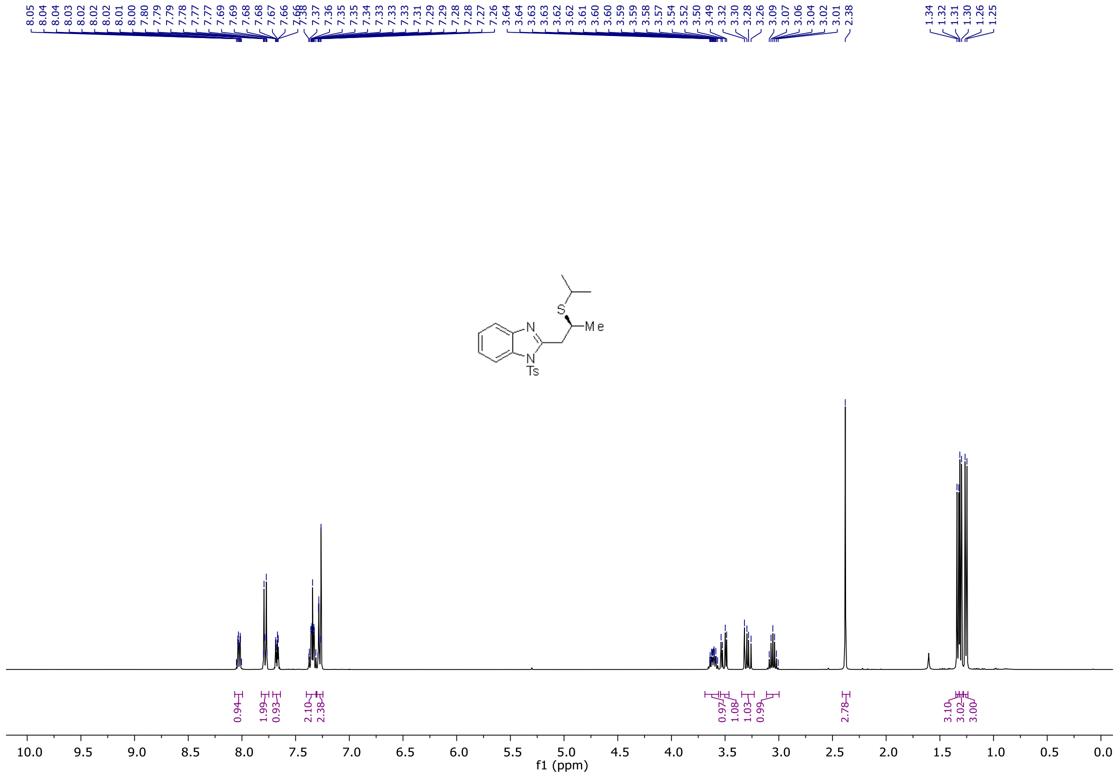

^{13}C NMR:

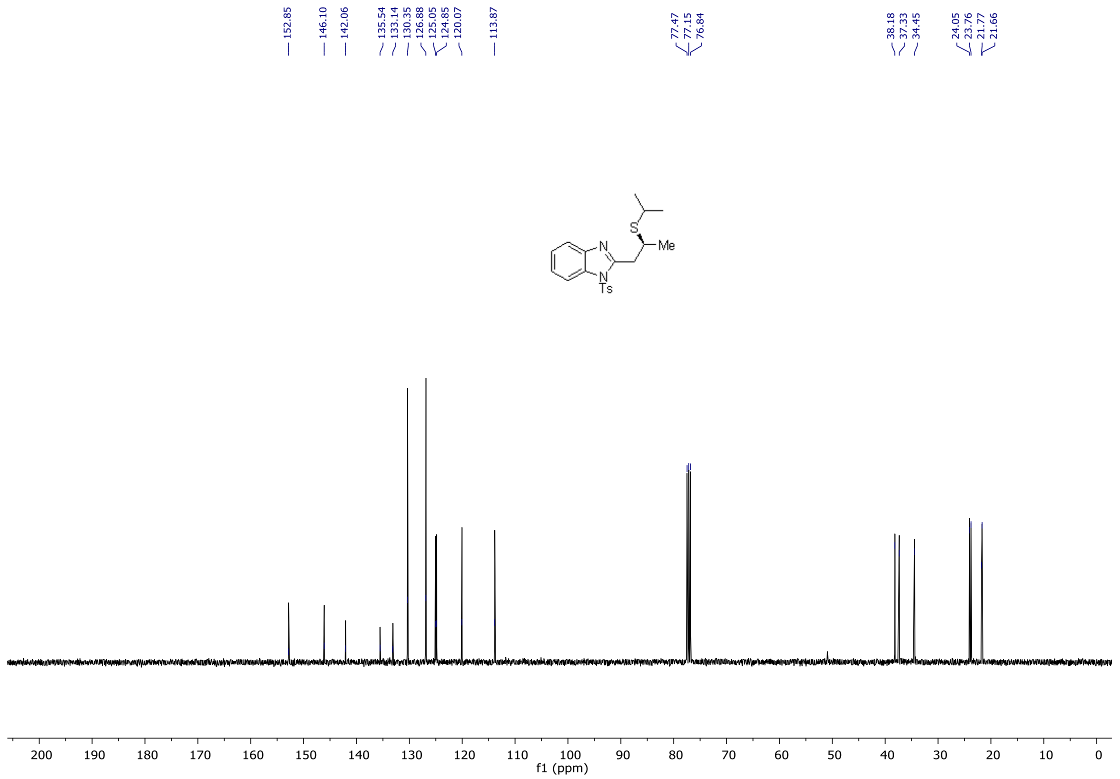

Spectra for **5**:

^{1}H NMR:

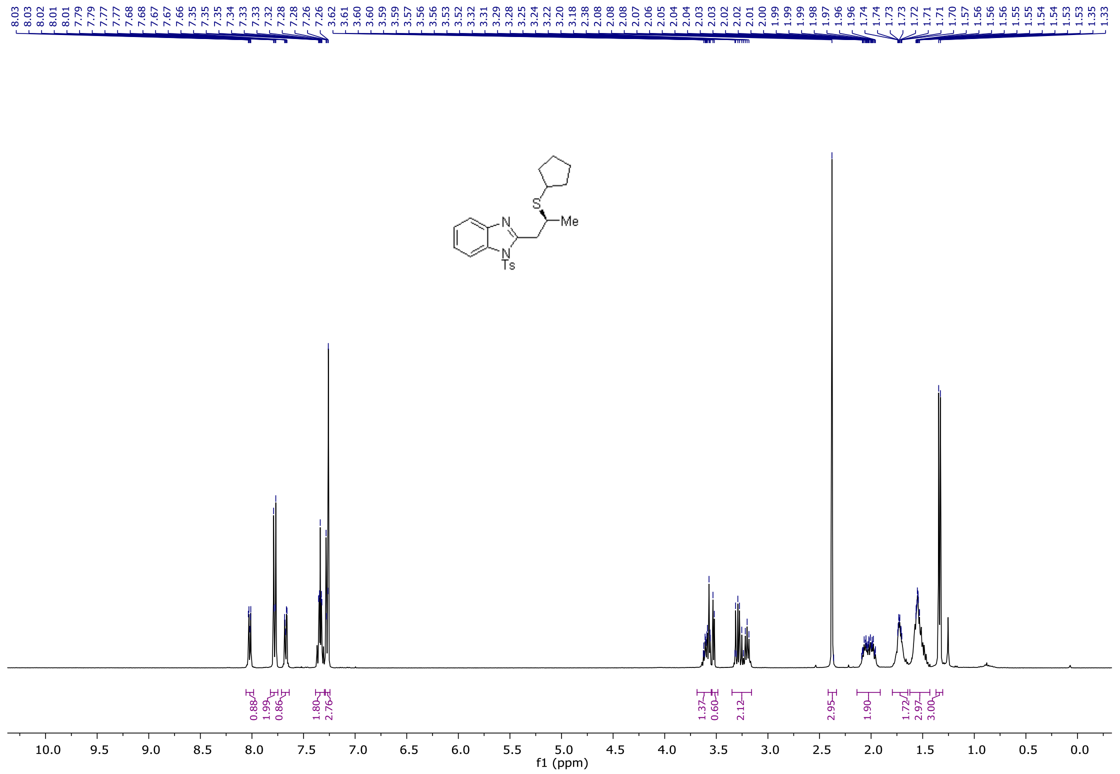

^{13}C NMR:

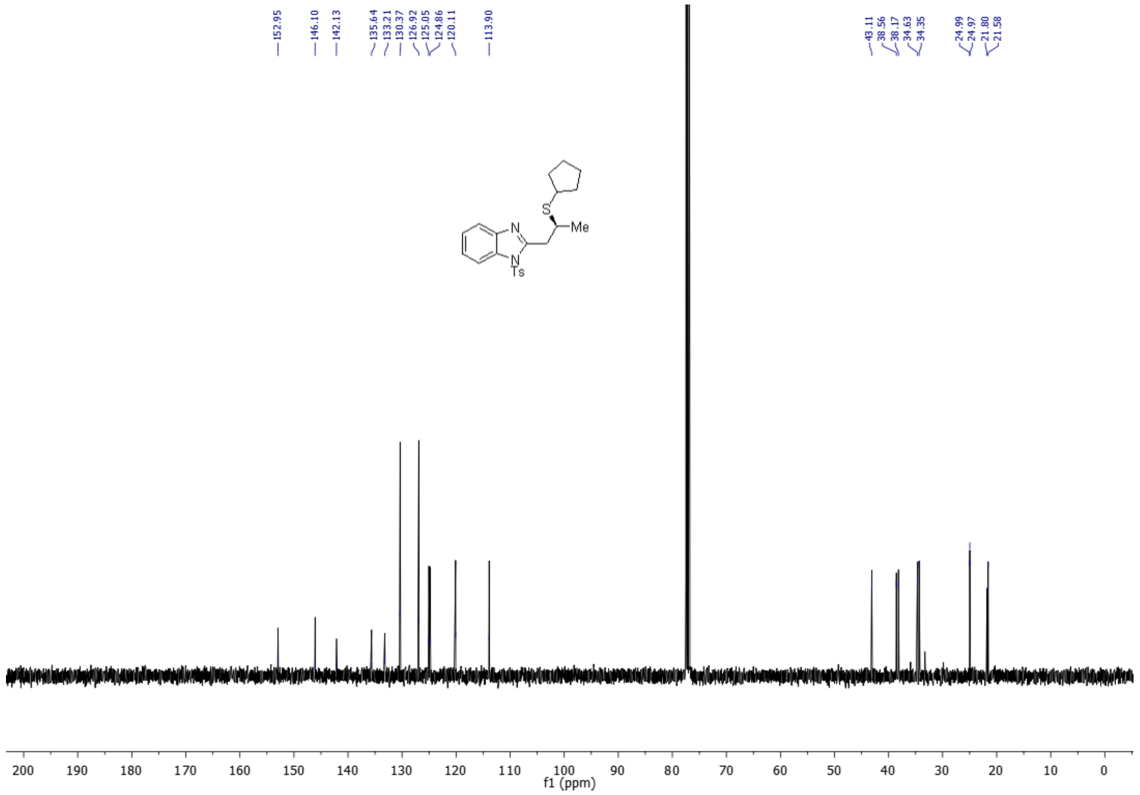

Spectra for **6**:

^{1}H NMR:

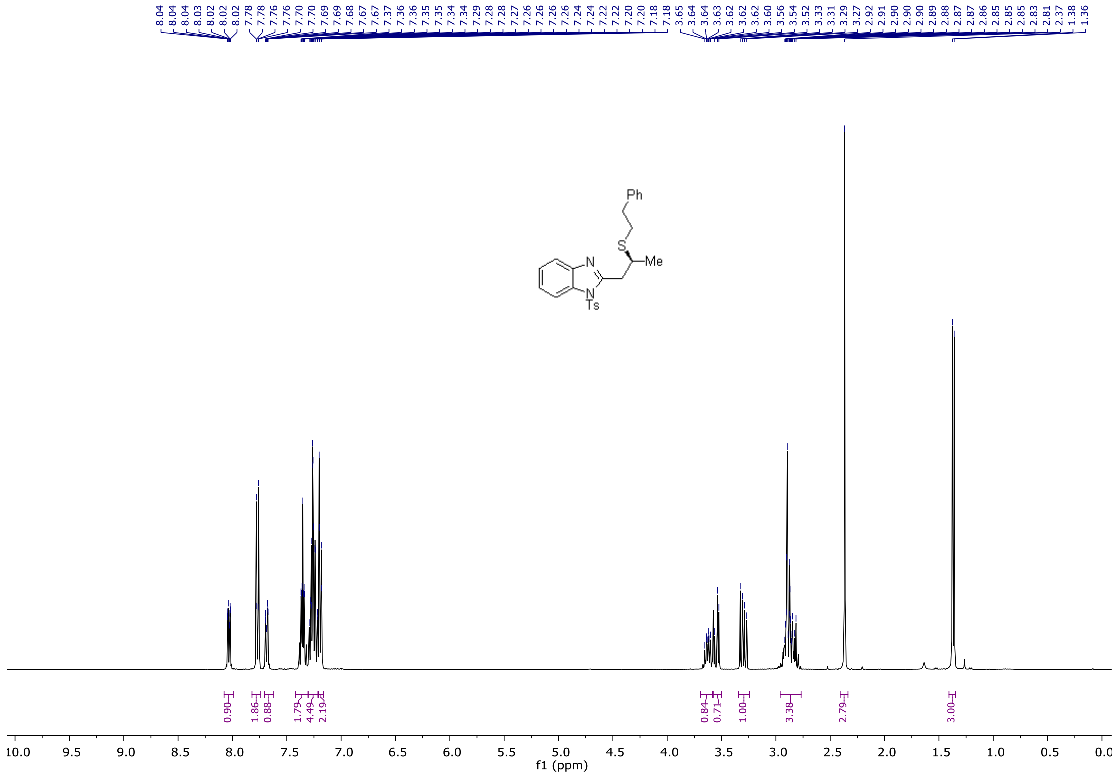

^{13}C NMR:

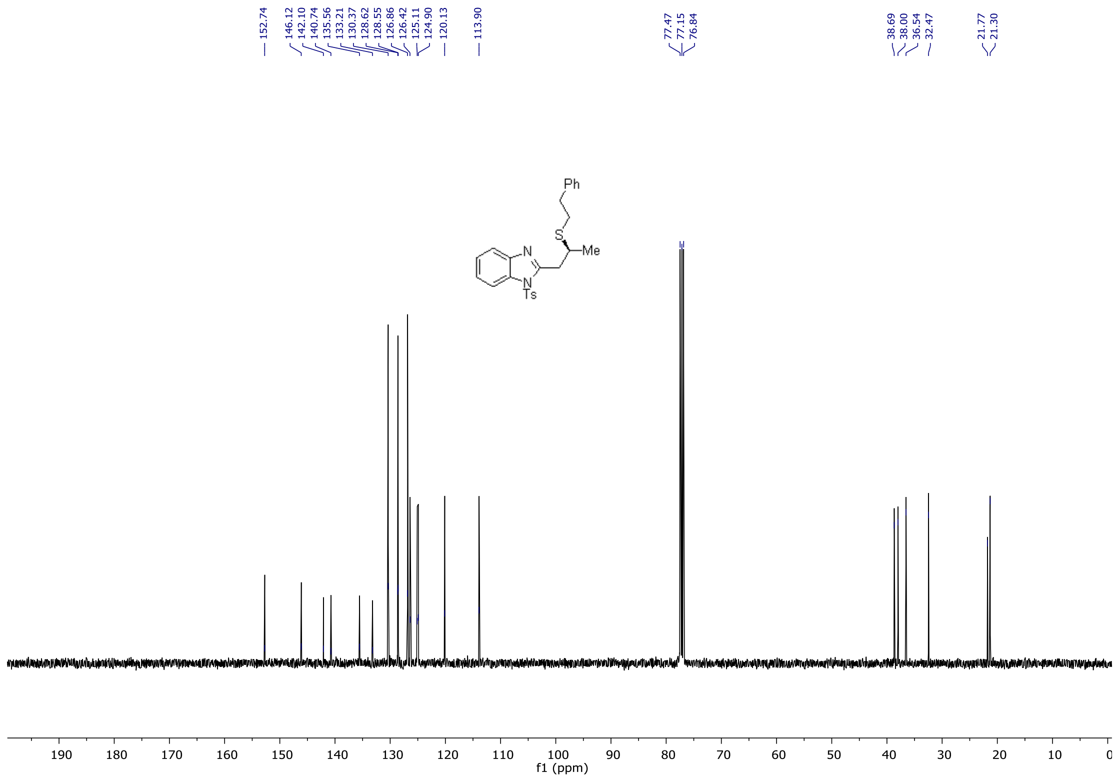

Spectra for **7**:

^{1}H NMR:

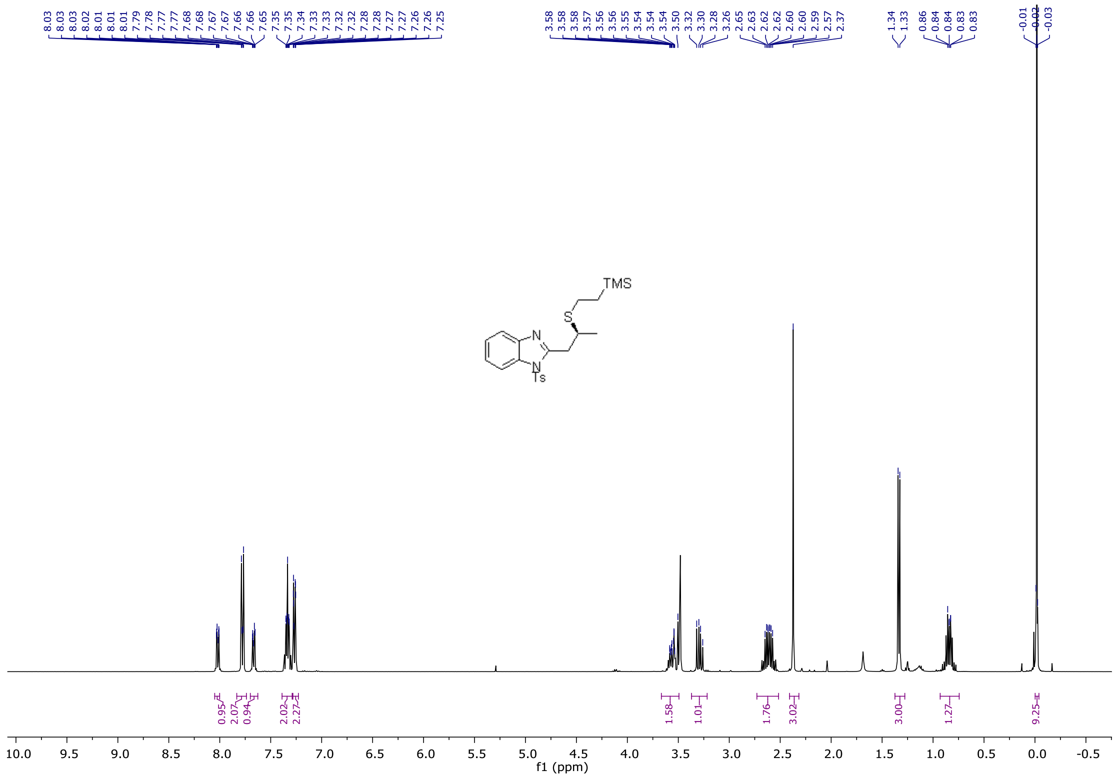

^{13}C NMR:

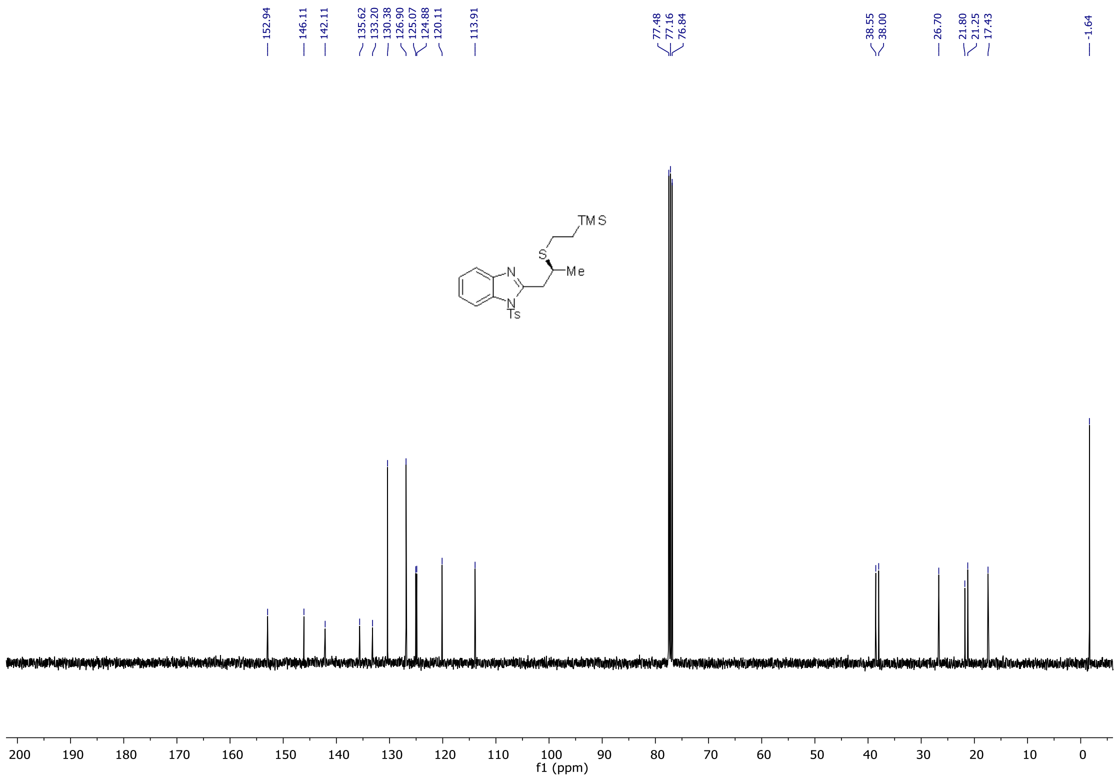

Spectra for **8**:

^{1}H NMR:

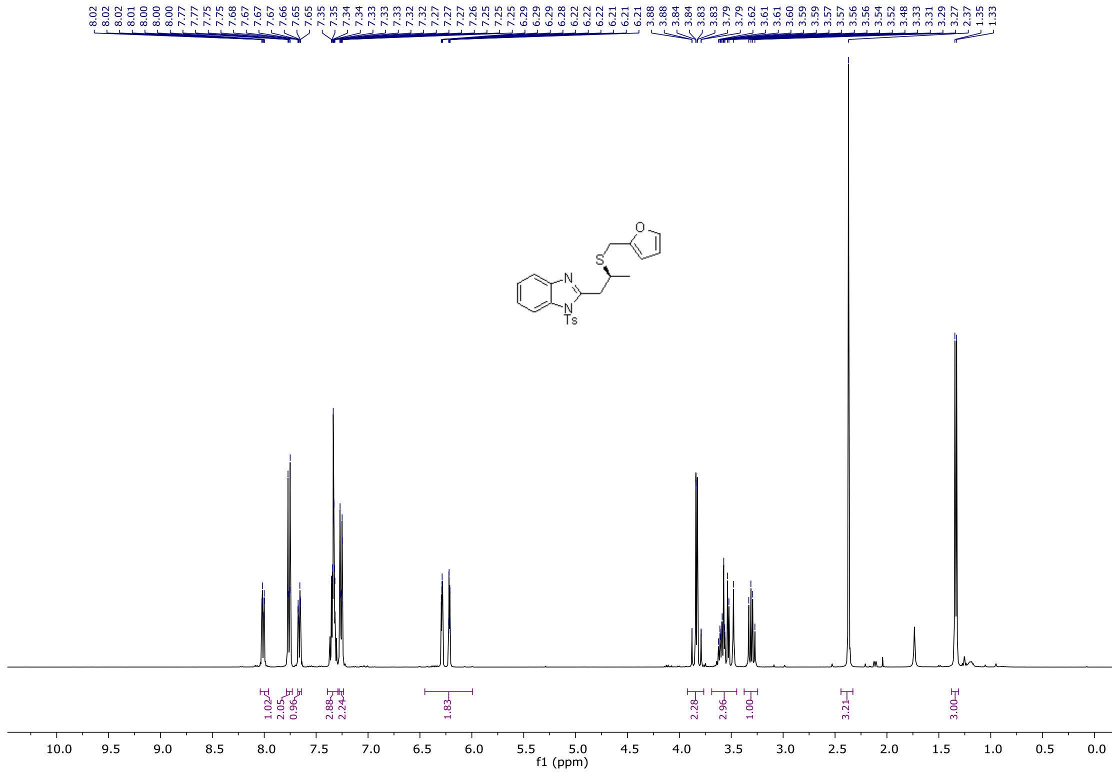

^{13}C NMR:

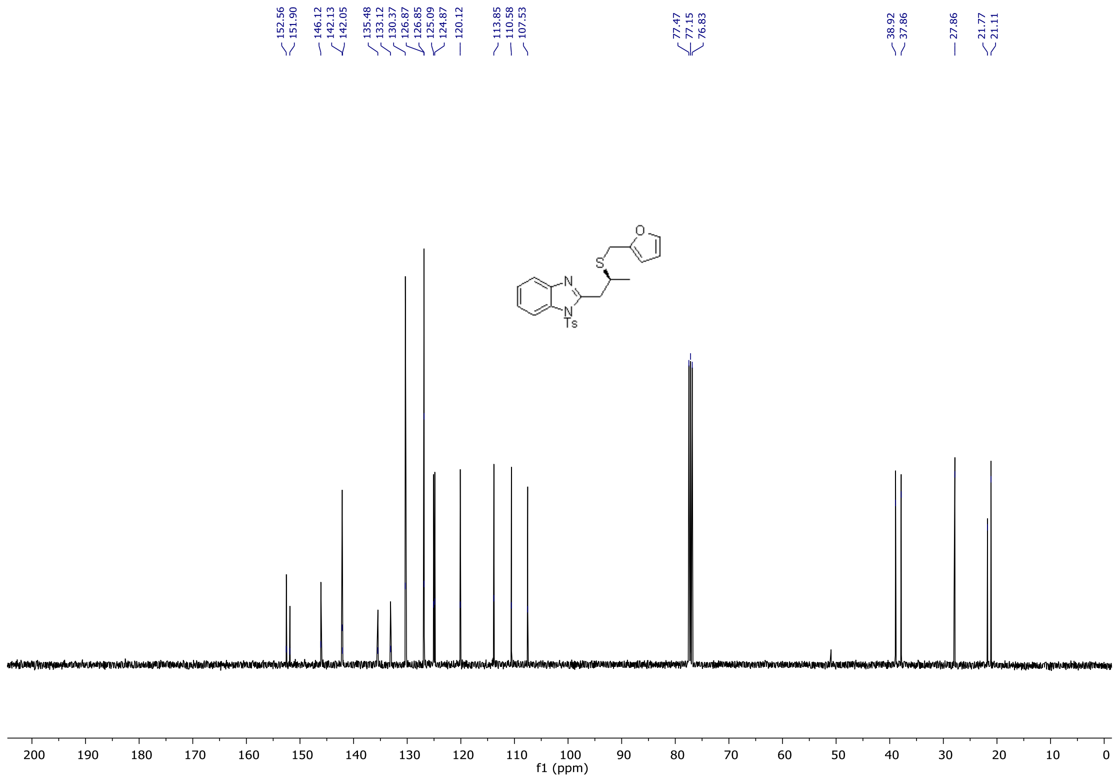

Spectra for **9**:

^{1}H NMR:

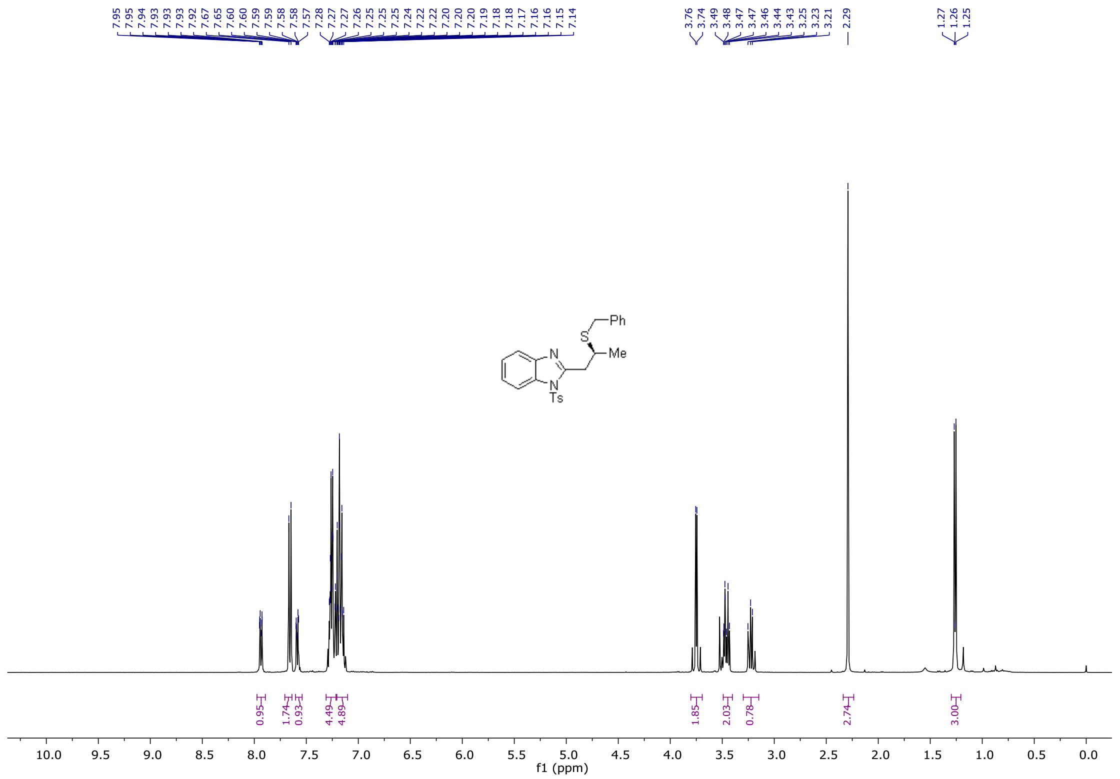

^{13}C NMR:

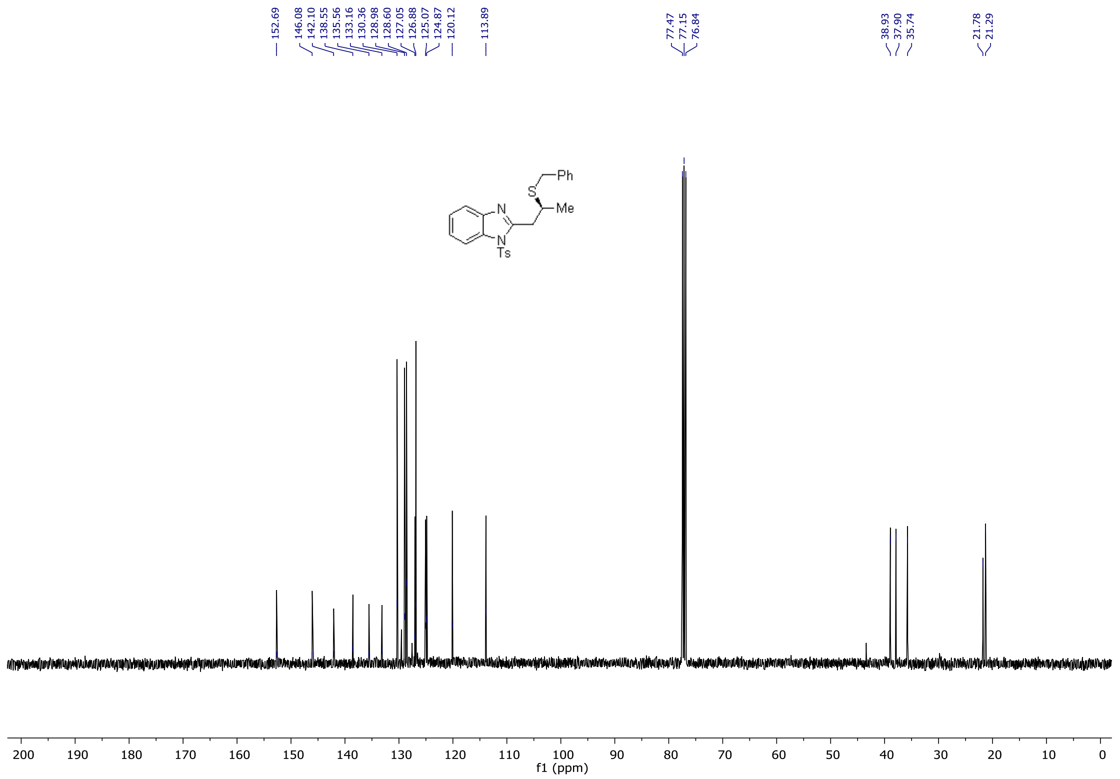

Spectra for **10**:

^{1}H NMR:

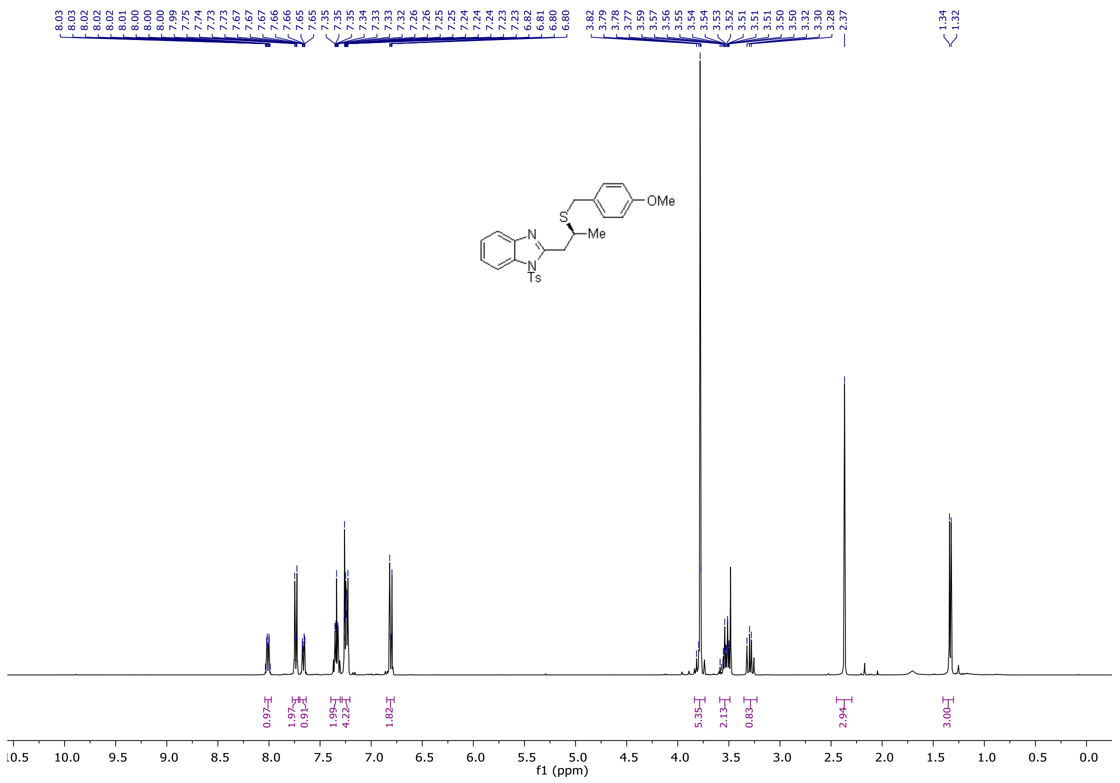

^{13}C NMR:

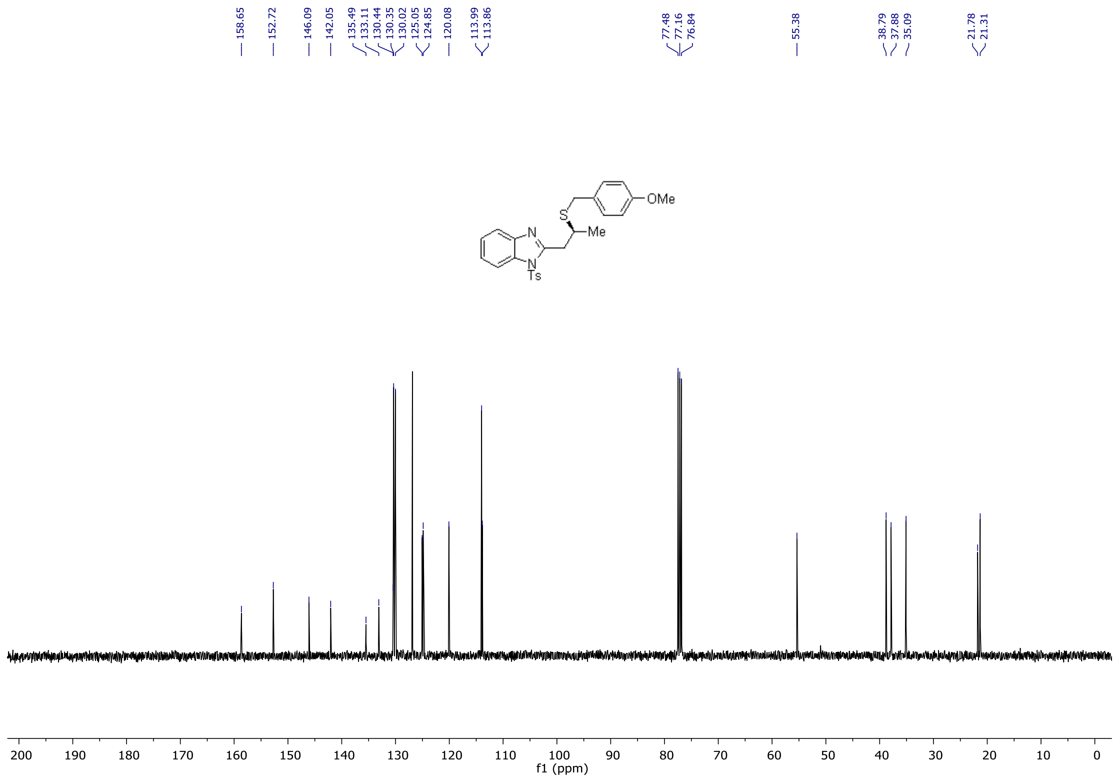

Spectra for **11**:

^{1}H NMR:

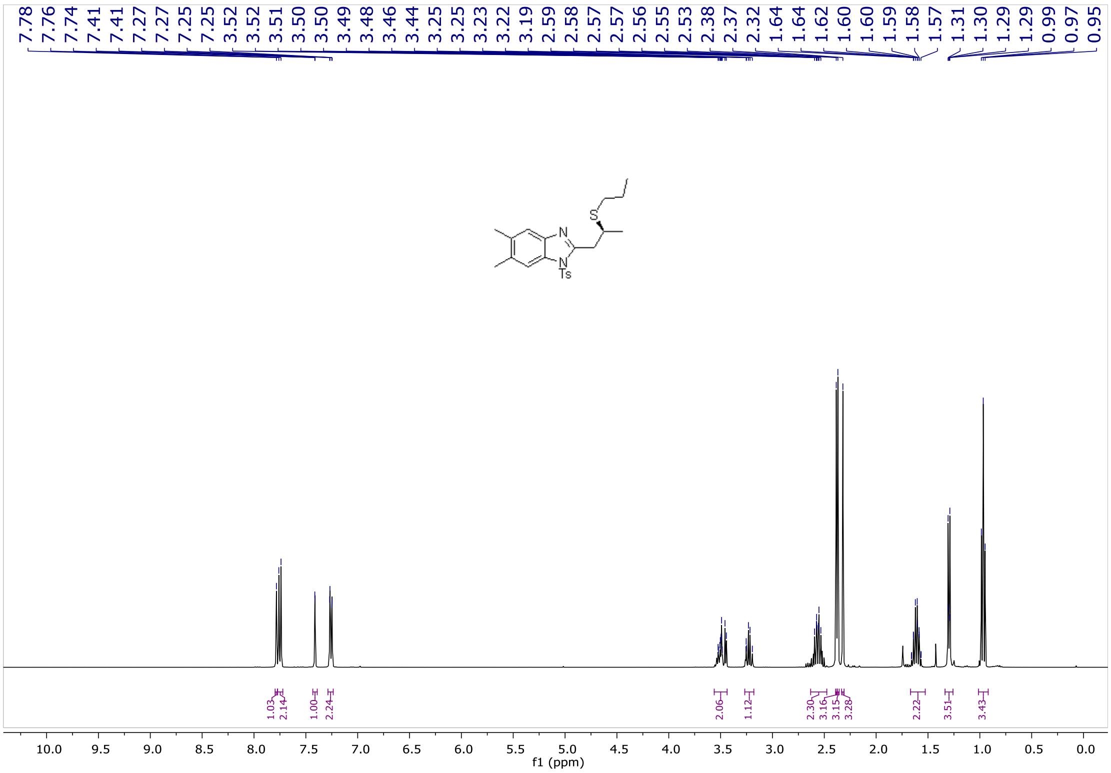

^{13}C NMR:

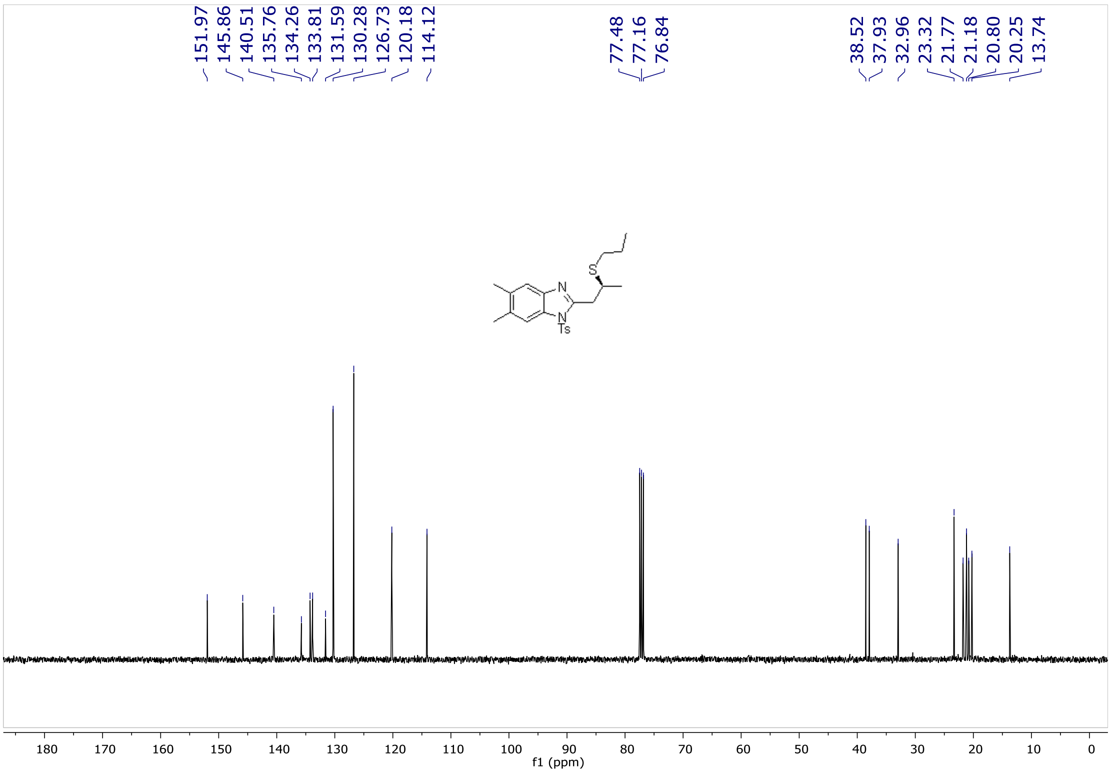

Spectra for **12**:

^{1}H NMR:

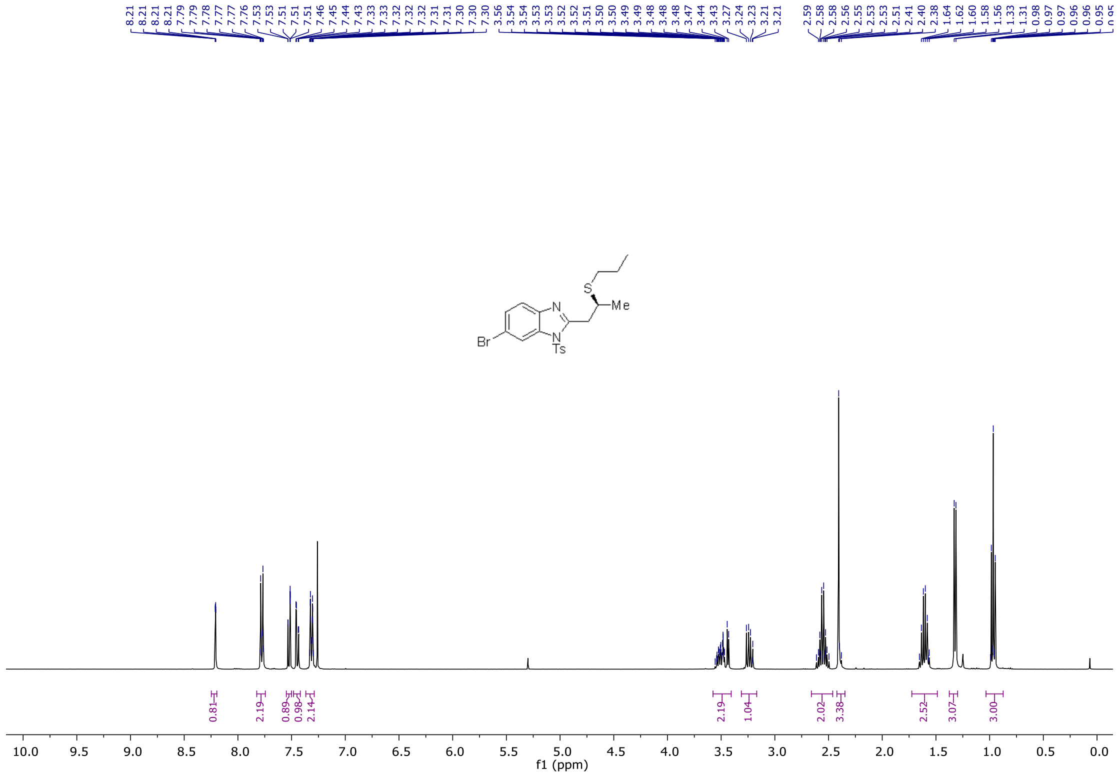

^{13}C NMR:

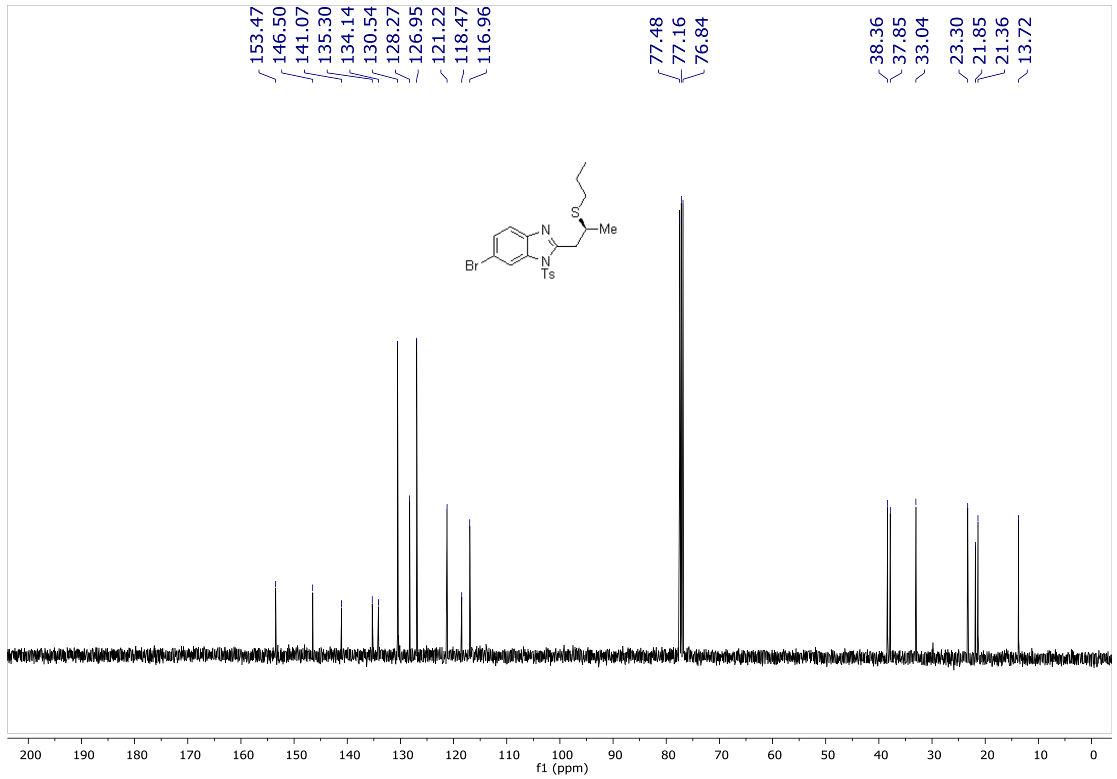

Spectra for **13**:

^{1}H NMR:

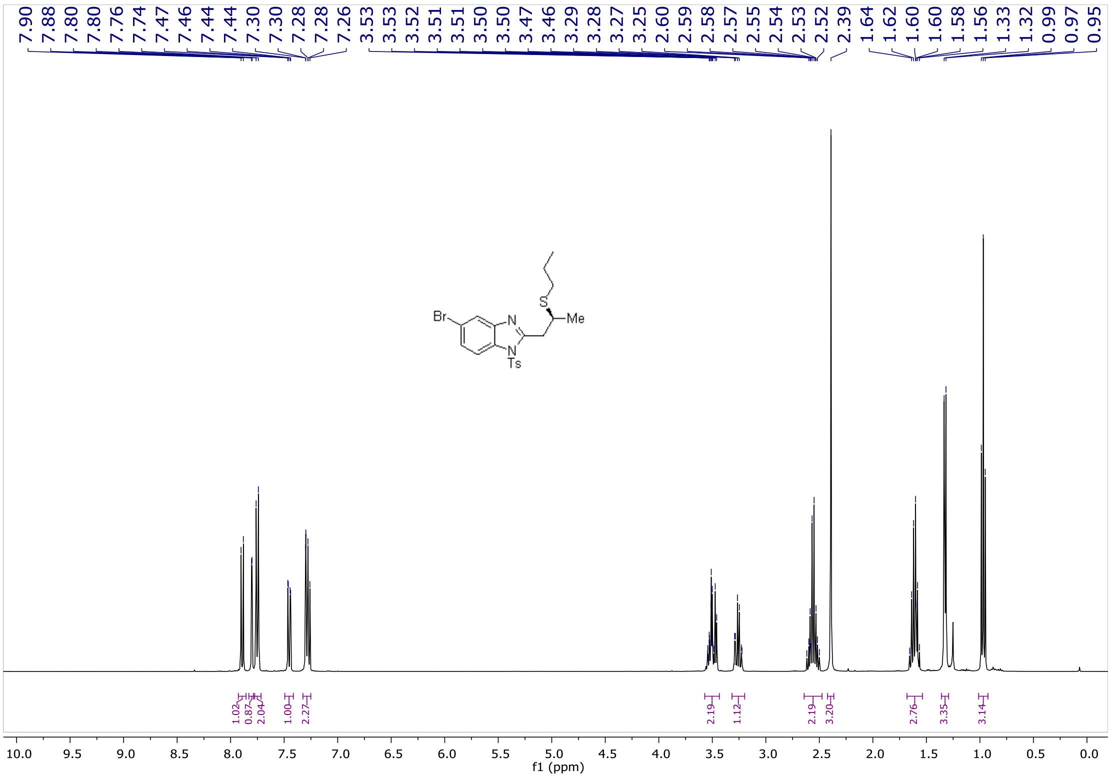

^{13}C NMR:

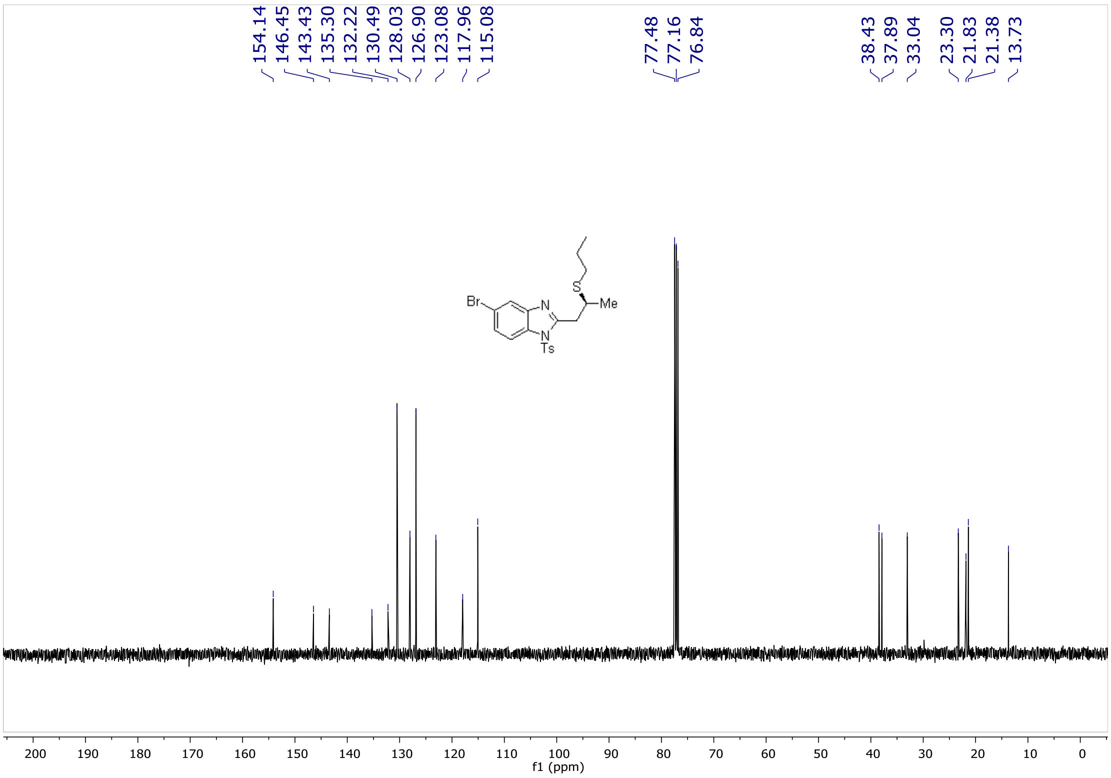

Spectra for **14**:

^{1}H NMR:

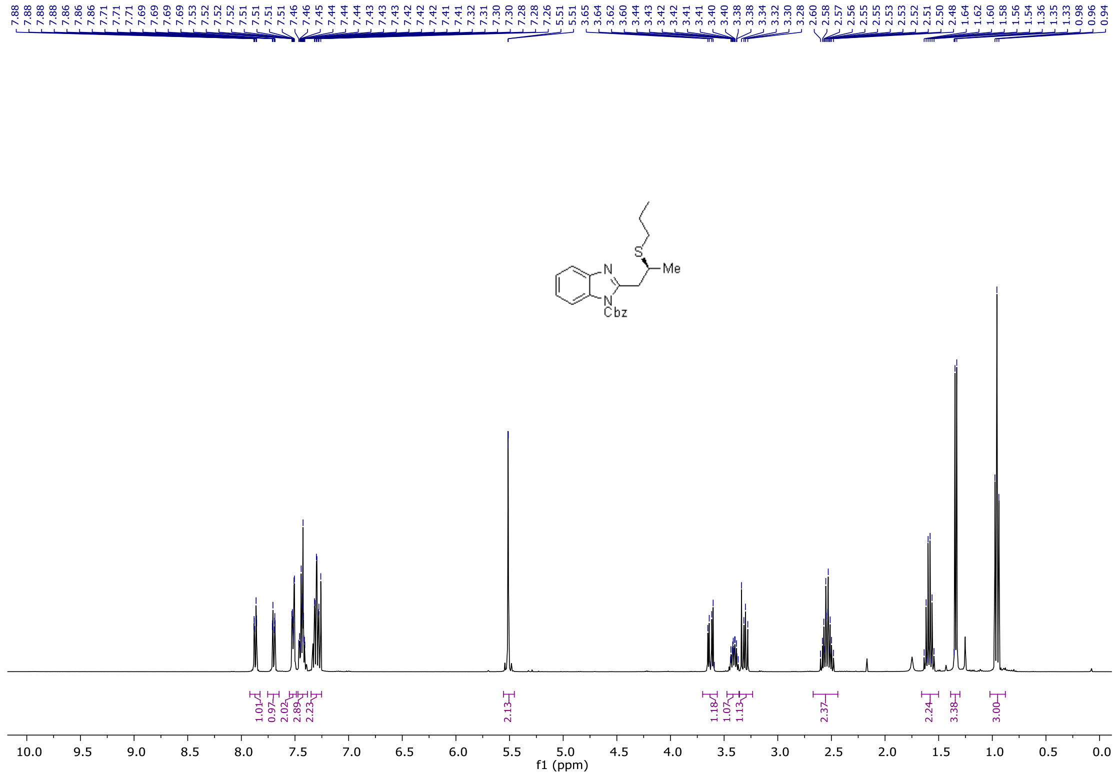

^{13}C NMR:

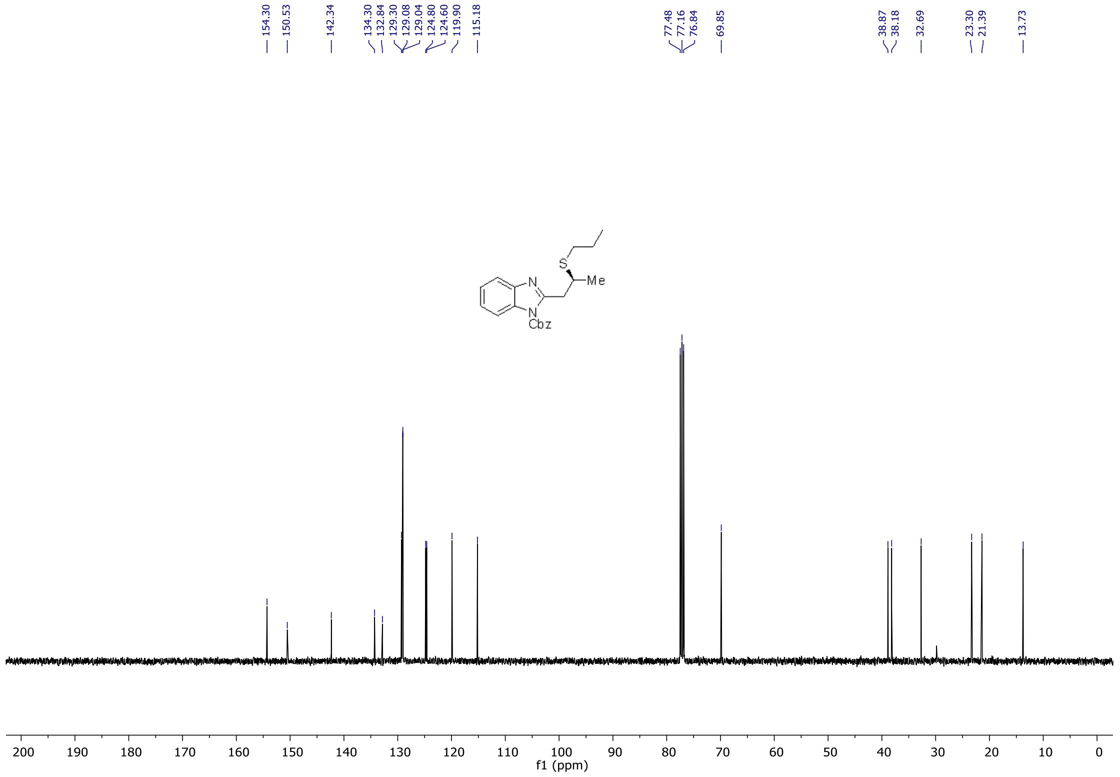

Spectra for **15**:

^{1}H NMR:

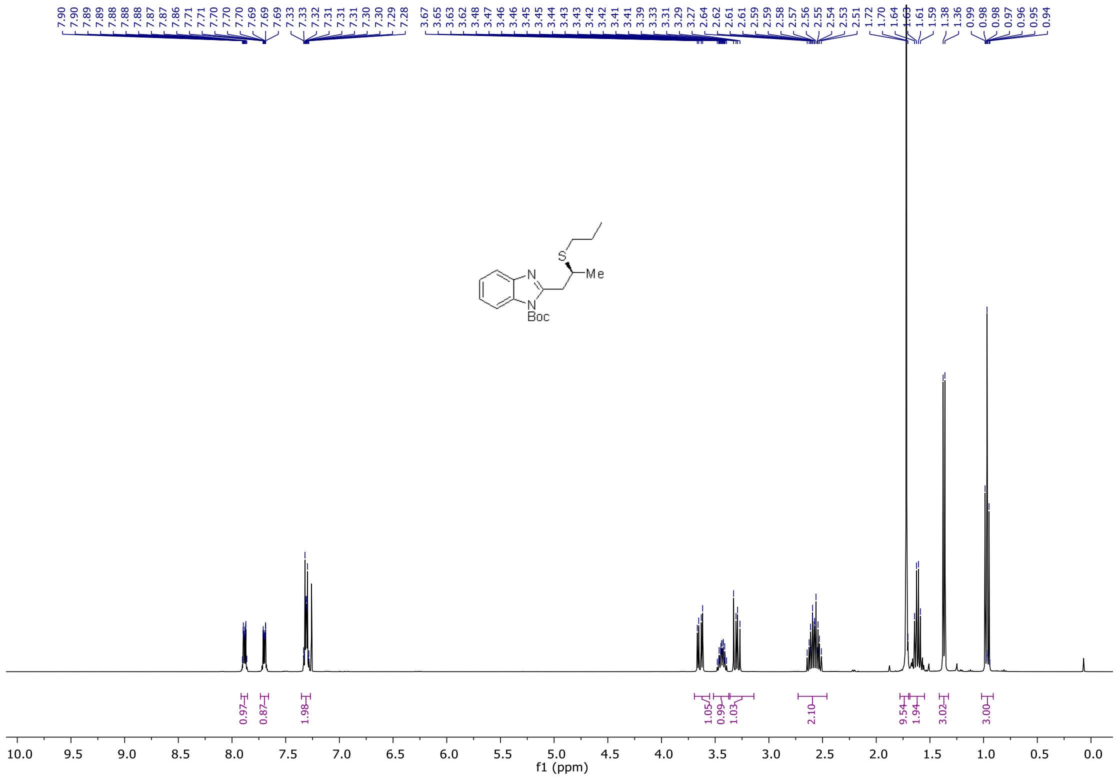

^{13}C NMR:

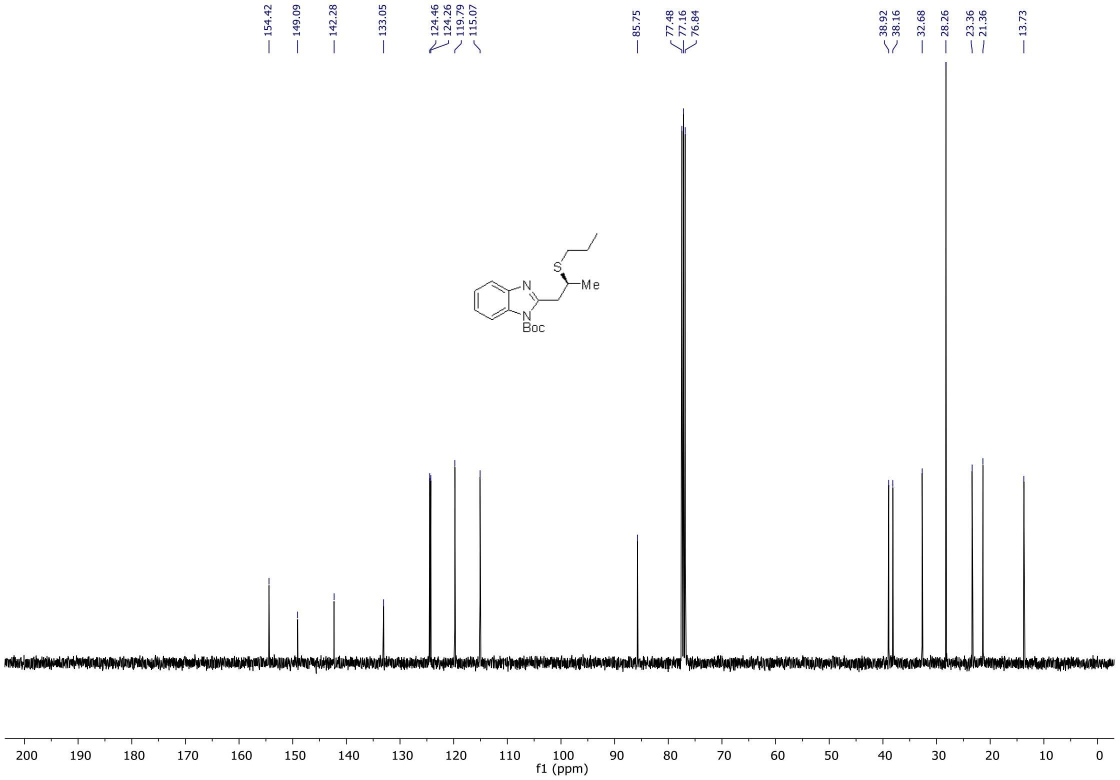

Spectra for **16**:

^{1}H NMR:

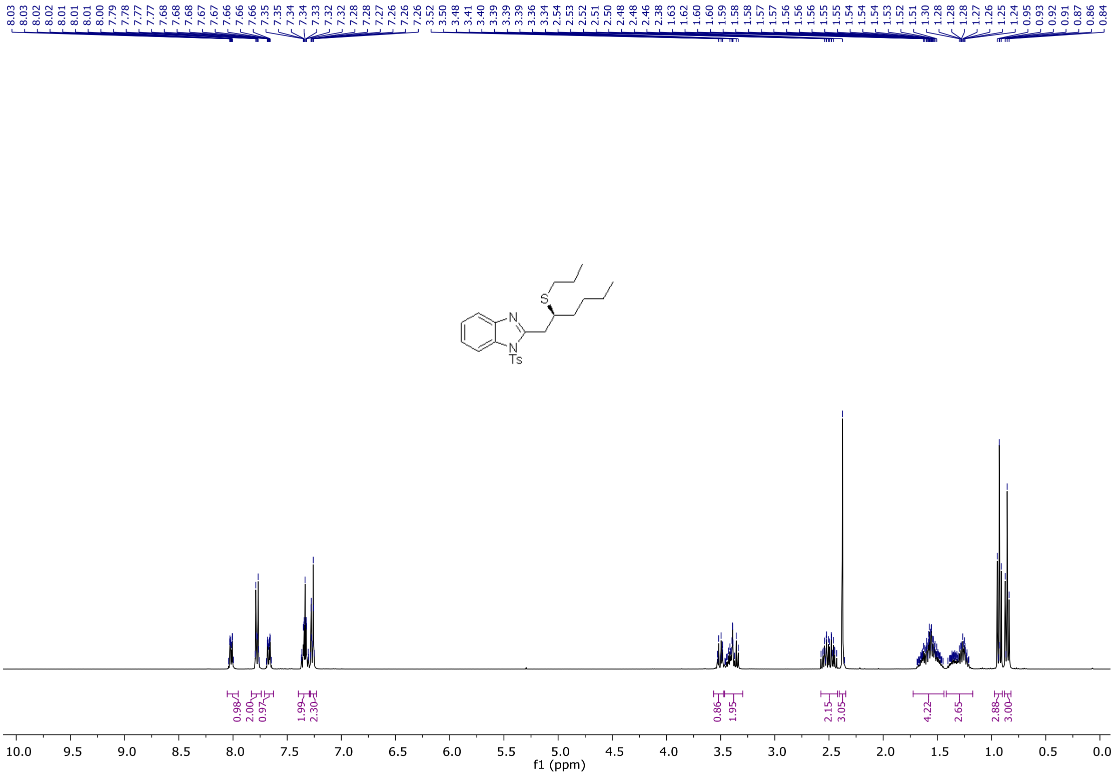

^{13}C NMR:

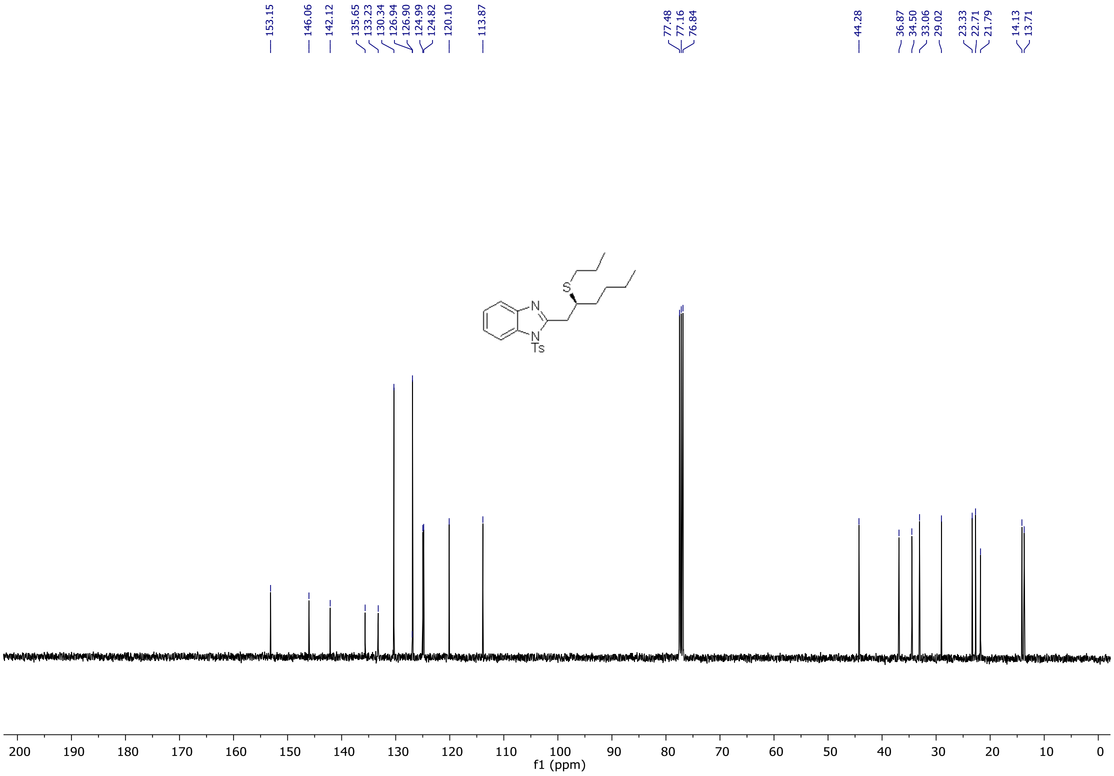

Spectra for **17**:

^{1}H NMR:

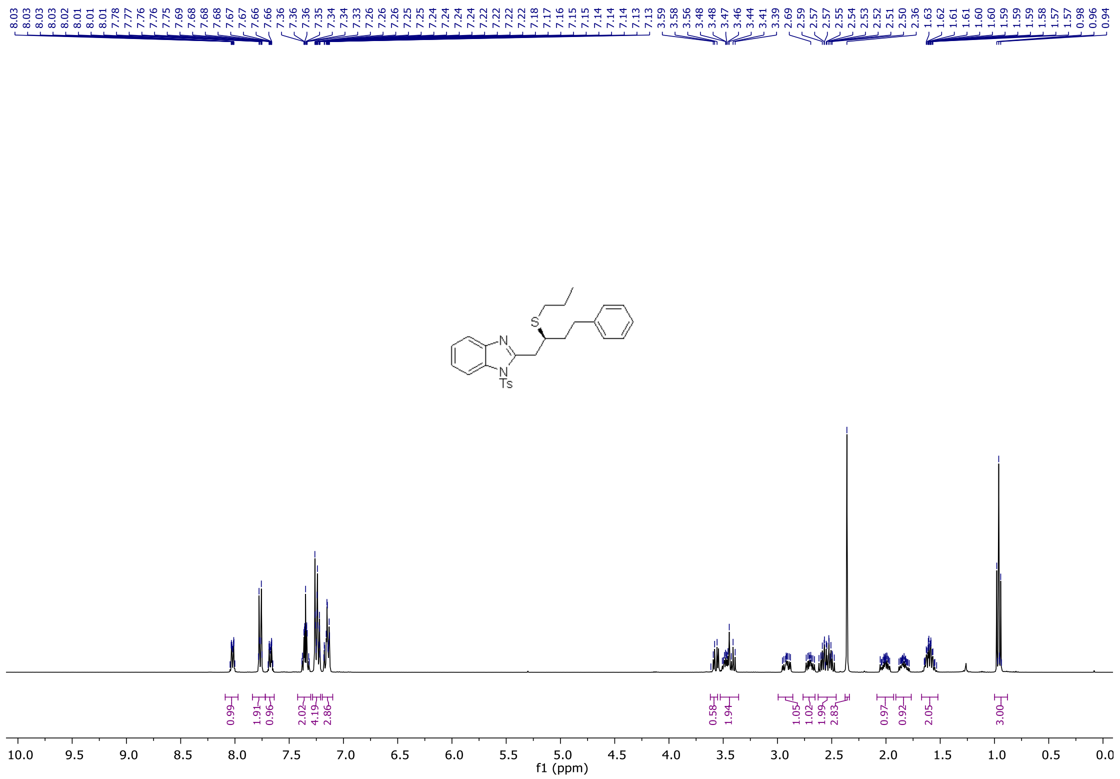

^{13}C NMR:

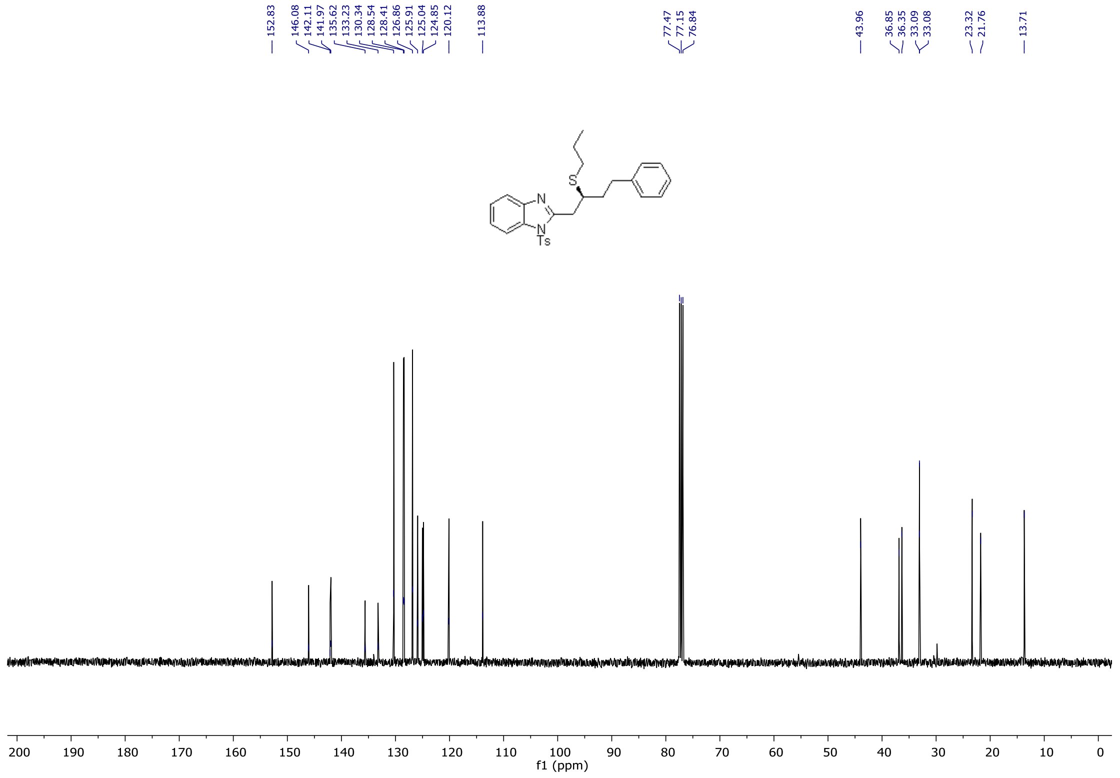

Spectra for **18**:

^{1}H NMR:

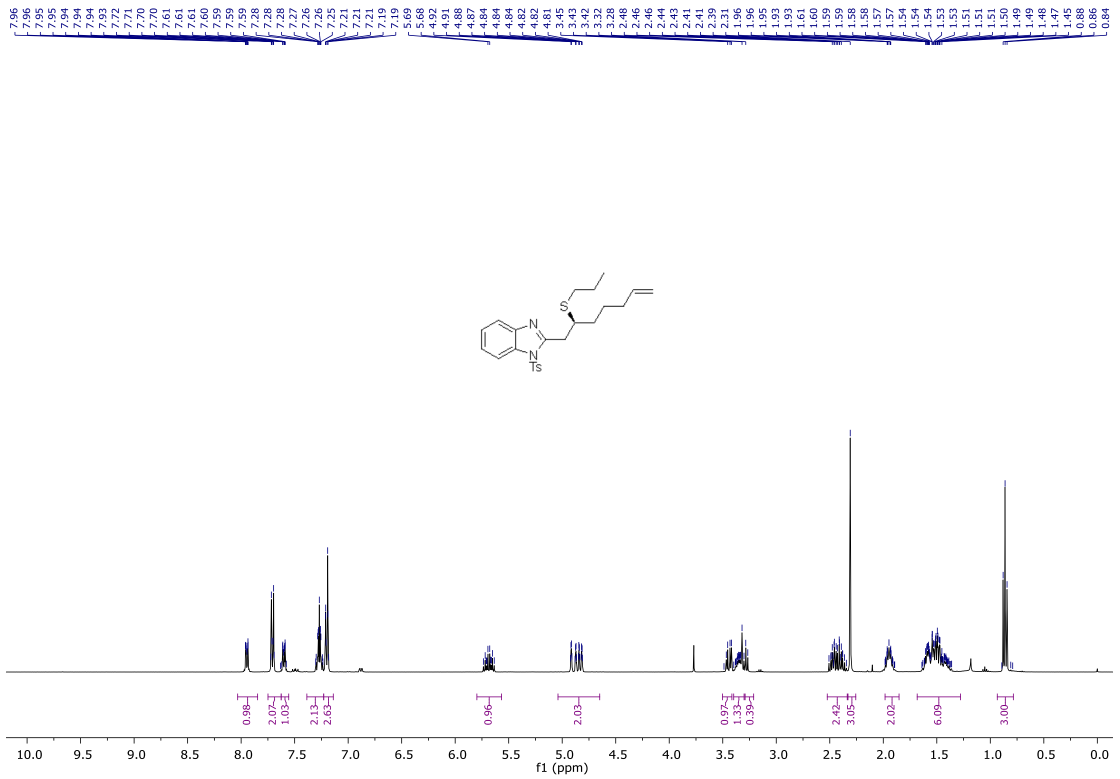

^{13}C NMR:

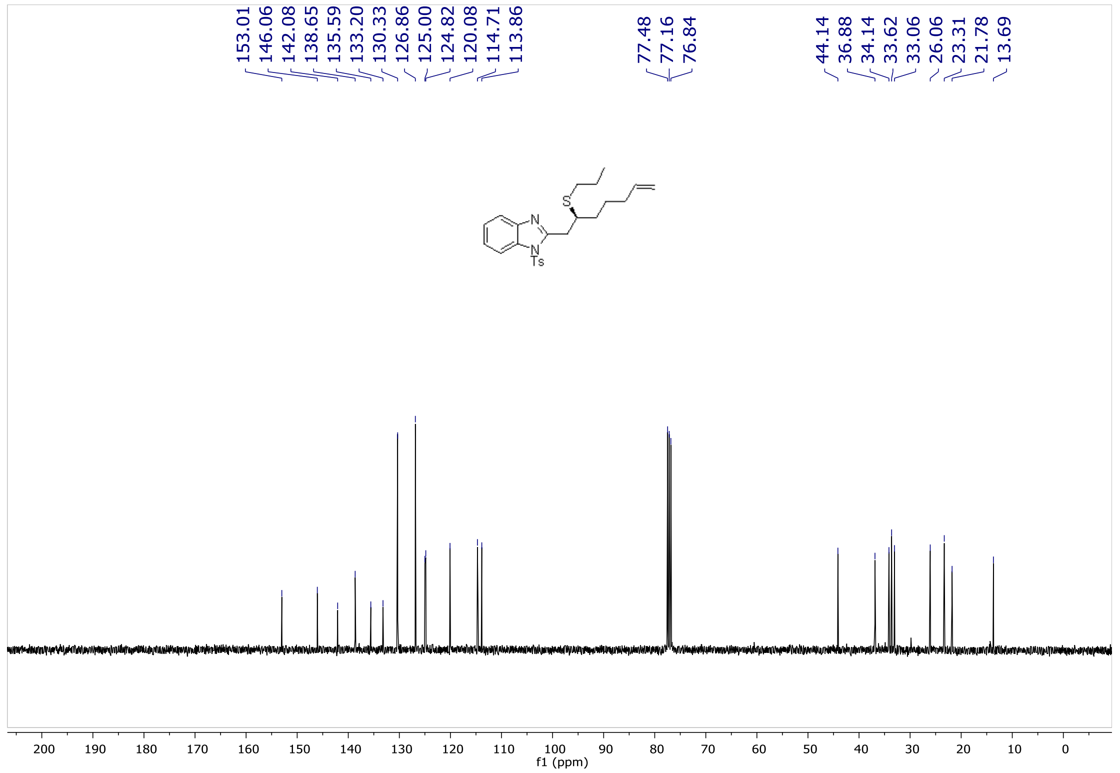

Spectra for **19**:

^{1}H NMR:

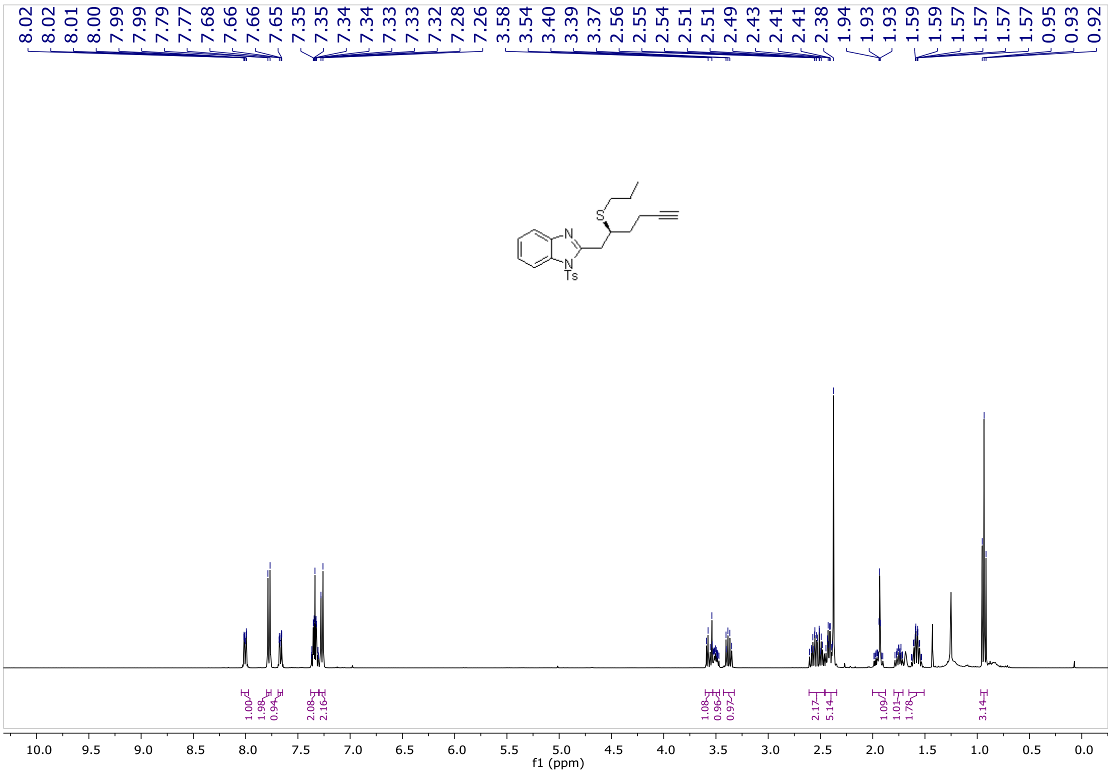

^{13}C NMR:

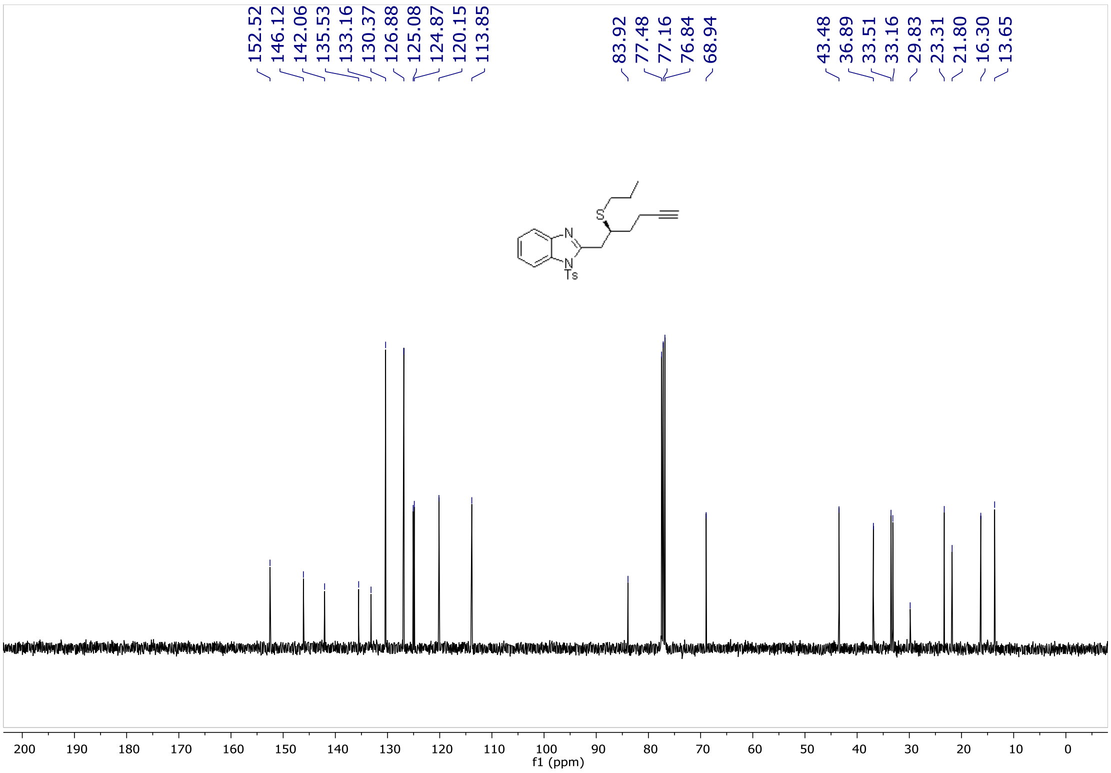

Spectra for **20**:

^{1}H NMR:

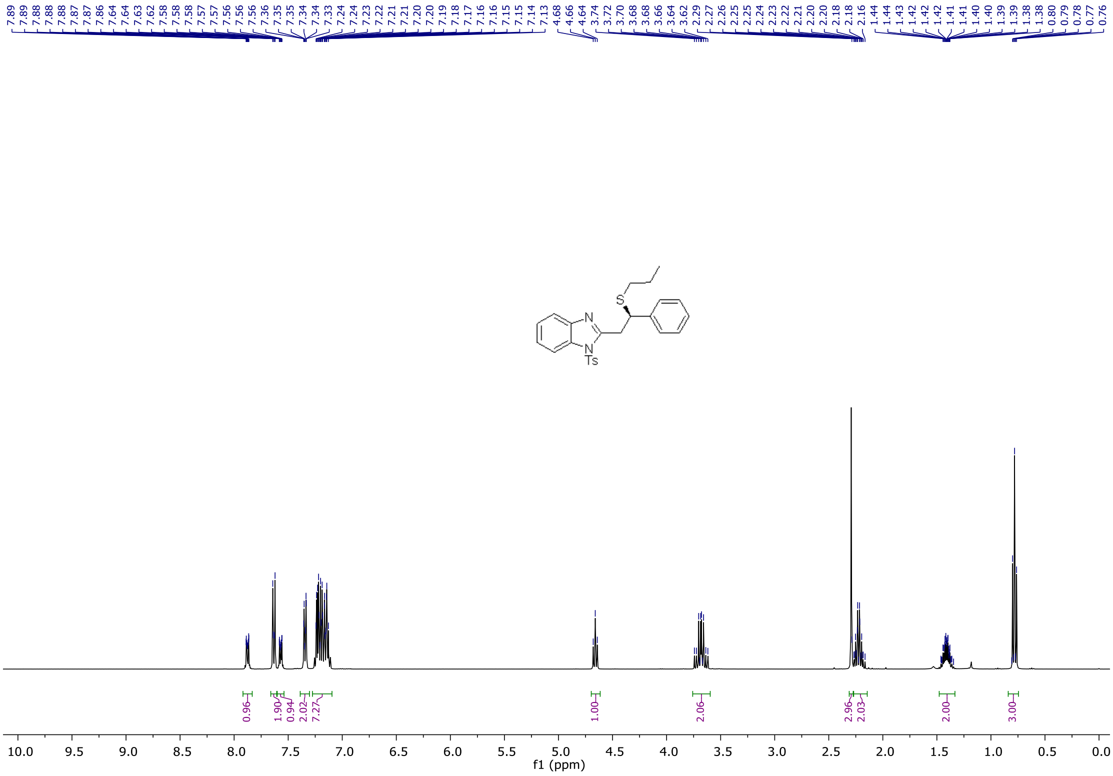

^{13}C NMR:

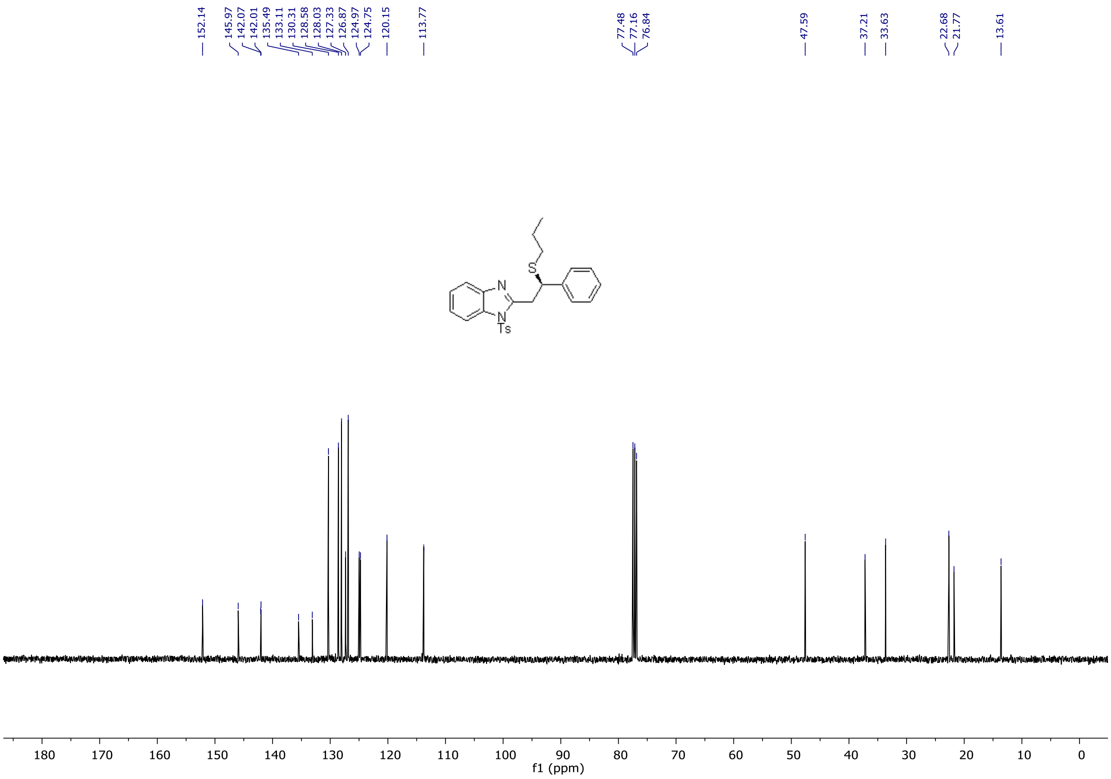

Spectra for **21**:

^{1}H NMR:

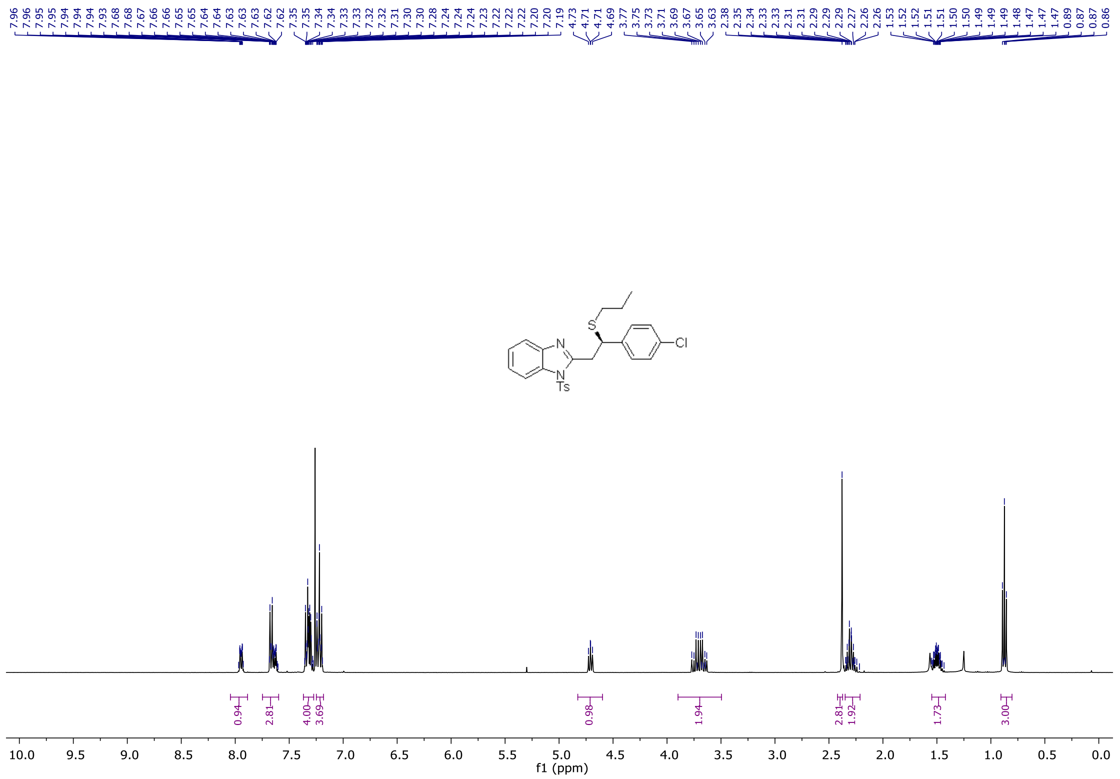

^{13}C NMR:

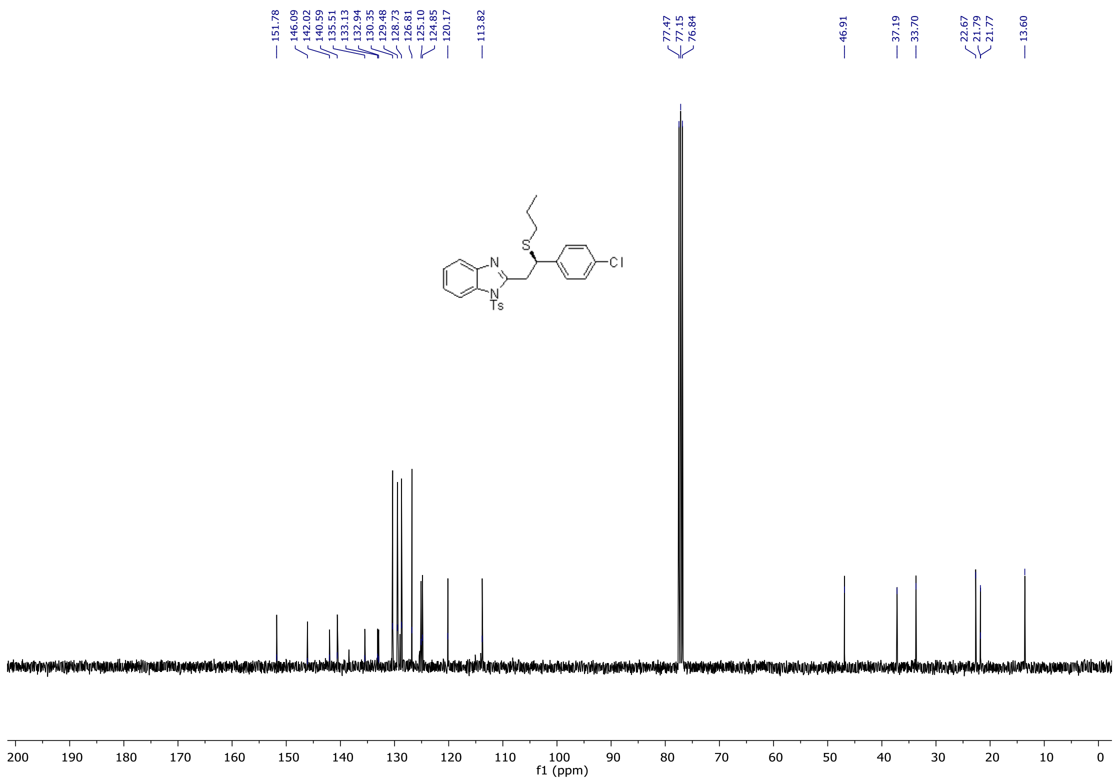

Spectra for **22**:

^{1}H NMR:

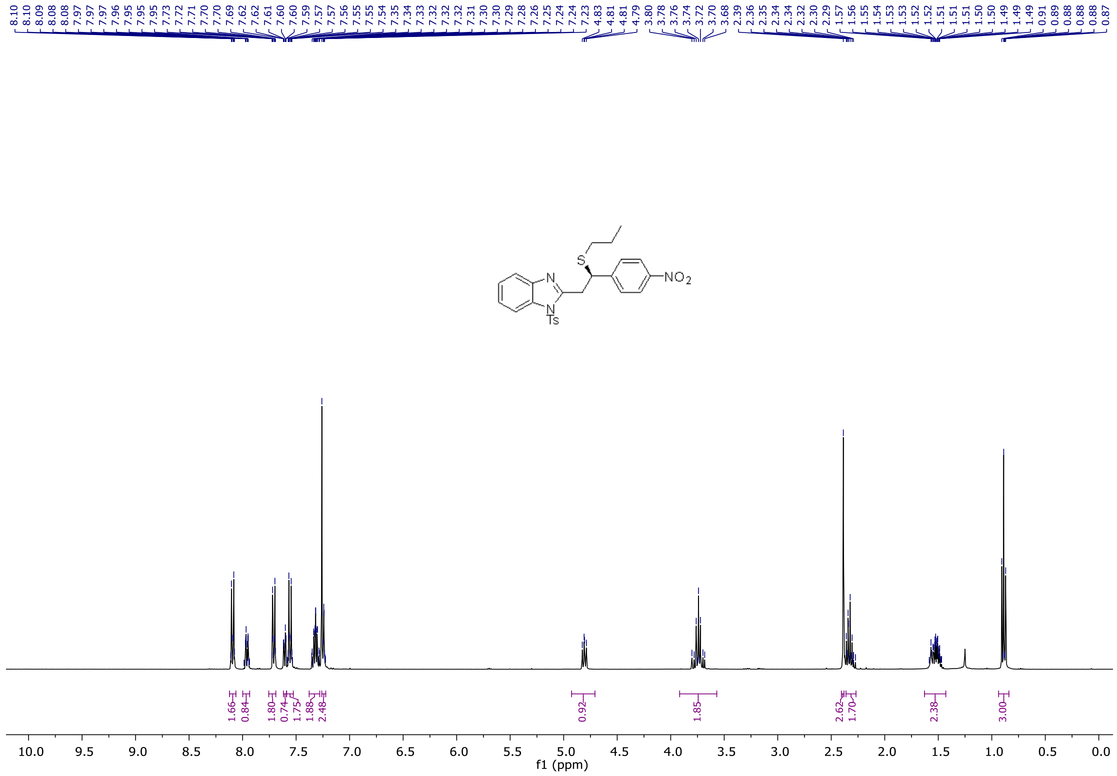

^{13}C NMR:

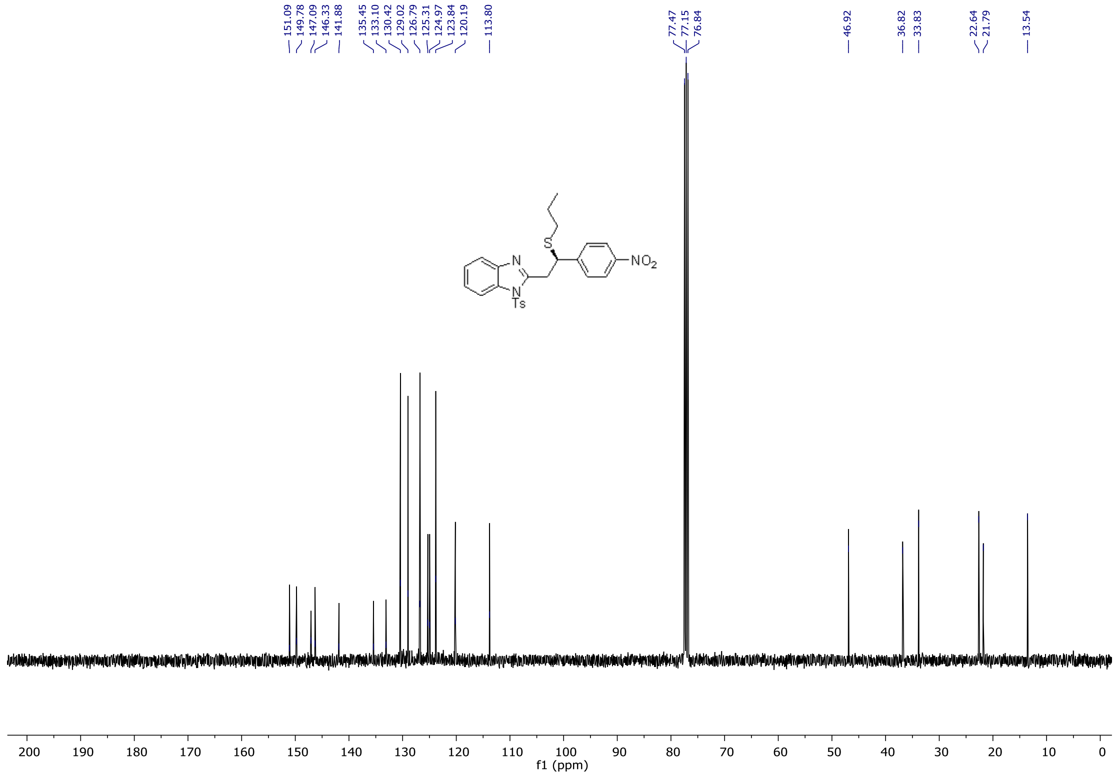

Spectra for **23**:

^{1}H NMR:

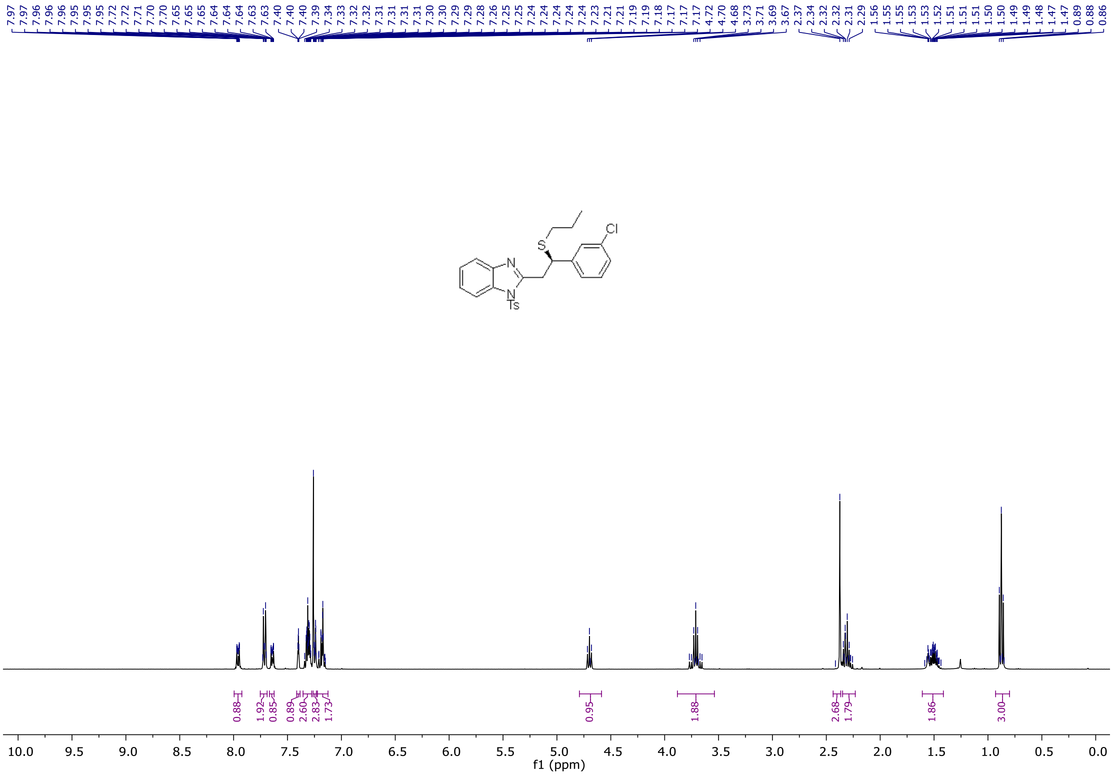

^{13}C NMR:

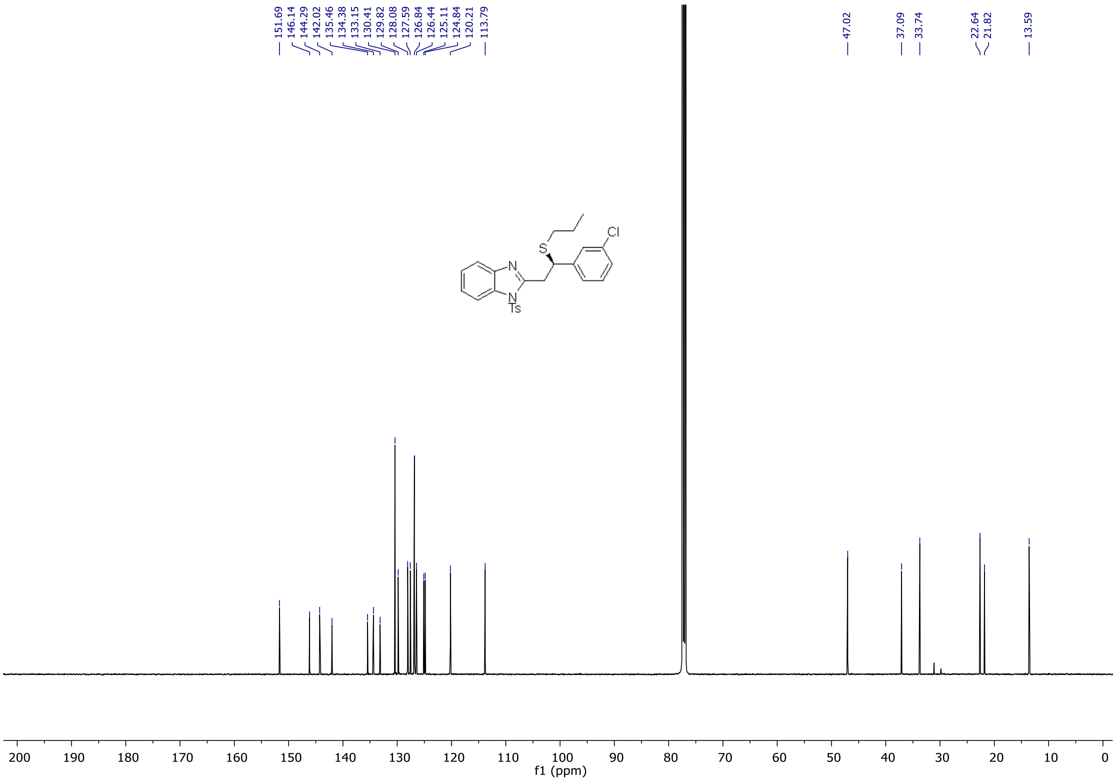

Spectra for **24**:

^{1}H NMR:

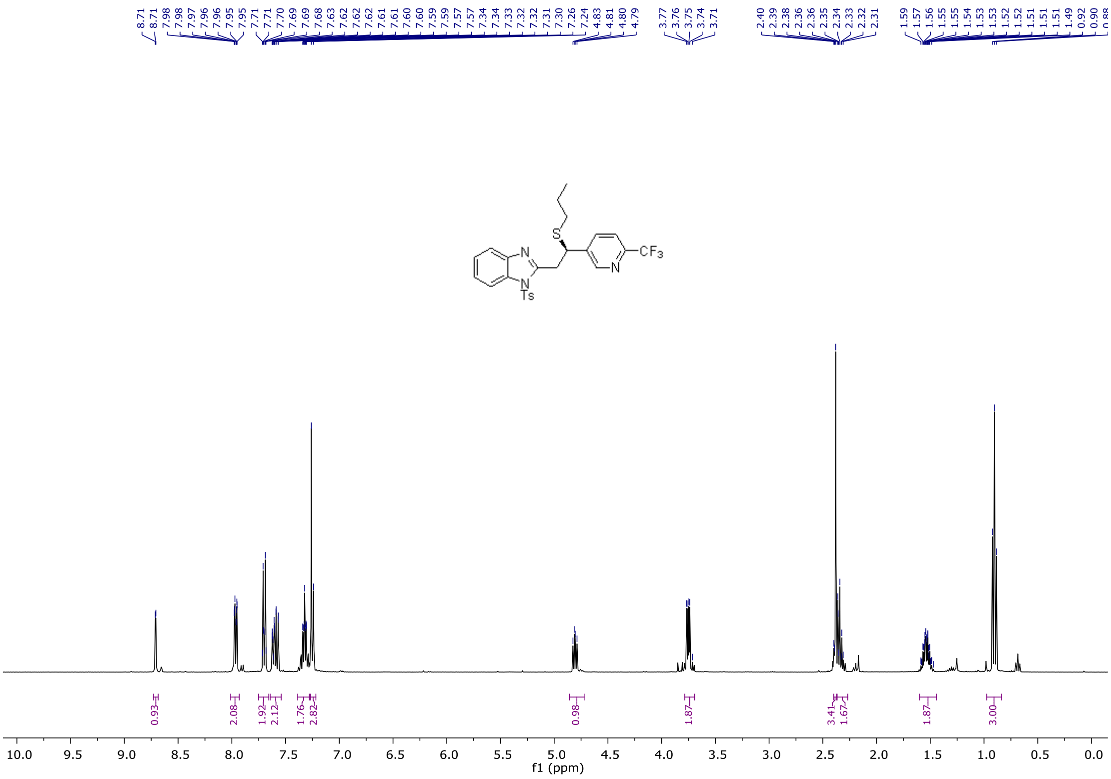

^{13}C NMR:

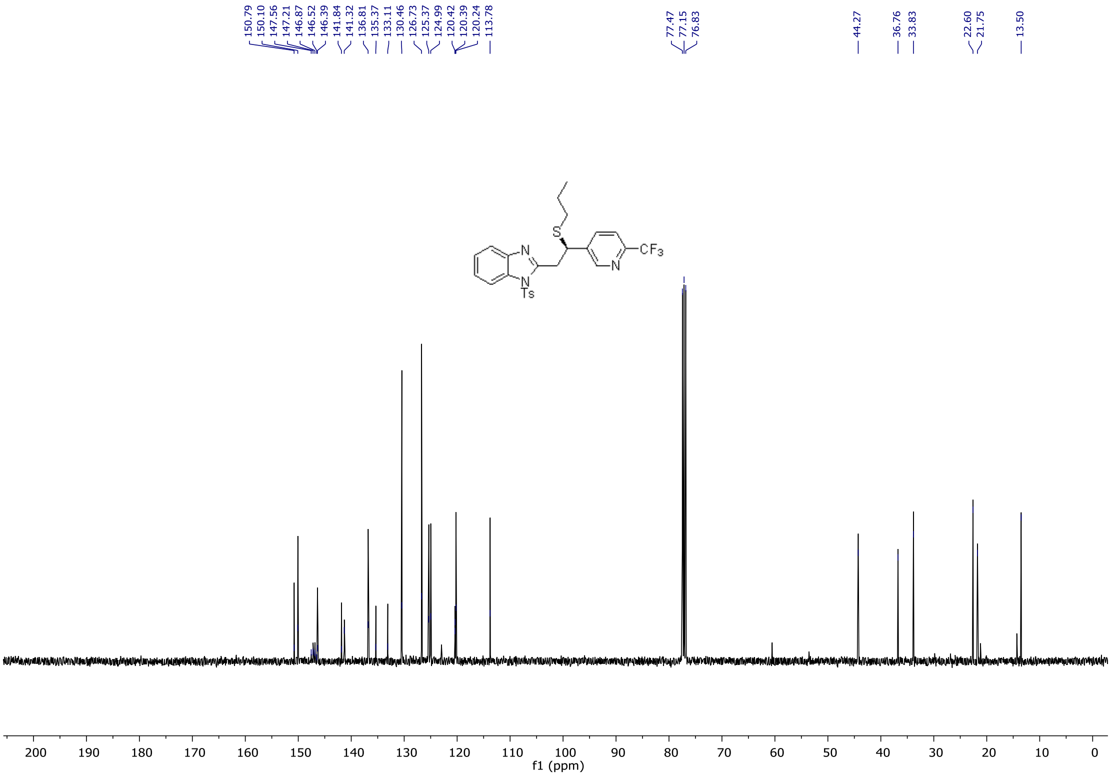

^{19}F NMR:

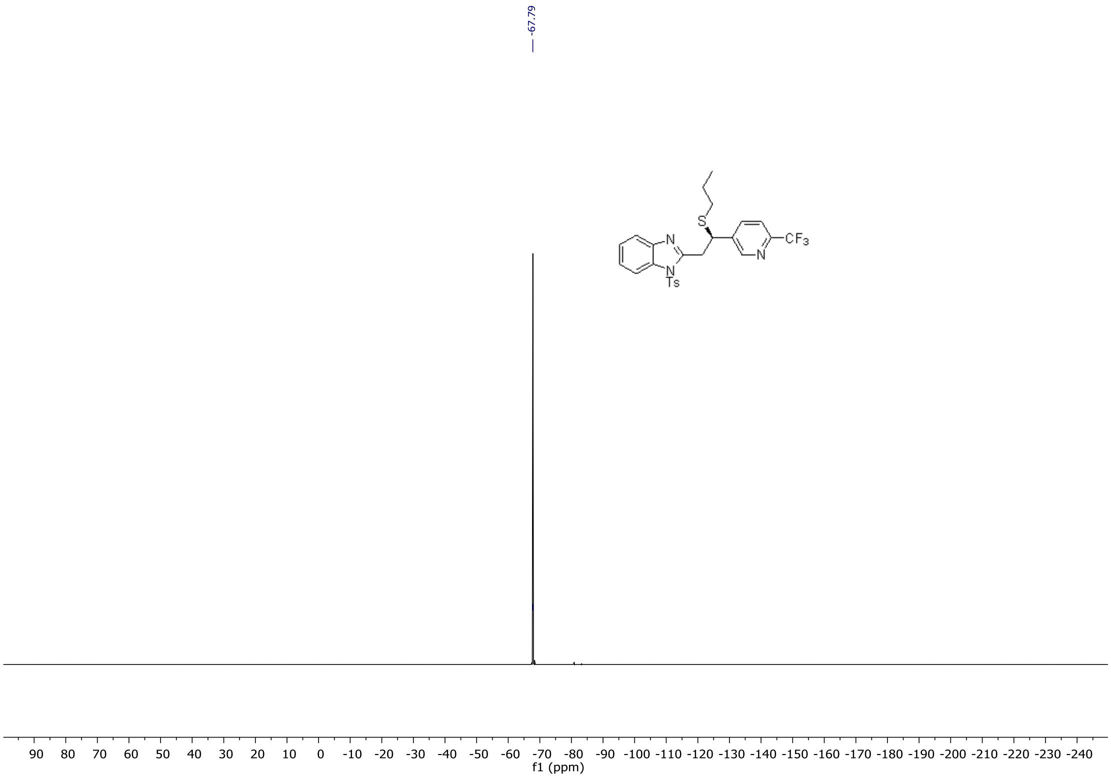

-67.79
S
N
N
Ts
N
CF3
90 80 70 60 50 40 30 20 10 0 -10 -20 -30 -40 -50 -60 -70 -80 -90 -100 -110 -120 -130 -140 -150 -160 -170 -180 -190 -200 -210 -220 -230 -240
f1 (ppm)

Spectra for **25**:

^{1}H NMR:

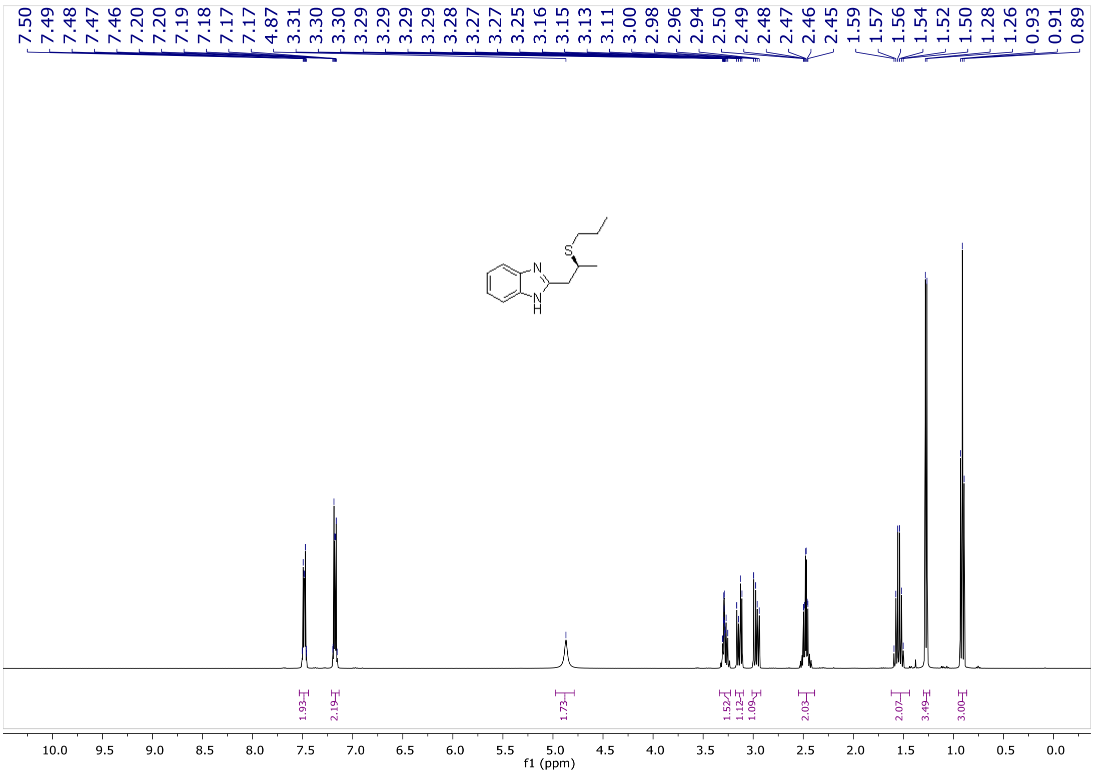

^{13}C NMR:

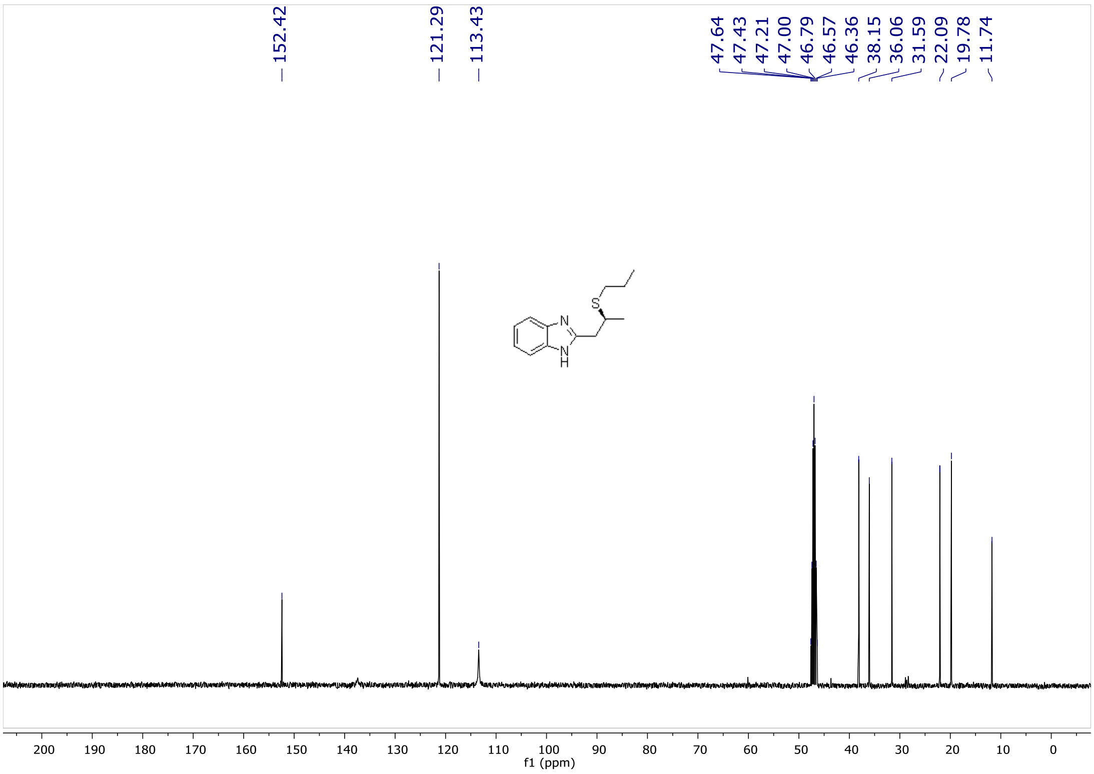

Spectra for **26**:

^{1}H NMR:

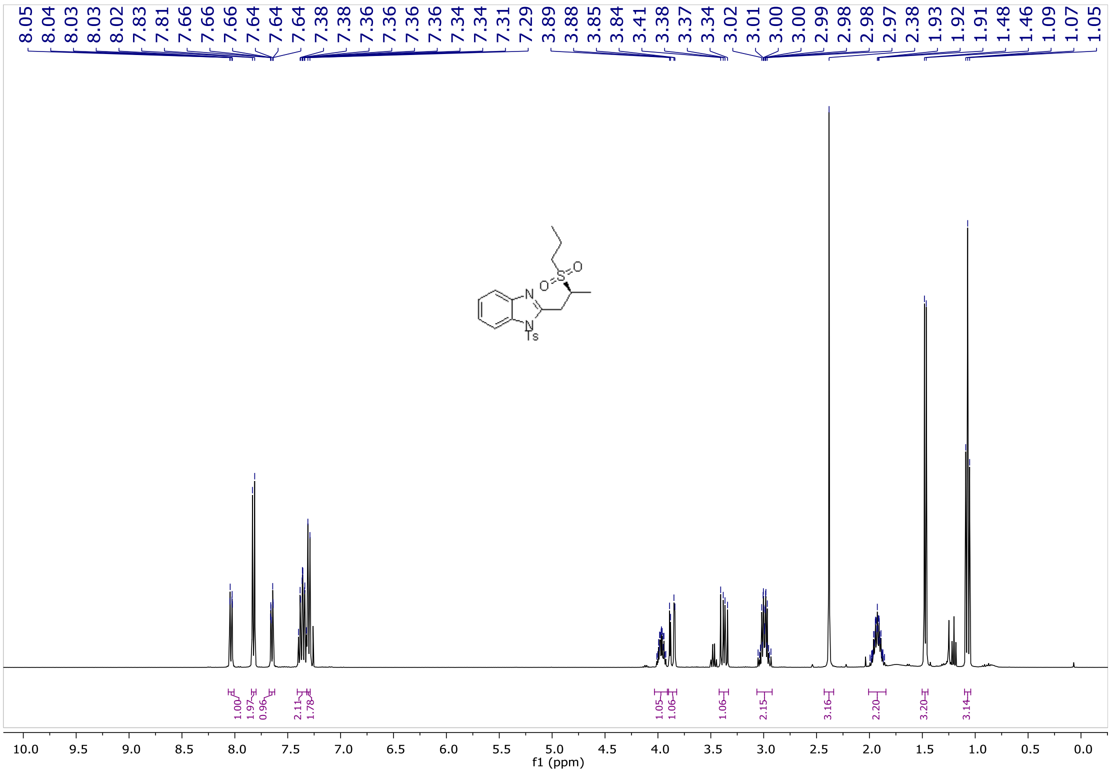

^{13}C NMR:

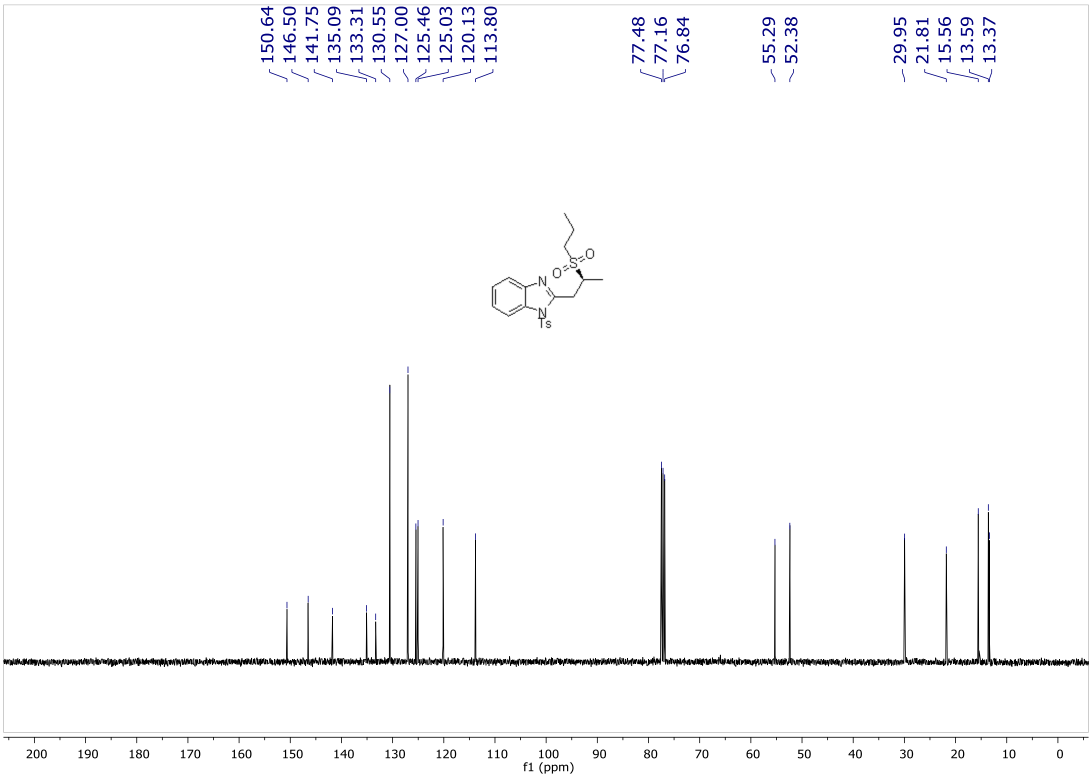

Spectra for **27 (see foonote 15)**:

^{1}H NMR:

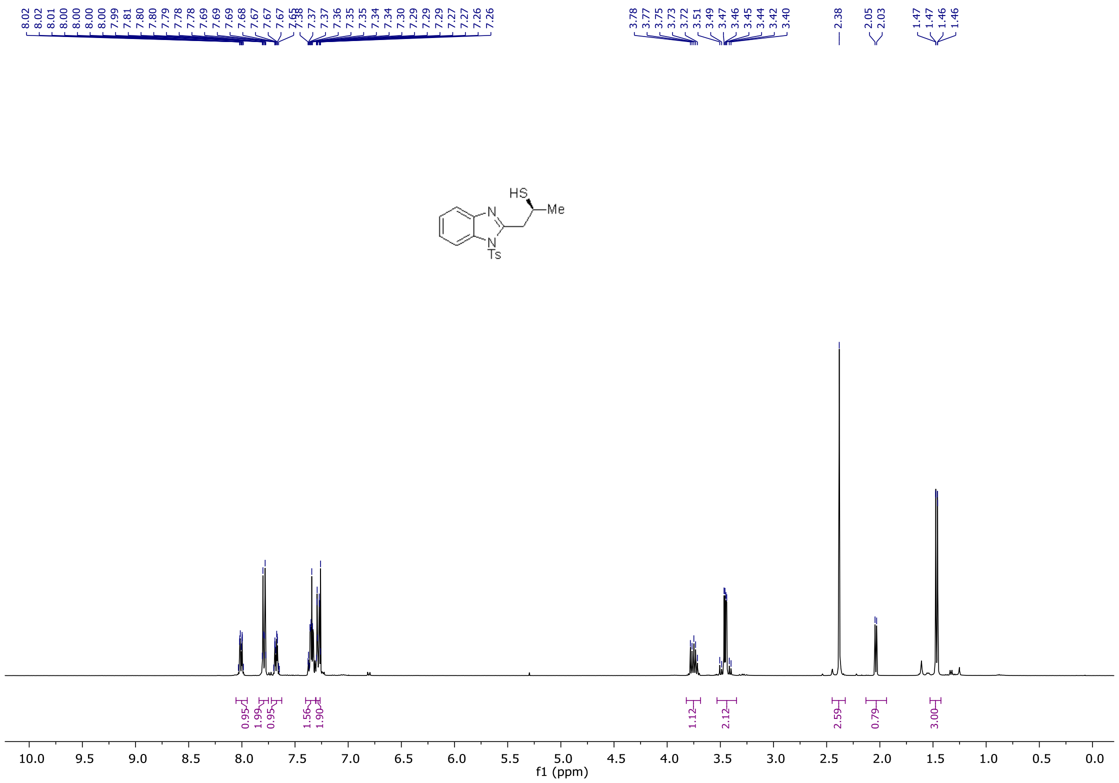

^{13}C NMR:

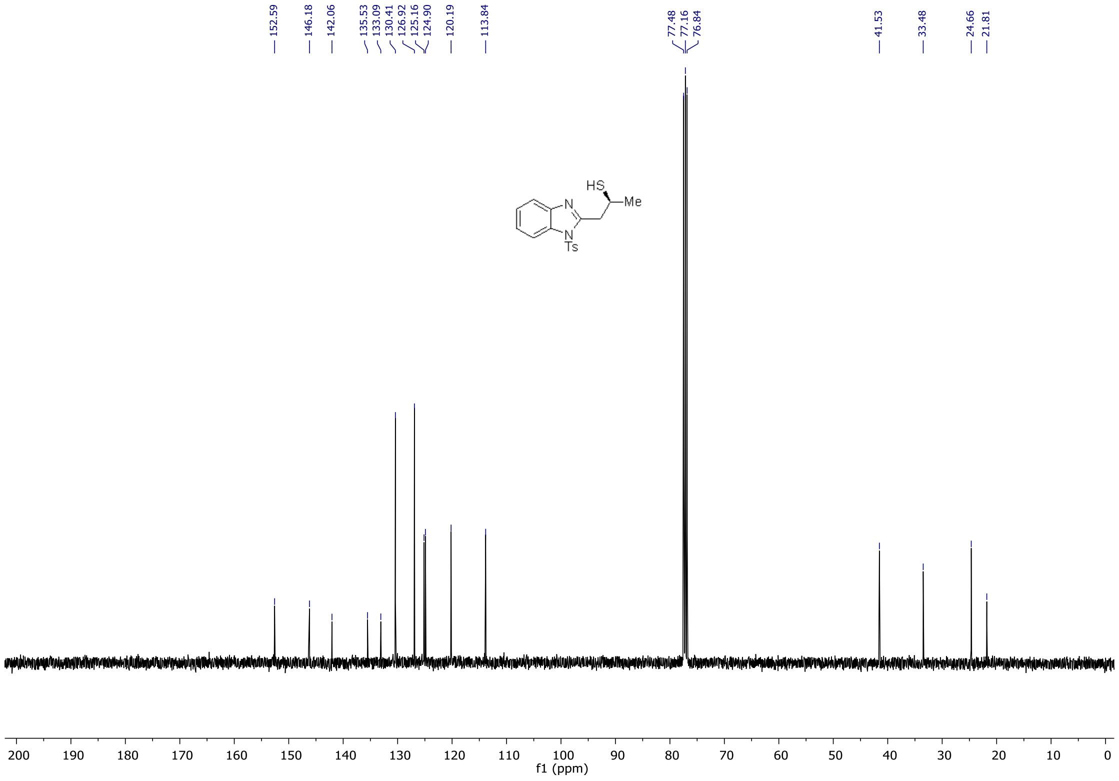

Spectra for **28 (see footnote 16)**

^{1}H NMR:

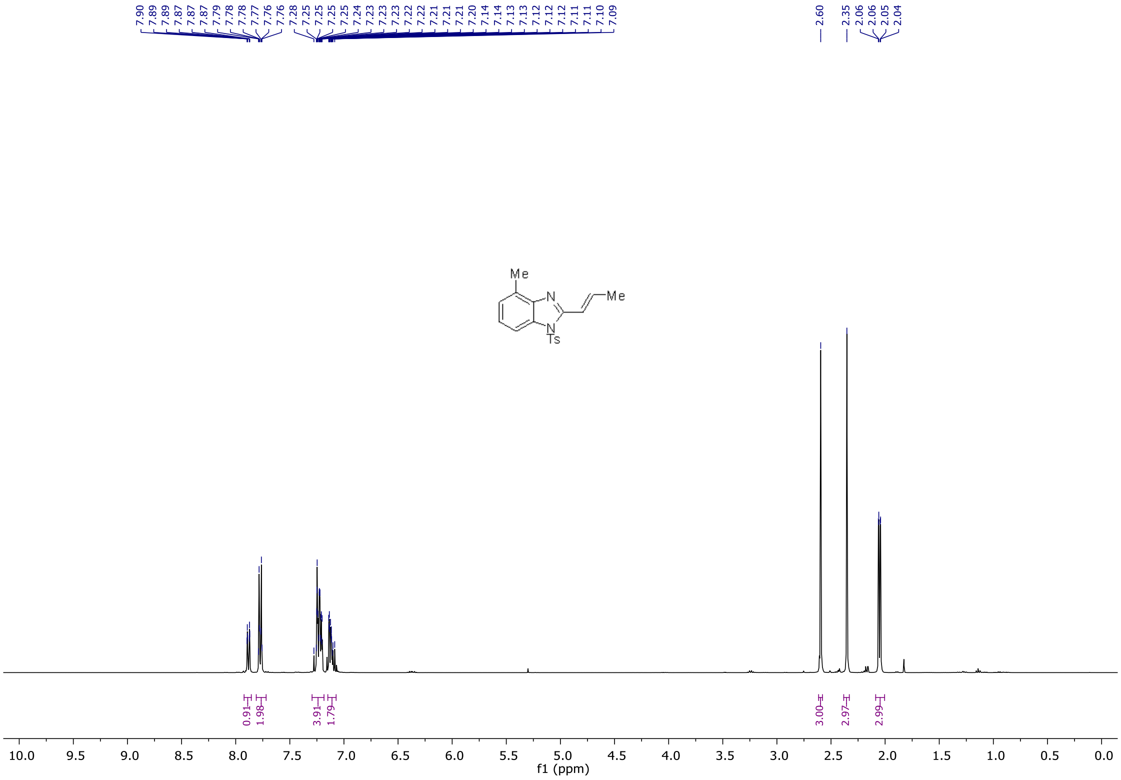

^{13}C NMR:

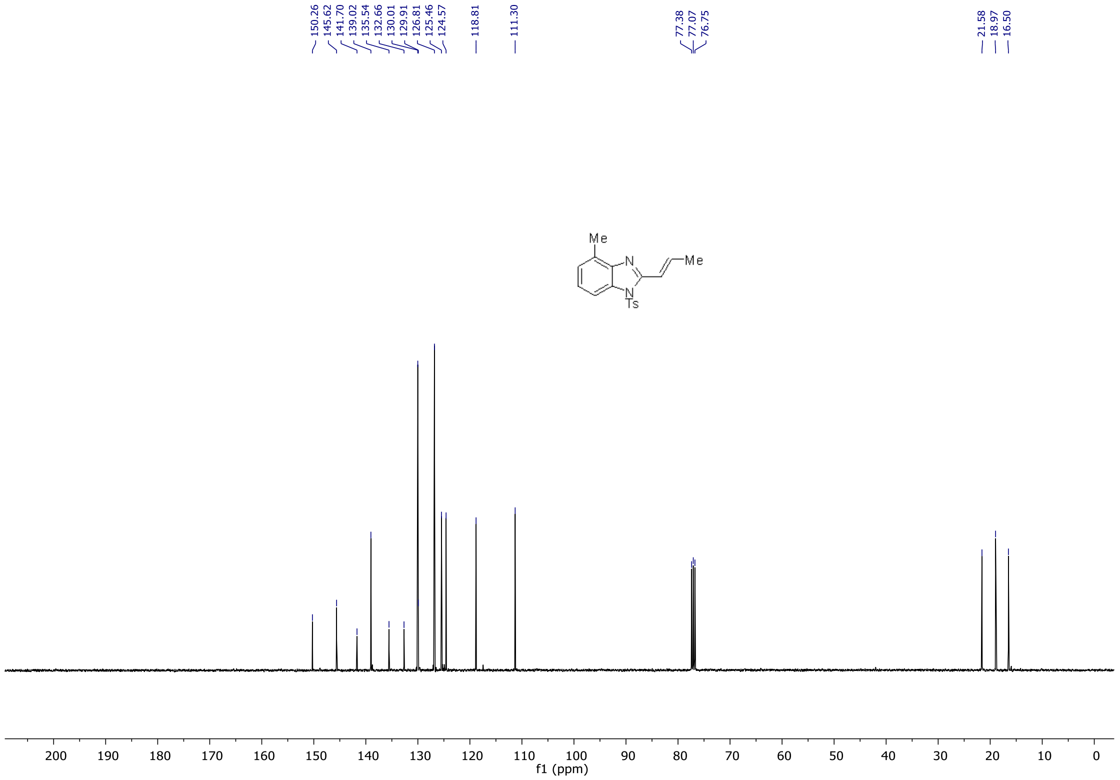

nOe:

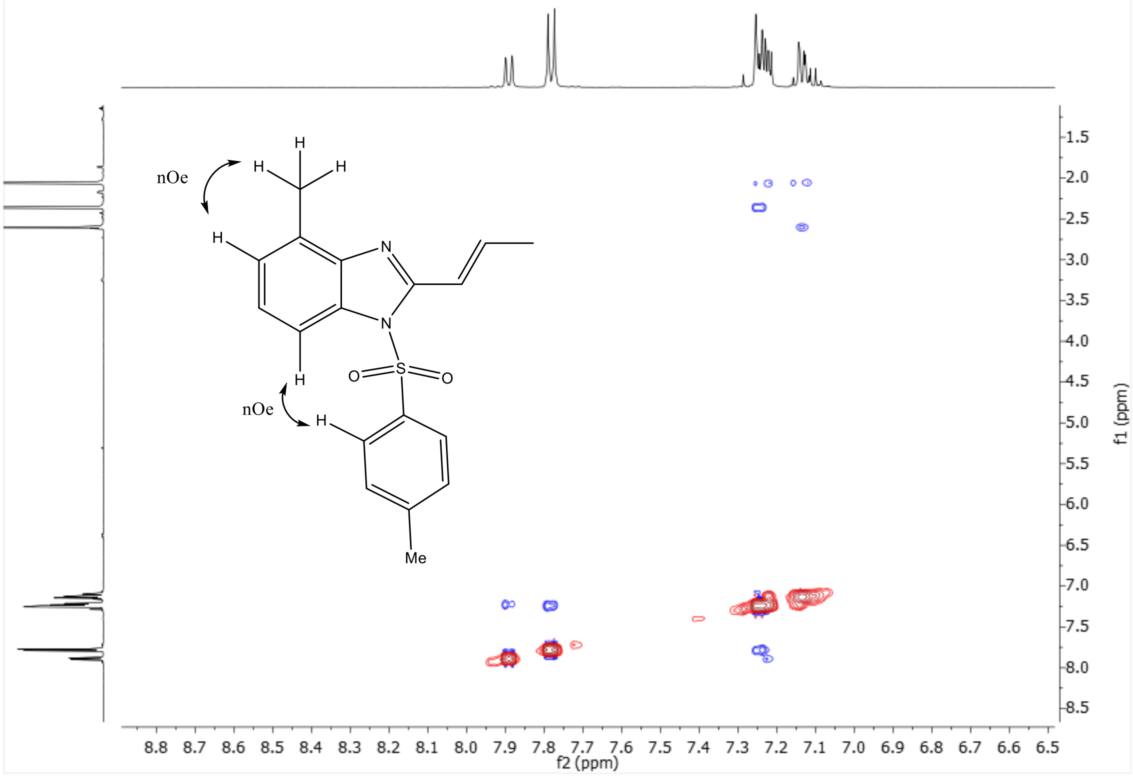

Spectra for **29 (see footnote 16)**

^{1}H NMR:

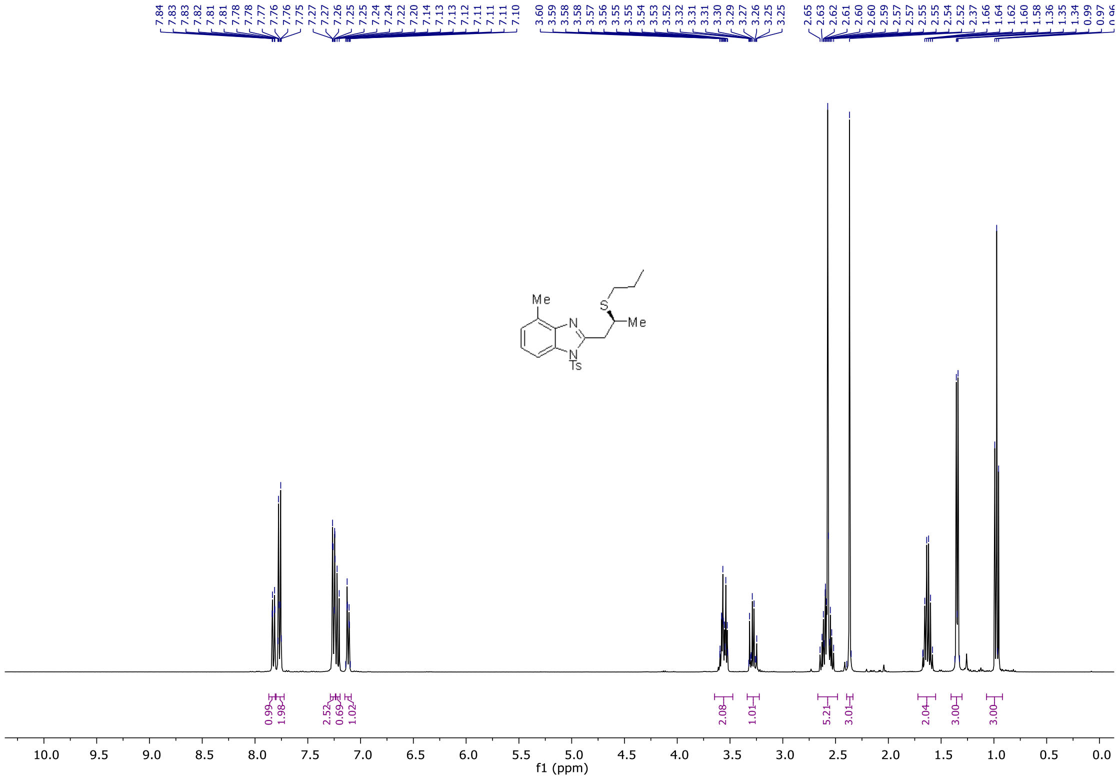

^{13}C NMR:

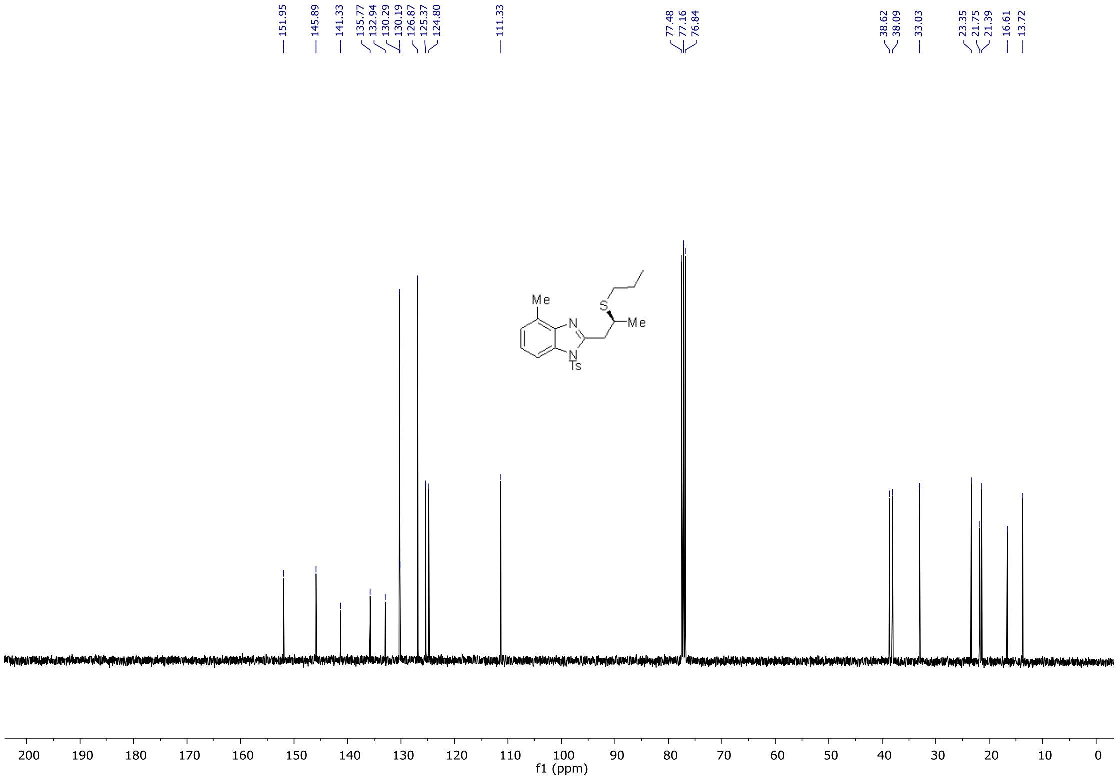

12/ HPLC traces

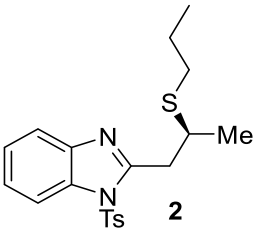

2

Racemic product

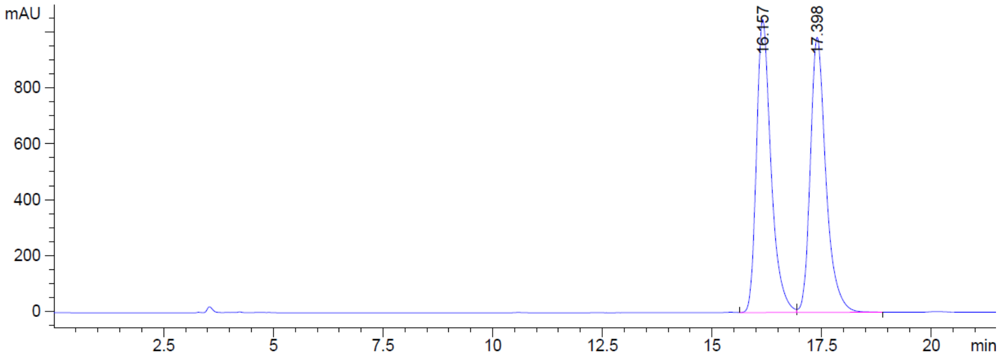

#	Time	Area	Height	Width	Area%
1	16.157	24223.5	1048.9	0.3494	49.832
2	17.398	24387.3	984.8	0.3769	50.168

Enantioenriched product

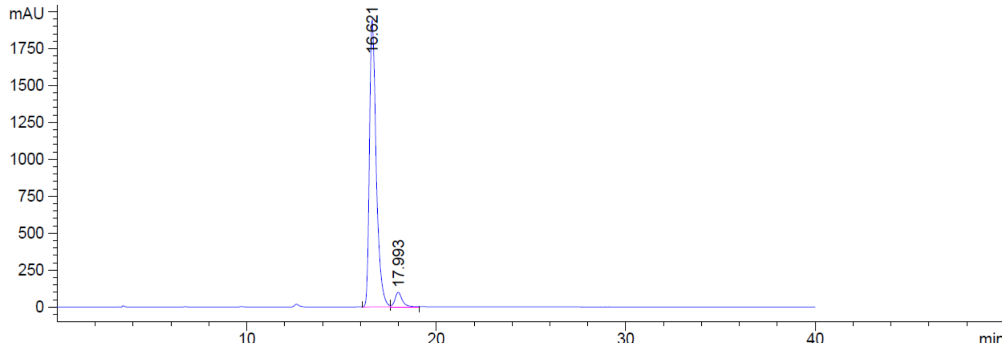

#	Time	Area	Height	Width	Area%
1	16.621	48256.6	1944.9	0.3755	94.870
2	17.993	2609.2	98.9	0.3998	5.130

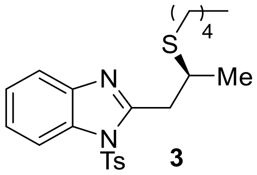

3

Racemic product

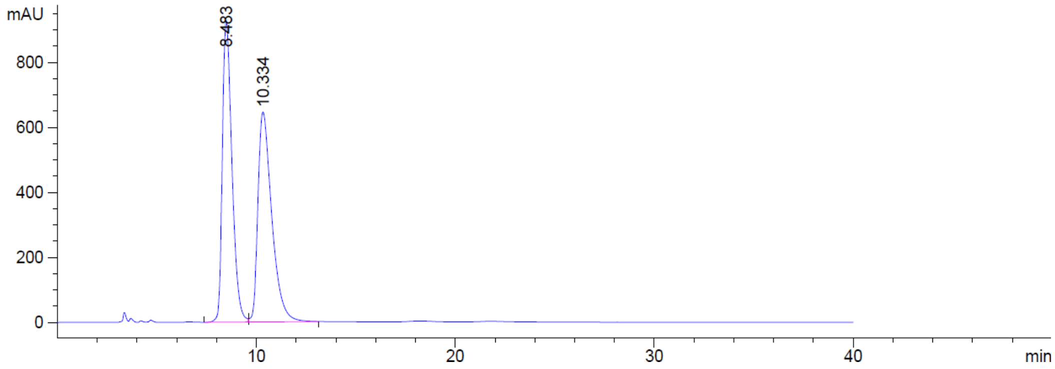

#	Time	Area	Height	Width	Area%
1	8.483	31084.0	927.7141	0.5149	49.864
2	10.334	31254.6	645.9370	0.7345	50.136

Enantioenriched product

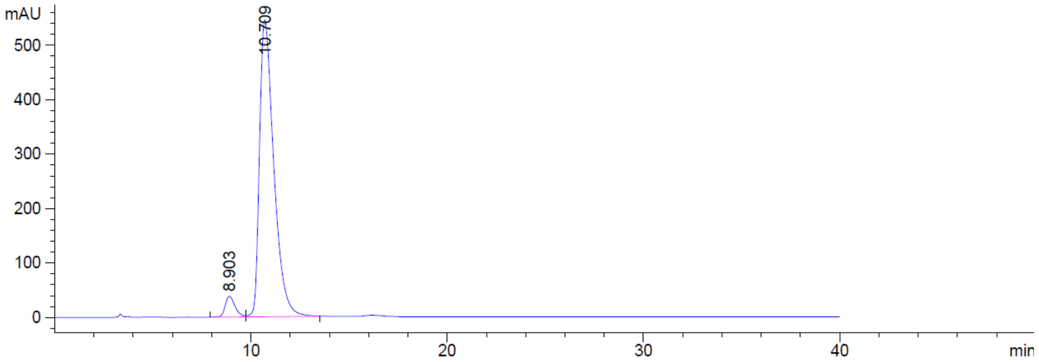

#	Time	Area	Height	Width	Area%
1	8.903	1339.9	38.2640	0.5366	4.636
2	10.709	27562.5	545.5766	0.7674	95.364

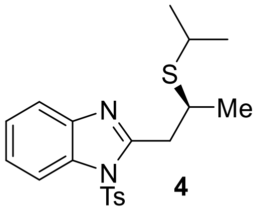

4

Racemic product

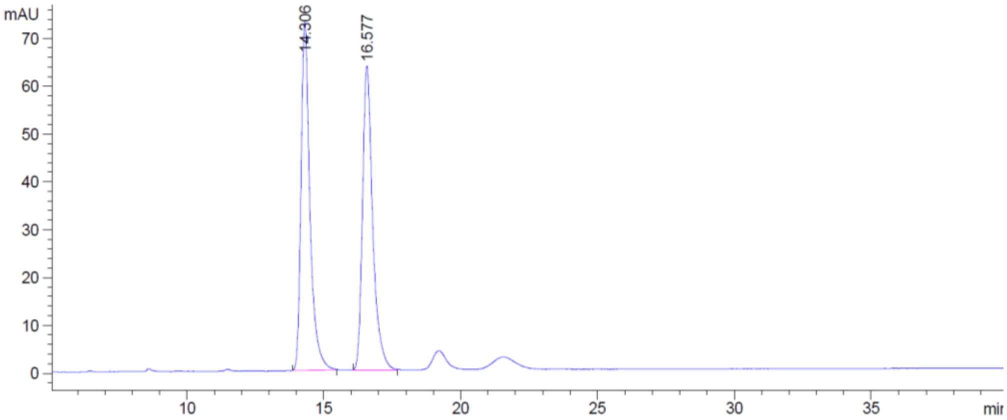

#	Time	Area	Height	Width	Area%
1	14.306	1649.7	72.7986	0.3384	50.052
2	16.577	1646.3	63.5925	0.3900	49.948

Enantioenriched product

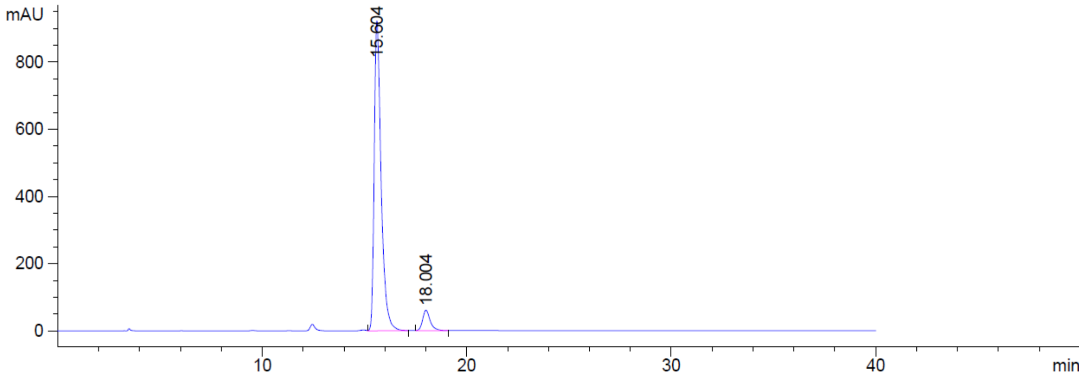

#	Time	Area	Height	Width	Area%
1	15.604	21620.1	922.3245	0.3535	93.202
2	18.004	1576.8	60.8959	0.3901	6.798

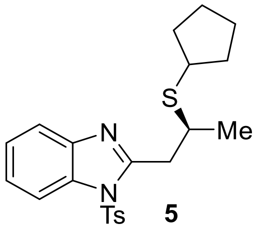

5

Racemic product

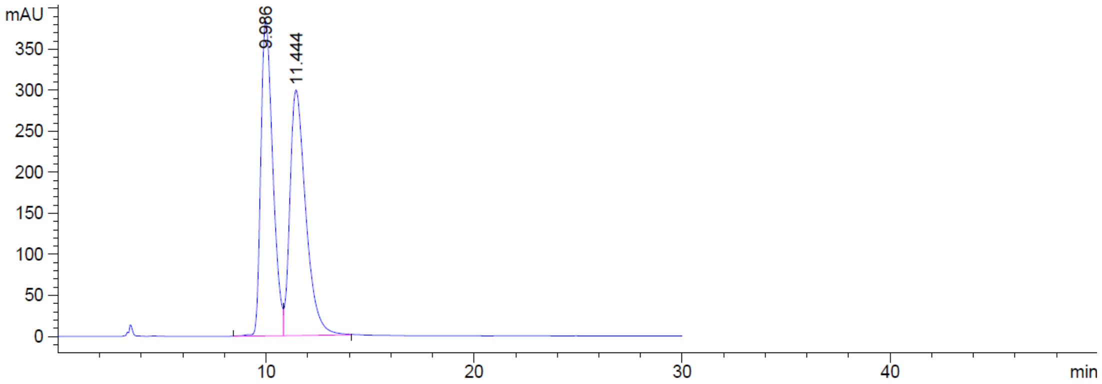

#	Time	Area	Height	Width	Area%
**1**	9.986	15351.7	383.4876	0.6190	49.182
**2**	11.444	15862.2	299.1960	0.8025	50.817

Enantioenriched product

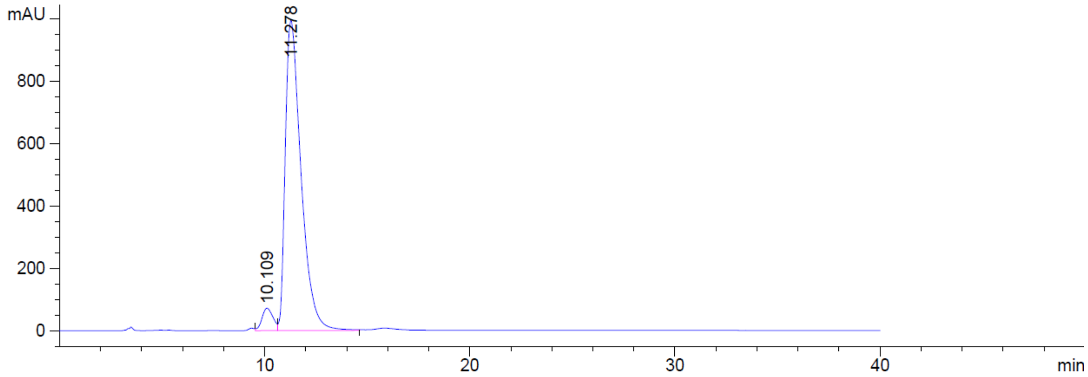

#	Time	Area	Height	Width	Area%
**1**	10.109	2658.4	71.6780	0.5827	4.764
**2**	11.278	53146.0	994.2653	0.8156	95.236

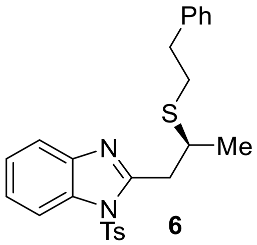

6

Racemic product

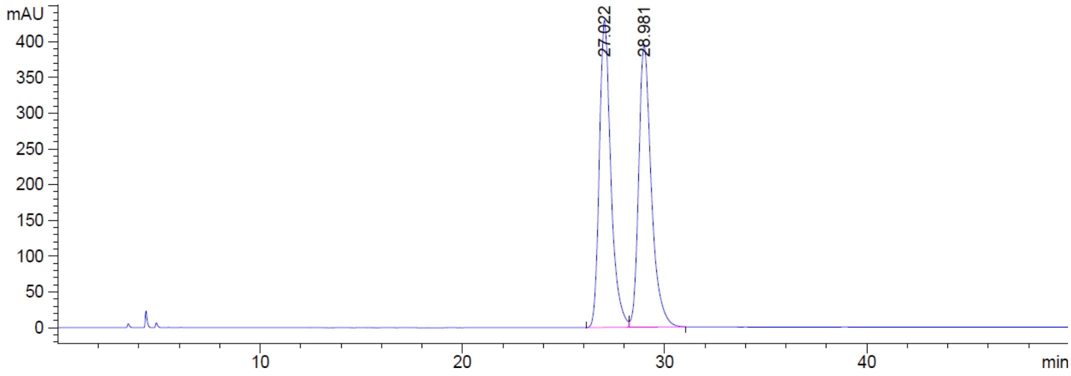

#	Time	Area	Height	Width	Area%
**1**	27.022	17032.2	429.5468	0.6026	49.755
**2**	28.981	17200.1	395.7534	0.6571	50.245

Enantioenriched product

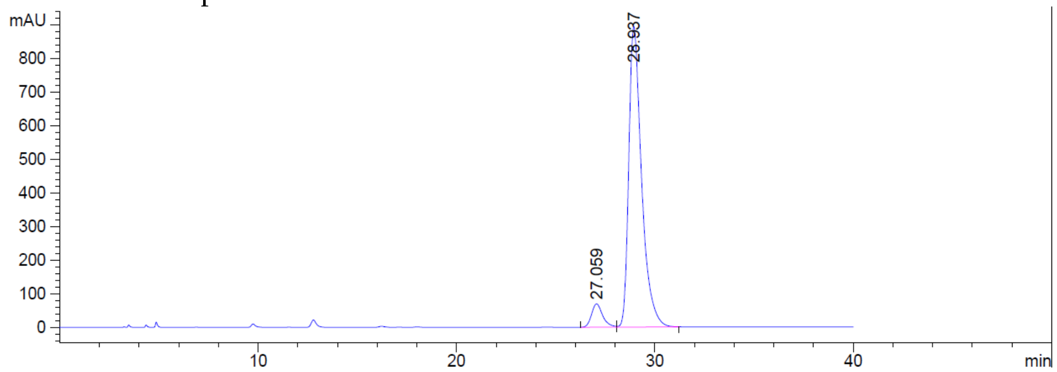

#	Time	Area	Height	Width	Area%
**1**	27.059	2723.8	69.5989	0.5926	6.351
**2**	28.937	40161.7	895.2585	0.6754	93.649

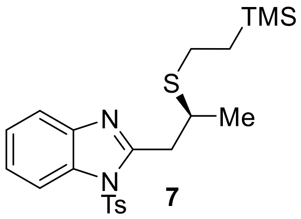

7

Racemic product

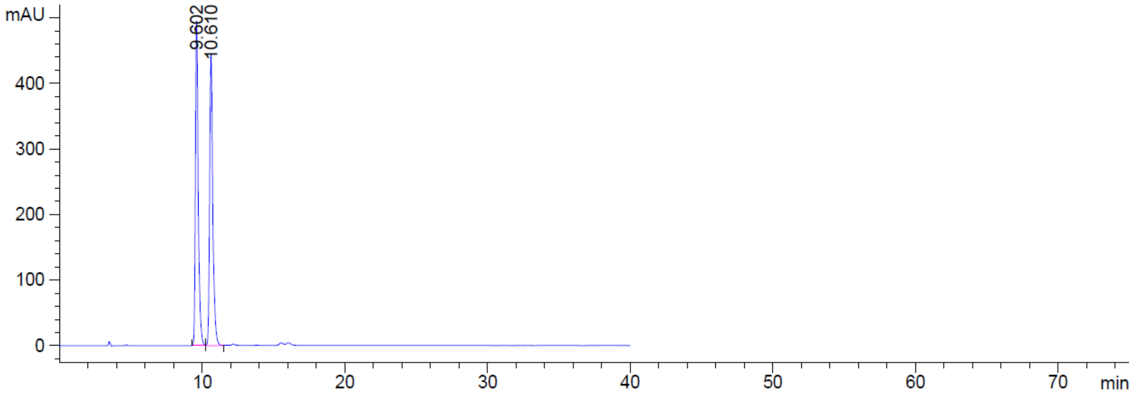

#	Time	Area	Height	Width	Area%
**1**	9.602	6850.8	495.3327	0.2074	49.918
**2**	10.610	6873.3	443.6841	0.2327	50.082

Enantioenriched product

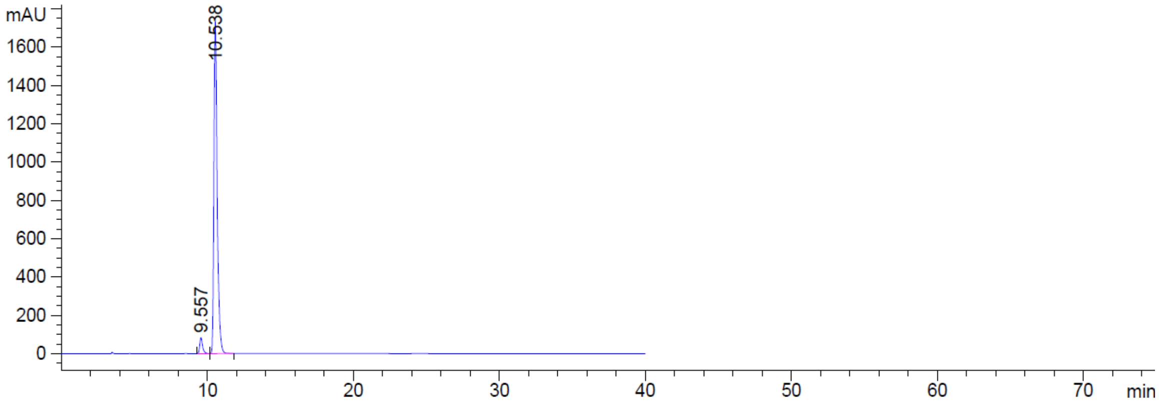

#	Time	Area	Height	Width	Area%
**1**	9.557	1134.6	83.2183	0.2051	4.002
**2**	10.538	27218.6	1733.9081	0.2371	95.998

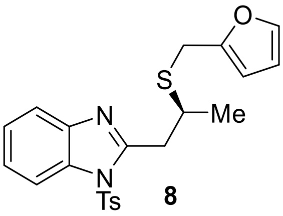

8

Racemic product

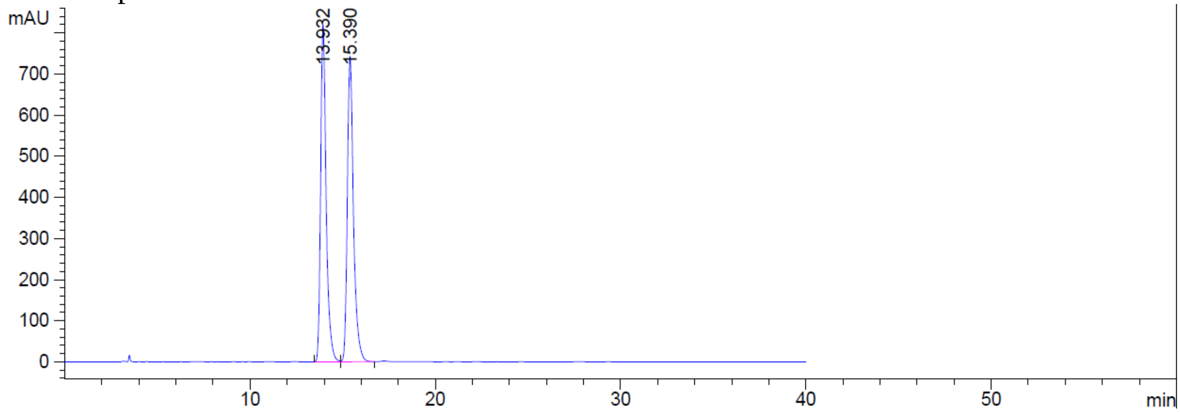

#	Time	Area	Height	Width	Area%
**1**	13.932	17002.3	818.9419	0.6141	49.852
**2**	15.390	17103.1	742.4780	0.6970	50.148

Enantioenriched product

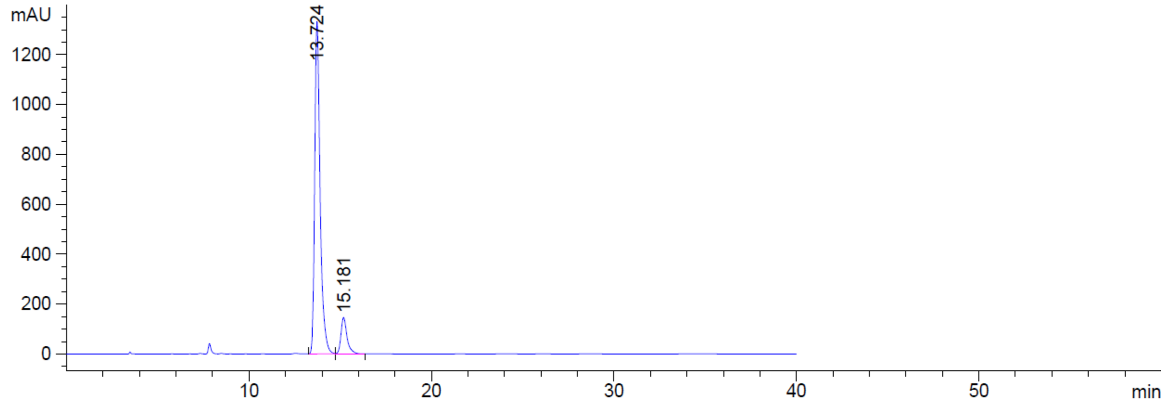

#	Time	Area	Height	Width	Area%
**1**	13.724	27589.1	818.9419	0.3115	89.106
**2**	15.181	3373.0	742.4780	0.3458	10.894

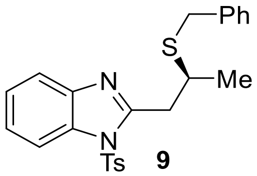

9

Racemic product

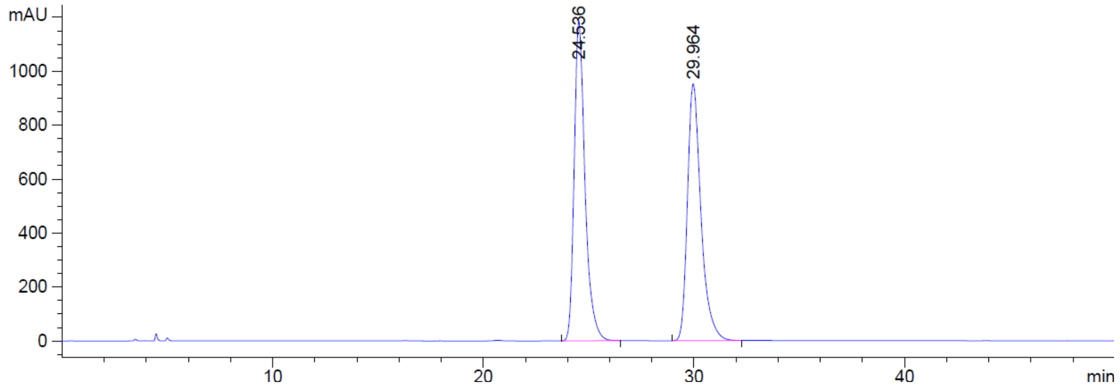

#	Time	Area	Height	Width	Area%
1	24.536	42477.2	1184.4417	0.5424	49.978
2	29.964	42515.1	952.5371	0.6727	50.022

Enantioenriched product

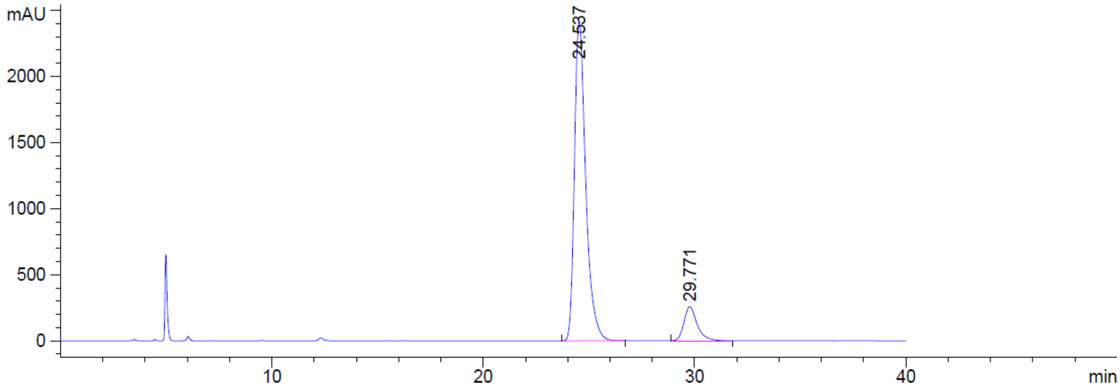

#	Time	Area	Height	Width	Area%
1	24.592	89767.9	2418.4070	0.5651	88.989
2	29.766	11107.6	257.3401	0.6516	11.011

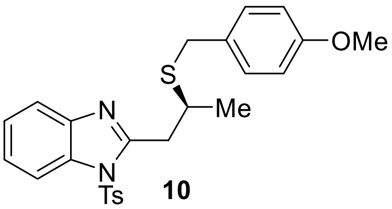

10

Racemic product

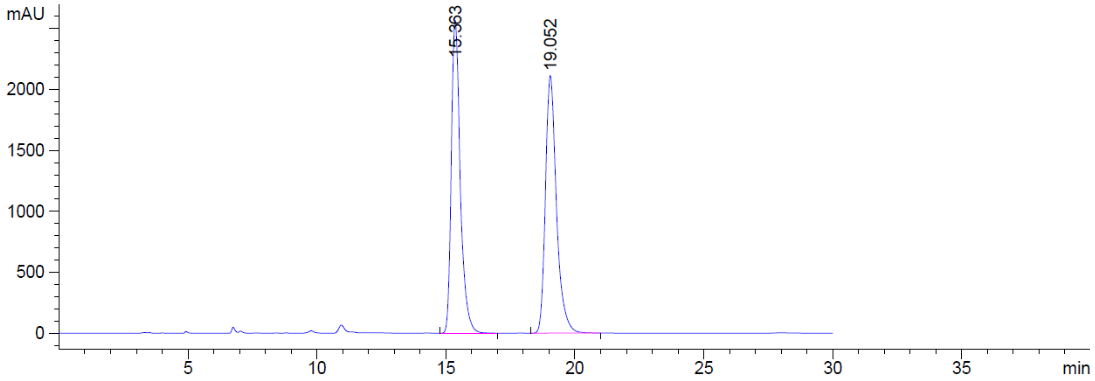

#	Time	Area	Height	Width	Area%
1	15.363	62104.3	2568.3023	0.3701	49.691
2	19.052	62877.0	2114.1353	0.4510	50.309

Enantioenriched product

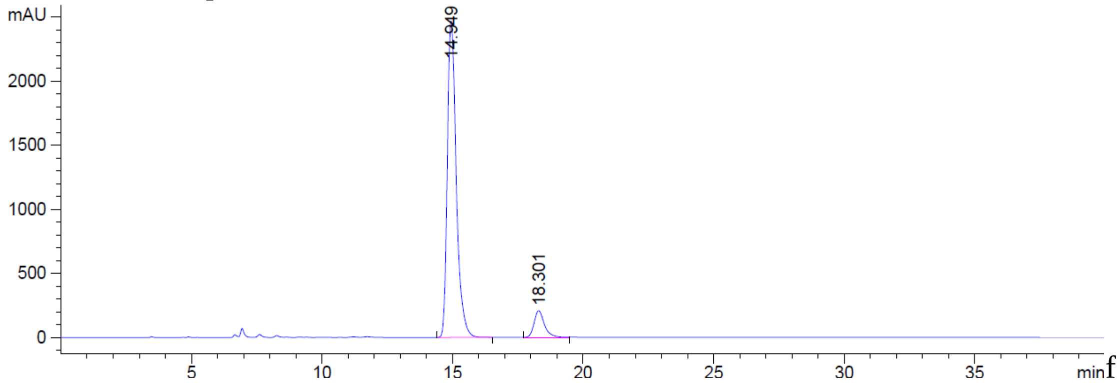

#	Time	Area	Height	Width	Area%
1	14.949	58773.1	2465.8980	0.3661	91.193
2	18.301	5676.0	207.8852	0.4145	8.807

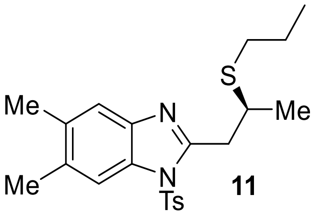

11

Racemic compound

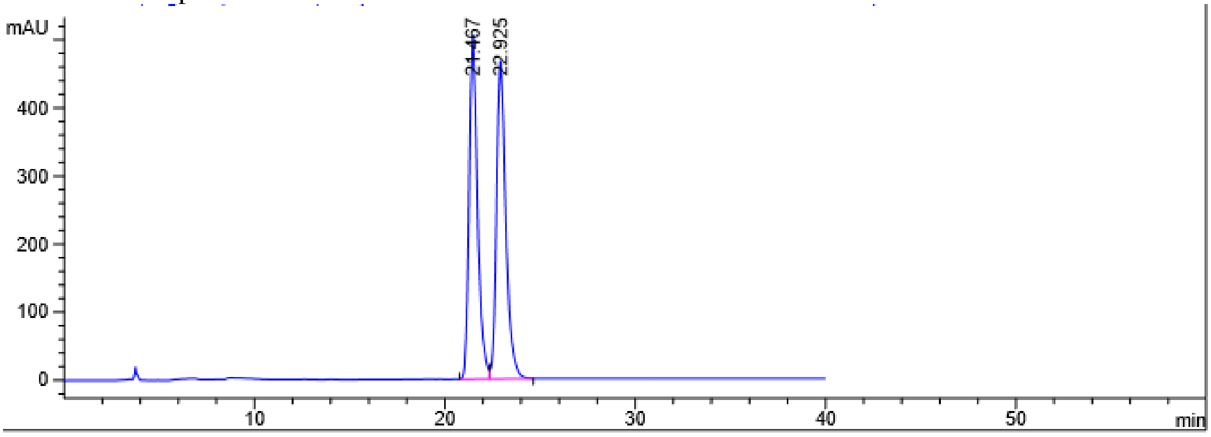

#	Time	Area	Height	Width	Area%
1	21.467	1.55473e4	505.28189	0.4670	49.6855
2	22.925	1.57441e4	466.74344	0.5095	50.3145

Enantioenriched compound

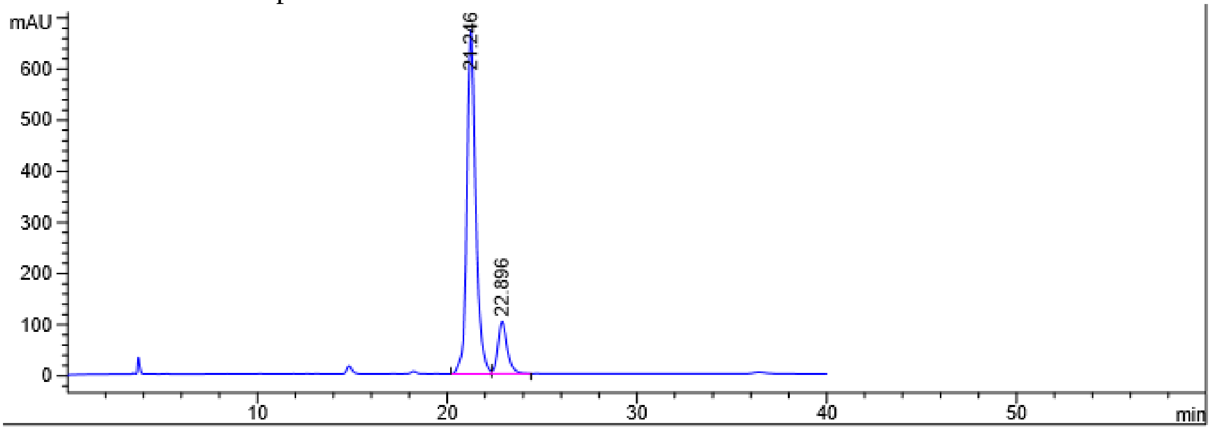

#	Time	Area	Height	Width	Area%
1	21.246	2.17454e4	673.19257	0.4849	86.2196
2	22.896	3475.55786	101.73867	0.5186	13.7804

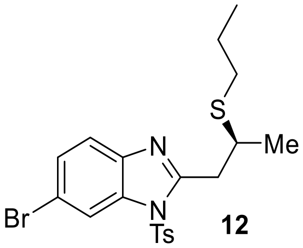

Racemic compound

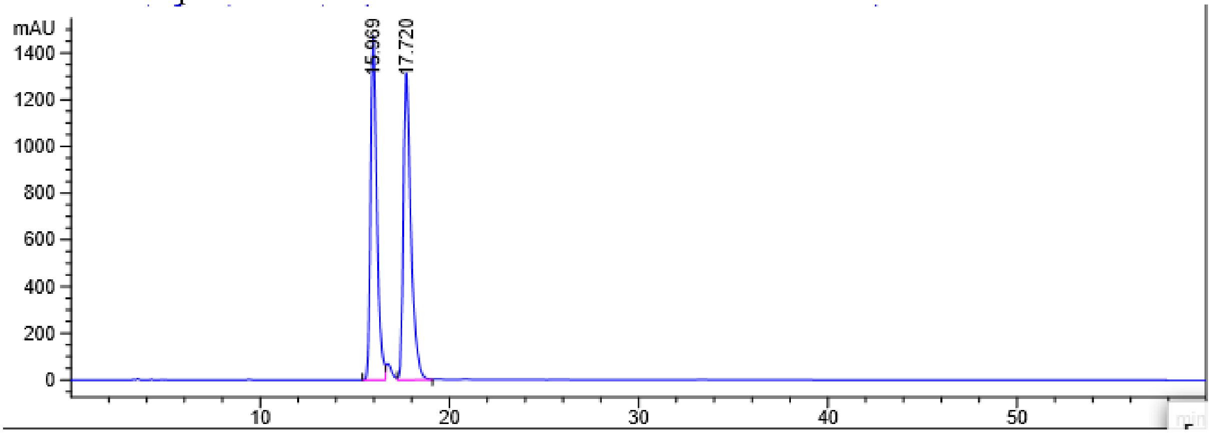

#	Time	Area	Height	Width	Area%
1	15.969	3.43880e4	1475.41064	0.3559	48.7907
2	17.720	3.60927e4	1313.82422	0.4085	51.2093

Enantioenriched compound

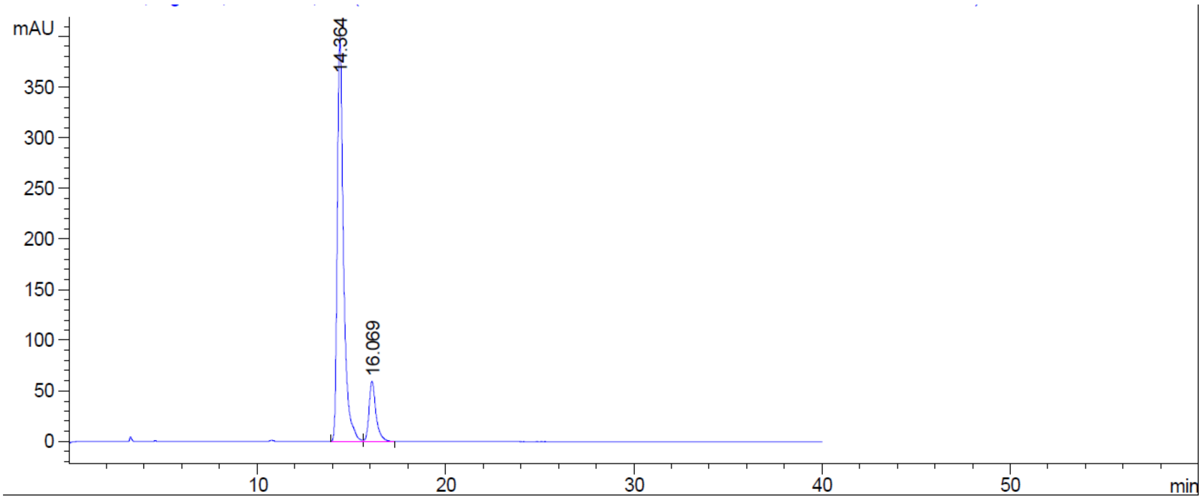

#	Time	Area	Height	Width	Area%
1	14.364	206.9619	399.40341	0.3449	85.8882
2	16.069	1512.7382	59.37542	0.3812	14.1118

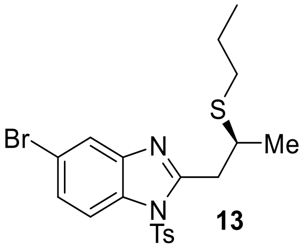

13

Racemic compound

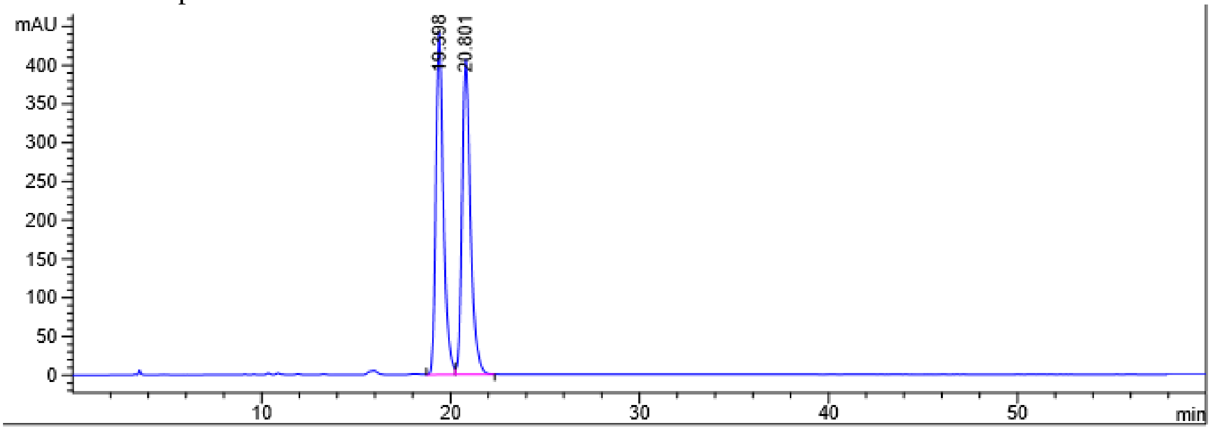

#	Time	Area	Height	Width	Area%
1	19.398	1.24443e4	442.41595	0.4282	49.7842
2	20.801	1.25521e4	407.14053	0.4637	50.2158

Enantioenriched compound

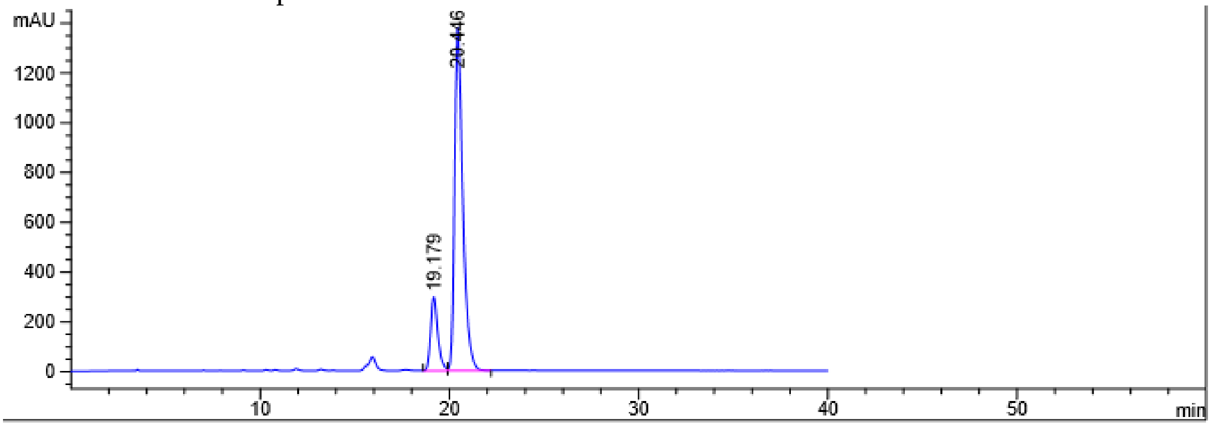

#	Time	Area	Height	Width	Area%
1	19.179	7845.54053	296.33978	0.4009	15.8078
2	20.446	4.17853e4	1379.10303	0.4575	84.1922

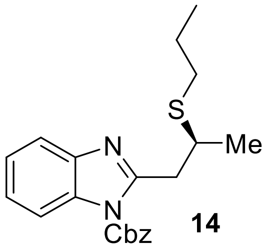

14

Racemic product

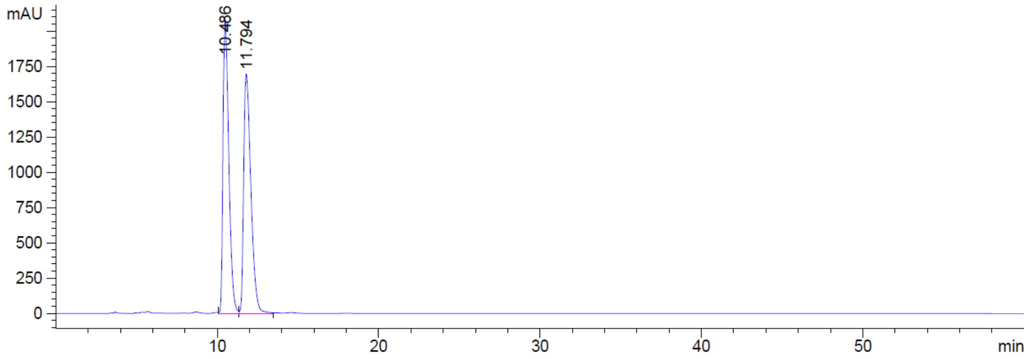

#	Time	Area	Height	Width	Area%
1	10.486	52056.1	2079.6	0.3880	49.664
2	11.794	52759.6	1696.7	0.4808	50.336

Enantioenriched Product

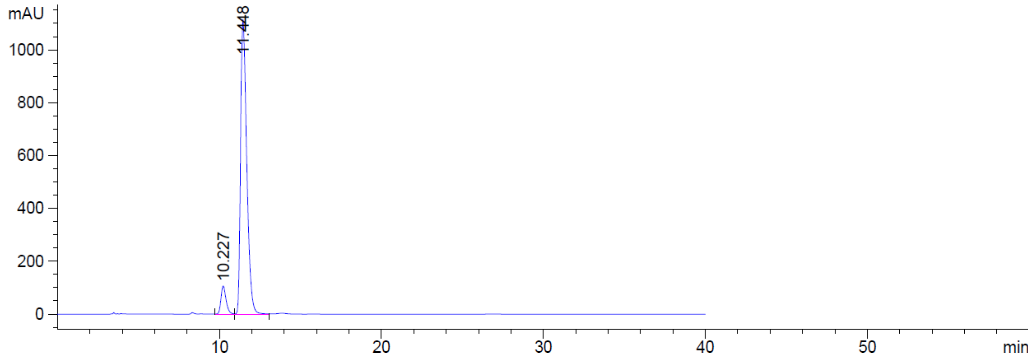

#	Time	Area	Height	Width	Area%
1	10.227	2339.1	106.8	0.3335	7.338
2	11.448	29543.4	1114.6	0.4808	92.662

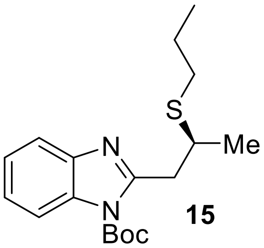

15

Racemic product

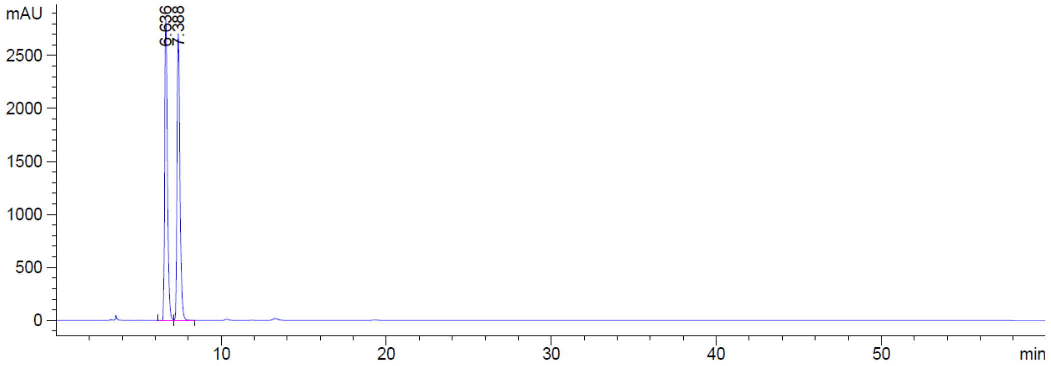

#	Time	Area	Height	Width	Area%
1	6.636	32821.6	2836.5	0.1790	49.600
2	7.388	33350.9	2705.3	0.1898	50.400

Enantioenriched Product

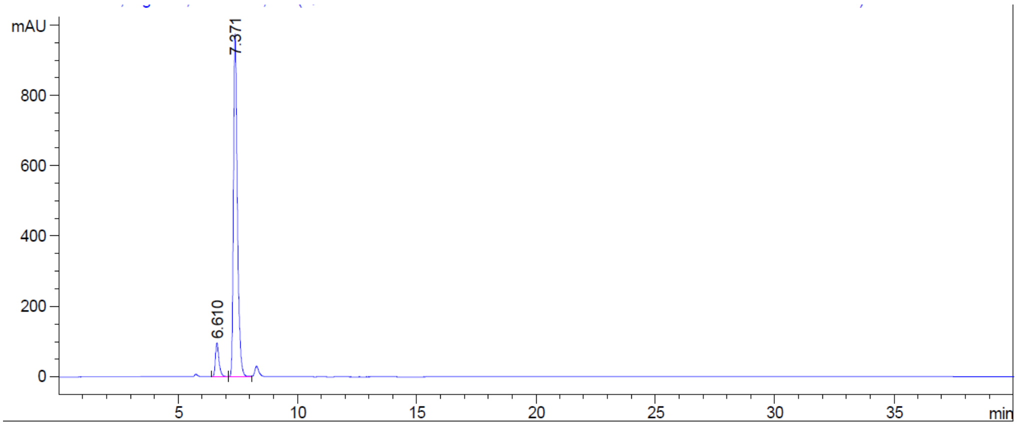

#	Time	Area	Height	Width	Area%
1	6.610	1767.4	168.3	0.1585	8.295
2	7.371	19538.6	1699.8	0.1741	91.705

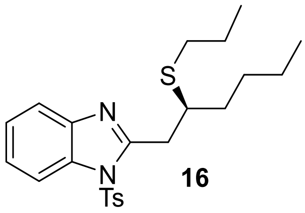

16

Racemic product

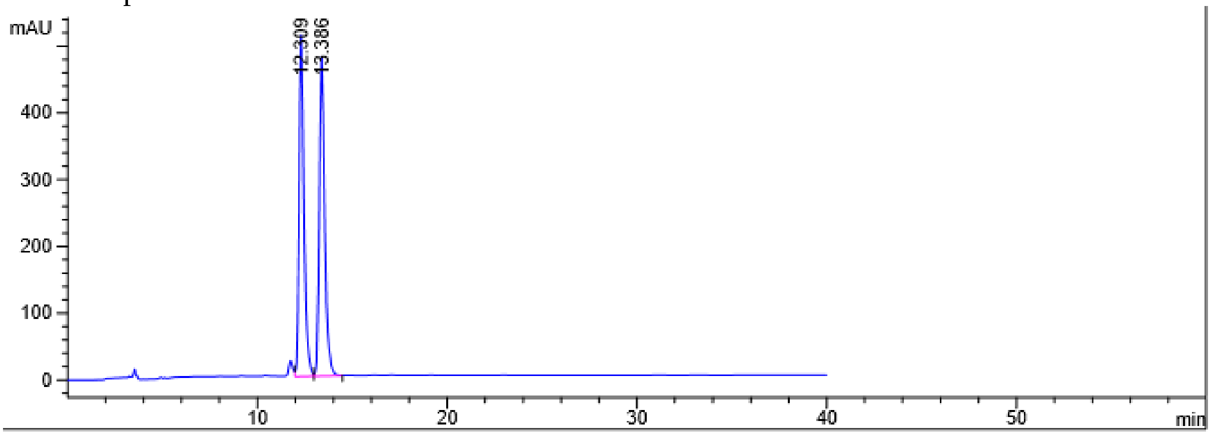

#	Time	Area	Height	Width	Area%
**1**	12.309	8994.98633	510.45645	0.2656	49.3848
**2**	13.386	9219.11035	478.34451	0.2909	50.6152

Enantioenriched product

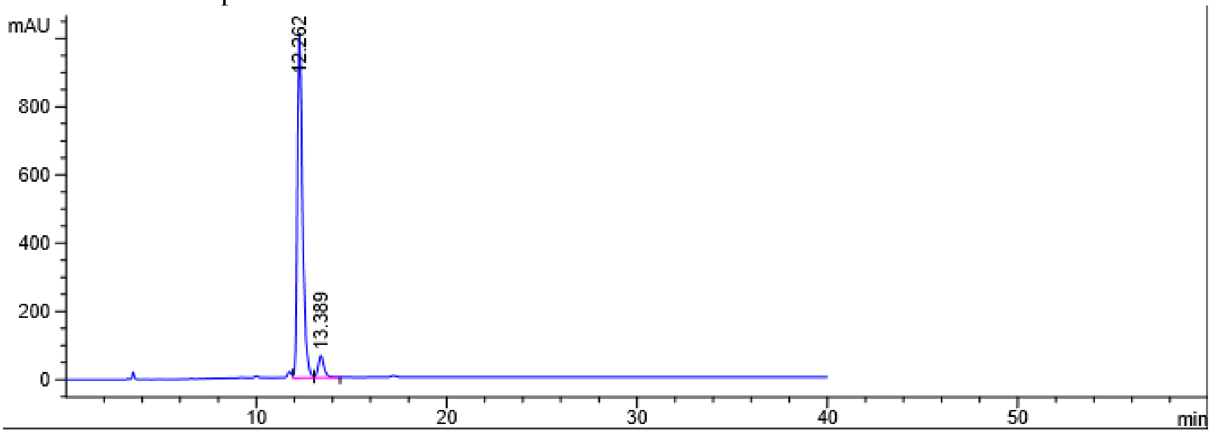

#	Time	Area	Height	Width	Area%
**1**	12.262	1.87781e4	1011.44312	0.2786	93.1804
**2**	13.389	1374.31885	64.74799	0.3156	6.8196

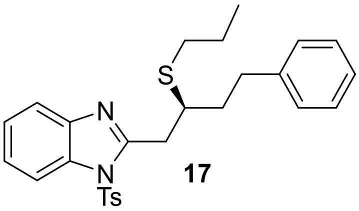

17

Racemic Product

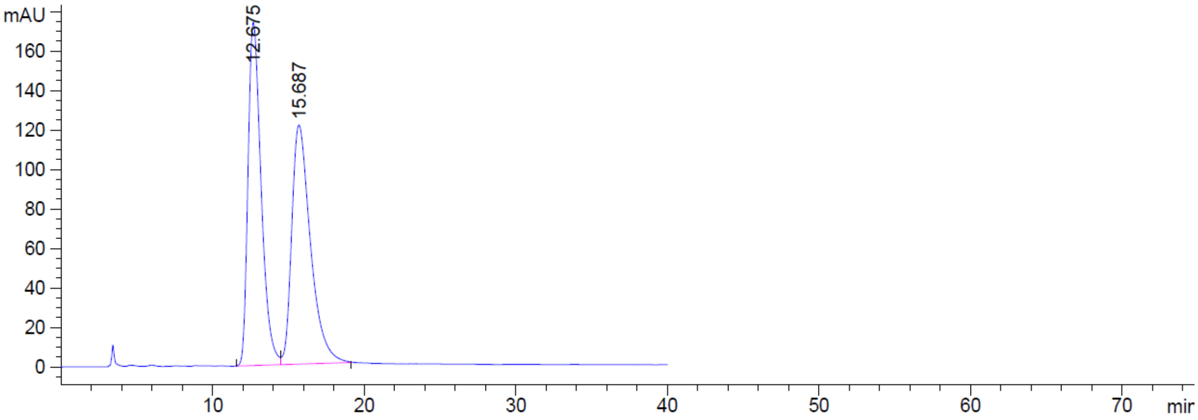

#	Time	Area	Height	Width	Area%
1	12.675	10457.9	173.8140	0.9179	50.063
2	15.687	10431.5	121.1182	1.3028	49.937

Enantioenriched product

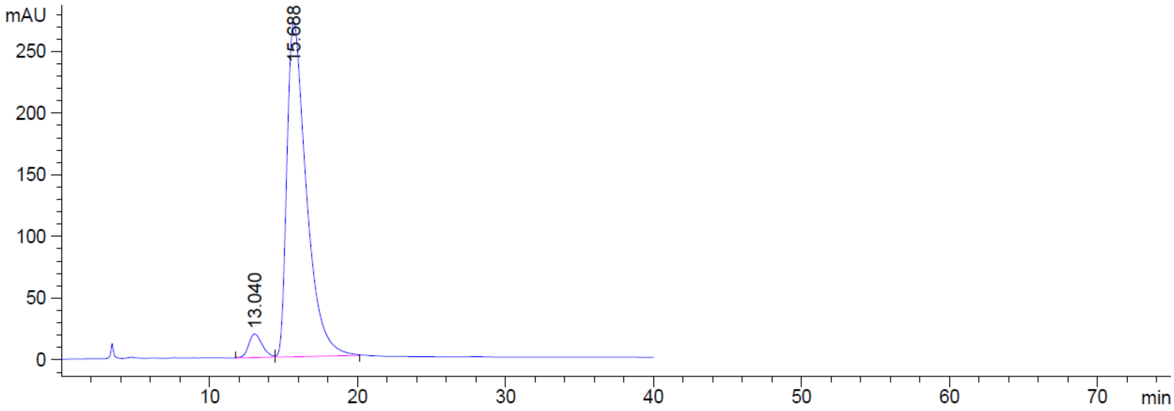

#	Time	Area	Height	Width	Area%
1	13.040	1212.1	19.0291	0.9811	4.666
2	15.688	24767.7	271.1863	1.3695	95.334

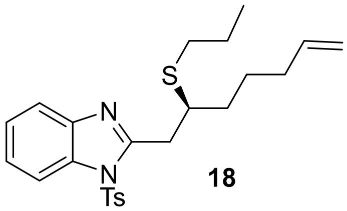

18

Racemic compound

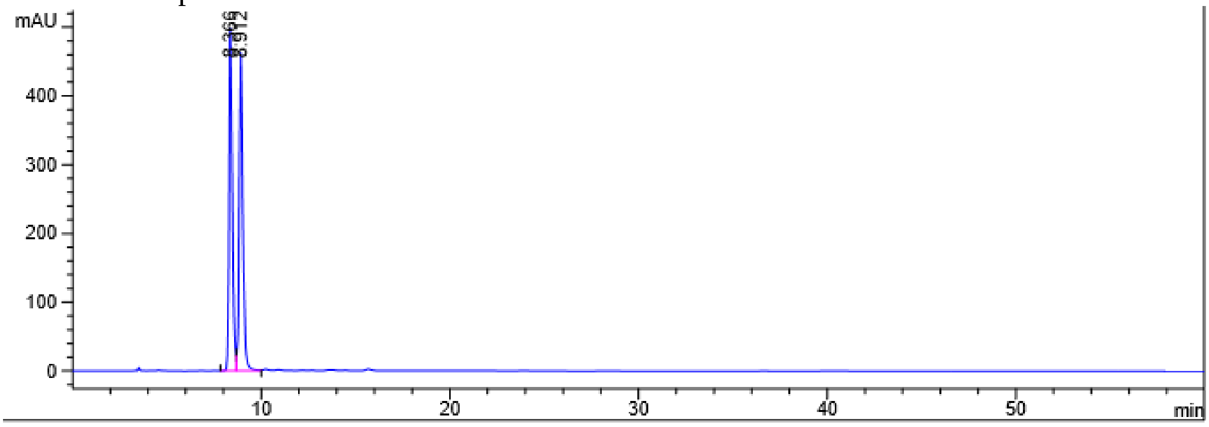

#	Time	Area	Height	Width	Area%
1	8.366	6081.06592	499.22632	0.1821	48.9965
2	8.912	6330.15381	463.47977	0.2033	51.0035

Enantioenriched compound

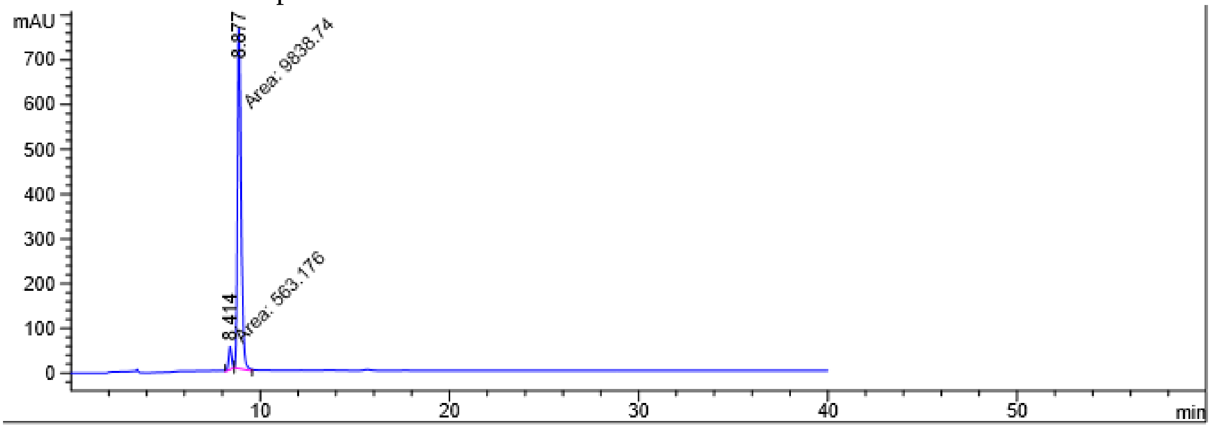

#	Time	Area	Height	Width	Area%
1	8.414	563.17572	51.41105	0.1826	5.4142
2	8.877	9838.74414	760.34290	0.2157	94.5858

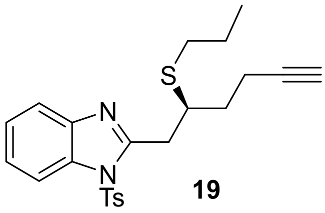

19

Racemic compound

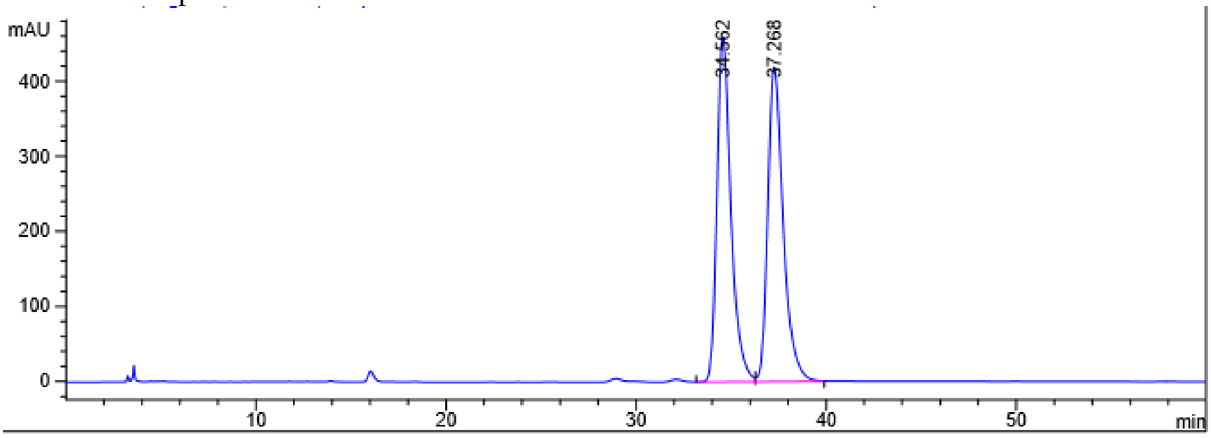

#	Time	Area	Height	Width	Area%
1	34.562	2.34292e4	459.58228	0.7708	49.8764
2	37.268	2.35453e4	418.68997	0.8561	50.1236

Enantioenriched product

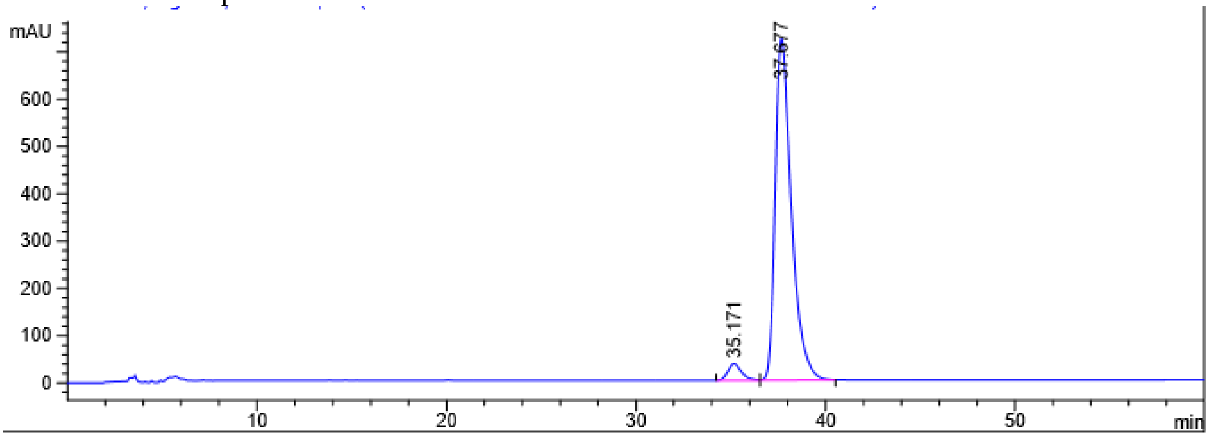

#	Time	Area	Height	Width	Area%
1	35.171	1776.26453	35.18375	0.7650	3.9900
2	37.677	4.27417e4	722.38684	0.8943	96.0100

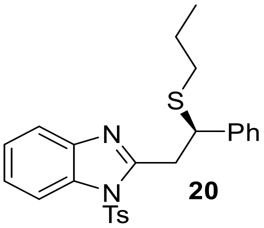

20

Racemic product

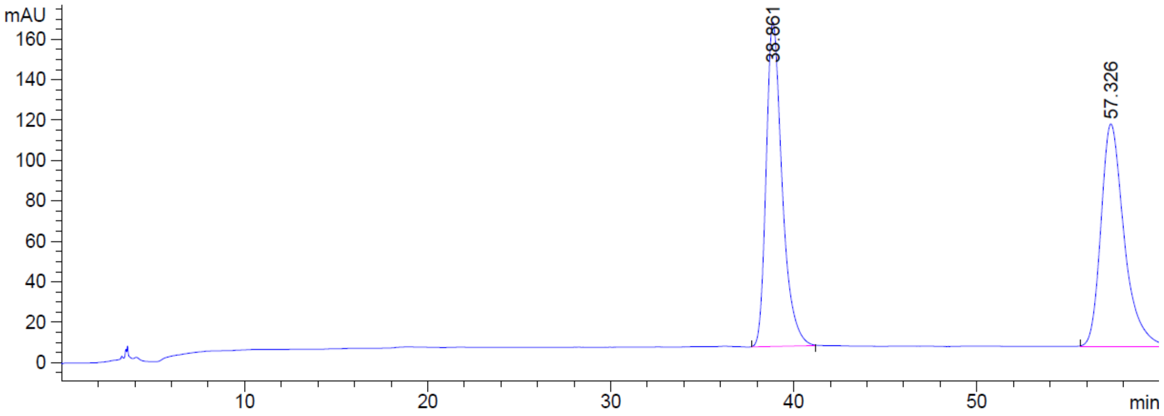

#	Time	Area	Height	Width	Area%
1	38.861	9754.6	160.4723	0.9252	50.252
2	57.326	9656.7	110.0178	1.3280	49.748

Enantioenriched product

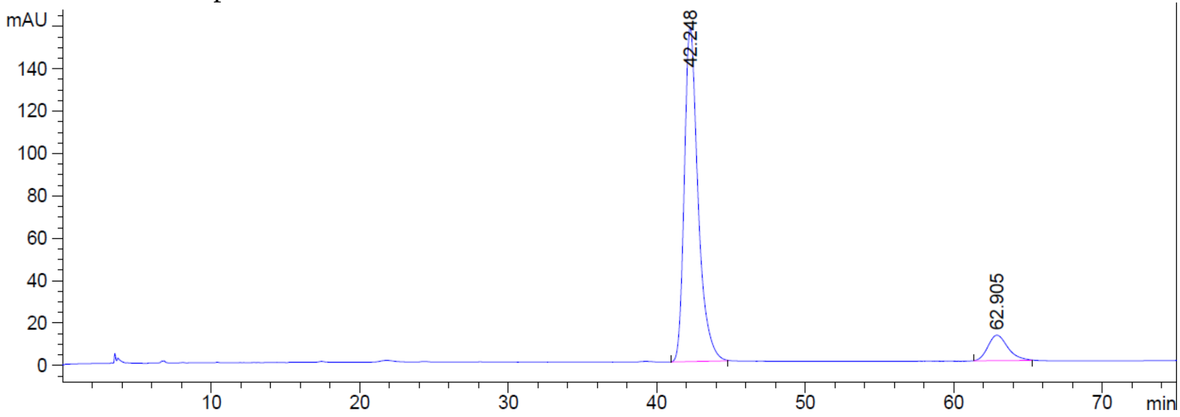

#	Time	Area	Height	Width	Area%
1	42.248	10133.4	158.0832	0.9679	89.967
2	62.905	1130.0	12.0323	1.3577	10.032

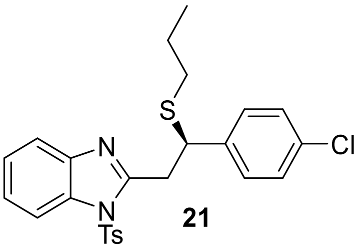

21

Racemic product

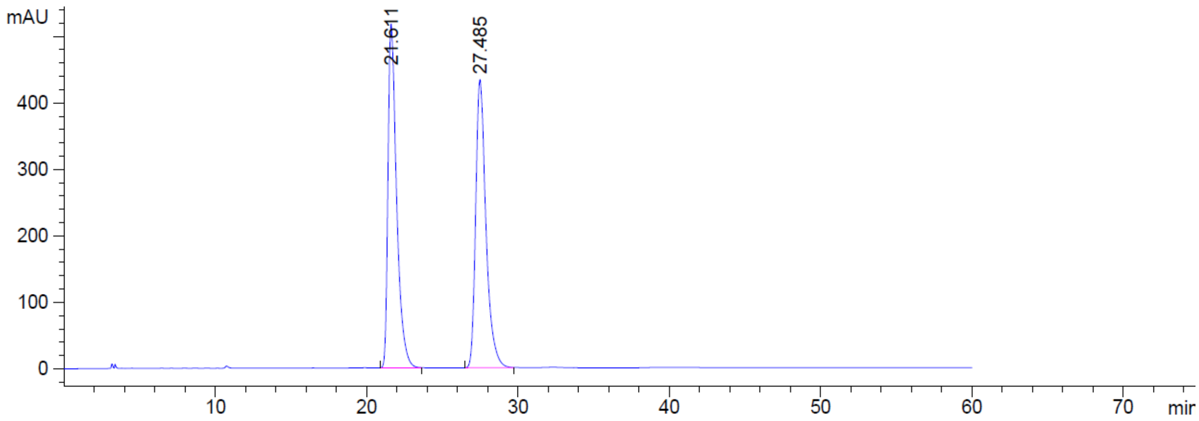

#	Time	Area	Height	Width	Area%
**1**	21.611	20460.5	517.2202	0.5955	50.037
**2**	27.485	20429.9	433.5056	0.7118	49.963

Enantioenriched product

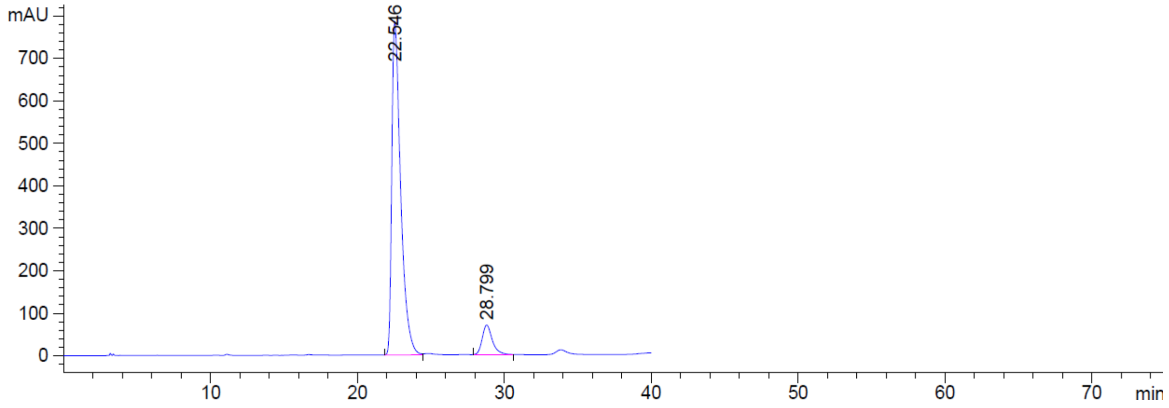

#	Time	Area	Height	Width	Area%
**1**	22.546	32986.8	785.3682	0.6300	90.856
**2**	28.799	3319.2	69.5783	0.7225	9.142

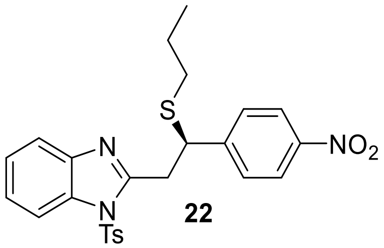

22

Racemic product

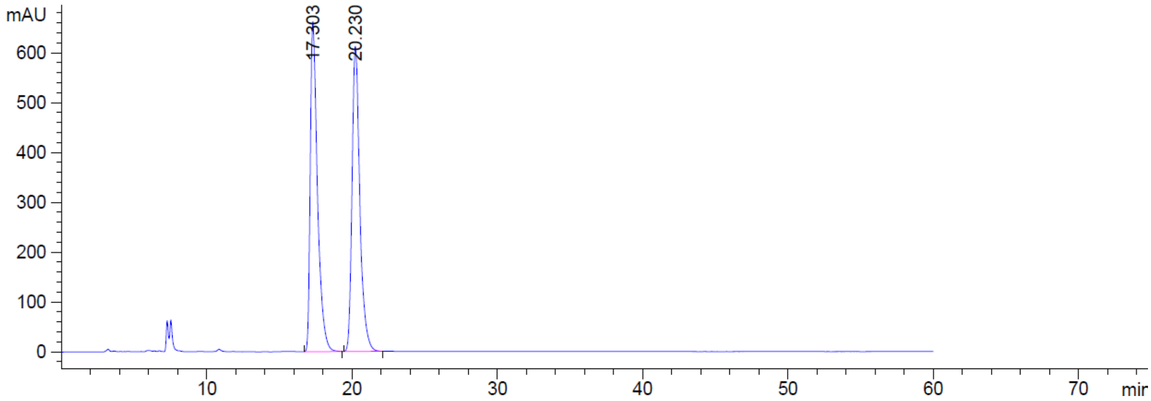

#	Time	Area	Height	Width	Area%
**1**	17.303	22852.9	662.3140	0.5166	50.125
**2**	20.230	22738.7	612.1039	0.5574	49.875

Enantioenriched product

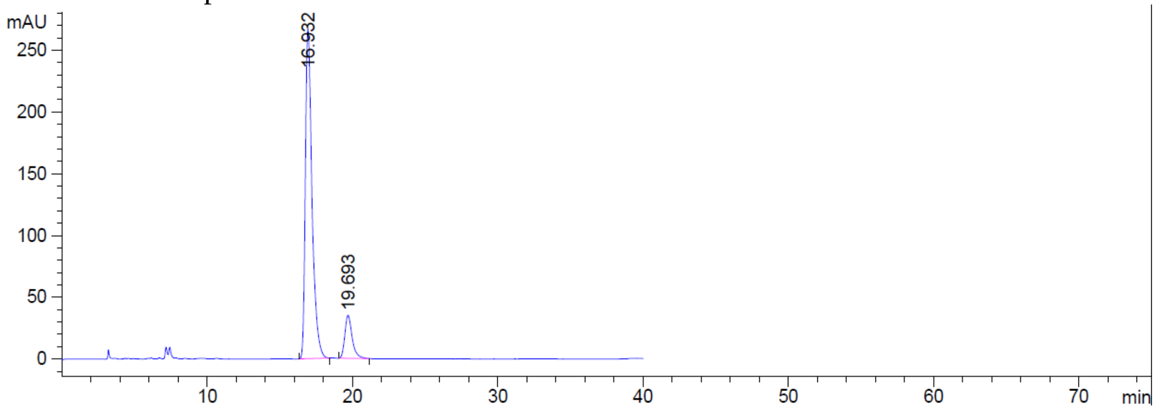

#	Time	Area	Height	Width	Area%
**1**	16.932	8521.5	267.1431	0.4821	87.486
**2**	19.693	1218.9	34.8653	0.5299	12.514

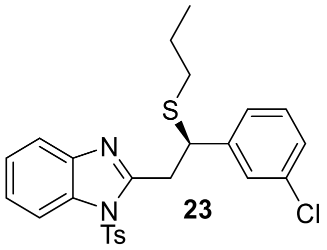

23

Racemic product

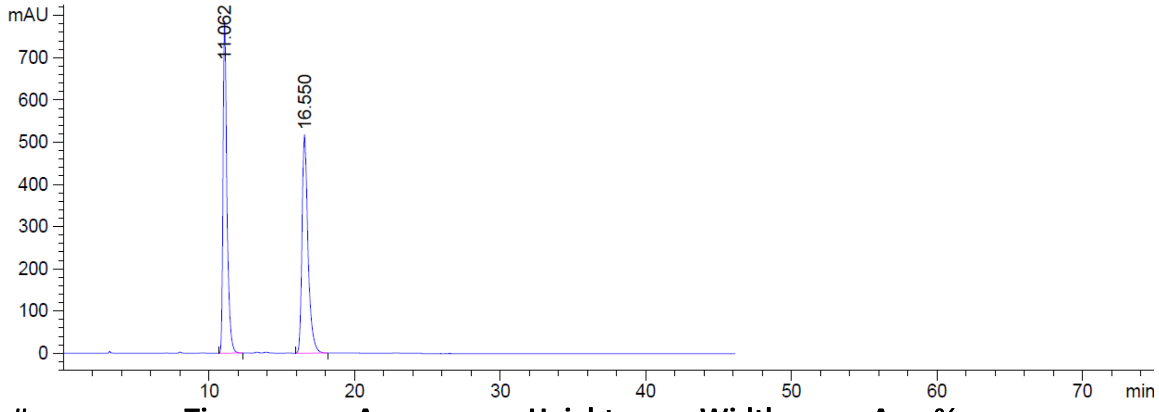

#	Time	Area	Height	Width	Area%
1	11.062	14813.3	786.5033	0.2837	49.895
2	16.550	14875.8	517.1082	0.4356	50.105

Enantioenriched product

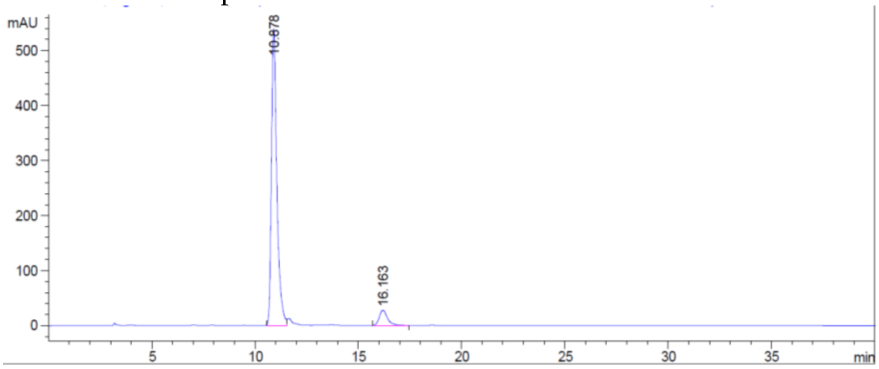

#	Time	Area	Height	Width	Area%
1	10.878	9806.3	537.7	0.2768	92.8014
2	16.163	760.7	27.9	0.4142	7.1986

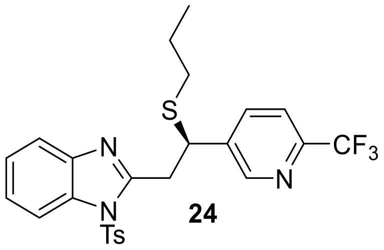

24

Racemic product

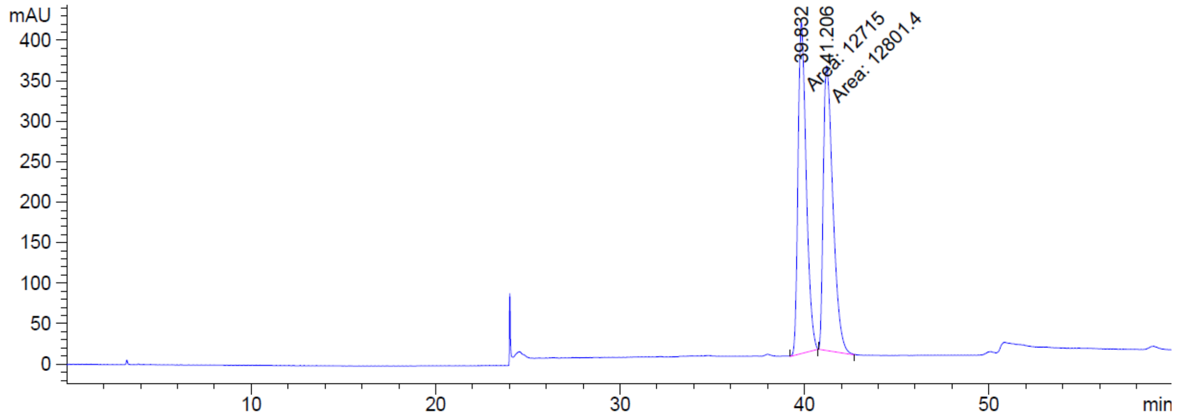

#	Time	Area	Height	Width	Area%
1	39.832	12715.0	408.8265	0.5184	49.831
2	41.206	12801.4	351.6669	0.6067	50.169

Enantioenriched product

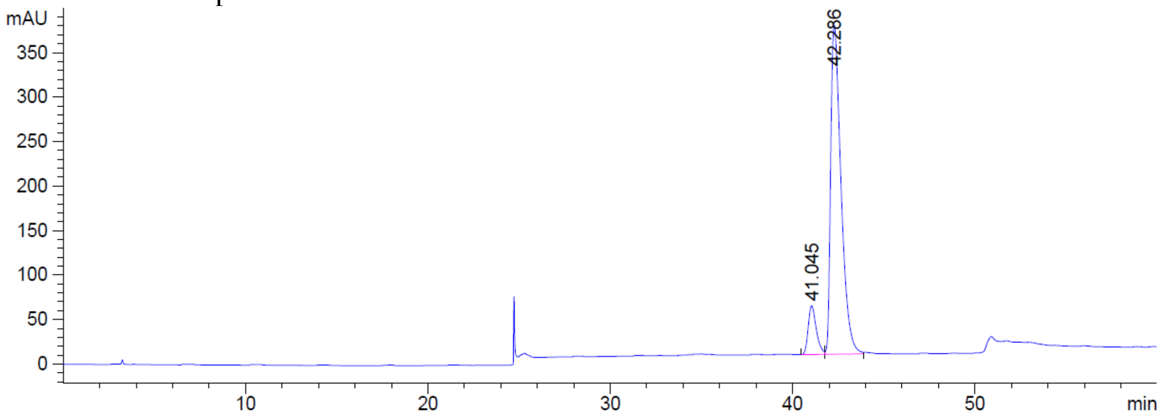

#	Time	Area	Height	Width	Area%
1	41.045	1692.3	54.7341	0.4767	10.432
2	42.286	14529.7	369.5265	0.5987	89.568

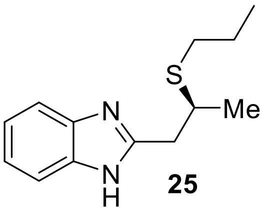

25

Racemic compound

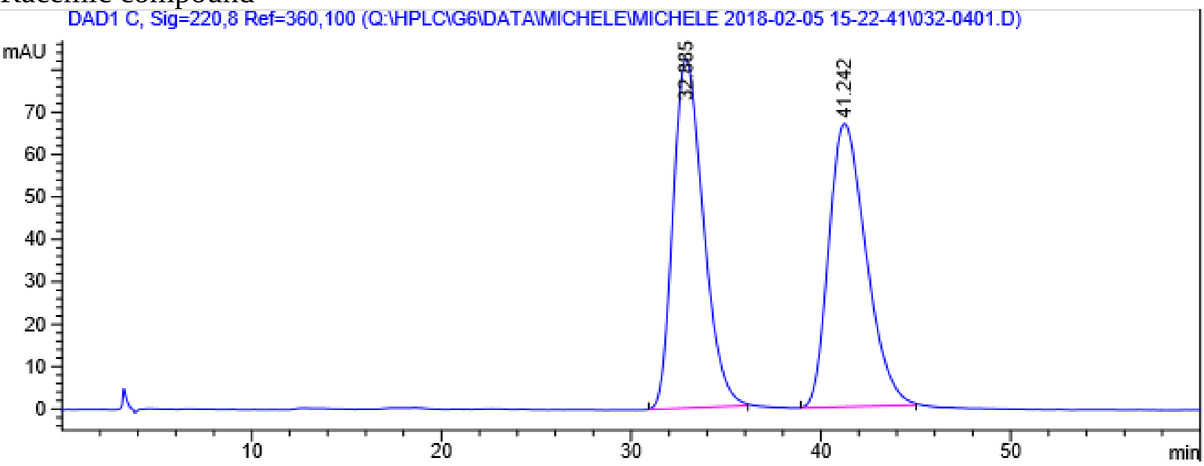

#	Time	Area	Height	Width	Area%
1	32.885	9137.54688	82.40344	1.6617	50.0216
2	41.242	9129.66797	66.75183	2.0777	49.9784

Enantioenriched compound

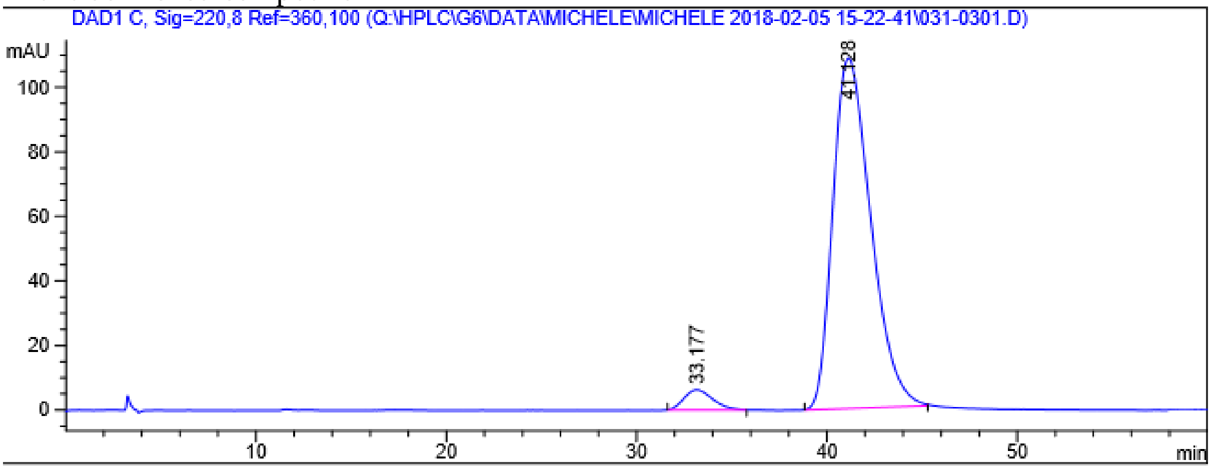

#	Time	Area	Height	Width	Area%
1	33.177	668.35852	6.28705	1.2762	4.2485
2	41.128	1.50633e4	108.59649	2.0903	95.7515

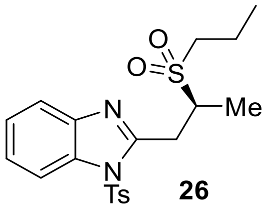

26

Racemic compound

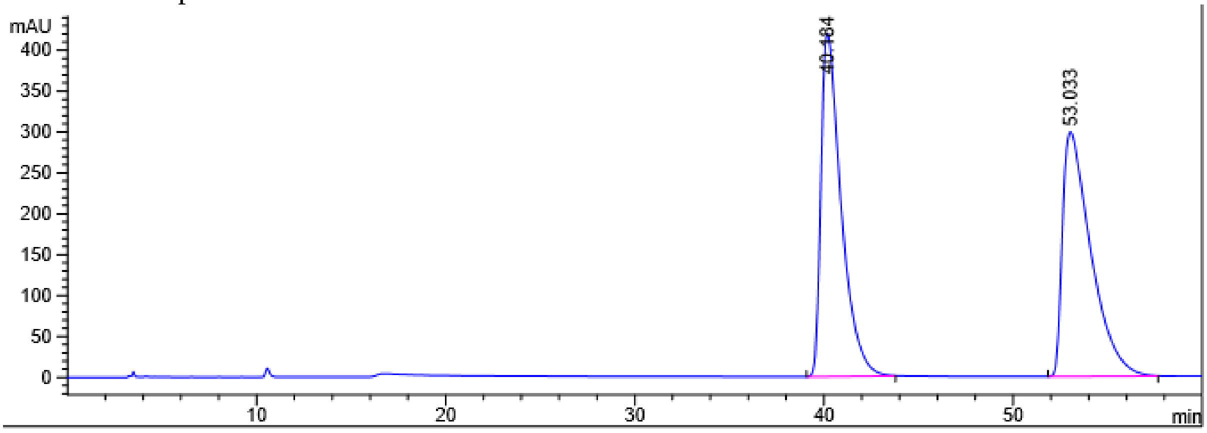

#	Time	Area	Height	Width	Area%
**1**	40.184	3.11824e4	419.67453	1.1108	49.9185
**2**	53.033	3.12842e4	299.08618	1.5523	50.0815

Enantioenriched compound

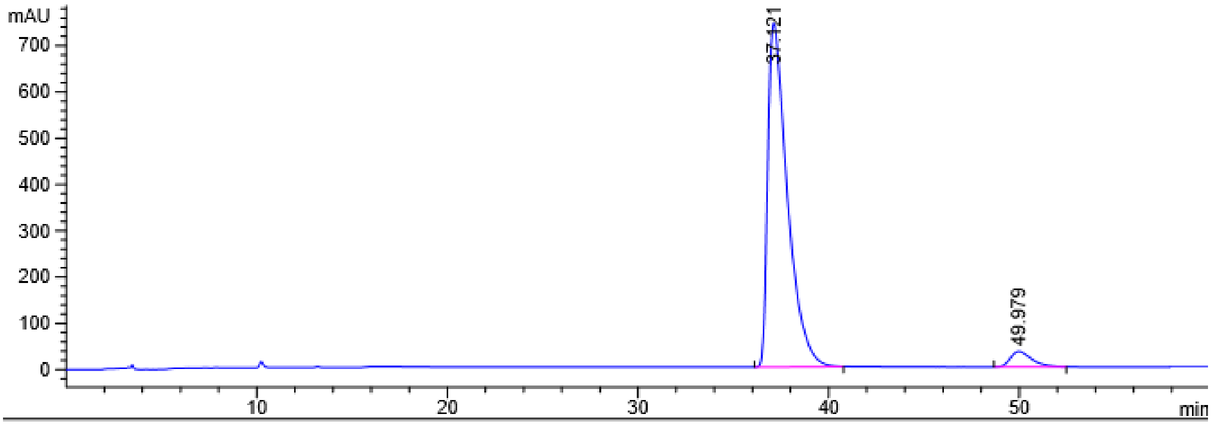

#	Time	Area	Height	Width	Area%
**1**	37.121	5.29723e4	743.85822	1.0648	95.1836
**2**	49.979	2680.45581	33.77411	1.1779	4.8164

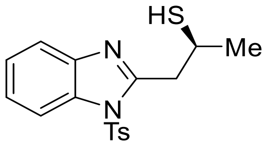

27

Racemic compound

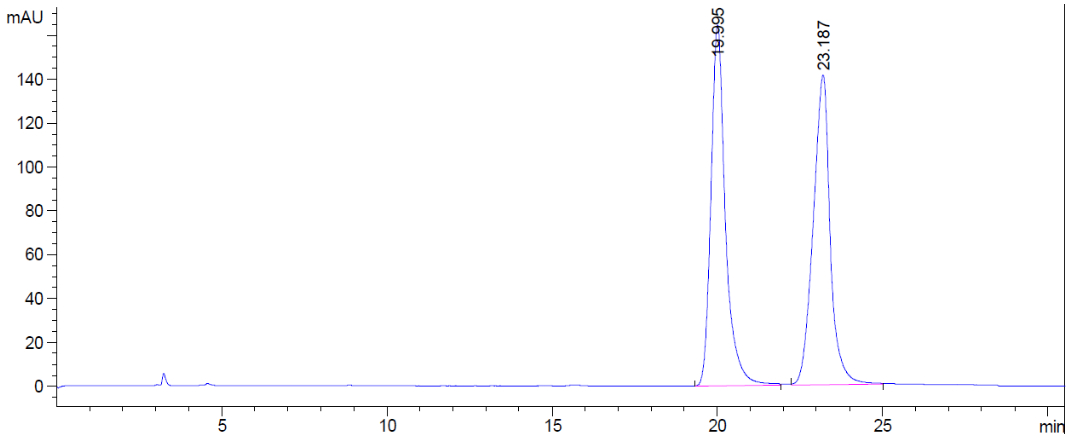

#	Time	Area	Height	Width	Area%
**1**	19.995	5018.7	164.5	0.4560	49.8617
**2**	23.187	5046.6	141.3	0.5328	50.1383

Enantioenriched compound

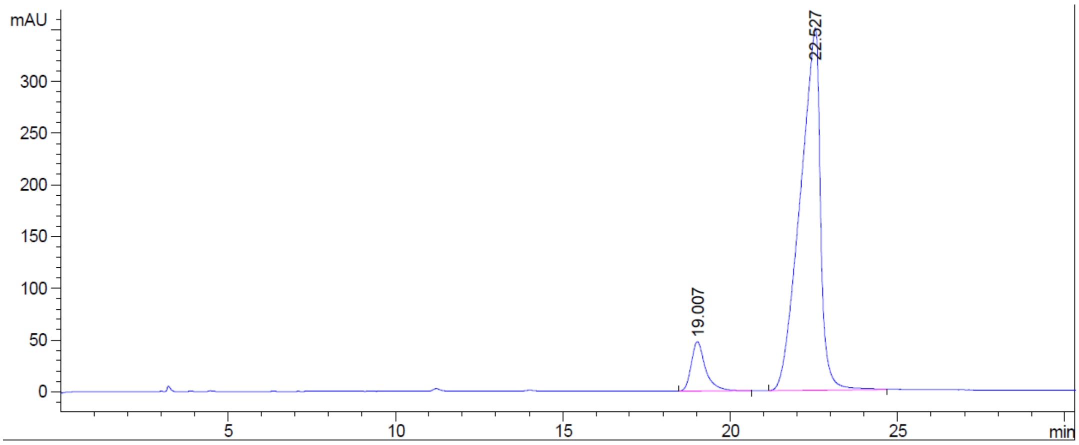

#	Time	Area	Height	Width	Area%
**1**	19.007	1415.6	47.77	0.4417	8.755
**2**	22.527	14753.6	350.1	0.6157	91.245

13/ Single Crystal X-Ray Diffraction Data

X-Ray structure data for compound **25**

CCDC:1833189

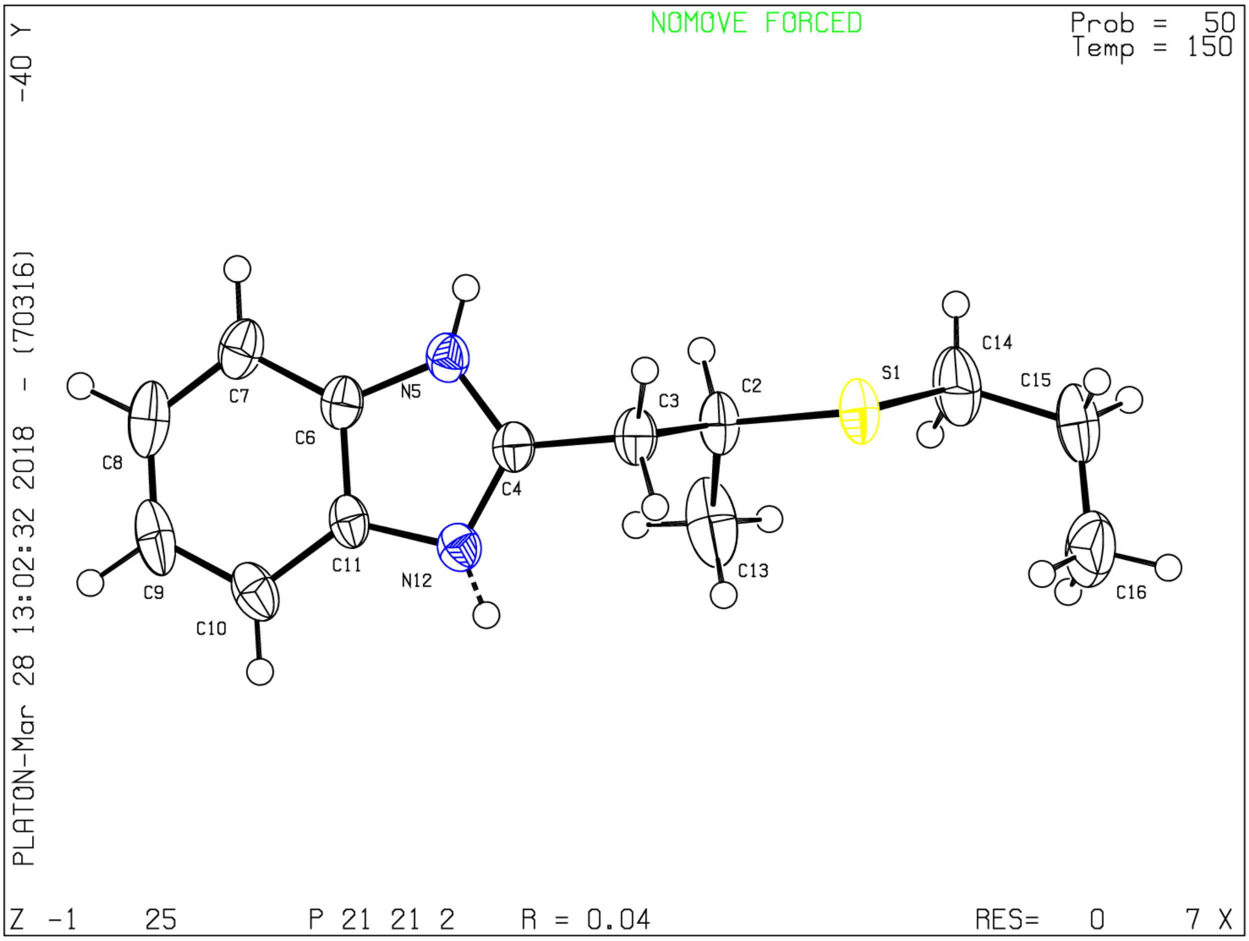

Table 1

Experimental details

Crystal data	
Chemical formula	$C_{13}H_{18}N_2S$
M_r	234.37
Crystal system, space group	Orthorhombic, $P2_12_12$
Temperature (K)	150
a, *b*, *c* (Å)	20.7206 (4), 9.8606 (2), 6.3486 (1)
V (Å^3)	1297.13 (4)
Z	4
Radiation type	Cu *K*α
μ (mm^{-1})	2.00
Crystal size (mm)	0.20 × 0.20 × 0.15
Data collection	
Diffractometer	Unknown
Absorption correction	Multi-scan<br>*DENZO*/*SCALEPACK* (Otwinowski & Minor, 1997)
T_{min}, T_{max}	0.42, 0.74

No. of measured, independent and observed [$I > 2.0\sigma(I)$] reflections	26964, 2686, 2630
R_{int}	0.047
$(\sin \theta/\lambda)_{max}$ (Å^{-1})	0.630
Refinement	
$R[F^2 > 2\sigma(F^2)]$, $wR(F^2)$, S	0.037, 0.108, 0.99
No. of reflections	2686
No. of parameters	155
No. of restraints	8
H-atom treatment	H atoms treated by a mixture of independent and constrained refinement
$\Delta\rho_{max}$, $\Delta\rho_{min}$ (e Å^{-3})	0.33, −0.34
Absolute structure	Flack (1983), 1104 Friedel-pairs
Absolute structure parameter	0.01 (2)

Computer programs: USER DEFINED DATA COLLECTION, USER DEFINED *CELL* REFINEMENT, USER DEFINED DATA REDUCTION, *SIR92* (Altomare *et al.*, 1994), *CRYSTALS*(Betteridge *et al.*, 2003), *CAMERON* (Watkin *et al.*, 1996).

Table 2

Selected geometric parameters (Å, º)

S1—C2	1.8212 (16)	C6—C11	1.394 (2)
S1—C14	1.821 (2)	C7—C8	1.377 (3)
C2—C3	1.538 (2)	C8—C9	1.388 (3)
C2—C13	1.511 (3)	C9—C10	1.383 (3)
C3—C4	1.490 (2)	C10—C11	1.398 (3)
C4—N5	1.332 (2)	C11—N12	1.389 (3)
C4—N12	1.338 (2)	C14—C15	1.527 (3)
N5—C6	1.390 (3)	C15—C16	1.491 (4)
C6—C7	1.398 (3)		
C2—S1—C14	104.02 (9)	C7—C6—C11	121.5 (2)
S1—C2—C3	104.10 (10)	C6—C7—C8	116.6 (2)
S1—C2—C13	113.04 (15)	C7—C8—C9	122.2 (3)
C3—C2—C13	112.10 (18)	C8—C9—C10	121.7 (3)
C2—C3—C4	113.54 (13)	C9—C10—C11	116.8 (2)
C3—C4—N5	123.70 (19)	C10—C11—C6	121.2 (2)
C3—C4—N12	123.28 (18)	C10—C11—N12	131.34 (19)
N5—C4—N12	113.02 (13)	C6—C11—N12	107.46 (18)
C4—N5—C6	106.14 (15)	C11—N12—C4	105.98 (15)
N5—C6—C7	131.08 (18)	S1—C14—C15	109.75 (16)
N5—C6—C11	107.40 (18)	C14—C15—C16	114.3 (2)

Table 3

Hydrogen-bond geometry (Å, º)

D—H···*A*	*D*—H	H···*A*	*D*···*A*	*D*—H···*A*
N5—H51···N5^{i}	0.85	1.98	2.792 (3)	161 (2)
N12—H121···N12^{ii}	0.86	1.98	2.801 (3)	162 (5)

Symmetry codes: (i) −*x*+1, −*y*+1, *z*; (ii) −*x*+1, −*y*, *z*.

Alert level C

PLAT250_ALERT_2_C Large U3/U1 Ratio for Average U(i,j) Tensor .... 2.6 Note

This alert is due to disorder in the structure.

14/ Computational Methods

The range-separated dispersion-corrected ωB97X-D density functional[i,ii] was used with the 6-31G(d) basis set to optimize geometries. Vibrational frequency calculations were carried out to confirm that stationary points were either minima or first-order saddle points on the potential energy surface, and to obtain thermal corrections to Gibbs free energies at 298.15 K (80 ˚C). Quasi-harmonic (QHA) corrections were applied to the computed vibrational entropies using a frequency cut-off value of 100.0 cm^{-1}, adopting the model proposed by Grimme.[iii] This was automated by the *GoodVibes* program.[iv]
Vibrational frequency calculations were carried out to confirm that stationary points were either minima or first-order saddle points on the potential energy surface, and to obtain thermal corrections to Gibbs free energies at 353.15 K (80 ˚C). Quasi-harmonic (QHA) corrections were applied to the computed vibrational entropies using a frequency cut-off value of 100.0 cm^{-1}, adopting the model proposed by Grimme.[v] This was automated by the *GoodVibes* program.[vi] Solvent effects were considered using the integral equation formalism variant of the polarizable continuum model (IEF-PCM)[vii,viii,ix,x,xi] with the SMD solvation model (solvent=ethanol).[xii] Density functional theory (DFT) calculations were carried out in *Gaussian 09*.[xiii]

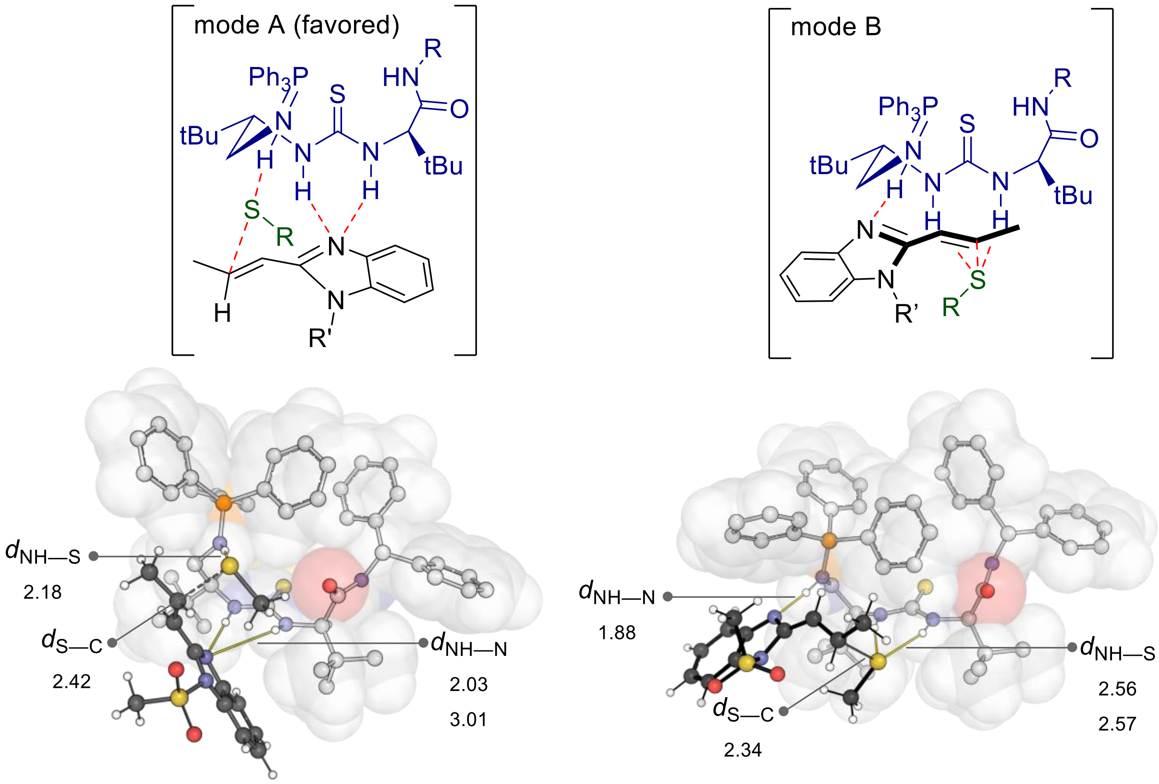

Figure S1. Comparison of 'mode A' and 'mode B' transition structures leading the major enantiomeric product. Mode A is favored by several kcal/mol using either wB97XD or M06-2X functionals.

Cartesian Coordinates (xyz format):

136

mode A TS (E = -4263.576613 H)

C -1.126728 -2.730766 0.944321

N -0.007387 -1.837566 1.158652

H 0.780734 -1.945485 0.532640

C	-0.070408	-0.683240	1.868412
S	-1.499399	-0.202053	2.666146
N	1.079709	0.015110	1.905089
H	1.931533	-0.424272	1.534365
C	1.218856	1.383877	2.378892
H	0.209980	1.712905	2.639152
H	1.177054	1.803527	-0.799539
C	1.723846	2.243898	1.210007
N	0.799005	2.236325	0.074436
H	2.670389	1.846266	0.837006
P	-0.253350	3.458853	-0.207747
C	0.566197	4.936354	-0.852937
C	1.793344	4.765370	-1.501702
C	-0.010260	6.208858	-0.765937
C	2.439858	5.863106	-2.059835
H	2.236992	3.776224	-1.580900
C	0.640958	7.301169	-1.328736
H	-0.960717	6.350653	-0.259584
C	1.864760	7.128364	-1.972947
H	3.391805	5.726403	-2.563381
H	0.193429	8.287805	-1.260857
H	2.369937	7.984361	-2.410275
C	-1.085940	3.897989	1.326436
C	-0.542886	4.844802	2.203309
C	-2.248565	3.204474	1.672516
C	-1.171035	5.097335	3.417503
H	0.363799	5.382982	1.942934
C	-2.871916	3.464548	2.887147
H	-2.656575	2.457664	0.999758

C	-2.334470	4.408630	3.757603
H	-0.750503	5.829007	4.100124
H	-3.770585	2.918908	3.157190
H	-2.821574	4.606533	4.707664
C	-1.425703	2.843536	-1.421769
C	-1.602746	1.465234	-1.570412
C	-2.159701	3.740274	-2.206745
C	-2.498445	0.987251	-2.520124
H	-1.044688	0.764517	-0.959343
C	-3.067875	3.252529	-3.136929
H	-2.016510	4.811812	-2.105244
C	-3.231175	1.877101	-3.297981
H	-3.639829	3.945498	-3.746135
H	-3.935674	1.499662	-4.032617
H	1.922933	3.264448	1.553324
H	-2.612067	-0.084998	-2.631836
C	4.395826	0.239232	-0.612983
C	4.325084	0.738685	-1.907251
H	4.458865	0.059279	-2.738135
S	2.026512	0.972250	-2.622057
C	1.560978	-0.652622	-1.953119
H	1.815967	-0.698977	-0.888969
H	0.486581	-0.829269	-2.050980
H	2.093273	-1.460360	-2.467630
C	4.778236	2.153659	-2.158641
H	4.409060	2.533697	-3.114849
H	4.446709	2.828039	-1.361084
H	5.876559	2.188426	-2.182412
H	4.345188	0.957356	0.202399

H	-1.708524	-2.736717	1.868994
C	-0.645207	-4.194452	0.694265
C	0.476026	-4.259236	-0.354288
C	-0.146052	-4.746991	2.035504
C	-1.826719	-5.050874	0.215769
H	0.198013	-3.732388	-1.273075
H	0.684573	-5.304749	-0.606805
H	-0.976077	-4.866669	2.742513
H	0.327575	-5.725314	1.896572
H	0.591703	-4.076988	2.485002
H	-1.523902	-6.103385	0.181216
H	-2.686570	-4.974515	0.891517
H	-2.155795	-4.769580	-0.790459
C	-2.030225	-2.225356	-0.189442
O	-1.589101	-1.880586	-1.282658
N	-3.352476	-2.259913	0.093343
H	-3.641653	-2.507899	1.029211
C	-4.389776	-2.071081	-0.906370
H	-3.901233	-2.238960	-1.871588
C	-5.446874	-3.150045	-0.708613
C	-6.477604	-2.984286	0.219011
C	-5.351047	-4.353335	-1.409710
C	-7.395995	-4.006444	0.442503
H	-6.569316	-2.046105	0.760410
C	-6.271714	-5.374806	-1.191384
H	-4.551686	-4.488962	-2.134304
C	-7.295275	-5.203939	-0.262058
H	-8.193006	-3.865888	1.167147
H	-6.188633	-6.304860	-1.746340

H	-8.015649	-5.998775	-0.091275
C	-4.977000	-0.665220	-0.922820
C	-5.878661	-0.333037	-1.938452
C	-4.641984	0.296226	0.027629
C	-6.433249	0.939126	-2.007164
H	-6.147024	-1.079670	-2.682257
C	-5.207479	1.570440	-0.035462
H	-3.920529	0.056633	0.805241
C	-6.098437	1.897632	-1.051179
H	-7.127575	1.184161	-2.806005
H	-4.944565	2.314032	0.711532
H	-6.530581	2.892852	-1.101349
C	2.096057	1.513612	3.660070
C	3.594654	1.363096	3.356574
C	1.837611	2.889860	4.294343
C	1.679639	0.433479	4.666794
H	3.809102	0.423986	2.833981
H	3.974986	2.189825	2.744248
H	4.165313	1.366288	4.293087
H	0.775907	3.021170	4.534224
H	2.408082	2.987637	5.225526
H	2.140717	3.716304	3.641202
H	2.208700	0.578700	5.616281
H	0.602655	0.468329	4.863615
H	1.920764	-0.568717	4.298034
C	4.233128	-3.356566	0.127027
C	3.703261	-2.698960	1.247792
C	3.258073	-3.427697	2.350191
C	3.347487	-4.813619	2.297535

C	3.850113	-5.459670	1.162710
C	4.293232	-4.743691	0.051867
C	4.209061	-1.090308	-0.135658
H	3.007400	-5.404712	3.142822
H	3.895461	-6.544344	1.138292
H	4.674714	-5.251316	-0.822662
N	4.569308	-2.314195	-0.770331
N	3.682330	-1.333614	1.056641
S	5.648001	-2.518305	-2.075242
O	6.023202	-3.921130	-2.080375
O	5.049549	-1.947742	-3.268510
C	7.057307	-1.554038	-1.585122
H	6.748886	-0.521778	-1.411348
H	7.472529	-1.994086	-0.676680
H	7.778550	-1.610164	-2.404295
H	1.415401	-3.838910	0.021475
H	2.850392	-2.914305	3.215950

136

mode B TS (E = -4263.566726 H)

C	-3.258044	-2.556884	0.620128
N	-1.905416	-2.054680	0.788823
H	-1.167320	-2.502433	0.246811
C	-1.519072	-1.189025	1.752088
S	-2.627440	-0.394029	2.784845
N	-0.187257	-0.987874	1.820879
H	0.385115	-1.518193	1.158066
C	0.510860	-0.416874	2.959182
H	-0.256140	-0.242268	3.717885
H	2.496952	0.746996	0.989758

C	1.100210	0.976633	2.626850
N	1.590894	1.177917	1.263098
H	1.913606	1.208443	3.321413
P	0.994389	2.281923	0.204486
C	2.392185	2.824495	-0.798397
C	3.611117	3.111229	-0.172446
C	2.277383	2.928932	-2.187341
C	4.705717	3.501048	-0.933179
H	3.712324	3.006244	0.902815
C	3.378989	3.316676	-2.943895
H	1.341398	2.693302	-2.683475
C	4.590444	3.599062	-2.318728
H	5.652373	3.709005	-0.444722
H	3.289192	3.389897	-4.023032
H	5.450470	3.893517	-2.913151
C	0.264526	3.677259	1.093564
C	0.964487	4.876099	1.255619
C	-1.005286	3.524898	1.667554
C	0.398510	5.913487	1.990752
H	1.941947	5.010432	0.803138
C	-1.555380	4.559770	2.414322
H	-1.560994	2.599343	1.539522
C	-0.855959	5.754292	2.573149
H	0.939178	6.847463	2.107297
H	-2.531241	4.430966	2.872287
H	-1.292127	6.564214	3.150026
C	-0.288611	1.660785	-0.895492
C	-0.351437	0.294612	-1.173505
C	-1.176673	2.547887	-1.517708

C	-1.319368	-0.188756	-2.046373
H	0.348070	-0.393880	-0.711889
C	-2.126630	2.059002	-2.405822
H	-1.136867	3.612301	-1.305040
C	-2.208323	0.691141	-2.656293
H	-2.829087	2.742965	-2.871242
H	-2.974496	0.304134	-3.320210
H	0.318479	1.712962	2.817476
H	-1.397536	-1.254071	-2.230346
C	3.055385	-0.783821	-1.635767
C	2.569397	-1.959322	-2.220435
H	3.236563	-2.813955	-2.232643
C	4.129095	-0.680388	-0.725777
S	1.144345	-3.070182	-0.734006
C	2.498169	-3.917796	0.137868
H	3.359015	-3.249686	0.258890
H	2.181225	-4.241696	1.133965
H	2.827304	-4.801183	-0.419121
C	1.677086	-1.853733	-3.429812
H	1.150693	-2.788442	-3.634961
H	0.941140	-1.051093	-3.320741
H	2.303400	-1.611667	-4.299105
H	2.393478	0.077060	-1.616461
H	-3.786791	-2.286147	1.536475
C	-3.318591	-4.105329	0.532432
C	-2.529705	-4.689035	-0.647319
C	-2.754968	-4.672665	1.843823
C	-4.797731	-4.500217	0.404343
H	-2.890707	-4.297999	-1.600638

H	-1.456270	-4.475696	-0.575167
H	-2.639791	-5.780303	-0.651354
H	-3.292544	-4.281348	2.716247
H	-2.846281	-5.764896	1.850790
H	-1.693919	-4.427698	1.964074
H	-4.901761	-5.590524	0.437709
H	-5.398166	-4.081007	1.221200
H	-5.224937	-4.156153	-0.543759
C	-3.947387	-1.832925	-0.548942
O	-3.730218	-2.086447	-1.730139
N	-4.819797	-0.877183	-0.148018
H	-4.953183	-0.733558	0.844427
C	-5.628807	-0.103160	-1.072036
H	-5.364441	-0.484950	-2.062271
C	-7.103502	-0.408814	-0.821565
C	-7.881075	0.372650	0.033762
C	-7.672808	-1.540672	-1.410870
C	-9.205576	0.029290	0.295370
H	-7.454063	1.259546	0.493899
C	-8.995973	-1.884168	-1.152319
H	-7.070867	-2.156796	-2.074683
C	-9.766858	-1.098489	-0.297341
H	-9.800832	0.647967	0.961058
H	-9.427100	-2.764464	-1.620684
H	-10.801404	-1.362355	-0.097242
C	-5.319026	1.385822	-1.038159
C	-5.817629	2.191962	-2.066560
C	-4.584558	1.978682	-0.013303
C	-5.592065	3.563696	-2.069282

H	-6.392759	1.737432	-2.870062
C	-4.366985	3.356059	-0.009356
H	-4.156488	1.367852	0.777227
C	-4.866833	4.152893	-1.033581
H	-5.984221	4.174106	-2.878003
H	-3.795198	3.806053	0.796417
H	-4.692242	5.225136	-1.028506
C	1.513229	-1.436285	3.578548
C	0.838944	-2.813324	3.676132
C	2.793838	-1.540502	2.748245
C	1.865709	-0.977006	5.001781
H	-0.128726	-2.748358	4.188349
H	0.668616	-3.258084	2.690121
H	1.475997	-3.501257	4.243860
H	3.385586	-0.622580	2.813319
H	3.426210	-2.359893	3.109100
H	2.581355	-1.723231	1.690667
H	2.582650	-1.672800	5.452715
H	2.326585	0.017080	5.019239
H	0.975846	-0.948257	5.642408
C	6.159261	-0.957318	0.268876
C	5.367394	-0.014816	0.941282
C	5.835847	0.592740	2.106339
C	7.096246	0.232131	2.573055
C	7.869056	-0.721222	1.901540
C	7.406246	-1.341677	0.740837
H	5.221613	1.317304	2.633862
H	7.483271	0.689948	3.479104
H	8.844852	-0.993700	2.292024

H	8.001148	-2.085079	0.227240
N	5.374747	-1.394688	-0.833156
N	4.141946	0.127996	0.323367
S	6.123259	-1.618246	-2.356619
O	7.503420	-1.979919	-2.080659
O	5.308181	-2.511146	-3.157531
C	6.073124	0.016707	-3.062366
H	5.027263	0.303640	-3.188455
H	6.594342	0.700469	-2.389339
H	6.580336	-0.034203	-4.028562

[i] A. D. Becke, Density-functional thermochemistry. V. Systematic optimization of exchange-correlation functionals. *J. Chem. Phys*. **107**, 8554–8560 (1997).

[ii] J.-D. Chai, M. Head-Gordon, Long-range corrected hybrid density functionals with damped atom–atom dispersion corrections. *Phys. Chem. Chem. Phys*. **10**, 6615–6620 (2008).

[iii] S. Grimme, Supramolecular binding thermodynamics by dispersion-corrected density functional theory. *Chem. Eur. J.* **18**, 9955–9964 (2012).

[iv] GoodVibes, version 2.0.1, I. Funes-Ardoiz, R. S. Paton, http://doi.org/10.5281/zenodo.595246 (accessed 13 April 2018).

[v] S. Grimme, Supramolecular binding thermodynamics by dispersion-corrected density functional theory. *Chem. Eur. J.* **18**, 9955–9964 (2012).

[vi] GoodVibes, version 2.0.1, I. Funes-Ardoiz, R. S. Paton, http://doi.org/10.5281/zenodo.595246 (accessed 13 April 2018).

[vii] E. Cancès, B. Mennucci, J. Tomasi, A new integral equation formalism for the polarizable continuum model: Theoretical background and applications to isotropic and anisotropic dielectrics. *J. Chem. Phys.* **107**, 3032–3041 (1997).

[viii] B. Mennucci, E. Cancès, J. Tomasi, Evaluation of solvent effects in isotropic and anisotropic dielectrics and in ionic solutions with a unified integral equation method: Theoretical bases, computational implementation, and numerical applications. *J. Phys. Chem. B* **101**, 10506–10517 (1997).

[ix] B. Mennucci, J. Tomasi, Continuum solvation models: A new approach to the problem of solute’s charge distribution and cavity boundaries. *J. Chem. Phys.* **106**, 5151–5158 (1997).

[x] J. Tomasi, B. Mennucci, E. Cancès, The IEF version of the PCM solvation method: an overview of a new method addressed to study molecular solutes at the QM ab initio level. *J. Mol. Struct. THEOCHEM* **464**, 211–226 (1999).

[xi] G. Scalmani, M. J. Frisch, Continuous surface charge polarizable continuum models of solvation. I. General formalism. *J. Chem. Phys.* **132**, 114110 (2010).

[xii] A. V. Marenich, C. J. Cramer, D. G. Truhlar, Universal solvation model based on solute electron density and on a continuum model of the solvent defined by the bulk dielectric constant and atomic surface tensions. *J. Phys. Chem. B* **113**, 6378–6396 (2009).

[xiii] Gaussian 09, Revision D.01, M. J. Frisch, G. W. Trucks, H. B. Schlegel, G. E. Scuseria, M. A. Robb, J. R. Cheeseman, G. Scalmani, V. Barone, G. A. Petersson, H. Nakatsuji, X. Li, M. Caricato, A. V. Marenich, J. Bloino, B. G. Janesko, R. Gomperts, B. Mennucci, H. P. Hratchian, J. V. Ortiz, A. F. Izmaylov, J. L. Sonnenberg, D. Williams-Young, F. Ding, F. Lipparini, F. Egidi, J. Goings, B. Peng, A. Petrone, T. Henderson, D. Ranasinghe, V. G. Zakrzewski, J. Gao, N. Rega, G. Zheng, W. Liang, M. Hada, M. Ehara, K. Toyota, R. Fukuda, J. Hasegawa, M. Ishida, T. Nakajima, Y. Honda, O. Kitao, H. Nakai, T. Vreven, K. Throssell, J. A. Montgomery, Jr., J. E. Peralta, F. Ogliaro, M. J. Bearpark, J. J. Heyd, E. N. Brothers, K. N. Kudin, V. N. Staroverov, T. A. Keith, R. Kobayashi, J. Normand, K. Raghavachari, A. P. Rendell, J. C. Burant, S. S. Iyengar, J. Tomasi, M. Cossi, J. M. Millam, M. Klene, C. Adamo, R. Cammi, J. W. Ochterski, R. L. Martin, K. Morokuma, O. Farkas, J. B. Foresman, D. J. Fox, Gaussian, Inc., Wallingford CT, 2009.
